# Substituent Effects on Cooperativity in Three-Component H-Bond Networks Involving Phenol–Phenol Interactions

**DOI:** 10.1021/jacs.4c15767

**Published:** 2024-12-18

**Authors:** Lucia Trevisan, Andrew D. Bond, Christopher A. Hunter

**Affiliations:** Yusuf Hamied Department of Chemistry, University of Cambridge, Lensfield Road, Cambridge CB2 1 EW, U.K.

## Abstract

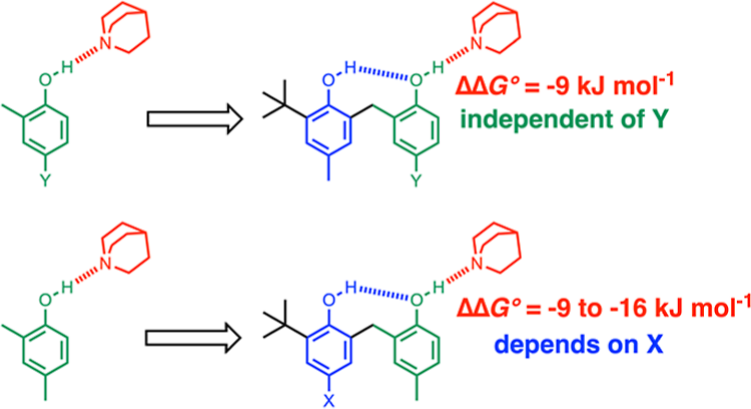

Cooperativity between H-bonding interactions in networks is a fundamental aspect of solvation and self-assembly in molecular systems. The interaction of a series of bisphenols, which make an intramolecular H-bond between the two hydroxyl groups, and quinuclidine was used to quantify cooperativity in three-component networks. The presence of the intramolecular H-bond in the bisphenols was established by using ^1^H NMR spectroscopy in solution and X-ray crystallography in the solid state. The interactions with quinuclidine were investigated using UV–vis and ^1^H NMR titrations, which show that the intramolecular hydrogen bonds persist in the 1:1 complexes. By varying substituents on one of the phenol groups, it was possible to measure the effect of changing the strength of the intramolecular H-bond between the hydroxyl groups on the strength of the intermolecular H-bond with quinuclidine. Strong positive cooperativity was observed between the two interactions, with increases in binding free energy of up to 16 kJ mol^–1^. By varying substituents on the other phenol group, which makes both an intramolecular H-bond and an intermolecular H-bond in the complex, it was possible to measure how the properties of this central hydroxyl group modulate cooperativity between the interactions with the other two functional groups. Changing the polarity of this phenol had no effect on the measured cooperativity. The results indicate that cooperativity in H-bond networks can be understood as a polar interaction between two remote functional groups that is damped by a central functional group. The extent of damping is quantified by cooperativity parameter κ, which is 0.33 for the hydroxyl group and appears to be an intrinsic property of the geometry or polarizability of the functional group rather than polarity.

## Introduction

Hydrogen bonds play a pivotal role in life as they determine the structure and function of a range of natural molecules such as proteins,^1^ DNA,^2^ and enzymes.^3^ Given the strong directional preference,^4^ H-bonding is one of the most important interactions in supramolecular chemistry^5^ and has been applied in synthetic systems for molecular recognition,^6^ catalysis,^7^ and materials.^8^ There is evidence that the H-bonding properties of functional groups can be dramatically changed if they form multiple H-bonds.^9−13^ The first experimental evidence of positive H-bond cooperativity for alcohols was gathered using infrared spectroscopy, and it was shown that the formation of a H-bond between an alcohol and a H-bond acceptor increases the strength of the H-bonding interaction with a second hydroxyl group.^14−16^ Cockroft and co-workers used molecular torsion balances to investigate cooperativity in chains of intramolecularly H-bonded hydroxyl groups.^17^

**Figure 1:**
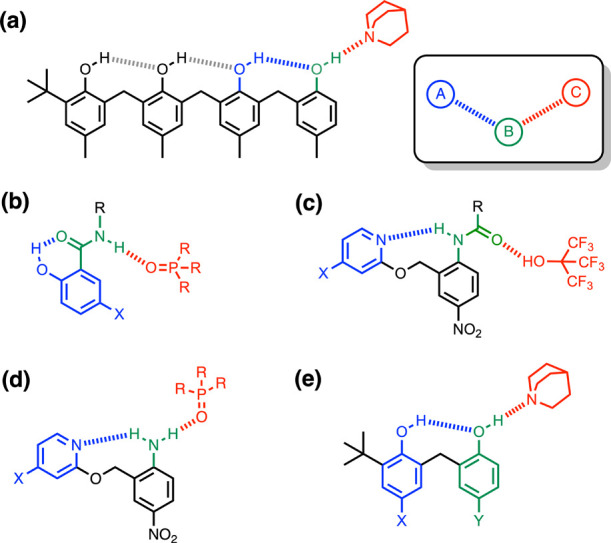
Complexes used to quantify cooperativity in H-bond networks by measuring the effect of intramolecular H-bonds between functional groups A (blue) and B (green) on the interaction with molecule C (red). Complexes described previously were used to measure (a) H-bond donor parameters for phenol oligomers, (b) H-bond donor parameters for secondary amides, (c) H-bond acceptor parameters for secondary amides, and (d) H-bond donor parameters for anilines. (e) Complexes used in this work to quantify the effects changing the H-bond properties of both the green and blue functional groups on the interaction with quinuclidine (red).

We have been investigating cooperative effects in H-bond networks by quantifying the effect of intramolecular H-bonds on intermolecular interactions that involve a second H-bond with the same functional group.^18−21^Figure 1 shows examples of different systems that have been studied to date. There is an intramolecular H-bond between the functional group highlighted in blue (A) and the functional group highlighted in green (B), which also makes an intermolecular H-bond with the red molecule (C). Phenol oligomers of different chain lengths were used to investigate positive cooperativity associated with H-bonding between multiple hydroxyl groups (Figure 1a).^18^ Variation in the X substituent was used to tune the H-bond donor or acceptor properties of the blue functional group in Figures 1b–d and hence modulate the strength of the intramolecular H-bond. The strength of the intermolecular H-bond formed with the phosphine oxide or perfluoro-*t*-butanol in the secondary amide complexes, as shown in Figure 1b and 1c, increased as the strength of the intramolecular interaction increased, i.e., positive allosteric cooperativity.^19,20^ In contrast, strength of the intermolecular H-bond formed with the phosphine oxide in the aniline complex, as shown in Figure 1d, decreased as the strength of the intramolecular interaction increased, i.e., negative allosteric cooperativity.^21^

The overall contribution of H-bond cooperativity to the stability of the complexes in Figure 1, ΔΔ*G*_coop_, could be quantitatively described as the product of the H-bond parameter for functional group A (either α_A_ or β_A_), the cooperativity parameter for functional group B (κ_B_), and the H-bond parameter for functional group C (either α_C_ or β_C_).^18^ If functional groups A and C were to interact in a pairwise complex, the free energy contribution from this H-bond would be the product of the H-bond parameters for A and C. Cooperativity in the three-component H-bond networks can be understood as an interaction between A and C (quantified by the product of the H-bond parameters for A and C) that is damped to different extents depending on the properties of B (quantified by κ_B_).

Experiments on the complexes in Figure 1a–d were used to determine cooperativity parameters for the phenol (0.33), secondary amide (0.20), and aniline (−0.10) functional groups.^18−21^ However, in all of these experiments, the central green functional group B was kept constant. Here, we extend this approach to the system, as shown in Figure 1e, where the X substituent is used to modulate the strength of the intramolecular H-bond, as in the other systems, but in addition, the Y substituent is used to modulate the H-bond donor and acceptor properties of the central functional group B. The results confirm the value of the cooperativity parameter κ_B_ as 0.33 for phenol hydroxyl groups. The polarity of the green OH group in Figure 1e has no effect on the cooperativity parameter, which appears to be an intrinsic property of the hydroxyl functional group and its ability to relay interactions between A and C, either via through-space interactions that are governed by spatial separation or via through-bond effects that are governed by polarizability.

## Results and Discussion

Two sets of bisphenols **1**–**5** and **6**–**9** were studied in order to determine the effect of the substituent on cooperativity in H-bonded phenols. In compounds **1**–**5**, the Y substituent is a methyl group and the X substituents (NO_2_, Br, F, CH_3_ and NMe_2_) modify the properties of the blue hydroxyl group. In compounds **6**–**9**, the X substituent is a methyl group, and the Y substituents (NO_2_, Br, F, CH_3_, and NMe_2_) modify the properties of the green hydroxyl group. Compounds **10**–**14** and **16**–**19** are reference monophenols used to quantify the H-bond donor properties of the hydroxyl groups in the absence of an intramolecular H-bond.

### Synthesis

**Scheme 1:**
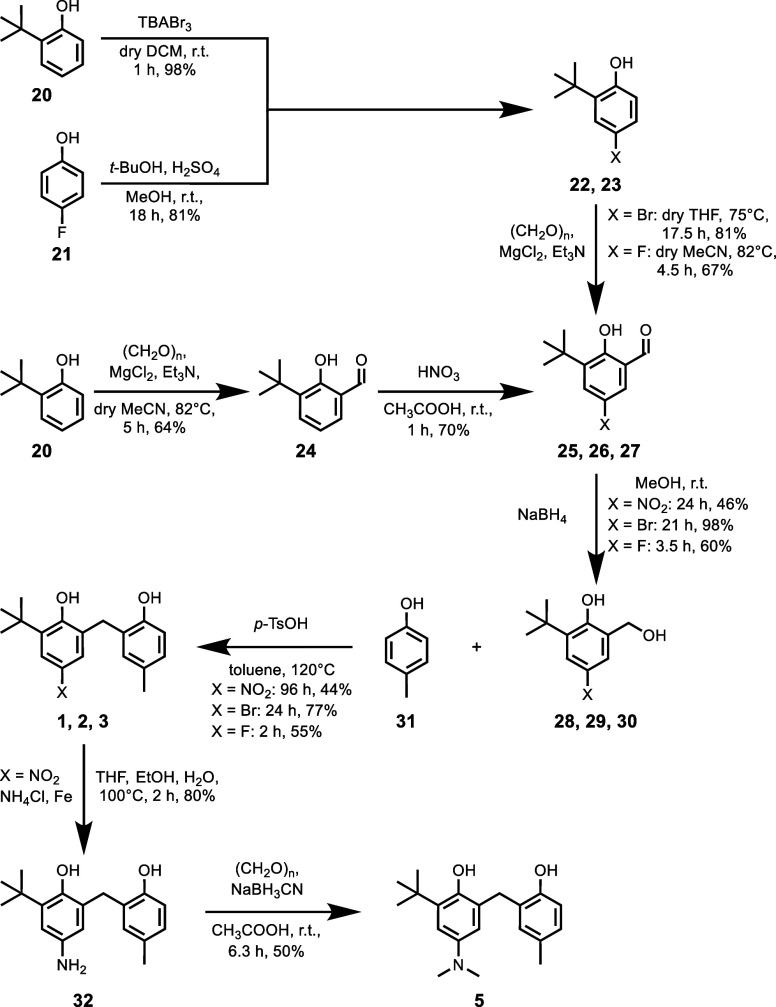
Synthesis of Bisphenols with Different X Substituents: 1 X = NO_2_, 2 X = Br, 3 X = F, and 5 X = NMe_2_

**Scheme 2:**
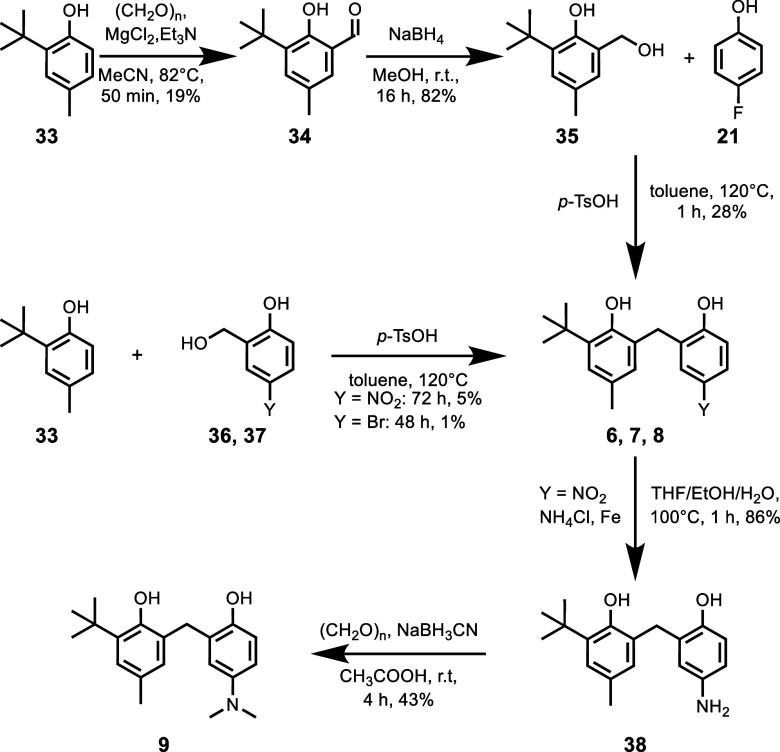
Synthesis of Bisphenols with Different Y Substituents: 6 X = NO_2_, 7 X = Br, 8 X = F, and 9 X = NMe_2_

The synthesis of bisphenols with different X substituents is shown in Scheme 1. Bisphenol **1** was synthesized through a condensation of benzyl alcohol **27** with 4-methylphenol **30**. Compound **27** was prepared starting with an *ortho*-formylation of 2-*t*-butylphenol **20**,^22^ followed by nitration,^23^ and reduction of the aldehyde with sodium borohydride.^24^ Compound **1** was also used as the starting point for the synthesis of **5** by reducing the nitro group and methylating the amino group. Compounds **2** and **3** were synthesized in a similar way through a condensation of the respective benzyl alcohols **28** (X = Br) and **29** (X = F) with 4-methylphenol **30**. Compound **28** was prepared by bromination of **20**,^25^ followed by *ortho*-formylation,^25^ and reduction.^22^ Similarly, **29** was synthesized from 4-fluorophenol **21**, which was *t*-butylated in the *ortho* position,^26^ followed by *ortho*-formylation, and reduction with sodium borohydride. Compound **4** was prepared, as previously reported.^18^ The synthesis of bisphenols with different Y substituents is shown in Scheme 2. Compound **6** was synthesized through condensation of 2-*t*-butyl-4-methylphenol **32** with 2-(hydroxymethyl)-4-nitrophenol **36**. Reduction of the nitro group of **6** followed by methylation gave **9**. Compound **7** was prepared by the condensation of 2-*t*-butyl-4-methylphenol **32** with 2-(hydroxymethyl)-4-bromophenol **37**. Finally, compound **8** was synthesized by condensation of benzyl alcohol **34** with 4-fluorophenol **35**.

Reference monophenols **13**, and **15**–**18** were commercially available, and the synthesis of the other monophenols **10**–**12**, **14**, and **19** is reported in the Supporting Information.

### Intramolecular H-Bonding Interactions

**Figure 2:**
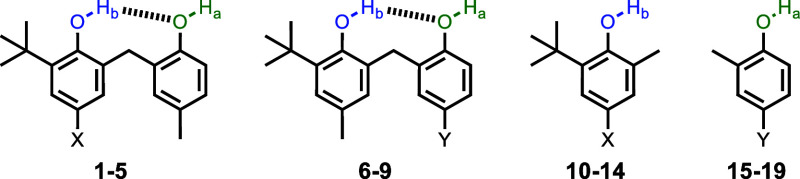
Chemical structures of bisphenols and reference monophenols: **1** and **10** (X = NO_2_), **2** and **11** (X = Br), **3** and **12** (X = F), **4** and **13** (X = Me), **5** and **14** (X = NMe_2_), **6** and **15** (Y = NO_2_), **7** and **16** (Y = Br), **8** and **17** (Y = F), **18** (Y = Me), and **9** and **19** (Y = NMe_2_). The ^1^H NMR labeling scheme for the hydroxyl groups is shown.

Bisphenol **4** (X = Y = Me) was previously characterized by ^1^H NMR spectroscopy and X-ray crystallography, and the blue hydroxyl group OH_b_ highlighted in Figure 2 was found to act as the H-bond donor in an intramolecular H-bond with the green hydroxyl group OH_a_ in both the solid state and solution (Figure 2).^18^ The ^1^H NMR spectra of bisphenols **1**–**8** recorded in CDCl_3_ all have two OH signals, but compound **9** was sparingly soluble in chloroform, and it was not possible to detect the OH signals. With the exception of compounds **5** and **7**, it was possible to unambiguously assign the two OH signals using 2D NMR spectroscopy (COSY, HSQC, and HMBC, see the Supporting Information). For compounds **1**–**4** (Y = Me), the signal due to OH_b_ is 1–3 ppm higher in chemical shift than the signal due to OH_a_ (Table 1), which implies that an intramolecular H-bond is present in all of these compounds in chloroform solution and that it is OH_b_ that acts as the H-bond donor.^27,28^ This conclusion is confirmed by comparison of the chemical shifts to those of the corresponding reference monophenols that are not affected by H-bonding interactions (Table 1). For compounds **1**–**4**, the chemical shifts of the signals due to OH_b_ are 2 ppm higher than the corresponding signals for **10**–**13**, suggesting that the former are involved in H-bonding. The chemical shifts of the signals due to OH_a_ are also higher than the corresponding signal for reference compound **18** (Y = Me, 4.5 ppm), but the differences are only 1 ppm.

**Table 1:** ^1^H NMR Chemical Shifts of the Signals Due to Phenol OH Groups in CDCl_3_ (ppm)

monophenols					bisphenols				
substituent		compound	signal		substituent		compound	signal	
X	Y		OH_b_	OH_a_	X	Y		OH_b_	OH_a_
NO_2_		10	5.5		NO_2_	Me	1	8.2	5.4
Br		11	4.7		Br	Me	2	6.9	5.2
F		12	4.5		F	Me	3	6.6	5.4
Me		13	4.6		Me	Me	4	6.5	5.5
NMe_2_		14	4.3		NMe_2_	Me	5^a^	5.6/5.9	5.6/5.9
	NO_2_	15		5.5	Me	NO_2_	6	5.7	7.8
	Br	16		4.6	Me	Br	7^a^	6.1/6.2	6.1/6.2
	F	17		4.5	Me	F	8	6.2	5.8
	Me	18		4.5	Me	Me	4	6.5	5.5
	NMe_2_	19		4.4	Me	NMe_2_	9	b	b

a For compounds **5** and **7**, the two OH signals could not be unambiguously assigned.

b Compound **9** is sparingly soluble in CDCl_3_, and the signals due to the OH groups were not detected.

**Figure 3:**
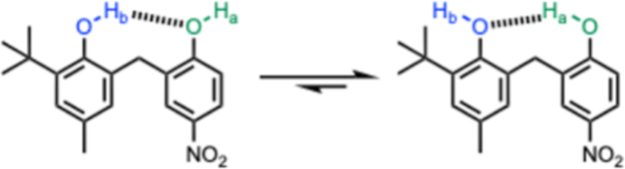
Preferred conformation for compound **6** in deuterochloroform involves OH_a_ as the donor in the intramolecular H-bond rather than OH_b_.

The behavior of the X = Me compounds is more complicated. For compound **6**, the chemical shift of OH_a_ (7.8 ppm) is 2 ppm higher than OH_b_ (5.7 ppm) and 2 ppm higher than the corresponding signal for reference monophenol **15**, which means that in this case it is OH_a_ that acts as the H-bond donor in the intramolecular H-bond (Figure 3). The 4-nitrophenol is a much better H-bond donor than OH_b_ in this compound, which presumably accounts for the difference in the conformation of this bisphenol. For compound **8**, the signal due to OH_b_ is at a higher chemical shift than OH_a_, but the difference is only 0.4 ppm. However, the chemical shift of the signal due to OH_b_ is 1.7 ppm higher than the corresponding signal for reference compound **17**, whereas for OH_a_ the difference compared with reference compound **13** is only 0.3 ppm. These results indicate that it is OH_b_ that is the H-bond donor in the intramolecular H-bond in compound **8**.

**Figure 4:**
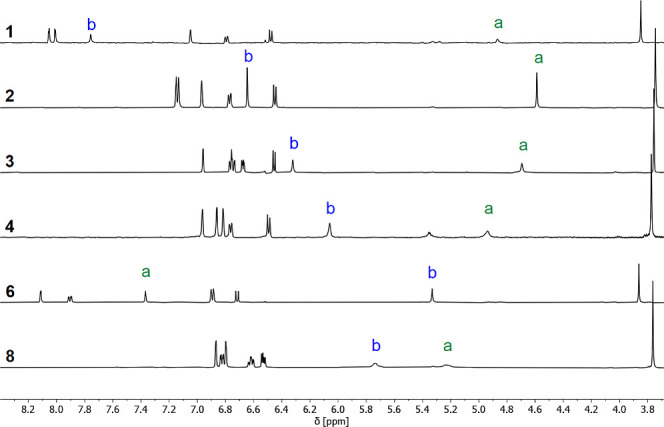
Partial 500 MHz ^1^H NMR spectra of **1** (0.14 mM), **2** (0.21 mM), **3** (0.24 mM), **4** (0.24 mM), **6** (0.15 mM), and **8** (0.24 mM) recorded at 298 K in *n*-octane with WET solvent suppression. Signals due to hydroxyl protons are labeled according to the scheme in Figure 2.

^1^H NMR spectra were also recorded in *n*-octane using WET solvent suppression.^29^^1^H NMR dilution experiments in *n*-octane show that there is no self-association of any of the compounds at millimolar concentrations (see the Supporting Information), and the spectra were very similar to those recorded in deuterochloroform, allowing direct assignment of the OH signals for compounds **1**–**4**, **6**, and **8**. Figure 4 shows that, with the exception of compound **6**, the signal due to OH_a_ appears at a lower chemical shift (<5.4 ppm) than the signal due to OH_b_, which confirms that OH_b_ is the donor in the intramolecular H-bond for compounds **1**–**4** and **8** in *n*-octane (Figure 2). For compound **6**, this pattern is reversed showing that OH_a_ is the donor in the intramolecular H-bond in both *n*-octane and deuterochloroform (Figure 3).

**Figure 5:**
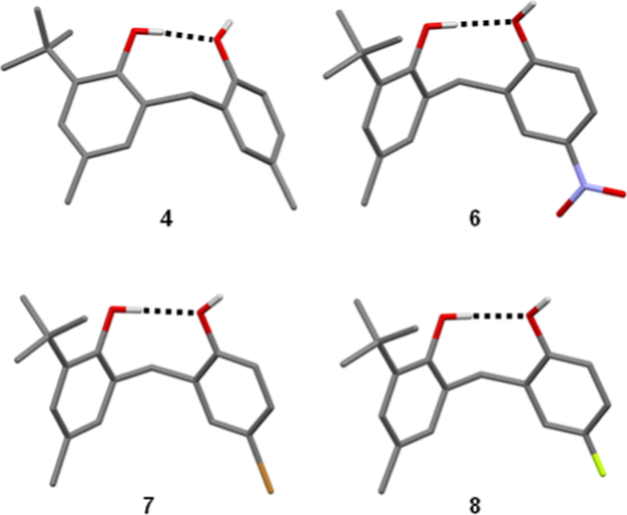
Molecular structures taken from the X-ray crystal structures of **4**, **6**, **7**, and **8**. Intramolecular H-bonding interactions are shown as dotted lines. For **4**, **6**, and **8**, the hydrogen atom positions are firmly established. For **7**, they are inferred from the similarity between the crystal structures of **4**, **7**, and **8** (see the text). The structure of **4** was reported previously (CSD REF code WEPTOE).^18^

X-ray crystal structures were obtained for compounds **4**,^20^**6**, **7**, and **8** (Figure 5). For compounds **4**, **6**, and **8**, the hydrogen atoms of the hydroxyl groups were located and refined freely, clearly identifying OH_b_ as the donor in the intramolecular H-bond. For compound **7**, the hydrogen atoms could not be confidently located on the electron density map. While the network of intermolecular H-bonding interactions in the crystal lattice could be consistent with either OH_a_ or OH_b_ acting as the donor in the intramolecular H-bond, comparisons with **4** and **8** suggest that the intramolecular donor in **7** is most likely to be OH_b_. The crystal structure of **7** is isomorphous with that of **4**, and the crystal structure of **8** contains identical one-dimensional H-bonded chains. Given the similarity in the crystal structures, it seems reasonable to infer that **7** should adopt the same conformation as **4** and **8** in the solid state. Interestingly, the intramolecular bond observed for compound **6** in the solid state is different from that in solution. In solution, OH_a_ acts as the intramolecular H-bond donor (Figure 3), but in the crystal structure, OH_b_ is the intramolecular H-bond donor, and OH_a_ is involved in an intermolecular H-bond to the nitro group of a neighboring molecule.

### Intermolecular H-Bonding Interactions

The formation of intermolecular H-bonds with quinuclidine in *n*-octane was investigated by using ^1^H NMR and UV–vis absorption titrations. For most of compounds **1**–**19**, the addition of quinuclidine led to the appearance of a blue-shifted band in the UV–vis spectrum, which is characteristic of formation of a H-bond.^18^ The UV–vis titration data fit well to a 1:1 binding isotherm, and the resulting association constants are reported in Table 2 (see the Supporting Information for details). For compounds **5**, **9**, **14**, and **19**, the changes in the UV–vis spectrum were too small to obtain reliable association constants, so the interaction with quinuclidine was quantified using ^1^H NMR titrations. For compounds where association constants could be determined by both ^1^H NMR and UV–vis absorption titrations, the results were consistent.^18^ The changes in the ^1^H NMR chemical shift observed on the addition of quinuclidine to the phenols fit well to a 1:1 binding isotherm, and the resulting association constants are reported in Table 2 (see the Supporting Information for details).

**Table 2:** Association Constants for Formation of 1:1 Complexes with Quinuclidine in *n*-Octane at 298 K^a^

monophenols				bisphenols			
X	Y	compound	*K*/M^–1^	X	Y	compound	*K*/M^–1^
NO_2_		10	490 ± 20	NO_2_	Me	1	(1.1 ± 0.3) × 10^5^
Br		11	93 ± 14	Br	Me	2	(1.9 ± 0.1) × 10^4^
F		12	78 ± 9	F	Me	3	(1.8 ± 0.2) × 10^4^
Me		13^b^	20 ± 6	Me	Me	4^b^	(9.1 ± 0.3) × 10^3^
NMe_2_		14	13 ± 4	NMe_2_	Me	5	(1.0 ± 0.1) × 10^4^
	NO_2_	15	8300 ± 100	Me	NO_2_	6	(1.2 ± 0.7) × 10^5^
	Br	16	820 ± 40	Me	Br	7	(3.3 ± 0.2) × 10^4^
	F	17	610 ± 10	Me	F	8	(1.6 ± 0.1) × 10^4^
	Me	18^b^	180 ± 10	Me	Me	4^b^	(9.1 ± 0.3) × 10^3^
	NMe_2_	19	140 ± 20	Me	NMe_2_	9	(7.0 ± 0.5) × 10^3^

a Errors are the standard error of the mean of three independent UV–vis absorption or ^1^H NMR titrations.

b These values were reported previously.^18^

**Figure 6:**
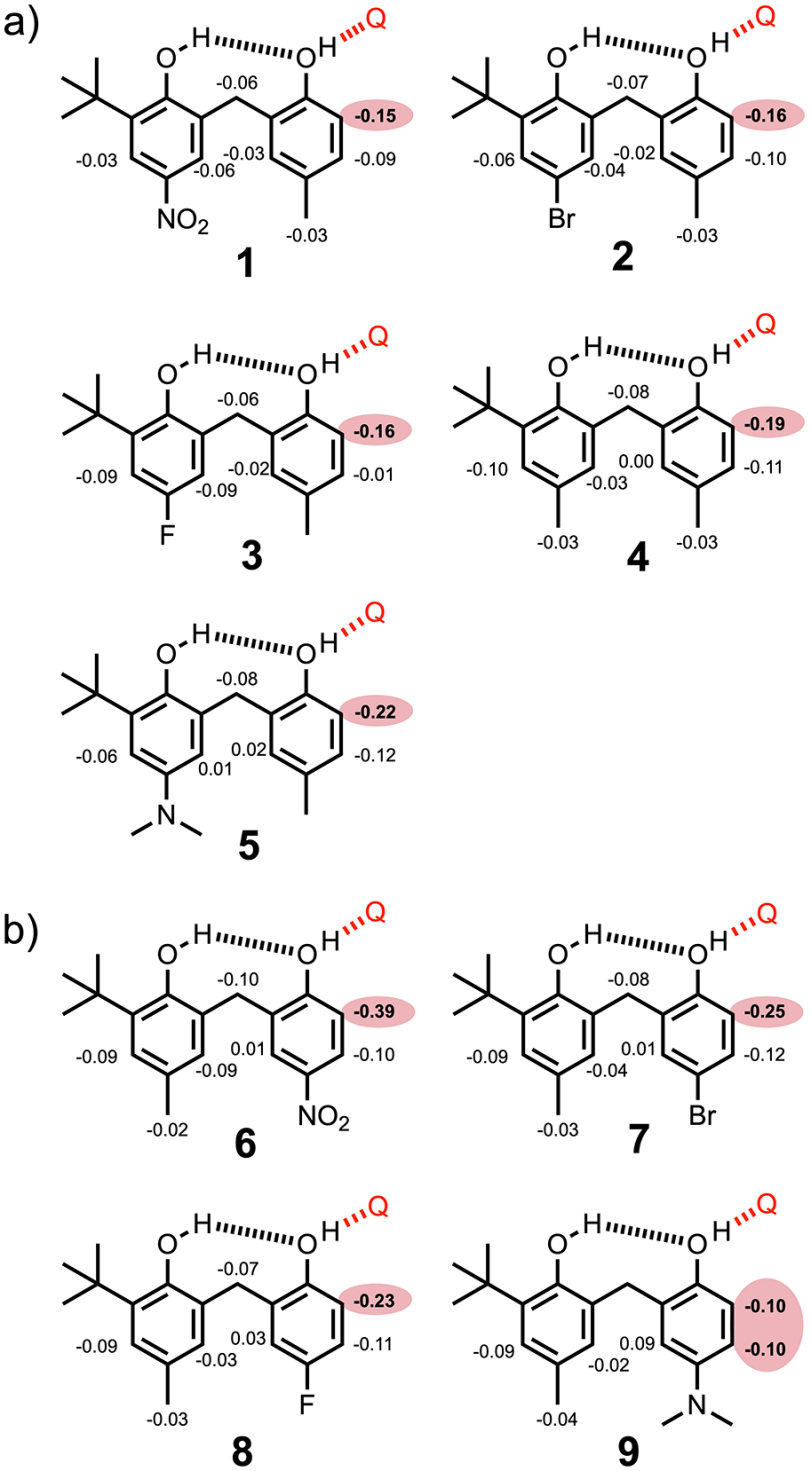
Limiting complexation-induced changes in ^1^H NMR chemical shift (ppm) for the formation of 1:1 complexes with quinuclidine (shown as a red Q) in *n*-octane at 298 K. The signal with the largest change in the chemical shift is highlighted in red in each case. The signals due to the methylene group and dimethylamino group on compound **9** were too broad to monitor in the titration.

The complexation-induced changes in the chemical shift for compound **5** obtained from the ^1^H NMR titration provide useful information on the site of interaction with quinuclidine. The signals due to the OH protons were too broad to detect on addition of quinuclidine, so it was not possible to measure complexation-induced changes in the chemical shift for these signals. The results for the CH signals are listed in Figure 6. A significantly larger change in the chemical shift was observed for the CH proton *ortho* to OH_a_ than that for any other signal. This result confirms that quinuclidine interacts with OH_a_ and that the intramolecular H-bond involving OH_b_ as the donor and OH_a_ as the acceptor remains intact in the complex. ^1^H NMR titrations with quinuclidine were therefore carried out for all of the bisphenol compounds in *n*-octane, and in all cases the pattern of complexation-induced changes in chemical shift was very similar to the largest change observed for the CH proton *ortho* to OH_a_ (highlighted in red in Figure 6). This result indicates that the intermolecular H-bond with quinuclidine is formed by OH_a_ in all cases, including compound **6**, where OH_a_ acts as the donor in the intramolecular H-bond with OH_b_ in the free state in *n*-octane solution, i.e., compound **6** undergoes a conformational change on binding quinuclidine. The association constant measured for **6** therefore includes a contribution due to the conformational change as well as the interaction of OH_a_ with quinuclidine, which means that this compound cannot be used to quantify cooperativity and will not be considered in the analysis below.

The association constants measured for the two sets of reference monophenols **10**–**14** and **15**–**19** show the expected substituent effects: electron-withdrawing groups make the phenol a better H-bond donor. A large steric effect is also apparent: compounds **10**–**14** have an additional *t*-butyl group *ortho* to the phenol OH, and the association constants are all an order of magnitude lower than the corresponding values for compounds **15**–**19**. The association constants measured for bisphenol compounds **1**–**9** are at least an order of magnitude higher than the values for the corresponding monophenol reference compounds. This result shows that the intramolecular H-bonding interaction between the two hydroxyl groups in the bisphenol compounds leads to a significant increase in the strength of the intermolecular H-bond formed with quinuclidine. The magnitude of this cooperative effect can be quantified by using the association constants in Table 2 to determine the H-bond donor parameters for the phenol OH groups.

### H-Bond Donor Parameters

The association constant for the formation of a H-bonded complex can be written in terms of the H-bond parameters for the H-bond donor, α, the H-bond acceptor, β, and the solvent, α_S_ and β_S_ (eq 1).^30^
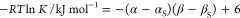
1

Using literature values for the solvent (α_S_ = 1.2, β_S_ = 0.6)^31^ and the H-bond acceptor quinuclidine (β = 9.0),^32^ and the experimental values of the association constant from Table 2 in eq 2 give the H-bond donor parameter α for the corresponding phenol group.
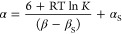
2

The results are listed in Table 3. The association constant for the complex formed with compound **6** includes a contribution due to the conformational change illustrated in Figure 3, so it is not possible to use eq 2 to obtain an accurate description of the H-bond donor properties of OH_a_ in this compound. The cooperativity associated with formation of the H-bond network in the other quinuclidine·bisphenol complexes can be quantified by comparing the H-bond donor parameter for OH_a_ of the bisphenol with the value for OH_a_ in the corresponding reference monophenol: compound **18** is the reference compound for the Y = Me bisphenols **1**–**5**, and compounds **16**–**19** are the reference compounds for the X = Me bisphenols **5**–**9**. The change in the H-bond donor parameter due to the intramolecular H-bond, Δα, is tabulated in Table 3.

**Table 3:** Experimentally Determined H-Bond Donor Parameters (α)

monophenols				bisphenols				
X	Y	compound	α	X	Y	compound	α	Δα
NO_2_		10	3.7	NO_2_	Me	1	5.3	1.9
Br		11	3.3	Br	Me	2	4.8	1.4
F		12	3.2	F	Me	3	4.8	1.3
Me		13^b^	2.8	Me	Me	4	4.6	1.2
NMe_2_		14	2.7	NMe_2_	Me	5	4.6	1.2
	NO_2_	15	4.6	Me	NO_2_	6^a^		
	Br	16	3.9	Me	Br	7	5.0	1.1
	F	17	3.8	Me	F	8	4.8	1.0
	Me	18	3.5	Me	Me	4^b^	4.6	1.2
	NMe_2_	19	3.4	Me	NMe_2_	9	4.5	1.2

a There is a change in conformation when **6** binds quinuclidine, which precludes accurate determination of the value of α for OH_a_ in this compound.

For the X = Me series, the donor involved in the intramolecular H-bond (OH_b_) is the same in all cases (α = 3.5), and the value of Δα for the donor involved in the intermolecular H-bond (OH_a_) is the same for all of the bisphenols (1.0–1.2). This result implies that the magnitude of the cooperative enhancement of the intermolecular H-bond is independent of the intrinsic H-bond donor properties of the OH group involved in the interaction with quinuclidine (OH_a_). In the reference monophenols, when the Y substituent is changed from the most electron-donating group NMe_2_ to the most electron-withdrawing group Br, the value of α for OH_a_ increases from 3.4 to 3.9. This change is exactly paralleled in bisphenols **1**–**5**, where the value of α for OH_a_ increases from 4.5 to 5.0 when the X substituent is changed.

For the Y = Me series (**1**–**5**), the value of Δα for the donor involved in the intermolecular H-bond (OH_a_) increases with the polarity of the donor involved in the intramolecular H-bond (OH_b_). This result implies that the magnitude of the cooperative enhancement of the intermolecular H-bond is a function of the intrinsic H-bond properties of the OH donor involved in the intramolecular H-bond (OH_b_). These observations are consistent with the relationship published previously, which was based on analysis of cooperativity in chains of H-bonded phenols (eq 3).^18^
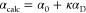
3where α_calc_ is the H-bond donor parameter for a phenol involved in an intramolecular H-bond with another phenol, which has a H-bond donor parameter α_D_, α_0_ is the H-bond donor parameter for the corresponding reference phenol that is not involved in an intramolecular H-bond, and the cooperativity parameter, κ, is 0.33 for a phenol OH group.

**Figure 7:**
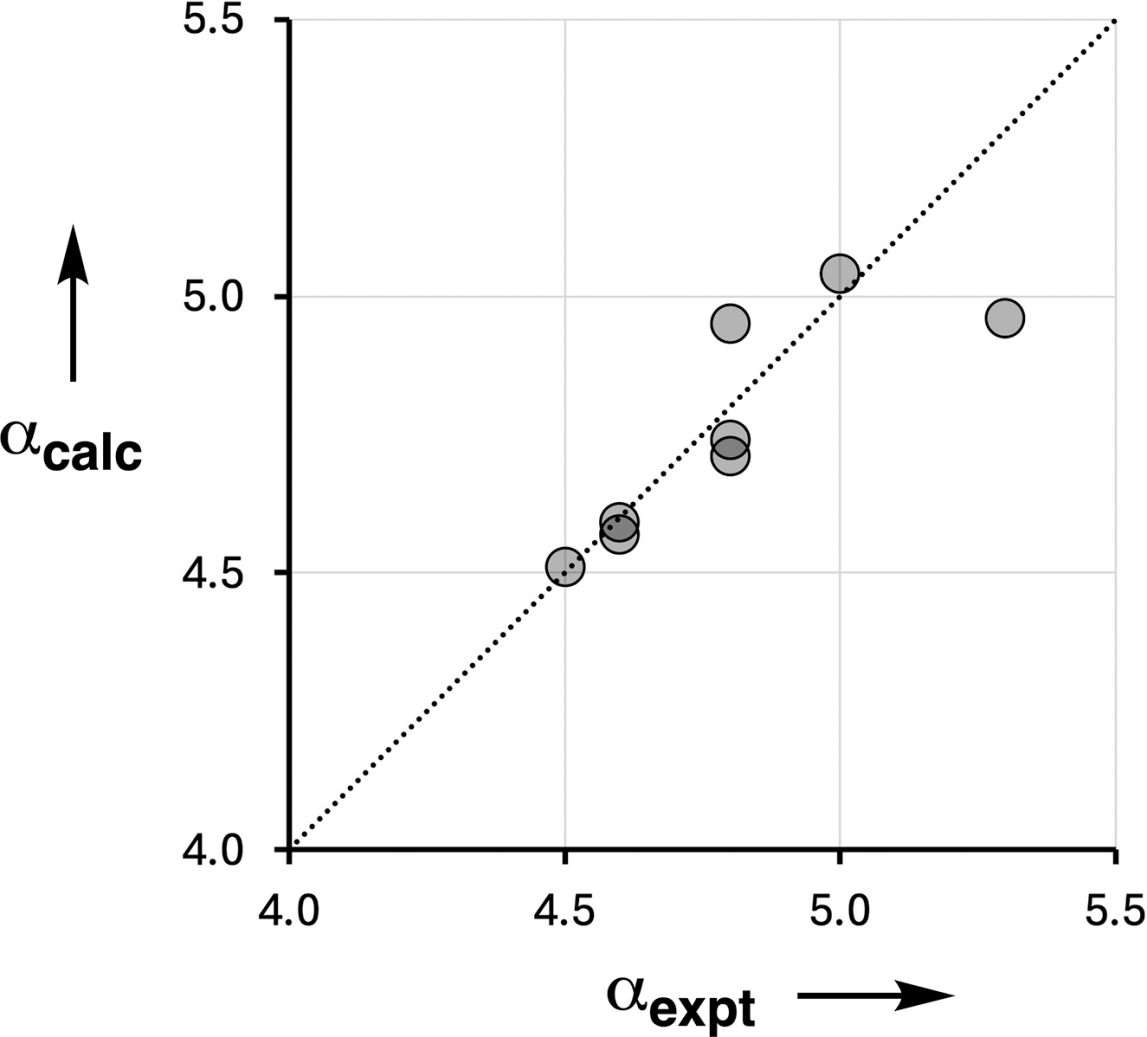
Relationship between the experimentally measured H-bond donor properties of bisphenols **1**–**5** and **7**–**9** (α_expt_) and the values calculated using eq 3 (α_calc_). The line is *y* = *x* (RMSE = 0.1).

Equation 3 was used to predict values of α_calc_ for compounds **1**–**9** using κ = 0.33. For compounds **1**–**5**, the experimentally determined value of α for compound **18** was used as α_0_, and the experimental values of α for compounds **15**–**19** were used as α_D_. The values of α measured for compounds **10**–**14** are not suitable reference points for estimating α_D_ because the intermolecular interactions with quinuclidine are perturbed by steric effects, as explained above. For compounds **6**–**9**, the experimental value of α for compound **18** was used as α_D_, and the experimental values of α for compounds **15**–**19** were used as α_0_. Figure 7 shows the relationship between α_calc_ and the values of the H-bond donor parameters measured in the cooperative H-bond networks in the bisphenol complexes of quinuclidine (α_expt_). With the exception of compound **1**, which is underpredicted by 0.3, the agreement is excellent (RMSE = 0.1).

**Figure 8:**
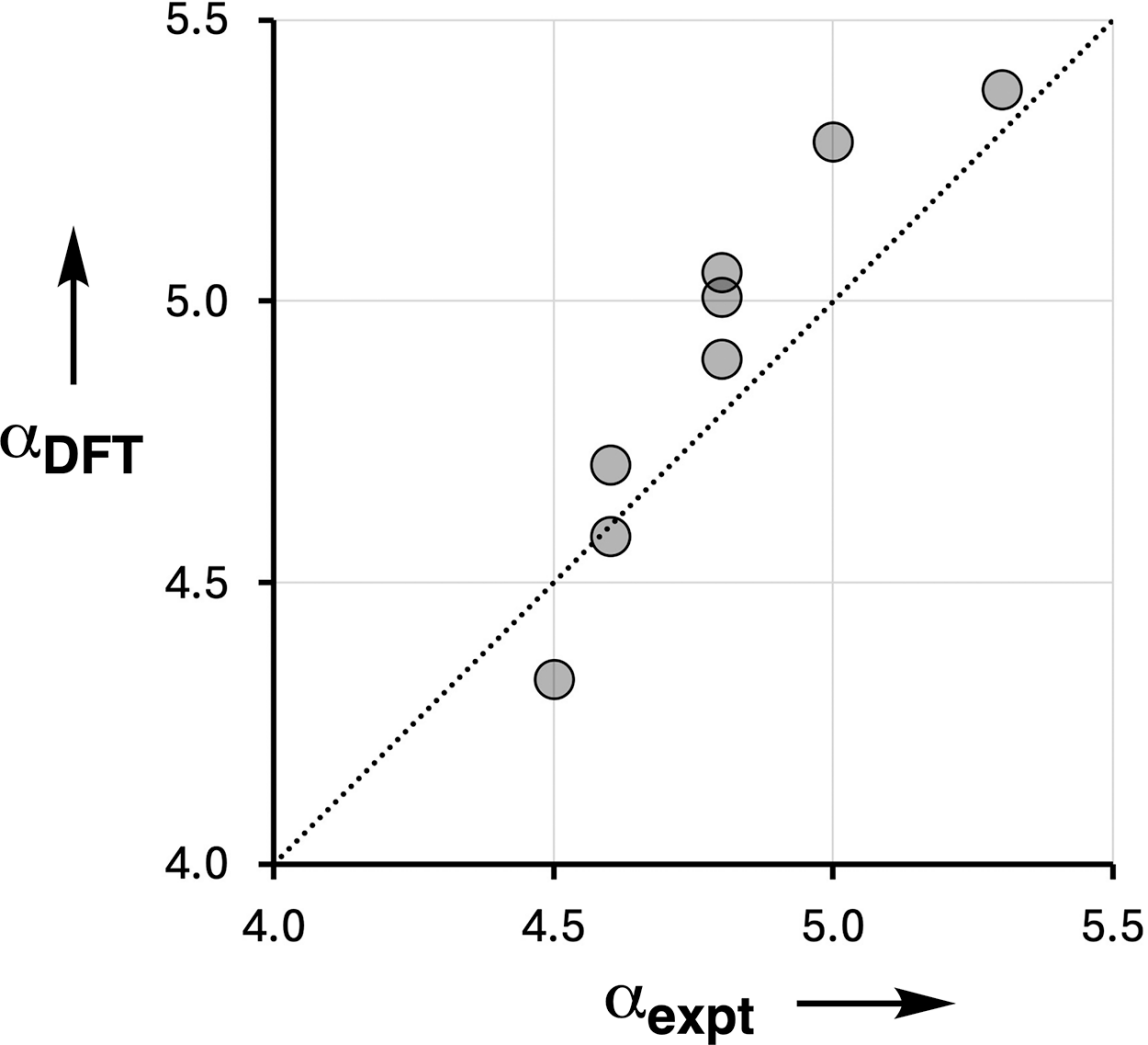
Relationship between the experimentally measured H-bond donor properties of bisphenols **1**–**5** and **7**–**9** (α_expt_) and the values calculated using DFT (α_DFT_). The line is *y* = *x* (RMSE = 0.2).

It is also possible to predict H-bond donor parameters from the maximum in the molecular electrostatic potential calculated on the van der Waals surface using density functional theory (DFT).^35^Figure 8 compares the computational parameters (α_DFT_) with the experimental results (see the Supporting Information for details). The agreement is good (RMSE = 0.2), which shows that gas phase ab initio calculations of molecular electrostatic potential of the isolated molecules provide an accurate description of the effects of H-bond cooperativity on the free energies of solution phase interactions. This result confirms that cooperativity can be understood in terms of the H-bonding properties of the individual components and does not require a more elaborate treatment of the multicomponent network.

## Conclusions

The supramolecular approach previously developed^18^ was used to study substituent effects in H-bond networks. The presence of an intramolecular H-bond between the two hydroxyl groups in a series of bisphenols was established by NMR spectroscopy and X-ray crystallography. The intramolecular hydrogen bonds persist when 1:1 complexes are formed with quinuclidine in *n*-octane. The association constants measured for the bisphenols are all orders of magnitude larger than the corresponding values measured for the quinuclidine complexes with reference monophenols that do not have an intramolecular H-bond. The increases in binding free energy can be as high as 16 kJ mol^–1^, which corresponds to 3 orders of magnitude increase in the association constant. The results were used to determine H-bond donor parameters α for the phenol hydroxyl group involved in the intermolecular H-bond with quinuclidine. The presence of the intramolecular H-bond increases the value of the H-bond donor parameter by between 1.0 and 1.9, i.e., by as much as 50% of the value for the corresponding monophenol reference compounds (α = 3.4–4.6). The experimental results are consistent with theoretical values for H-bond parameters obtained from molecular electrostatic potential surface calculated in the gas phase using ab initio methods.

The key feature of these experiments is the use of substituents to modulate the properties of both of the hydroxyl groups involved in the intramolecular H-bond. The strength of the intermolecular H-bond with quinuclidine increases in proportion to the polarity of the hydroxyl group that acts as the donor in the intramolecular H-bond. In contrast, changing the polarity of the hydroxyl group that acts as the acceptor in the intramolecular H-bond and the donor in the intermolecular H-bond had no effect on the magnitude of the cooperativity measured in the H-bond network. The results show that cooperativity in three-component H-bond networks depends on the ability of the central functional group to modulate interactions between the other two functional groups that do not make direct contact with one another. Thus, the overall contribution of H-bond cooperativity to the stabilities of these complexes can be quantitatively described as the product of the H-bond parameters for the acceptor in the intermolecular interaction and the donor in the intramolecular interaction and a cooperativity parameter that describes the extent to which the central hydroxyl group dampens this interaction. The fact that cooperativity in this system is independent of the polarity of this central hydroxyl group suggests that the cooperativity parameter is an intrinsic property of the hydroxyl functional group and its ability to relay interactions between A and C, either via through-space interactions that are governed by spatial separation or via through-bond effects that are governed by polarizability. The results suggest that it should be possible to develop simple models to account for cooperative effects in more complex systems, provided cooperativity parameters are known for the interacting functional groups.

## Supporting Information Available

Materials and methods, detailed synthetic procedures, full characterization including ^1^H and ^13^C NMR spectra of all compounds, details of X-ray crystallography, NMR experiments, and UV–vis absorption titrations and ab initio calculation of H-bond parameters (PDF)
