# Supplementary material for: Substituent Effects on Cooperativity in Three-Component H-Bond Networks Involving Phenol–Phenol Interactions
Source: J Am Chem Soc. 2024 Dec 18;147(1):1319–26. doi: 10.1021/jacs.4c15767 (PMC11726576; doi:10.1021/jacs.4c15767)
Supplement: Supplementary file 1 — ja4c15767_si_001.pdf [file ja4c15767_si_001.pdf]

Supporting Information

Substituent effects on cooperativity in three-component H-bond networks involving phenol-phenol interactions.

Lucia Trevisan, Andrew D. Bond and Christopher A. Hunter*

Yusuf Hamied Department of Chemistry, University of Cambridge, Lensfield Road, Cambridge, CB2 1 EW, UK.

Table of contents **Page**

**Materials and Methods**	S3
**Synthesis**	S4
**NMR Characterisation**	S32
**Single-Crystal X-ray Diffraction**	S118
**NMR Experiments**	S124
**UV-Vis experiments**	S148
**Details of computational study**	S176
**References**	S182

1. Materials and Methods

All reagents were purchased from commercial sources (Sigma Aldrich UK, Acros, Fluorochem) and were used as received without any further purification. Dry solvents were obtained by means of a Grubbs solvent system.

Flash chromatography was done with an automated system (Combiflash Companion) using pre-packed cartridges of silica (50 µm PuriFlash® column) or reverse phase C18HP (15 µm, PuriFlash® column).

The LC-MS analysis of samples was performed using Waters Acquity H-class UPLC coupled with a single quadrupole Waters SQD2. ACQUITY UPLC CSH C18 Column, 130 Å, 1.7 µm, 2.1 mm X 50 mm was used as the UPLC column for all samples. The conditions of the UPLC method are as follows: Solvent A: Water +0.1% Formic acid; Solvent B: Acetonitrile +0.1% Formic acid; Gradient of 0-2 minutes 5% -100%B + 1 minute 100% B with re-equilibration time of 2 minutes. Flow rate: 0.6 ml/min; column temperature of 40°C; injection volume of 2 µL. The signal was monitored with MS-ES^+, MS ES^-, at 254nm or at 290 nm.

^{1}H-NMR and ^{13}C-NMR were recorded on a 400 MHz, 500 MHz, 600 MHz or 700 MHz Bruker spectrometer as indicated. The reference values used for the chemical shifts of the various spectra are reported in the literature.[1] The splitting pattern is indicated with the following abbreviations: s for singlet, br s for broad singlet, d for doublet, t for triplet, q for quartet, quint for quintet, m for multiplet, dd for doublet of doublets and dt for doublet of triplets.

FT-IR spectra were collected with an ALPHA FT-IR Spectrometer from Bruker.

UV-Vis spectra were recorded with a UV-Vis Cary 60 spectrophotometer (Agilent).

Melting points were recorded with a Mettler Toledo MP90 melting point apparatus.

2. Synthesis

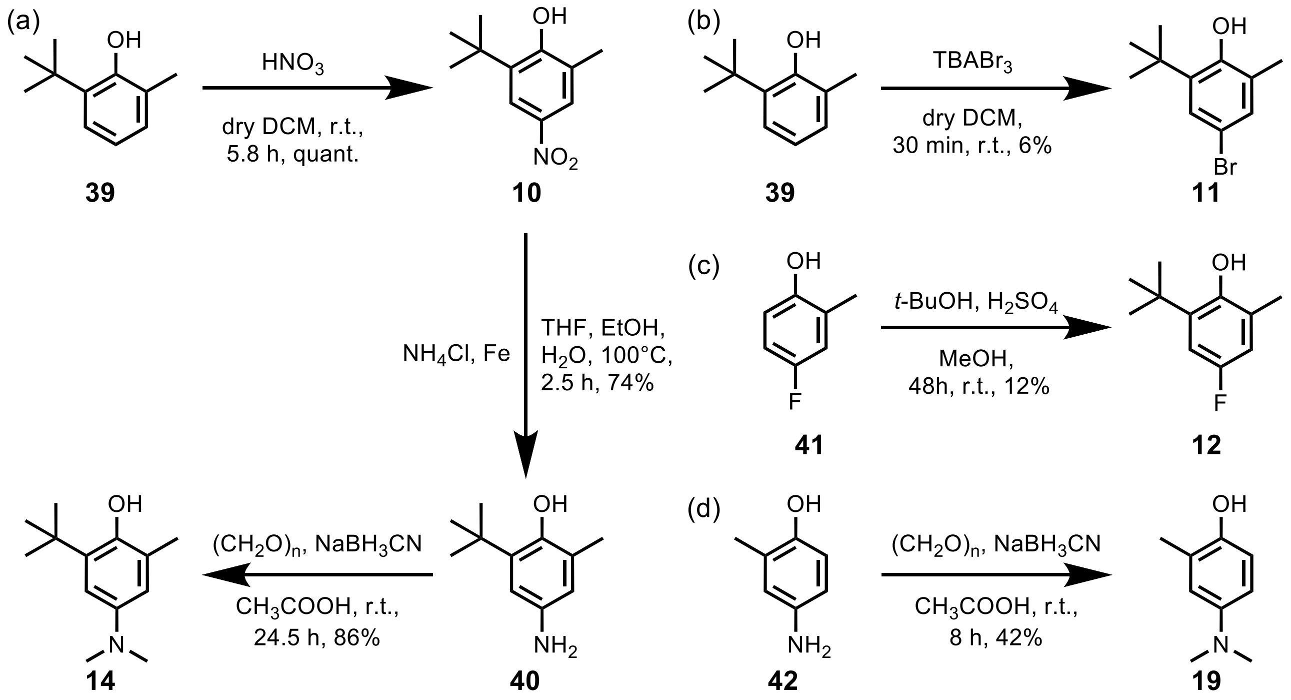

Scheme S1 Synthesis of the reference compounds (a) **10** and **14**, (b) **11**, (c) **12**, (d) **19**.

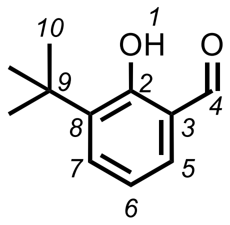

24

Modified from a previously reported procedure.[2]

To a solution of 2-*tert*-butylphenol (2.0 mL, 13 mmol) in dry acetonitrile (30 mL), magnesium chloride (1.9012 g, 20 mmol), triethylamine (7.0 mL, 50 mmol) and paraformaldehyde (2.7031 g, 90 mmol) were added. The mixture was stirred at reflux (T = 82°C) for 5 hours. The mixture was then cooled to room temperature and quenched with 5% aqueous hydrochloric acid (42 mL). The aqueous layer was extracted with DCM (3x75 mL), the combined organic phases were washed once with brine (1x100 mL). The combined organic phases were dried over magnesium sulphate and dried under reduced pressure. The crude product was purified by flash column chromatography (SiO_2, 0-100% gradient of DCM in petroleum ether). The desired product was obtained as a transparent oil (1.4946 g, 8.4 mmol, 64%).

^{1}H-NMR (700 MHz, $CDCl_3$) δ_H (ppm): 11.78 (d, J = 0.6 Hz, 1H, H(1)), 9.88 (s, 1H, H(4)), 7.53 (dd, J = 7.7 Hz, 1.7 Hz, 0.6 Hz, 1H, H(7)), 7.40 (dd, J = 7.6 Hz, 1.7 Hz, 1H, H(5)), 6.95 (t, J = 7.7 Hz, 1H, H(6)), 1.42 (s, 9H, H(10)).

^{13}C{^{1}H}-NMR (176 MHz, $CDCl_3$) δ_C (ppm): 197.3 (1C, C(4)), 161.4 (1C, C(2)), 138.4 (1C, C(8)), 134.2 (1C, C(7)), 132.1 (1C, C(5)), 120.8 (1C, C(3)), 119.3 (1C, C(6)), 35.0 (1C, C(9)), 29.3 (3C, C(10)).

HRMS: calc. for $C_{11}H_{13}O_2^-$ $[M-H]^-$ 177.0916, found 177.0917.

IR spectrum $\tilde{v}$ (cm^{-1}): 2958 ($v_{O\text{-}H\ alcohol}$), 2913 ($v_{C\text{-}H\ alkene}$), 2871 ($v_{C\text{-}H\ alkane}$), 2741 ($v_{C\text{-}H\ aldehyde}$), 1650 ($v_{C=O\ conjugated\ aldehyde}$), 1612 ($v_{C=C\ cyclic\ alkene}$), 1453 ($\delta_{C\text{-}H\ alkane,\ methyl\ group}$), 1386 ($\delta_{C\text{-}H\ aldehyde}$), 1312 ($\delta_{O\text{-}H\ phenol}$).

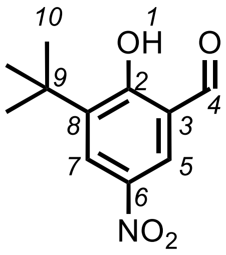

25

Modified from a previously reported procedure.[3]

3-(*tert*-Butyl)-2-hydroxybenzaldehyde (1.2299 g, 6.9 mmol) was dissolved in glacial acetic acid (19 mL) and nitric acid (7.5 mL, 168 mmol) was added dropwise at 0°C. The reaction mixture was stirred for 1 hour at room temperature. The resulting mixture was poured into water (150 mL) cooled to 0°C with vigorous stirring. A light yellow solid precipitated, it was filtered and washed with water (50 mL). The obtained light yellow solid was dried under vacuum (1.0846 g, 4.9 mmol, 70%).

^{1}H-NMR (400 MHz, $CDCl_3$) δ_H (ppm): 12.44 (s, 1H, H(1)), 9.97 (s, 1H, H(4)), 8.41 (s, 2H, H(5), H(7)), 1.46 (s, 9H, H(10)).

^{13}C{^{1}H}-NMR (101 MHz, $CDCl_3$) δ_C (ppm): 196.4 (1C, C(4)), 166.0 (1C, C(2)), 140.8 (1C, C(8)), 140.2 (1C, C(6)), 128.8 (1C, C(7)), 128.0 (1C, C(5)), 119.5 (1C, C(3)), 35.5 (1C, C(9)), 29.0 (3C, C(10)).

HRMS: calc. for $C_{11}H_{14}NO_4^+$ $[M+H]^+$ 224.0923, found 224.0916.

IR spectrum $\tilde{\nu}$ (cm^{-1}): 2963 ($\nu_{\text{O-H alcohol}}$), 2916 ($\nu_{\text{C-H alkene}}$), 2874 ($\nu_{\text{C-H alkane}}$), 1656 ($\nu_{\text{C=O conjugated aldehyde}}$), 1620 ($\nu_{\text{C=C cyclic alkene}}$), 1530 ($\nu_{\text{N-O}}$), 1435 ($\delta_{\text{C-H alkane, methyl group}}$), 1365 ($\delta_{\text{C-H aldehyde}}$), 1343 ($\delta_{\text{O-H phenol}}$).

m.p.: 78.5-81.0°C (82.5-83.5°C[4]).

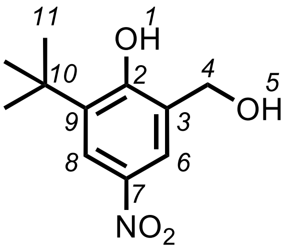

28

Modified from a previously reported procedure.[5]

To a solution of 3-(*tert*-butyl)-2-hydroxy-5-nitrobenzaldehyde (917.8 mg, 4.1 mmol) in methanol (40 mL) cooled to 0°C and stirred vigorously, sodium borohydride (565.4 mg, 15 mmol) was added. The reaction mixture was then warmed to room temperature and stirred for 24 hours. The solvent was removed under reduced pressure to yield a yellow, oily residue to which water (5 mL) and glacial acetic acid (2 mL) were added. More water (100 mL) was added and the aqueous phase was extracted with DCM (3x150 mL), dried over magnesium sulphate and the solvent was removed under reduced pressure. The crude product was purified by flash column chromatography (SiO_2, 0-100% gradient of ethyl acetate in petroleum ether). The desired product was obtained as an orange oil (425.0 g, 1.9 mmol, 46%).

^{1}H-NMR (400 MHz, $CDCl_3$) δ_H (ppm): 8.92 (s, 1H, H(1)), 8.15 (d, J = 2.7 Hz, 1H, H(8)), 7.82 (d, J = 2.7 Hz, 1H, H(6)), 4.98 (d, J = 5.4 Hz, 2H, H(4)), 2.51 (t, J = 5.5 Hz, 1H, H(5)), 1.44 (s, 9H, H(11)).

^{13}C{^{1}H}-NMR (101 MHz, $CDCl_3$) δ_C (ppm): 162.0 (1C, C(2)), 140.1 (1C, C(7)), 138.7 (1C, C(9)), 124.4 (1C, C(3)), 123.3 (1C, C(8)), 121.8 (1C, C(6)), 65.2 (1C, C(4)), 35.2 (1C, C(10)), 29.3 (3C, C(11)).

HRMS: calc. for $C_{11}H_{16}NO_4^+$ $[M+H]^+$ 226.1079, found 226.1071.

IR spectrum $\tilde{\nu}$ (cm^{-1}): 3437, 3270 ($\nu_{O\text{-}H\ alcohol}$), 2963 ($\nu_{C\text{-}H\ alkene}$), 2918, 2873 ($\nu_{C\text{-}H\ alkane}$), 1590 ($\nu_{C=C\ cyclic\ alkene}$), 1518 ($\nu_{N\text{-}O}$), 1455 ($\delta_{C\text{-}H\ alkane,\ methyl\ group}$), 1334 ($\delta_{O\text{-}H\ phenol}$).

m.p.: 70.8°C -72.2°C.

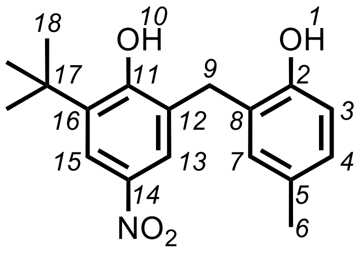

1

Modified from a previously reported procedure on a different substrate.[6]

p-Cresol (755.7 mg, 7.0 mmol), 2-(*tert*-butyl)-6-(hydroxymethyl)-4-nitrophenol (334.9 mg, 1.5 mmol) and *p*-toluenesulfonic acid monohydrate (57.8 mg, 0.30 mmol) were dissolved in dry toluene (10 mL) and heated to reflux (T = 120°C) under an inert atmosphere for 4 days. The solvent was then removed under reduced pressure and the crude product was dried under high vacuum. It was then purified by flash column chromatography (SiO_2, 0-10% gradient of ethyl acetate in petroleum ether). The desired product was obtained as a yellow solid (0.2074 g, 0.66 mmol, 44%).

^{1}H-NMR (700 MHz, $CDCl_3$) δ_H (ppm): 8.15 (s, 1H, H(10)), 8.11 (d, J = 2.8 Hz, 1H, H(13)), 8.09 (d, J = 2.8 Hz, 1H, H(15)), 7.14 (d, J = 1.6 Hz, 1H, H(7)), 6.94 (dd, J = 8.1 Hz, 1.6 Hz, 1H, H (4)), 6.71 (d, J = 8.1 Hz, 1H, H(3)), 5.37 (s, 1H, H(1)), 3.93 (s, 2H, H(9)), 2.27 (s, 3H, H(6)), 1.40 (s, 9H, H(18)).

^{13}C{^{1}H}-NMR (176 MHz, $CDCl_3$) δ_C (ppm): 159.3 (1C, C(11)), 148.7 (1C, C(2)), 140.6 (1C, C(14)), 138.2 (1C, C(16)), 132.3 (1C, C(5)), 131.7 (1C, C(7)), 129.0 (1C, C(4)), 128.0 (1C, C(12)), 125.1 (1C, C(8)), 124.6 (1C, C(13)), 122.1 (1C, C(15)), 115.2 (1C, C(3)), 35.4 (1C, C(17)), 30.9 (1C, C(9)), 29.5 (3C, C(18)), 20.7 (1C, C(6)).

HRMS: calc. for $C_{18}H_{20}NO_4^-$ [M-H]$^-$ 314.1392, found 314.1417.

IR spectrum $\tilde{\nu}$ (cm^{-1}): 3289 ($\nu_{\text{O-H alcohol}}$), 2961 ($\nu_{\text{C-H alkene}}$), 2930-2862 ($\nu_{\text{C-H alkane}}$), 1585 ($\nu_{\text{C=C cyclic alkene}}$), 1509 ($\nu_{\text{N-O}}$), 1472 ($\delta_{\text{C-H alkane, methyl group}}$), 1336 ($\delta_{\text{O-H phenol}}$).

m.p.: 199.8-201.2°C.

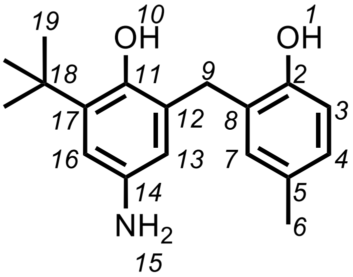

32

1 (91.6 mg, 0.29 mmol), ammonium chloride (48.5 mg, 0.91 mmol) and iron powder(192.8 mg, 3.5 mmol) were dissolved in THF/ethanol/water (2:2:1, 5.5 mL). The mixture was refluxed at 100°C for 2 hours. After cooling to room temperature, the mixture was filtered through Celite and evaporated to dryness. The residue was partitioned between water (50 mL) and ethyl acetate (50 mL). The organic layer was washed with brine (1x50 mL), dried with magnesium sulphate, filtered and dried under reduced pressure. The crude product was purified by flash column chromatography (SiO_2, 0-50% gradient of ethyl acetate in petroleum ether). The desired product was obtained as a red solid (65.9 mg, 0.23 mmol, 80%).

^{1}H-NMR (400 MHz, $CDCl_3$) δ_H (ppm): 7.05 (s, 1H, H(7)), 6.87 (d, J = 8.2 Hz, 1H, H(4)), 6.63 (d, J = 8.2 Hz, H(3)), 6.59-6.50 (m, 2H, H(13), H(16)), 3.80 (s, 2H, H(9)), 2.24 (s, 3H, H(6)), 1.37 (s, 9H, H(19)).

^{13}C{^{1}H}-NMR (101 MHz, $CDCl_3$) δ_C (ppm): 150.4 (1C, C(2)), 145.9 (1C, C(11)), 138.6 (1C, C(17)), 131.4 (1C, C(7)), 130.8 (1C, C(5)), 128.9 (1C, C(12)), 128.4 (1C, C(4)), 126.7 (1C, C(8)), 115.6 (1C, C(3)), 114.1 (1C, C(14)), 34.8 (1C; C(18)), 31.5 (1C, C(9)), 30.0 (3C, C(19)), 20.7 (1C, C(6)).

HRMS: calc. for $C_{18}H_{24}NO_2^+$ $[M+H]^+$ 286.1807, found 286.1773.

IR spectrum ṽ (cm^{-1}): 3358 ($\nu_{N\text{-}H}$), 3295 ($\nu_{O\text{-}H\ alcohol}$), 3006 ($\nu_{C\text{-}H\ alkene}$), 2952-2863 ($\nu_{C\text{-}H\ alkane}$), 1605 ($\nu_{C=C\ cyclic\ alkene}$), 1441 ($\delta_{C\text{-}H\ alkane,\ methyl\ group}$), 1359 ($\delta_{O\text{-}H\ phenol}$).

m.p.: 153.3-155.2°C.

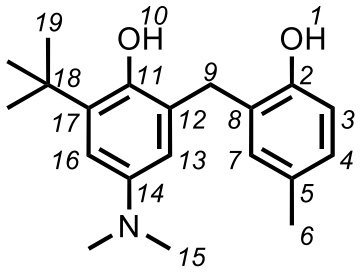

5

32 (21.3 mg, 0.075 mmol), paraformaldehyde (40.1 mg, 1.3 mmol) and sodium cyanoborohydride (28.7 mg, 0.50 mmol) were dissolved in glacial acetic acid (1.28 mL) and water (0.1 mL). The mixture was stirred for 6.5 hours. Then saturated aqueous potassium carbonate was poured into the reaction mixture to adjust the pH to 7-8. It was then extracted with DCM (2x50 mL). The combined organic layers were dried over magnesium sulphate, filtered and dried under reduced pressure. The crude product was purified by flash column chromatography (SiO_2, 0-40% gradient of ethyl acetate in petroleum ether). The desired product was obtained as a red solid (11.8 mg, 0.038 mmol, 50%).

^{1}H-NMR (400 MHz, d_6-DMSO) δ_H (ppm): 9.71 (s, 1H, H(1)), 7.63 (s, 1H, H(10)), 6.90 (d, J = 2.2 Hz, 1H, H(7)), 6.81 (dd, J = 8.1, 1.8 Hz, 1H, H(4)), 6.70 (d, J = 8.1 Hz, 1H, H(3)), 6.48 (d, J = 3.0 Hz, 1H, H(16)), 6.39 (d, J = 3.0 Hz, 1H, H(13)), 3.74 (s, 2H, H(9)), 2.70 (s, 6H, H(15)), 2.14 (s, 3H, H(6)), 1.35 (s, 9H, H(19)).

^{13}C{^{1}H}-NMR (176 MHz, d_6-DMSO) δ_C (ppm): 151.6 (1C, C(2)), 144.8 (1C, C(11)), 144.2 (1C, C(14)), 137.1 (1C, C(17)), 130.9 (1C, C(7)), 129.1 (1C, C(12)), 127.8 (1C, C(5)), 127.4 (1C, C(4)), 126.8 (1C, C(8)), 114.6 (1C, C(3)), 113.4 (1C, C(13)), 110.6 (1C, C(16)), 41.4 (2C, C(15)), 34.7 (1C, C(18)), 30.4 (1C, C(9)), 29.8 (3C, C(19)), 20.2 (1C, C(6)).

HRMS: calc. for $C_{20}H_{28}NO_2^+$ $[M+H]^+$ 314.2120, found 314.2103.

IR spectrum $\tilde{\nu}$ (cm^{-1}): 3296 ($\nu_{O\text{-}H\ alcohol}$), 3001 ($\nu_{C\text{-}H\ alkene}$), 2950-2790 ($\nu_{C\text{-}H\ alkane}$), 1603 ($\nu_{C=C\ cyclic\ alkene}$), 1478 ($\delta_{C\text{-}H\ alkane,\ methyl\ group}$), 1357 ($\delta_{O\text{-}H\ phenol}$).

m.p.: 119.6-121.4°C.

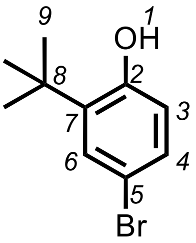

22

Modified from a previously reported procedure.[7]

A solution of tetrabutylammonium tribromide (5.2128 g, 11 mmol) and 2-(*tert*-butyl)-phenol (1.5 mL, 9.8 mmol) in dry DCM (42 mL) was stirred at room temperature for 1 hour. Diethyl ether (200 mL) and water (100 mL) were added to the reaction mixture. The organic phase was washed twice with a 1 M solution of hydrochloric acid (2x150 mL) and brine (1x150 mL). The organic phase was then dried over magnesium sulphate, filtered and dried under reduced pressure. A dark orange oil was obtained as the product (2.2035 g, 9.6 mmol, yield 98%).

^{1}H-NMR (400 MHz, $CDCl_3$) δ_H (ppm): 7.35 (d, J = 2.4 Hz, 1H, H(6)), 7.17 (dd, J = 8.4 Hz, 2.4 Hz, 1H, H(4)), 6.55 (d, J = 8.4 Hz, 1H, H(3)), 4.74 (s, 1H, H(1)), 1.39 (s, 1H, H(9)).

^{13}C{^{1}H}-NMR (101 MHz, $CDCl_3$) δ_C (ppm): 153.4 (1C, C(2)), 138.7 (1C, C(7)), 130.3 (1C, C(6)), 129.7 (1C, C(4)), 118.3 (1C, C(3)), 113.1 (1C, C(5)), 34.9 (1C, C(8)), 29.5 (3C, C(9)).

LC-MS: calc. for $C_{10}H_{12}BrO^-$ $[M-H]^-$ 229.0, found 228.8.

IR spectrum $\tilde{\nu}$ (cm^{-1}): 3548 ($\nu_{O\text{-}H\ alcohol}$), 2999 ($\nu_{C\text{-}H\ alkene}$), 2957-2869 ($\nu_{C\text{-}H\ alkane}$), 1459 ($\delta_{C\text{-}H\ alkane,\ methyl\ group}$), 1363 ($\delta_{O\text{-}H\ phenol}$), 631 ($\nu_{C\text{-}Br}$).

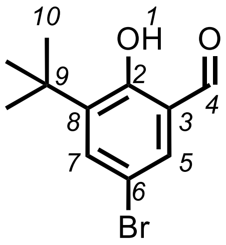

26

Modified from a previously reported procedure.[7]

Under an inert atmosphere, a solution of 4-bromo-2-(*tert*-butyl)phenol (1.7575 g, 7.7 mmol), magnesium chloride (1.5007 g, 16 mol) and triethylamine (4.1 mL, 29 mmol) in dry THF (40 mL) was stirred at room temperature for 30 minutes. Then paraformaldehyde (1.7515 g, 58 mmol) was added and the obtained reaction mixture was stirred for 17.5 hours at 75°C. After cooling down to room temperature, the mixture was diluted with ethyl acetate (50 mL) and washed with a 1 M aqueous solution of hydrochloric acid (5x50 mL) and brine (2x50 mL). The organic phase was dried over magnesium sulphate, filtered and dried under reduced pressure. The crude product was purified by flash column chromatography (SiO_2, petroleum ether). The product was obtained as a light yellow solid (1.5957 g, 6.2 mmol, 81%).

^{1}H-NMR (400 MHz, $CDCl_3$) δ_H (ppm): 11.73 (s, 1H, H(1)), 9.81 (s, 1H, H(4)), 7.58 (dd, J = 2.5 Hz, 1H, H(7)), 7.52 (dd, J = 2.5 Hz, H(5)), 1.41 (s, 9H, H(10)).

^{13}C{^{1}H}-NMR (101 MHz, $CDCl_3$) δ_C (ppm): 196.2 (1C, C(4)), 160.4 (1C, C(2)), 141.3 (1C, C(8)), 137.2 (1C, C(7)), 133.8 (1C, C(5)), 121.8 (1C, C(3)), 111.3 (1C, C(6)), 35.3 (1C, C(9)), 29.2 (3C, C(10)).

LC-MS: calc. for $C_{11}H_{12}BrO^-$ $[M+H]^+$ 257.0, found 256.9.

IR spectrum $\tilde{\nu}$ (cm^{-1}): 3058 ($\nu_{O\text{-}H\ alcohol}$), 3000 ($\nu_{C\text{-}H\ alkene}$), 2961-2870 ($\nu_{C\text{-}H\ alkane}$), 1659 ($\nu_{C=O\ aldehyde}$), 1604 ($\nu_{C=C\ cyclic\ alkene}$), 1465 ($\delta_{C\text{-}H\ alkane,\ methyl\ group}$), 1364 ($\delta_{O\text{-}H\ phenol}$), 667 ($\nu_{C\text{-}Br}$).

m.p.: 68.3-68.8°C (lit. 62-64°C[8]).

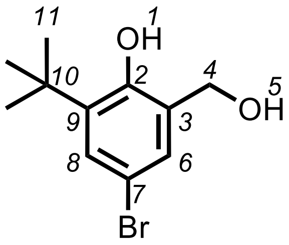

29

Modified from a previously reported procedure.[2]

To a vigorously stirred solution of 5-bromo-3-(*tert*-butyl)-2-hydroxybenzaldehyde (1.0227 g, 4.0 mmol) in methanol (40 mL) cooled to 0°C, sodium borohydride (544.7 mg, 14 mmol) was added. The reaction mixture was then warmed to room temperature and stirred for 21 hours. The solvent was removed under reduced pressure, water (50 mL) and glacial acetic acid (5 mL) were added to the residue. The aqueous phase was extracted with DCM (3x50 mL) and the combined organic phases were dried over magnesium sulphate, filtered and dried under reduced pressure. The crude product was purified by flash column chromatography (SiO_2, 0-20% gradient of ethyl acetate in petroleum ether). The product was obtained as a light yellow oil (1.0053 g, 3.9 mmol, 98%).

^{1}H-NMR (700 MHz, $CDCl_3$) δ_H (ppm): 7.80 (s, 1H, H(1)), 7.32 (d, J = 2.4 Hz, 1H, H(8)), 7.01 (d, J = 2.5 Hz, 1H, H(6)), 4.83 (d, J = 4.5 Hz, 2H, H(4)), 2.10 (t, J = 5.2 Hz, 1H, H(5)), 1.39 (s, 9H, H(11)).

^{13}C{^{1}H}-NMR (176 MHz, $CDCl_3$) δ_C (ppm): 154.9 (1C, C(2)), 139.9 (1C, C(9)), 130.1 (1C, C(8)), 128.3 (1C, C(6)), 126.5 (1C, C(3)), 111.6 (1C, C(7)), 65.1 (1C, C(4)), 35.1 (1C, C(10)), 29.5 (1C, C(11)).

HRMS: calc. for $C_{11}H_{14}BrO_2^-$ $[M-H]^-$ 257.0183, found 257.0168.

IR spectrum $\tilde{\nu}$ (cm^{-1}): 3393 ($\nu_{O\text{-}H\ alcohol}$), 2999 ($\nu_{C\text{-}H\ alkene}$), 2954-2870 ($\nu_{C\text{-}H\ alkane}$), 1469 ($\delta_{C\text{-}H\ alkane,\ methyl\ group}$), 1361 ($\delta_{O\text{-}H\ phenol}$), 656 ($\nu_{C\text{-}Br}$).

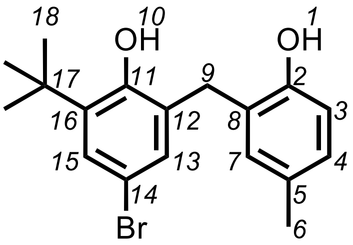

2

Modified from a previously reported procedure on a different substrate.[9]

p-Cresol (1.4147 g, 13 mmol), 4-bromo-2-(*tert*-butyl)-6-(hydroxymethyl)phenol (663.6 mg, 2.6 mmol) and *p*-toluensulfonic acid monohydrate (109.1 mg, 0.57 mmol) were dissolved in dry toluene (20 mL) and heated to reflux (T = 120°C) under an inert atmosphere for 24 hours. The solvent was removed under reduced pressure and purified by flash column chromatography (C18, 10-100% gradient of acetonitrile in water). The product was obtained as a off-white solid (685.9 mg, 2.0 mmol, 77%).

^{1}H-NMR (400 MHz, $CDCl_3$) δ_H (ppm): 7.26 (d, J = 2.4 Hz, 1H, H(13)), 7.23 (d, J = 2.5 Hz, 1H, H(15)), 7.07 (d, J = 2.1 Hz, 1H, H(7)), 6.98-6.88 (m, 2H, H(10), H(4)), 6.66 (d, J = 8.1 Hz, 1H, H(4)), 5.22 (s, 1H, H(1)), 3.83 (s, 2H, H(9)), 2.27 (s, 3H, H(6)), 1.37 (s, 9H, H(18)).

^{13}C{^{1}H}-NMR (101 MHz, $CDCl_3$) δ_C (ppm): 152.0 (1C, C(11)), 149.3 (1C, C(2)), 139.6 (1C, C(16)), 131.7 (1C, C(5)), 131.6 (1C, C(7)), 130.9 (1C, C(13)), 129.6 (1C, C(12)), 128.7 (1C, C(4)), 128.6 (1C, C(15)), 125.9 (1C, C(8)), 115.3 (1C, C(3)), 112.5 (1C, C(14)), 35.1 (1C, C(17)), 30.8 (1C, C(9)), 29.7 (3C, C(18)), 20.7 (1C, C(6)).

HRMS: calc. for $C_{18}H_{21}BrO_2^+$ $[M]^+$ 348.0725, found 348.0720.

IR spectrum $\tilde{\nu}$ (cm^{-1}): 3355 ($\nu_{O\text{-}H\ alcohol}$), 3011-3000 ($\nu_{C\text{-}H\ alkene}$), 2957-2870 ($\nu_{C\text{-}H\ alkane}$), 1468 ($\delta_{C\text{-}H\ alkane,\ methyl\ group}$), 1361 ($\delta_{O\text{-}H\ phenol}$), 649 ($\nu_{C\text{-}Br}$).

m.p.: 107.6-108.7°C.

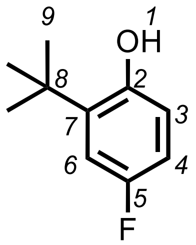

23

Modified from a previously reported procedure.[10]

To a solution of 4-fluorophenol (5.0579 g, 41 mmol) and *tert*-butanol (8.5 mL, 89 mmol), concentrated sulphuric acid (4.0 mL, 75 mmol) was added dropwise. The mixture was stirred at room temperature for 18 hours. It was then diluted with diethyl ether (70 mL). The acid layer was removed, and the organic phase was neutralised with saturated aqueous hydrogen carbonate (100 mL), then washed with brine (100 mL). The organic phase was dried over magnesium sulphate, filtered and dried under reduced pressure. The crude product was purified by flash column chromatography (SiO_2, 0-10% gradient of ethyl acetate in petroleum ether). The product was obtained as a light yellow oil (5.6742 g, 34 mmol, 81%).

^{1}H-NMR (400 MHz, $CDCl_3$) δ_H (ppm): 6.98 (dd, J = 10.9, 3.0 Hz, 1H, H(6)), 6.75 (ddd, J = 8.6, 7.5, 3.1 Hz, 1H, H(4)), 6.59 (dd, 1H, J = 8.7, 4.9 Hz, H(3)), 4.61 (s, 1H, H(1)), 1.39 (s, 9H, H(9)).

^{13}C{^{1}H}-NMR (101 MHz, $CDCl_3$) δ_C (ppm): 157.2 (d, J = 236.7 Hz, 1C, C(5)), 150.2 (d, J = 2.1 Hz, 1C, C(2)), 138.2 (d, J = 6.0 Hz, 1C, C(7)), 117.1 (d, J = 8.3 Hz, 1C, C(3)), 114.2 (d, J = 24.0 Hz, 1C, C(6)), 112.8 (d, J = 23.0 Hz, 1C, C(4)), 34.8 (s, 1C, C(8)), 29.5 (s, 3C, C(9)).

^{19}F-NMR (471 MHz, $CDCl_3$) δ_F (ppm): -122.74.

LC-MS: calc. for $C_{10}H_{12}FO^-$ $[M-H]^-$ 167.1, found 166.9.

IR spectrum $\tilde{\nu}$ (cm^{-1}): 3595 ($\nu_{O\text{-}H\ alcohol}$), 2998 ($\nu_{C\text{-}H\ alkene}$), 2959-2871 ($\nu_{C\text{-}H\ alkane}$), 1420 ($\delta_{C\text{-}H\ alkane,\ methyl\ group}$), 1364 ($\delta_{O\text{-}H\ phenol}$).

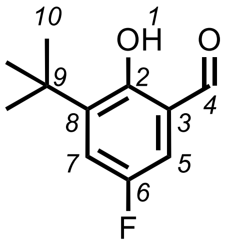

27

To a solution of **23** (5.0699 g, 30 mmol) in dry acetonitrile (80 mL), magnesium chloride (4.5162 g, 47 mmol), triethylamine (16 mL, 115 mmol) and paraformaldehyde (6.9471 g, 231 mmol) were added. The reaction mixture was stirred at 82°C for 4.5 hours. The reaction was then cooled to room temperature and quenched with an aqueous solution of hydrochloric acid 1 M (20 mL). The aqueous layer was extracted with ethyl acetate (5x100 mL). The combined organic phases were dried over magnesium sulphate, filtered and dried under reduced pressure. The crude product was purified by flash column chromatography (SiO_2, 0-20% gradient of ethyl acetate in petroleum ether). The product was obtained as a light yellow solid (3.9498 g, 20 mmol, 67%).

^{1}H-NMR (700 MHz, $CDCl_3$) δ_H (ppm): 11.58 (s, 1H, H(1)), 9.82 (s, 1H, H(4)), 7.28 (dd, J = 10.4 Hz, 3.1 Hz, 1H, H(7)), 7.07 (dd, J = 7.0, 3.1 Hz, 1H, H(5)), 1.41 (s, 9H, H(10)).

^{13}C{^{1}H}-NMR (176 MHz, $CDCl_3$) δ_C (ppm): 196.2 (d, J = 2.7 Hz, 1C, C(4)), 157.7 (d, J = 1.4 Hz, 1C, C(2)), 155.4 (d, J = 238.6 Hz, 1C, C(6)), 141.2 (d, J = 5.4 Hz, 1C, C(8)), 122.5 (d, J = 24.5 Hz, 1C, C(7)), 120.0 (d, J = 6.5 Hz, 1C, C(3)), 115.6 (d, J = 22.4 Hz, 1C, C(5)), 35.3 (1C, C(9)), 29.1 (3C, C(10)).

^{19}F-NMR (471 MHz, $CDCl_3$) δ_F (ppm): -122.78.

LC-MS: calc. for $C_{11}H_{12}FO_2^-$ $[M-H]^-$ 195.1, found 195.0.

IR spectrum $\tilde{\nu}$ (cm^{-1}): 3604 ($\nu_{O\text{-}H\ alcohol}$), 2958 ($\nu_{C\text{-}H\ alkene}$), 2913-2873 ($\nu_{C\text{-}H\ alkane}$), 1482 ($\delta_{C\text{-}H\ alkane,\ methyl\ group}$), 1362 ($\delta_{O\text{-}H\ phenol}$).

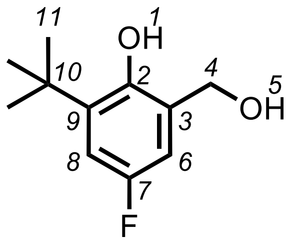

30

To a solution of **27** (1.1662 g, 5.9 mmol) in methanol (50 mL) cooled at 0°C, sodium borohydride (812.6 mg, 21 mmol) was added. The reaction mixture was stirred at room temperature for 3.5 hours. The solvent was removed under reduced pressure and the residue was dissolved in ethyl acetate (50 mL). It was washed with an aqueous solution of hydrochloric acid 3 M (50 mL) and brine (50 mL). The organic phase was dried over magnesium sulphate, filtered and dried under reduced pressure. The crude product was purified by flash column chromatography (SiO_2, 0-15% gradient of ethyl acetate in petroleum ether). The product was obtained as a transparent oil (0.7103 g, 3.6 mmol, 60%).

^{1}H-NMR (400 MHz, $CDCl_3$) δ_H (ppm): 7.55 (s, 1H, H(1)), 6.96 (dd, J = 10.9, 3.1 Hz, 1H, H(8)), 6.61 (dd, J = 7.8, 3.1 Hz, 1H, H(6)), 4.82 (d, J = 5.1 Hz, 2H, H(4)), 2.11 (t, J = 5.6 Hz, 1H, H(5)), 1.40 (s, 9H, H(11)).

^{13}C{^{1}H}-NMR (101 MHz, $CDCl_3$) δ_C (ppm): 156.0 (d, J = 236.4 Hz, 1C, C(7)), 151.5 (d, J = 2.3 Hz, 1C, C(2)), 139.4 (d, J = 6.0 Hz, 1C, C(9)), 125.4 (d, J = 7.0 Hz, 1C, C(3)), 113.9 (d, J = 23.4 Hz, 1C, C(8)), 111.8 (d, J = 23.1 Hz, 1C, C(6)), 65.3 (d, J = 1.9 Hz, 1C, C(4)), 35.1 (d, J = 1.1 Hz, 1C, C(10)), 29.5 (s, 3C, C(11)).

^{19}F-NMR (471 MHz, $CDCl_3$) δ_F (ppm): -123.83 ppm.

HRMS: calc. for $C_{11}H_{14}FO_2^-$ [M-H]$^-$ 197.0978, found 197.0981.

IR spectrum $\tilde{\nu}$ (cm^{-1}): 3328 ($\nu_{O\text{-}H\ alcohol}$), 3033 ($\nu_{C\text{-}H\ alkene}$), 2966-2874 ($\nu_{C\text{-}H\ alkane}$), 1469 ($\delta_{C\text{-}H\ alkane,\ methyl\ group}$), 1361 ($\delta_{O\text{-}H\ phenol}$).

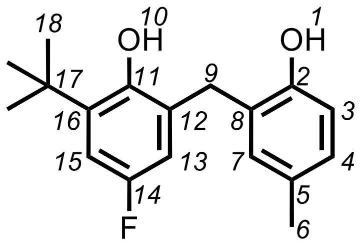

3

p-Cresol (1.6470 g, 15 mmol), 2-(*tert*-butyl)-4-fluoro-6-(hydroxymethyl)phenol (572.4 mg, 2.9 mmol) and *p*-toluensulfonic acid monohydrate (110.3 mg, 0.6 mmol) were dissolved in dry toluene (20 mL) and heated to reflux (T = 120°C) under an inert atmosphere for 2 hours. The solvent was removed under reduced pressure and the crude product purified by flash column chromatography (SiO_2, 0-100% gradient of ethyl acetate in petroleum ether, then C18, 10-100% gradient of acetonitrile in water). The product was obtained as a transparent oil (459.9 g, 1.6 mmol, 55%).

^{1}H-NMR (400 MHz, $CDCl_3$) δ_H (ppm): 7.07 (d, J = 2.1 Hz, 1H, H(7)), 6.91 (dd, J = 8.2, 2.1 Hz, 1H, H(4)), 6.85 (m, 2H, H(13), H(15)), 6.66 (d, J = 8.1 Hz, 1H, H(3)), 6.62 (s, 1H, H(10)), 5.39 (s, 1H, H(1)), 3.85 (s, 2H, H(9)), 2.26 (s, 3H, H(6)), 1.38 (s, 9H, H(18)).

^{13}C{^{1}H}-NMR (101 MHz, $CDCl_3$) δ_C (ppm): 156.7 (d, J = 236.5 Hz, 1C, C(14)), 149.7 (s, 1C, C(2)), 148.5 (d, J = 2.3 Hz, 1C, C(11)), 139.1 (d, J = 6.3 Hz, 1C, C(16)), 131.5 (s, 1C, C(7)), 131.5 (s, 1C, C(5)), 128.7-128.6 (m, 2C, C(4), C(12)), 126.1 (s, 1C, C(8)), 115.4 (s, 1C, C(3)), 114.1 (d, J = 22.4 Hz, H(13)), 112.3 (d, J = 23.6 Hz, C(15)), 35.0 (d, J = 1.2 Hz, C(17)), 31.2 (d, J = 1.5 Hz, C(9)), 29.7 (s, 3C, C(18)), 20.7 (s, 1C, C(6)).

^{19}F-NMR (471 MHz, $CDCl_3$) δ_F (ppm): -123.26 ppm.

HRMS: calc. for $C_{18}H_{20}FO_2^-$ [M-H]$^-$ 287.1447, found 287.1453.

IR spectrum $\tilde{\nu}$ (cm^{-1}): 3327 ($\nu_{\text{O-H alcohol}}$), 3006 ($\nu_{\text{C-H alkene}}$), 2958-2872 ($\nu_{\text{C-H alkane}}$), 1475 ($\delta_{\text{C-H alkane, methyl group}}$), 1361 ($\delta_{\text{O-H phenol}}$).

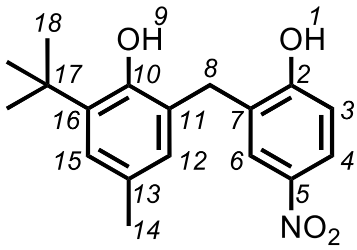

6

2-Hydroxy-5-nitrobenzyl alcohol (1.5807 g, 9.3 mmol), 2-*tert*-butyl-4-methylphenol (7.5402 g, 46 mmol) and *p*-toluensulfonic acid monohydrate (411.2 mg, 2.2 mmol) were dissolved in dry toluene (60 mL) and heated to reflux (T = 120°C) under an inert atmosphere for 72 hours. The solvent was removed under reduced pressure and the crude product purified by flash column chromatography (SiO_2, 0-100% gradient of ethyl acetate in petroleum ether, then C18, 10-100% gradient of acetonitrile in water). The product was obtained as a yellow solid (162.8 mg, 0.52 mmol, 5%).

^{1}H-NMR (400 MHz, $CDCl_3$) δ_H (ppm): 8.21 (d, J = 2.7 Hz, 1H, H(6)), 8.03 (dd, J = 8.9, 2.8 Hz, 1H, H(4)), 7.77 (s, 1H, H(1)), 7.02-6.95 (m, 2H, H(12), H(15)), 6.88 (d, J = 8.9 Hz, 1H, H(3)), 5.70 (s, 1H, H(9)), 3.94 (s, 2H, H(8)), 2.26 (s, 3H, H(14)), 1.43 (s, 9H, H(18)).

^{13}C{^{1}H}-NMR (101 MHz, $CDCl_3$) δ_C (ppm): 159.8 (1C, C(2)), 148.3 (1C, C(10)), 141.6 (1C, C(5)), 135.8 (1C, C(16)), 131.2 (1C, C(13)), 129.4 (1C, C(12)), 127.9 (1C, C(7)), 126.8 (1C, C(15)), 126.8 (1C, C(6)), 126.2 (1C, C(11)), 124.5 (1C, C(4)), 116.7 (1C, C(3)), 34.1 (1C, C(17)), 31.0 (1C, C(8)), 30.5 (3C, C(18)), 21.0 (1C, C(14)).

HRMS: calc. for $C_{18}H_{20}NO_4^-$ [M-H]$^-$ 314.1392, found 314.1403.

IR spectrum $\tilde{\nu}$ (cm^{-1}): 3458-3243 ($\nu_{\text{O-H alcohol, intermolecular bonded}}$), 3012 ($\nu_{\text{C-H alkene}}$), 2964-2873 ($\nu_{\text{C-H alkane}}$), 1587 ($\nu_{\text{C=C cyclic alkene}}$), 1518 ($\nu_{\text{N-O}}$), 1487 ($\delta_{\text{C-H alkane, methyl group}}$), 1332 ($\delta_{\text{O-H phenol}}$).

m.p.: 194.8-196.9°C.

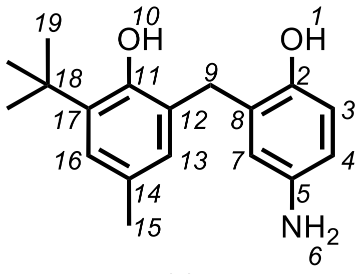

38

6 (116.5 mg, 0.37 mmol), ammonium chloride (62.0 mg, 1.2 mmol) and iron powder (237.3 mg, 4.2 mmol) were dissolved in THF/EtOH/H_2O (2:2:1, 7.0 mL). The reaction mixture was refluxed at 100°C for 1 hour. After cooling to room temperature, it was filtered through Celite and evaporated to dryness under reduced pressure. The residue was partitioned between water (50 mL) and ethyl acetate (50 mL). The organic layer was washed with brine (1x50 mL), dried with magnesium sulphate and dried under vacuum. An ochre solid was isolated (118.8 mg, 0.32 mmol, 86%).

^{1}H-NMR (400 MHz, $CDCl_3$) δ_H (ppm): 6.99-6.89 (m, 2H, H(13), H(16)), 6.64 (d, J = 2.8 Hz, 1H, H(7)), 6.58 (d, J = 8.4 Hz, 1H, H(3)), 6.44 (dd, J = 8.4, 2.8 Hz, 1H, H(4)), 3.81 (s, 2H, H(9)), 2.25 (s, 3H, H(15)), 1.38 (s, 9H, H(19)).

^{13}C{^{1}H}-NMR (101 MHz, $CDCl_3$) δ_C (ppm): 150.4 (1C, C(11)), 145.1 (1C, C(2)), 140.7 (1C, C(5)), 136.8 (1C, C(17)), 129.2 (1C, C(14)), 129.0 (1C, C(13)), 127.9 (1C, C(8)), 127.2 (1C, C(12)), 126.3 (1C, C(16)), 118.0 (1C, C(7)), 116.5 (1C, C(3)), 115.0 (1C, C(4)), 34.7 (1C, C(18)), 31.3 (1C, C(9)), 30.0 (3C, C(19)), 21.0 (1C, C(15)).

HRMS: calc. for $C_{18}H_{22}NO_2^-$ [M-H]$^-$ 284.1651, found 284.1656.

IR spectrum $\tilde{v}$ (cm^{-1}): 3295 ($v_{O\text{-}H\ alcohol,}$ $v_{N\text{-}H}$), 2999 ($v_{C\text{-}H\ alkene}$), 2949-2855 ($v_{C\text{-}H\ alkane}$), 1611 ($\delta_{N\text{-}H}$), 1509 ($v_{C=C\ cyclic\ alkene}$), 1454 ($\delta_{C\text{-}H\ alkane,\ methyl\ group}$), 1363 ($\delta_{O\text{-}H\ phenol}$).

m.p.: 132.4-133.7°C

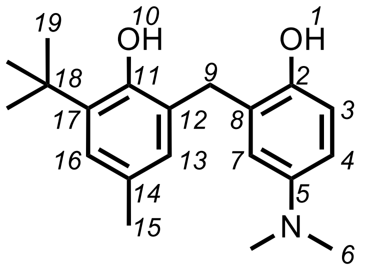

9

38 (97.0 mg, 0.34 mmol), paraformaldehyde (181.0 mg, 6.0 mmol) and sodium cyanoborohydride (119.0 mg, 1.9 mmol) were dissolved in glacial acetic acid (5.8 mL) and water (0.49 mL). The mixture was stirred for 4 hours at room temperature. Saturated aqueous potassium carbonate was poured into the reaction mixture to adjust the pH to 7. It was then extracted with DCM (4x50 mL). The combined organic phases were dried over magnesium sulphate, filtered and dried under vacuum. The crude product purified by flash column chromatography (SiO_2, 0-100% gradient of ethyl acetate in petroleum ether). The product was obtained as a red solid (46.3 mg, 0.15 mmol, 43%).

^{1}H-NMR (700 MHz, d_6-DMSO) δ_H (ppm): 9.32 (s, 1H, H(1)), 8.15 (s, 1H, H(10)), 6.80 (d, J = 2.2 Hz, 1H, H(16)), 6.72 (d, J = 2.2 Hz, 1H, H(13)), 6.69 (d, J = 8.7 Hz, 1H, H(3)), 6.59 (d, J = 3.1 Hz, 1H, H(7)), 6.48 (dd, J = 8.7 Hz, 3.1 Hz, 1H, H(4)), 3.74 (s, 2H, H(9)), 2.71 (s, 6H, H(6)), 2.12 (s, 3H, H(15)), 1.34 (s, 9H, H(19)).

^{13}C{^{1}H}-NMR (176 MHz, d_6-DMSO) δ_C (ppm): 150.6 (1C, C(11)), 145.6 (1C, C(2)), 144.8 (1C, C(5)), 136.3 (1C, C(17)), 128.4 (1C, C(12)), 128.1 (1C, C(13)), 127.3 (1C, C(14)), 127.0 (1C, C(8)), 124.8 (1C, C(16)), 116.1 (1C, C(7)), 115.3 (1C, C(3)), 112.4 (1C, C(4)), 41.3 (2C, C(6)), 34.4 (1C, C(18)), 30.6 (1C, C(9)), 29.7 (3C, C(19)), 20.6 (1C, C(15)).

HRMS: calc. for $C_{20}H_{26}NO_2^-$ [M-H]$^-$ 312.1964, found 312.1966.

IR spectrum $\tilde{\nu}$ (cm^{-1}): 3381 ($\nu_{O\text{-}H\ alcohol}$), 3011-2996 ($\nu_{C\text{-}H\ alkene}$), 2969-2872 ($\nu_{C\text{-}H\ alkane}$), 1616 ($\nu_{C=C\ cyclic\ alkene}$), 1477 ($\delta_{C\text{-}H\ alkane,\ methyl\ group}$), 1389 ($\delta_{O\text{-}H\ phenol}$).

m.p.: 59.8-61.7°C.

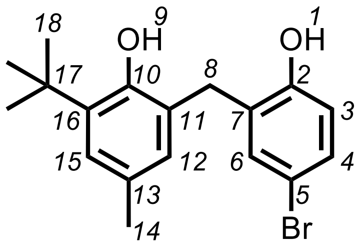

7

5-Bromo-2-hydroxybenzyl (1.5828 g, 7.8 mmol), 2-*tert*-butyl-4-methylphenol (6.4675 g, 39 mmol) and *p*-toluensulfonic acid monohydrate (297.0 mg, 1.6 mmol) were dissolved in dry toluene (50 mL) and heated to reflux (T = 120°C) under an inert atmosphere for 48 hours. The solvent was then removed under reduced pressure and the crude product was purified by flash column chromatography (SiO_2, 0-100% gradient of ethyl acetate in petroleum ether, then 0-10% gradient of ethyl acetate in petroleum ether). The product was obtained as a white solid (29.3 mg, 0.084 mmol, 1%).

^{1}H-NMR (400 MHz, $CDCl_3$) δ_H (ppm): 7.39 (s, 1H, H(6)), 7.19 (d, J = 8.4 Hz, 1H, H(4)), 6.97 (s, 1H, H(15)), 6.94 (s, 1H, H(12)), 6.66 (d, J = 8.4 Hz, H(3)), 6.21 (s, 1H, H(1/9)), 6.07 (s, 1H, H(1/9)), 3.85 (s, 2H, H(8)), 2.27 (s, 3H, H(14)), 1.41 (s, 9H, H(18)).

^{13}C{^{1}H}-NMR (101 MHz, $CDCl_3$) δ_C (ppm): 152.0 (1C, C(2)), 149.5 (1C, C(10)), 136.4 (1C, C(16)), 133.5 (1C, C(6)), 130.7 (1C, C(4)), 130.1 (1C, C(13)), 129.3 (1C, C(7)), 129.2 (1C, C(12)), 126.7 (1C, C(11)), 126.6 (1C, C(15)), 117.6 (1C, C(3)), 113.5 (1C, C(5)), 34.5 (1C, C(17)), 30.9 (1C, C(8)), 30.2 (3C, C(18)), 21.0 (1C, C(14)).

HRMS: calc. for $C_{18}H_{20}BrO_2^-$ $[M-H]^-$ 347.0652, found 347.0652.

IR spectrum $\tilde{v}$ (cm^{-1}): 3343 ($v_{O\text{-}H\ alcohol}$), 2997 ($v_{C\text{-}H\ alkene}$), 2952-2867 ($v_{C\text{-}H\ alkane}$), 1586 ($v_{C=C\ cyclic\ alkene}$), 1491 ($\delta_{C\text{-}H\ alkane,\ methyl\ group}$), 1359 ($\delta_{O\text{-}H\ phenol}$).

m.p.: 141.8-142.6°C.

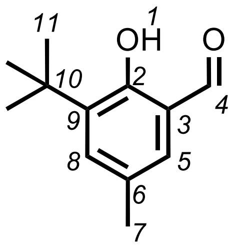

34

To a solution of 2-(*tert*-butyl)-4-methylphenol (5.1361 g, 31 mmol) in dry acetonitrile (720 mL), magnesium chloride (4.4968 g, 47 mmol), triethylamine (16.6 mL, 119 mmol) and paraformaldehyde (7.0845 g, 236 mmol) were added. The reaction mixture was stirred at reflux (T = 82°C) for 50 minutes under an inter atmosphere. The mixture was cooled to room temperature and quenched with a 5% aqueous solution of hydrochloric acid (42 mL). The aqueous layer was extracted with ethyl acetate (3x150 mL). The combined organic phases were concentrated under reduced pressure, washed with brine (1x150 mL), dried over magnesium sulphate, filtered and concentrated under reduced pressure. The crude product was purified by flash column chromatography (SiO_2, petroleum ether). The product was obtained as a light yellow solid (1.1667 g, 6.1 mmol, 19%).

^{1}H-NMR (400 MHz, $CDCl_3$) δ_H (ppm): 11.60 (s, 1H, H(1)), 9.83 (s, 1H, H(4)), 7.33 (d, J = 2.2 Hz, 1H, H(8)), 7.18 (d, J = 2.1 Hz, 1H, H(5)), 2.32 (s, 3H, H(7)), 1.41 (s, 9H, H(11)).

^{13}C{^{1}H}-NMR (101 MHz, $CDCl_3$) δ_C (ppm): 197.2 (1C, C(4)), 159.3 (1C, C(2)), 138.1 (1C, C(9)), 135.5 (1C, C(8)), 131.6 (1C, C(5)), 128.3 (1C, C(6)), 120.5 (1C, C(3)), 34.9 (1C, C(10)), 29.4 (3C, C(11)), 20.7 (1C, C(7)).

HRMS: calc. for $C_{12}H_{16}O_2^-$ [M-H]$^-$ 192.1150, found 191.1077.

IR spectrum $\tilde{\nu}$ (cm^{-1}): 3023 ($\nu_{O\text{-}H\ alcohol}$), 3001 ($\nu_{C\text{-}H\ alkene}$), 2954-2839 ($\nu_{C\text{-}H\ alkane}$), 1483 ($\delta_{C\text{-}H\ alkane,\ methyl\ group}$), 1356 ($\delta_{O\text{-}H\ phenol}$).

m.p.: 75.1-75.9°C (lit. 69-71°C[11]).

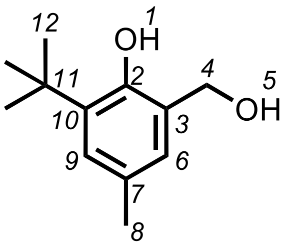

35

To a vigorously stirred solution of **34** (1.5916 g, 8.3 mmol) in methanol (45 mL) cooled to 0°C, sodium borohydride (1.1338 g, 30 mmol) was added. The reaction mixture was then warmed to room temperature and stirred for 16 hours. The solvent was removed under reduced pressure, water (50 mL) and glacial acetic acid (5 mL) were added to the residue. The aqueous phase was extracted with DCM (3x50 mL) and the combined organic phases were dried over magnesium sulphate, filtered and dried under reduced pressure. The crude product was purified by flash column chromatography (SiO_2, 0-20% gradient of ethyl acetate in petroleum ether). The product was obtained as a white solid (1.3187 g, 6.8 mmol, 82%).

^{1}H-NMR (400 MHz, $CDCl_3$) δ_H (ppm): 7.50 (s, 1H, H(1)), 7.05 (d, J = 2.2 Hz, 1H, H(9)), 6.71 (s, J = 2.1 Hz, 1H, H(6)), 4.82 (d, J = 5.7 Hz, 2H, H(4)), 2.26 (s, 3H, H(8)), 2.03 (t, J = 5.8 Hz, 1H, H(5)), 1.42 (s, 9H, H(12)).

^{13}C{^{1}H}-NMR (101 MHz, $CDCl_3$) δ_C (ppm): 153.3 (1C, C(2)), 137.2 (1C, C(10)), 128.3 (1C, C(7)), 127.8 (1C, C(9)), 126.4 (1C, C(6)), 124.7 (1C, C(3)), 65.7 (1C, C(4)), 34.8 (1C, C(11)), 29.8 (3C, C(12)), 20.9 (1C, C(8)).

HRMS: calc. for $C_{12}H_{17}O_2^-$ [M-H]- 193.1234, found 193.1235.

IR spectrum ṽ (cm^{-1}): 3350 ($\nu_{O\text{-}H\ alcohol}$), 2999 ($\nu_{C\text{-}H\ alkene}$), 2952-2868 ($\nu_{C\text{-}H\ alkane}$), 1479 ($\delta_{C\text{-}H\ alkane,\ methyl\ group}$), 1360 ($\delta_{O\text{-}H\ phenol}$).

m.p.: 53.3-54.9°C.

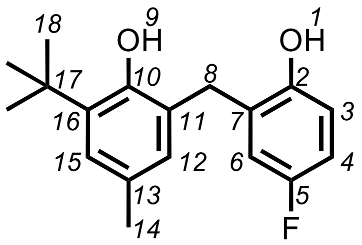

8

35 (0.6205 g, 3.2 mmol), 4-fluorophenol (1.2870 g, 11 mmol) and *p*-toluensulfonic acid monohydrate (81.3 mg, 0.43 mmol) were dissolved in dry toluene (10 mL) and heated to reflux (T = 120°C) under an inert atmosphere for 1 hour. The solvent was removed under reduced pressure and the crude product purified by flash column chromatography (SiO_2, 0-100% gradient of ethyl acetate in petroleum ether, then C18, 10-100% gradient of acetonitrile in water). The product was obtained as a white solid (258.4 mg, 0.90 mmol, 28%).

^{1}H-NMR (400 MHz, $CDCl_3$) δ_H (ppm): 7.02-6.91 (m, 3H, H(6), H(12), H(15)), 6.78 (m, 1H, H(4)), 6.71 (dd, J = 8.8 Hz, 4.7 Hz, 1H, H(3)), 6.19 (s, 1H, H(9)), 5.84 (s, 1H, H(1)), 3.86 (s, 2H, H(8)), 2.26 (s, 3H, H(14)), 1.40 (s, 9H, H(18)).

^{13}C{^{1}H}-NMR (101 MHz, $CDCl_3$) δ_C (ppm): 157.6 (d, J = 239.0 Hz, 1C, C(5)), 149.8 (s, 1C, C(10)), 148.5 (d, J = 2.3 Hz, 1C, C(2)), 136.6 (s, 1C, C(16)), 129.9 (s, 1C, C(13)), 129.1 (s, 1C, C(12)), 128.6 (d, J = 7.1 Hz, 1C, C(7)), 126.7 (s, 1C, C(11)), 126.6 (s, 1C, C(15)), 117.2 (d, J = 23.0 Hz, 1C, C(6)), 116.6 (d, J = 8.3 Hz, 1C, C(3)), 114.2 (d, J = 23.1 Hz, 1C, C(4)), 34.6 (s, 1C, C(17)), 31.1 (s, 1C, C(8)), 30.2 (s, 3C, C(18)), 21.0 (s, 1C, C(14)).

^{19}F-NMR (471 MHz, $CDCl_3$) δ_F (ppm): -121.94 ppm.

HRMS: calc. for $C_{18}H_{20}FO_2^-$ [M-H]$^-$ 287.1453, found 287.1453.

IR spectrum $\tilde{\nu}$ (cm^{-1}): 3333 ($\nu_{\text{O-H alcohol, intermolecular bonded}}$), 2999 ($\nu_{\text{C-H alkene}}$), 2955-2868 ($\nu_{\text{C-H alkane}}$), 1504 ($\nu_{\text{C=C cyclic alkene}}$), 1476 ($\delta_{\text{C-H alkane, methyl group}}$), 1361 ($\delta_{\text{O-H phenol}}$).

m.p.: 141.5-142.8°C.

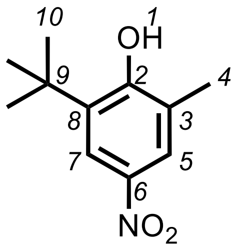

10

To a solution of 2-*tert*-butyl-6-methylphenol (0.6 mL, 3.5 mmol) in dry DCM (4.0 mL) cooled to 0°C, nitric acid (0.16 mL, 3.6 mmol) was added dropwise. The reaction mixture was stirred for 16 hours at room temperature. The resulting mixture was diluted with more DCM (50 mL) and it was washed with brine (3x50 mL). The organic phase was dried with magnesium sulphate, filtered and the solvent was removed under reduced pressure. The crude product was purified by flash column chromatography (SiO_2, 0-10% gradient of ethyl acetate in petroleum ether). The product was obtained as a yellow solid (166.4 mg, 0.80 mmol, 23%).

^{1}H-NMR (400 MHz, $CDCl_3$) δ_H (ppm): 8.10 (s, 1H, H(7)), 7.96 (s, 1H, H(5)), 5.48 (s, 1H, H(1)), 2.33 (s, 3H, H(4)), 1.44 (s, 9H, H(10)).

^{13}C{^{1}H}-NMR (101 MHz, $CDCl_3$) δ_C (ppm): 158.5 (1C, C(2)), 140.9 (1C, C(6)), 136.6 (1C, C(8)), 124.5 (1C, C(5)), 123.8 (1C, C(3)), 121.8 (1C, C(7)), 35.0 (1C, C(9)), 29.5 (3C, C(10)), 16.1 (1C, C(4)).

LC-MS: calc. for $C_{11}H_{14}NO_3^-$ [M-H]$^-$ 208.1, found 208.0.

IR spectrum $\tilde{\nu}$ (cm^{-1}): 3458 ($\nu_{\text{O-H alcohol, intermolecular bonded}}$), 2967 ($\nu_{\text{C-H alkene}}$), 2917-2873 ($\nu_{\text{C-H alkane}}$), 1588 ($\nu_{\text{C=C cyclic alkene}}$), 1515 ($\nu_{\text{N-O}}$), 1471 ($\delta_{\text{C-H alkane, methyl group}}$), 1327 ($\delta_{\text{O-H phenol}}$)

m.p.: 144.0 - 145.0°C (lit. 137 - 138°C[12]).

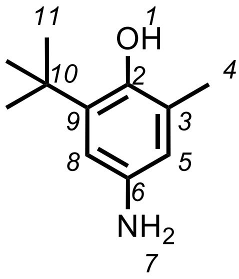

40

10 (53.5 mg, 0.26 mmol), ammonium chloride (43.0 mg, 0.80 mmol) and iron powder (164.3 mg, 2.9 mmol) were dissolved in THF/ethanol/water (2:2:1, 5 mL). The mixture was refluxed at 100°C for 2.5 hours. After cooling to room temperature, the mixture was filtered through Celite and evaporated to dryness. The residue was partitioned between water (50 mL) and ethyl acetate (50 mL). The organic layer was washed with brine (1x50 mL), dried with magnesium sulphate, filtered and dried under reduced pressure. The crude product was purified by flash column chromatography (SiO_2, 0-50% gradient of ethyl acetate in petroleum ether). The desired product was obtained as a red solid (34.0 mg, 0.19 mmol, 74%).

^{1}H-NMR (400 MHz, $CDCl_3$) δ_H (ppm): 6.54 (d, J = 2.7 Hz, 1H, H(8)), 6.40 (dd, J = 2.8, 0.8 Hz, 1H, H(5)), 4.30 (br s, 1H, H(1)), 3.33 (br s, 2H, H(7)), 2.17 (s, 3H, H(4)), 1.39 (s, 9H, H(11)).

^{13}C{^{1}H}-NMR (101 MHz, $CDCl_3$) δ_C (ppm): 145.7 (1C, C(2)), 139.0 (1C, C(6)), 137.1 (1C, C(9)), 124.5 (1C, C(3)), 115.7 (1C, C(5)), 112.8 (1C, C(8)), 34.6 (1C, C(10)), 29.9 (3C, C(11)), 16.4 (1C, C(4)).

HRMS: calc. for $C_{11}H_{18}NO^+$ $[M+H]^+$ 180.1388, found 180.1372.

IR spectrum $\tilde{\nu}$ (cm^{-1}): 3327 ($\nu_{N\text{-}H}$, $\nu_{O\text{-}H\ alcohol}$), 2999 ($\nu_{C\text{-}H\ alkene}$), 2954-2871 ($\nu_{C\text{-}H\ alkane}$), 1612 ($\nu_{C=C\ cyclic\ alkene}$), 1447 ($\delta_{C\text{-}H\ alkane,\ methyl\ group}$), 1360 ($\delta_{O\text{-}H\ phenol}$).

m.p.: 90.8-93.1°C.

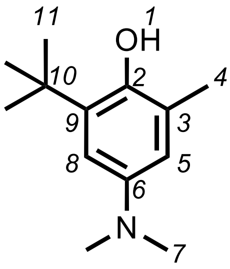

14

40 (15.1 mg, 0.084 mmol), paraformaldehyde (45.6 mg, 1.5 mmol) and sodium cyanoborohydride (29.7 mg, 0.47 mmol) were dissolved in glacial acetic acid (1.44 mL) and water (0.11 mL). The mixture was stirred for 24.5 hours. Then saturated aqueous potassium carbonate was poured into the reaction mixture to adjust the pH to 7-8. It was then extracted with DCM (2x50 mL). The combined organic layers were dried over magnesium sulphate, filtered and dried under reduced pressure. The crude product was purified by flash column chromatography (SiO_2, 0-100% gradient of ethyl acetate in petroleum ether). The desired product was obtained as a red solid (15.0 mg, 0.072 mmol, 86%).

^{1}H-NMR (400 MHz, d_6-DMSO) δ_H (ppm): 7.31 (s, 1H, H(1)), 6.46 (d, J = 3.0 Hz, 1H, H(8)), 6.39 (d, J = 3.0 Hz, 1H, H(5)), 2.74 (s, 6H, H(7)), 2.13 (s, 3H, H(4)), 1.34 (s, 9H, H(11)).

^{13}C{^{1}H}-NMR (101 MHz, d_6-DMSO) δ_C (ppm): 145.3 (1C, C(2)), 144.2 (1C, C(6)), 137.2 (1C, C(9)), 126.0 (1C, C(3)), 113.8 (1C, C(5)), 110.2 (1C, C(8)), 41.5 (2C, C(7)), 34.6 (1C, C(10)), 29.8 (3C, C(11)), 17.5 (1C, C(4)).

HRMS: calc. for $C_{13}H_{22}NO^+$ $[M+H]^+$ 208.1701, found 208.1701.

IR spectrum $\tilde{\nu}$ (cm^{-1}): 3250 ($\nu_{O\text{-}H\ alcohol}$), 2946 ($\nu_{C\text{-}H\ alkene}$), 2906-2809 ($\nu_{C\text{-}H\ alkane}$), 1606 ($\nu_{C=C\ cyclic\ alkene}$), 1432 ($\delta_{C\text{-}H\ alkane,\ methyl\ group}$), 1361 ($\delta_{O\text{-}H\ phenol}$).

m.p.: 117.0-119.3°C.

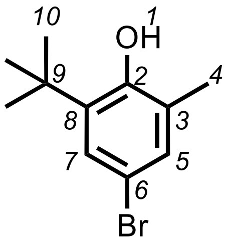

11

A solution of tetrabutylammonium tribromide (4.7882 g, 9.9 mmol) and 2-(*tert*-butyl)-6-methylphenol (1.5 mL, 8.8 mmol) in dry DCM (38 mL) was stirred at room temperature for 30 minutes. Diethyl ether (200 mL) and water (100 mL) were added to the reaction mixture. The organic phase was washed twice with a 1 M solution of hydrochloric acid (2x150 mL) and brine (1x150 mL). The organic phase was then dried over magnesium sulphate, filtered and dried under reduced pressure. The crude product was purified by flash column chromatography (SiO_2, 0-30% gradient of ethyl acetate in petroleum ether; C18, 5-100% gradient of acetonitrile in water). The product was obtained as an orange oil (0.1195 g, 0.49 mmol, 6%).

^{1}H-NMR (400 MHz, $CDCl_3$) δ_H (ppm): 7.23 (s, 1H, H(7)), 7.13 (s, 1H, H(5)), 4.72 (br s, 1H, H(1)), 2.22 (s, 3H, H(4)), 1.39 (s, 9H, H(10)).

^{13}C{^{1}H}-NMR (101 MHz, $CDCl_3$) δ_C (ppm): 151.9 (1C, C(2)), 138.1 (1C, C(8)), 131.0 (1C, C(5)), 128.1 (1C, C(7)), 125.3 (1C, C(3)), 112.5 (1C, C(6)), 34.9 (1C, C(9)), 29.7 (3C, C(10)), 16.0 (1C, C(4)).

HRMS: calc. for $C_{11}H_{14}BrO^-$ $[M\text{-}H]^-$ 241.0234, found 241.0217.

IR spectrum $\tilde{\nu}$ (cm^{-1}): 3578 ($\nu_{O\text{-}H\ alcohol}$), 2957 ($\nu_{C\text{-}H\ alkene}$), 2910-2871 ($\nu_{C\text{-}H\ alkane}$), 1473 ($\delta_{C\text{-}H\ alkane,\ methyl\ group}$), 1362 ($\delta_{O\text{-}H\ phenol}$), 670 ($\nu_{C\text{-}Br}$).

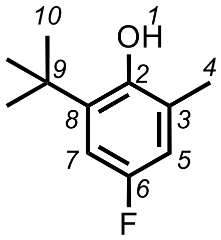

12

Concentrated sulphuric acid (1.6 mL) was added dropwise to a solution of 2-methyl-4-fluorophenol (1.0566 g, 8.4 mmol) in *tert*-butanol (3.4 mL, 36 mmol). The reaction mixture was stirred at room temperature for 48 hours. It was then diluted with diethyl ether (50 mL). The organic phase was neutralised with a saturated aqueous sodium bicarbonate (1x100 mL) and washed with brine (1x100 mL). The organic phase was dried with magnesium sulphate, filtered and dried under vacuum. The crude product purified by flash column chromatography (SiO_2, 0-20% gradient of ethyl acetate in petroleum ether). The product was obtained as a yellow solid (178.3 mg, 1.0 mmol, 12%).

^{1}H-NMR (400 MHz, $CDCl_3$) δ_H (ppm): 6.86 (dd, J = 10.9, 3.1 Hz, 1H, H(7)), 6.71 (dd, J = 8.2, 2.8 Hz, 1H, H(5)), 4.53 (s, 1H, H(1)), 2.23 (s, 3H, H(4)), 1.40 (s, 9H, H(10)).

^{13}C{^{1}H}-NMR (101 MHz, $CDCl_3$) δ_C (ppm): 156.5 (d, J = 236.1 Hz, 1C, C(6)), 148.6 (d, J = 2.3 Hz, 1C, C(2)), 137.6 (d, J = 6.4 Hz, 1C, C(8)), 124.5 (d, J = 8.1 Hz, 1C, C(3)), 114.4 (d, J = 22.7 Hz, 1C, C(5)), 111.8 (d, J = 23.7 Hz, 1C, C(7)), 34.8 (d, J = 1.2 Hz, 1C, C(9)), 29.7 (s, 3C, C(10)), 16.4 (d, J = 1.6 Hz, 1C, C(4)).

^{19}F-NMR (471 MHz, $CDCl_3$) δ_F (ppm): -123.36.

HRMS: calc. for $C_{11}H_{14}FO^-$ $[M-H]^-$ 181.1034, found 181.1024.

IR spectrum $\tilde{\nu}$ (cm^{-1}): 3602 ($\nu_{\text{O-H alcohol}}$), 2964 ($\nu_{\text{C-H alkene}}$), 2914-2871 ($\nu_{\text{C-H alkane}}$), 1421 ($\delta_{\text{C-H alkane, methyl group}}$), 1364 ($\delta_{\text{O-H phenol}}$).

m.p.: 32.3–33.8°C.

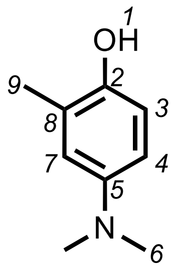

19

2-Amino-2-methylphenol (111.7 mg, 0.91 mmol), paraformaldehyde (479.8 mg, 16 mmol) and sodium cyanoborohydride (319.2 mg, 5.1 mmol) were dissolved in glacial acetic acid (15.2 mL) and water (1.2 mL). The mixture was stirred for 8 hours at room temperature. Then saturated aqueous potassium carbonate was poured into the reaction mixture to adjust the pH to 7-8. It was then extracted with DCM (5x50 mL). The combined organic layers were dried over magnesium sulphate, filtered and dried under reduced pressure. The crude product was purified by flash column chromatography (SiO_2, 0-100% gradient of ethyl acetate in petroleum ether). The desired product was obtained as a red solid (57.0 mg, 3.7 mmol, 42%).

^{1}H-NMR (400 MHz, d_6-DMSO) δ_H (ppm): 8.42 (s, 1H, H(1)), 6.62 (d, J = 8.6 Hz, 1H, H(3)), 6.53 (d, J = 3.0 Hz, 1H, H(7)), 6.43 (dd, J = 8.6, 3.1 Hz, 1H, H(4)), 2.72 (s, 6H, H(6)), 2.08 (s, 3H, H(9)).

^{13}C{^{1}H}-NMR (101 MHz, d_6-DMSO) δ_C (ppm): 147.3 (1C, C(2)), 144.3 (1C, C(5)), 123.9 (1C, C(8)), 116.5 (1C, C(7)), 115.0 (1C, C(3)), 112.0 (1C, C(4)), 41.5 (2C, C(6)), 16.5 (1C, C(9)).

HRMS: calc. for $C_9H_{14}NO^+$ $[M+H]^+$ for 152.1070, found 152.1062.

IR spectrum $\tilde{v}$ (cm^{-1}): 3286 ($v_{O\text{-}H\ alcohol}$), 3036 ($v_{C\text{-}H\ alkene}$), 2948-2793 ($v_{C\text{-}H\ alkane}$), 1422 ($\delta_{C\text{-}H\ alkane,\ methyl\ group}$), 1422 ($\delta_{O\text{-}H\ phenol}$).

m.p.: 78.8-79.8°C.

3. NMR Characterisation

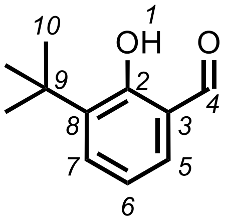

24

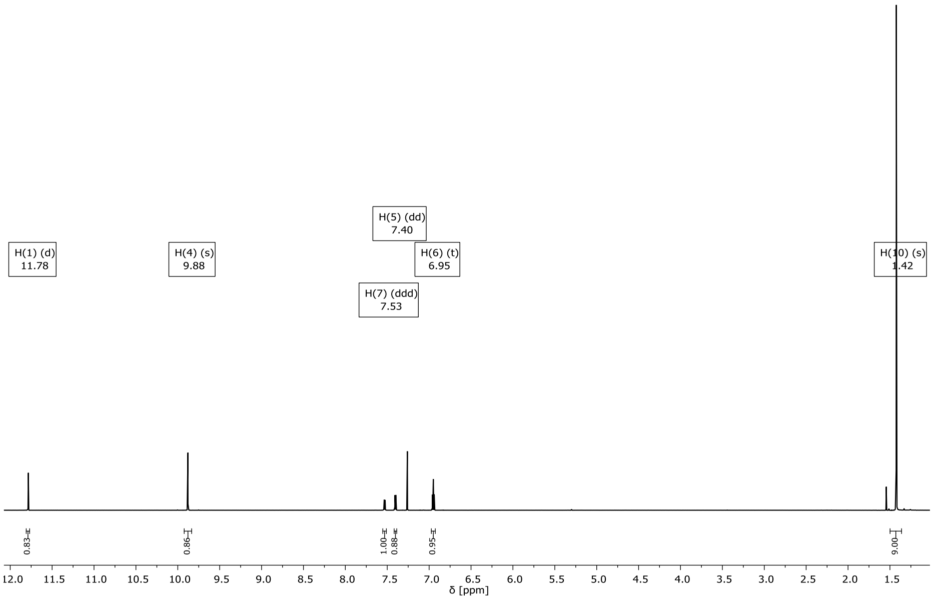

Figure S1 700 MHz ^{1}H-NMR of **24** in $CDCl_3$.

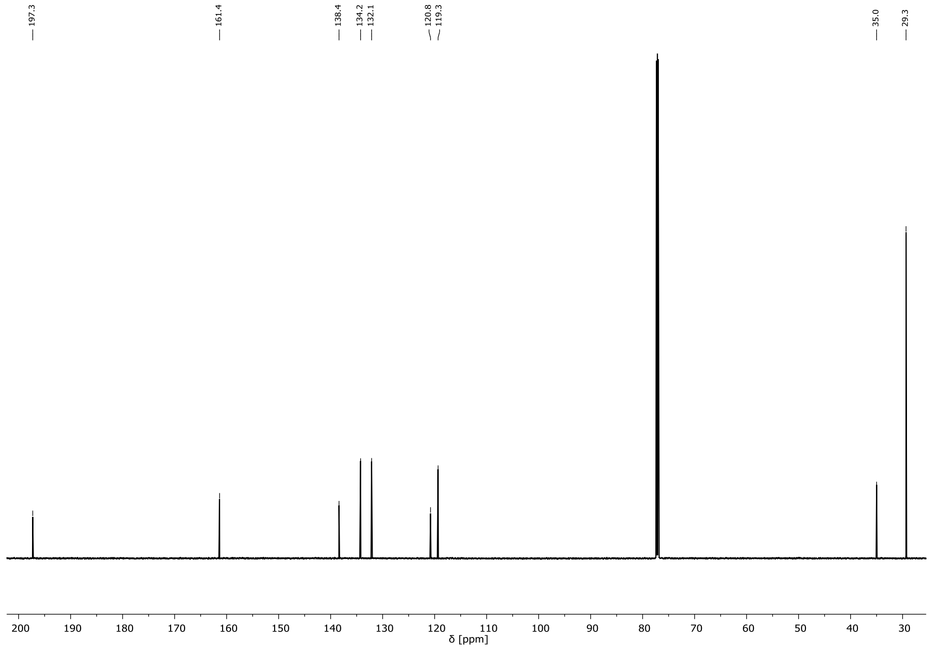

Figure S2 176 MHz ^{13}C-NMR of **24** in $CDCl_3$.

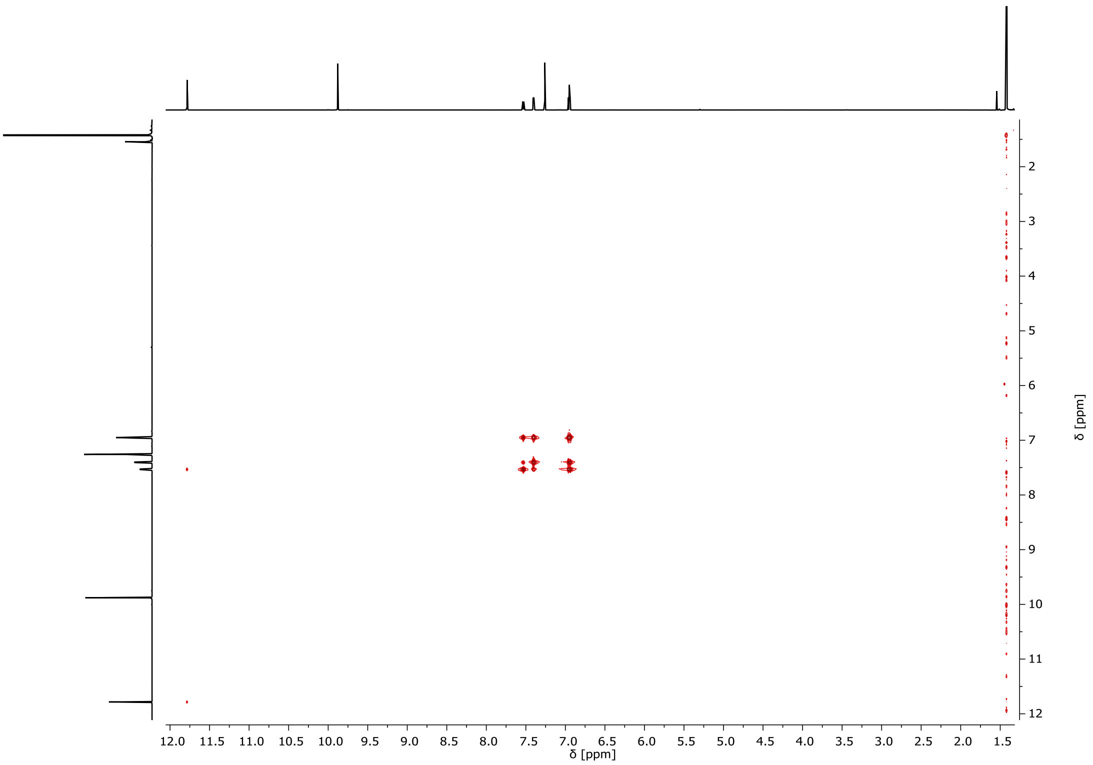

Figure S3 700 MHz ^{1}H-^{1}H COSY spectrum of **24** in $CDCl_3$.

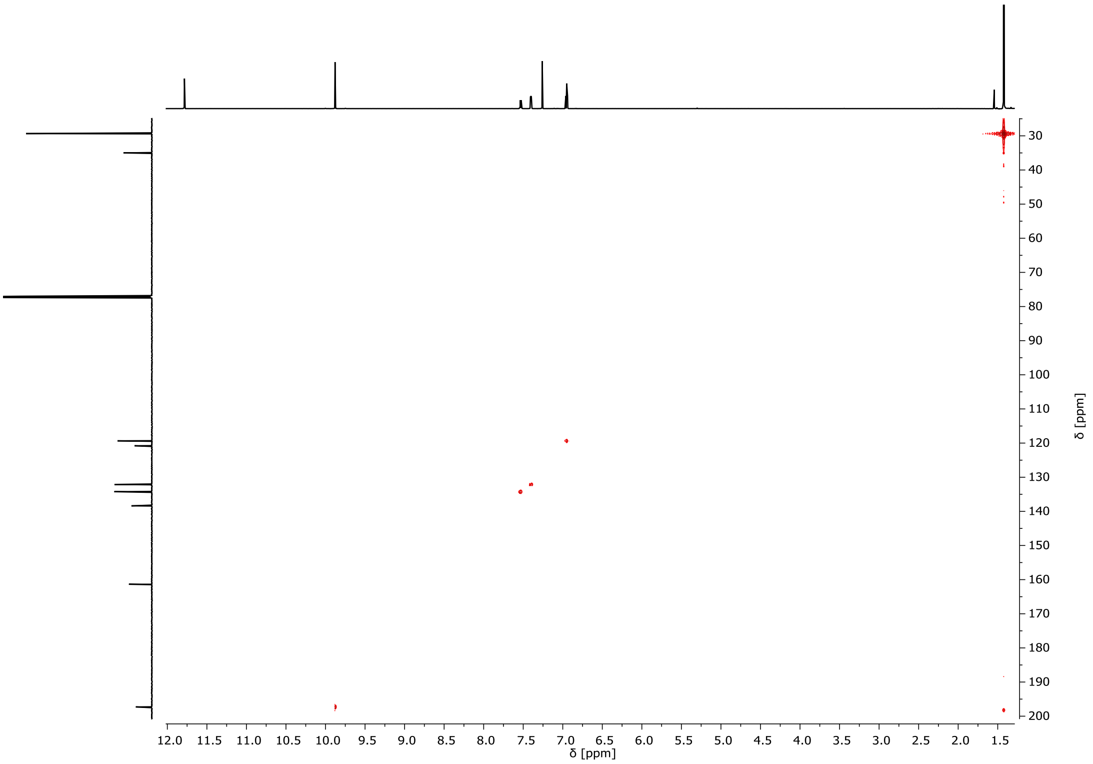

Figure S4 700 MHz ^{1}H-^{13}C Heteronuclear Single Quantum Coherence (HSQC) spectrum of **24** in $CDCl_3$.

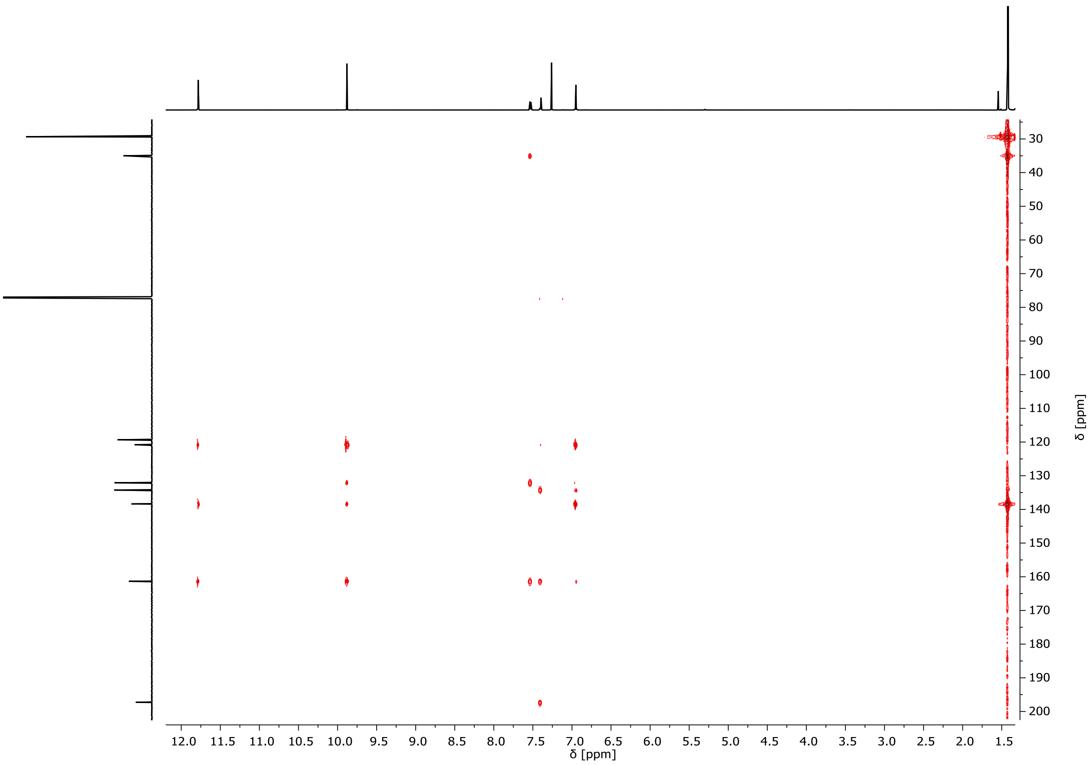

Figure S5 700 MHz ^{1}H-^{13}C Heteronuclear Multiple Bond Correlation (HMBC) spectrum of **24** in $CDCl_3$.

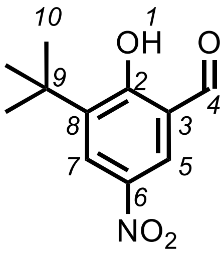

25

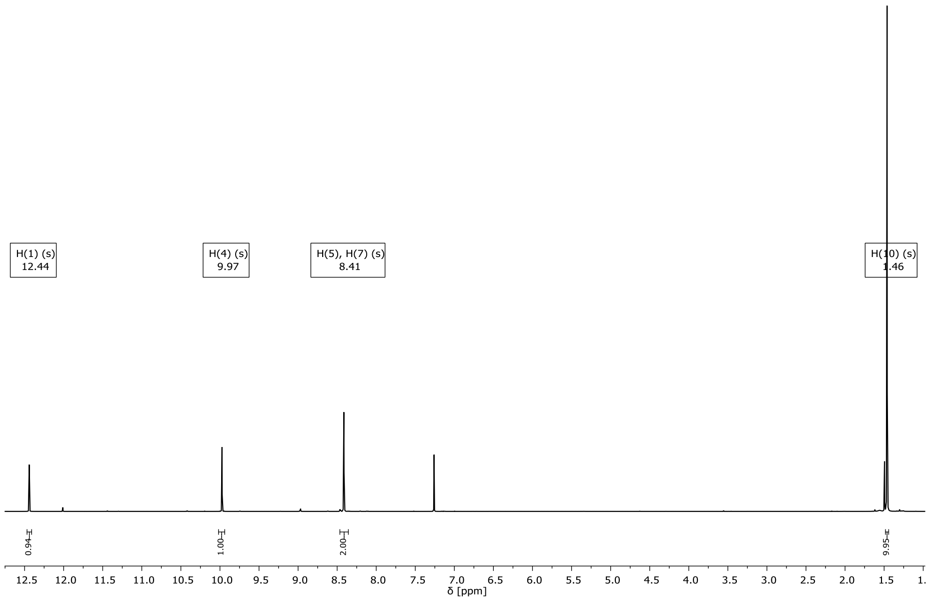

Figure S6 400 MHz ^{1}H-NMR of **25** in $CDCl_3$.

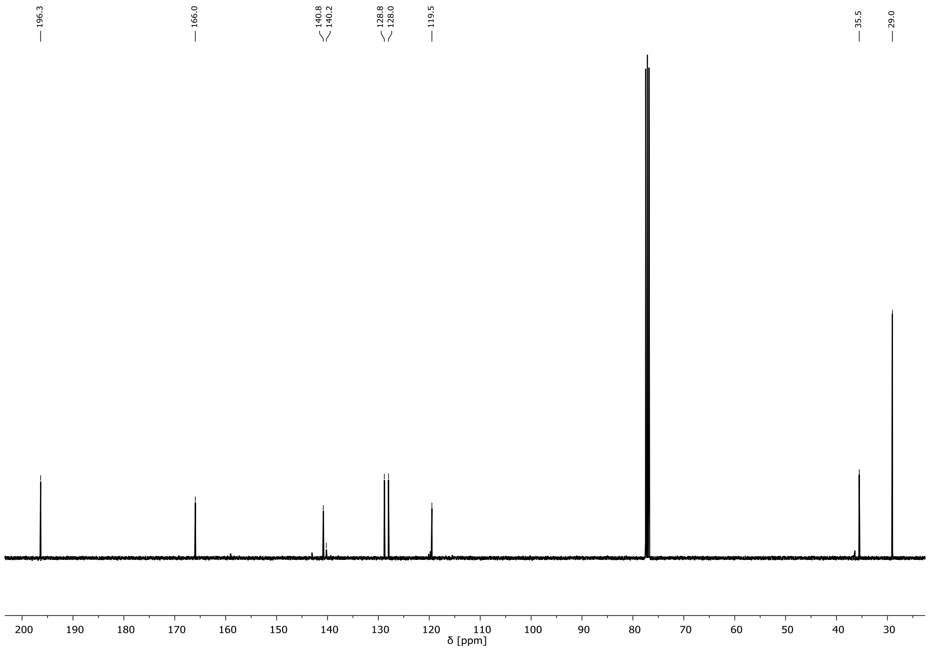

Figure S7 101 MHz ^{13}C-NMR of **25** in $CDCl_3$.

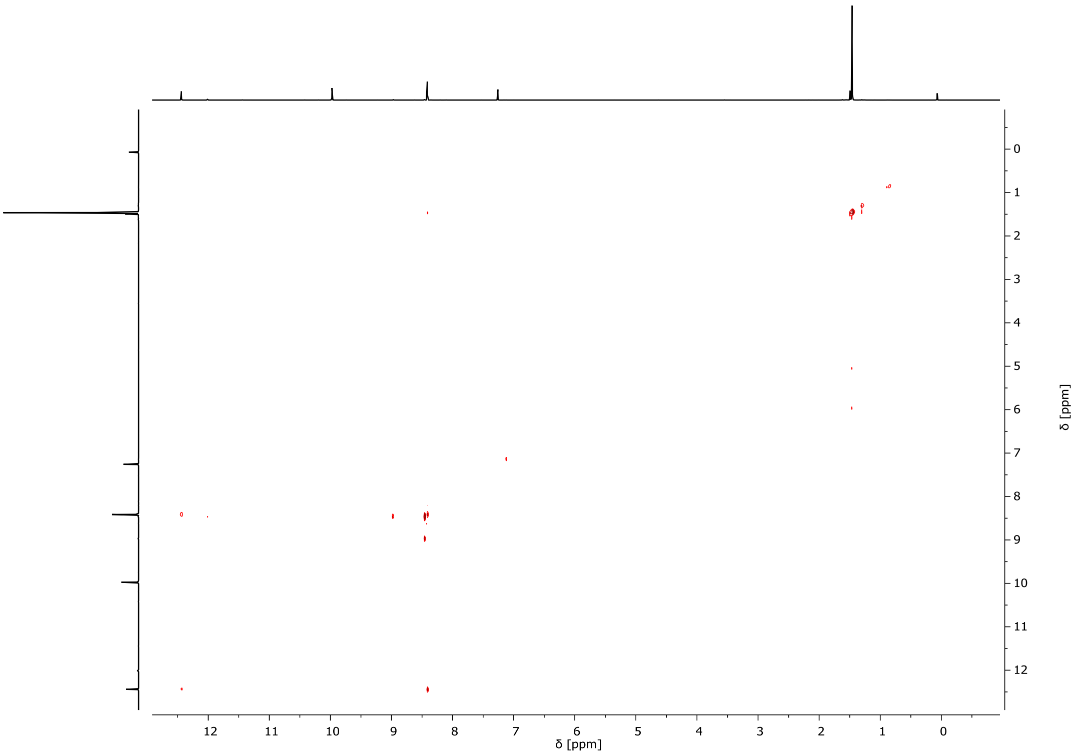

Figure S8 400 MHz ^{1}H-^{1}H COSY spectrum of **25** in $CDCl_3$.

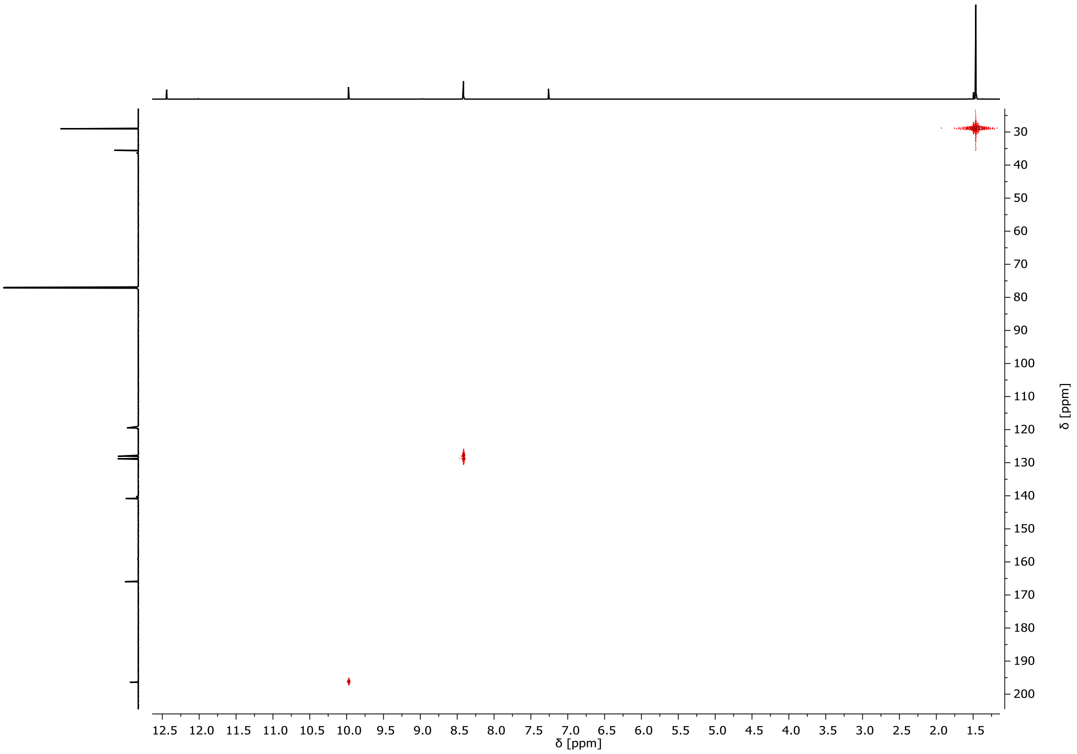

Figure S9 700 MHz ^{1}H-^{13}C Heteronuclear Single Quantum Coherence (HSQC) spectrum of **25** in $CDCl_3$.

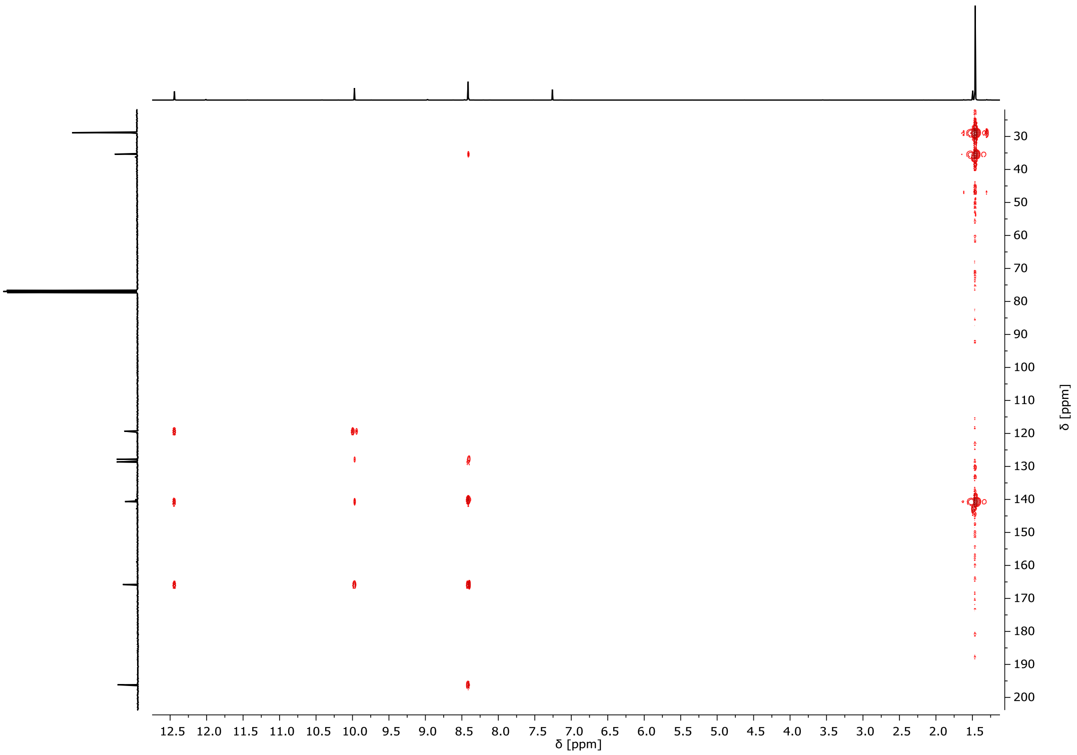

Figure S10 400 MHz ^{1}H-^{13}C Heteronuclear Multiple Bond Correlation (HMBC) spectrum of **25** in $CDCl_3$.

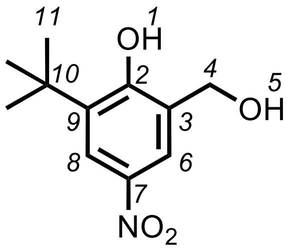

28

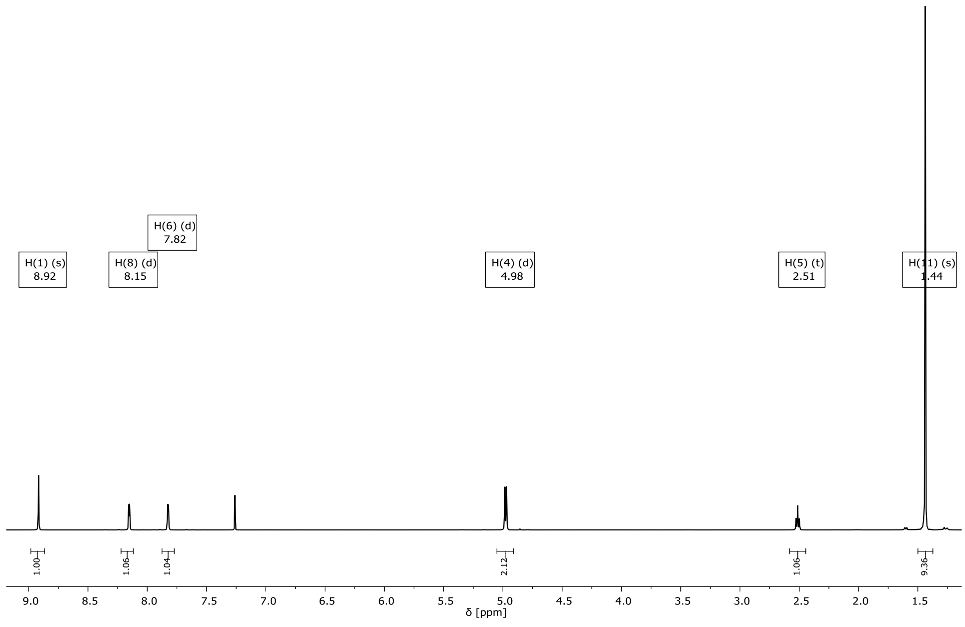

Figure S11 400 MHz ^{1}H-NMR of **28** in $CDCl_3$.

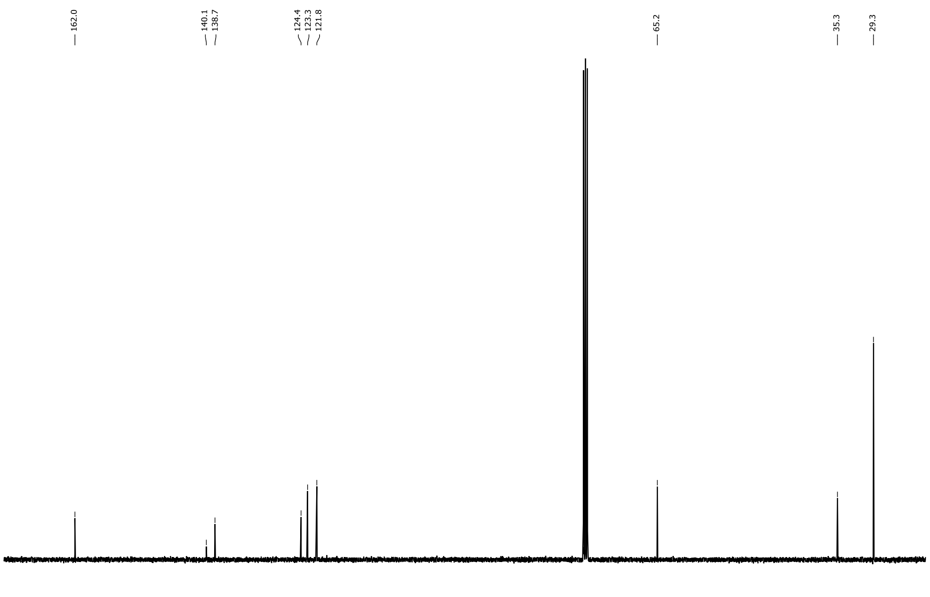

Figure S12 101 MHz ^{13}C-NMR of **28** in $CDCl_3$.

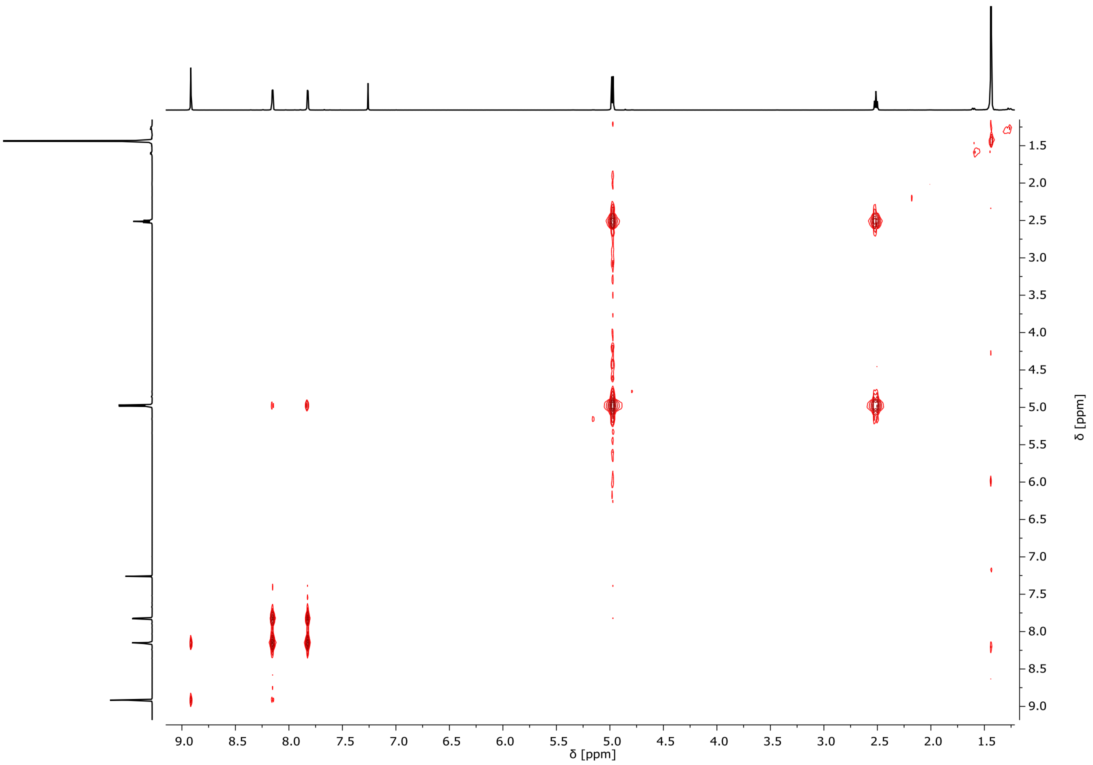

Figure S13 400 MHz ^{1}H-^{1}H COSY spectrum of **28** in $CDCl_3$.

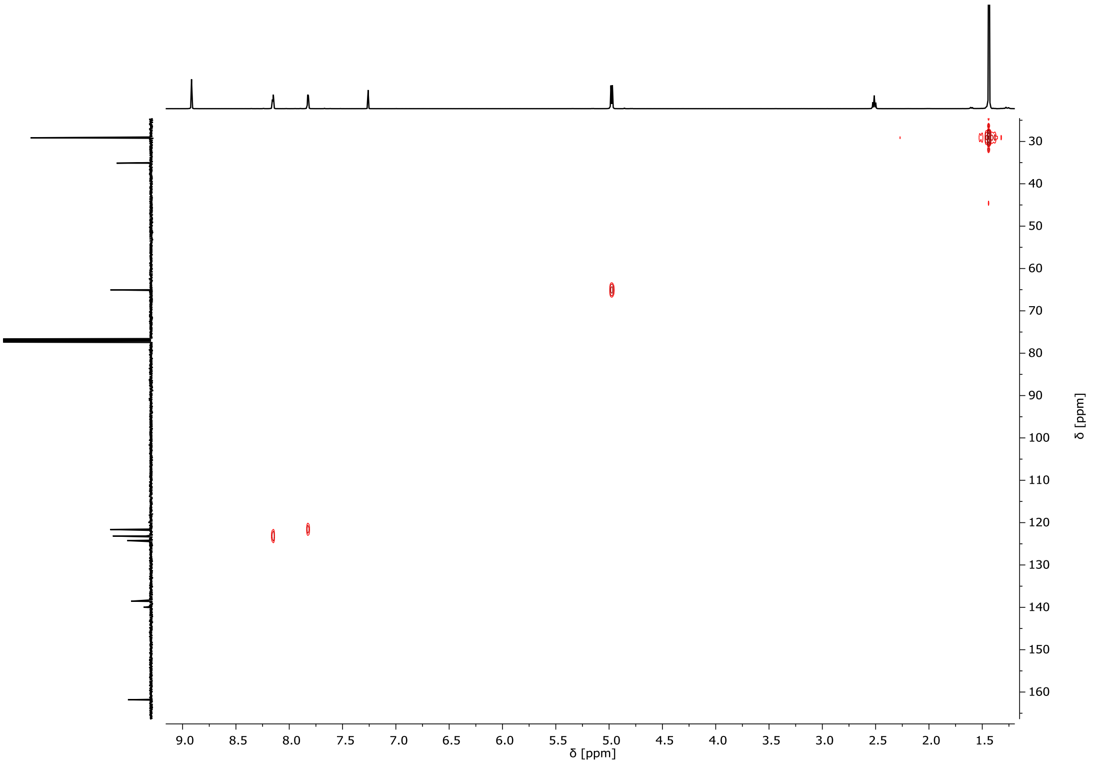

Figure S14 400 MHz ^{1}H-^{13}C Heteronuclear Single Quantum Coherence (HSQC) spectrum of **28** in $CDCl_3$.

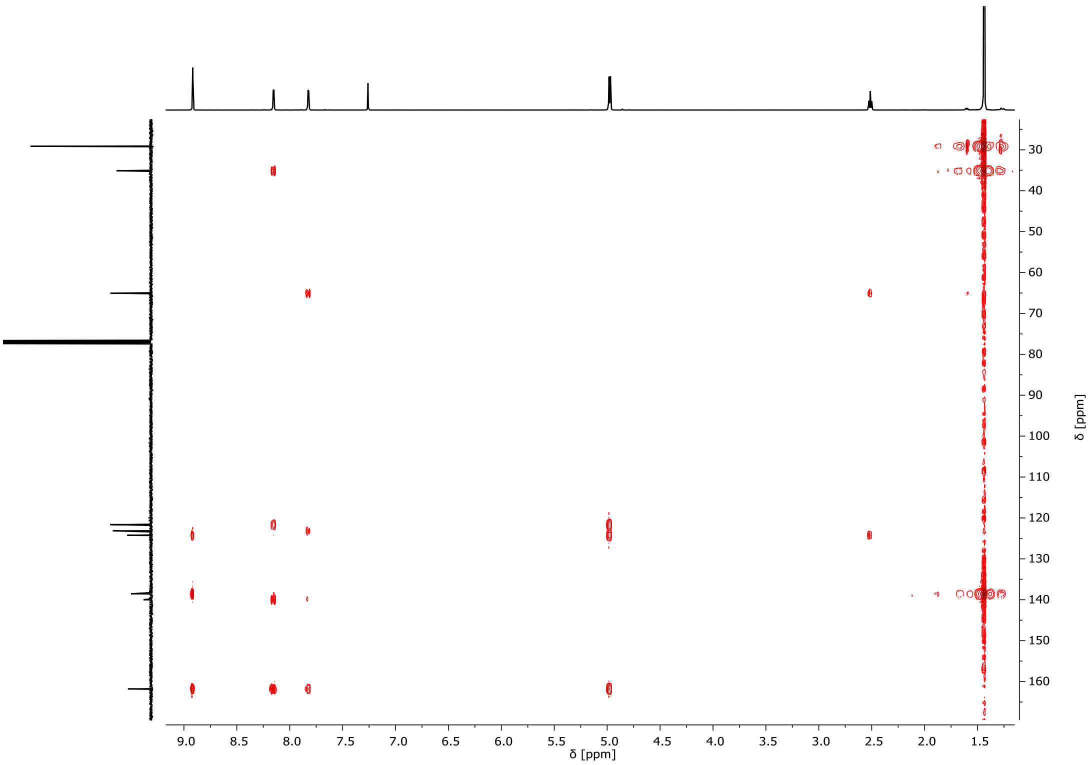

Figure S15 400 MHz ^{1}H-^{13}C Heteronuclear Multiple Bond Correlation (HMBC) spectrum of **28** in $CDCl_3$.

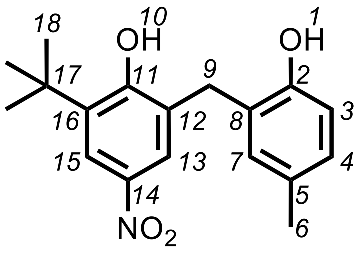

1

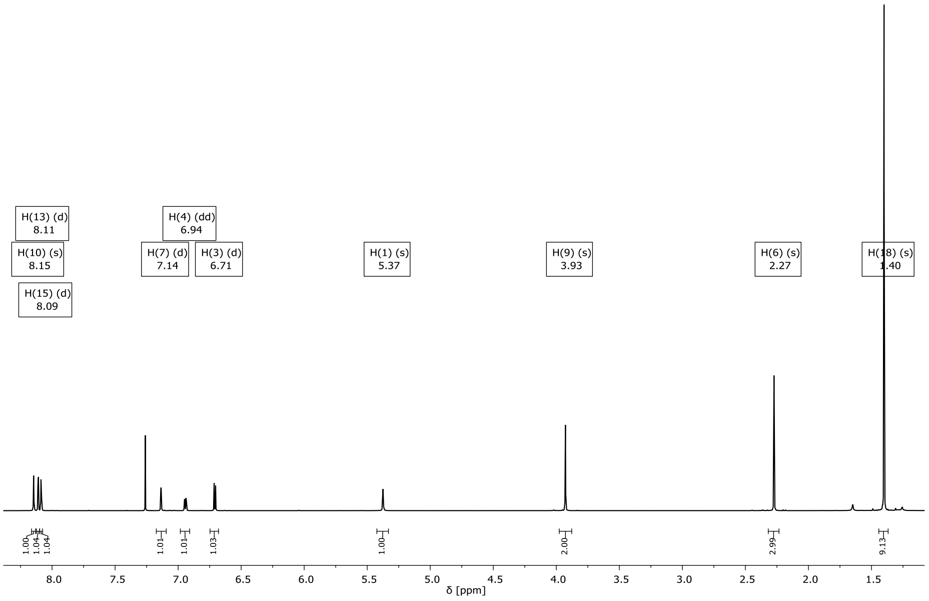

Figure S16 700 MHz ^{1}H-NMR of **1** in $CDCl_3$.

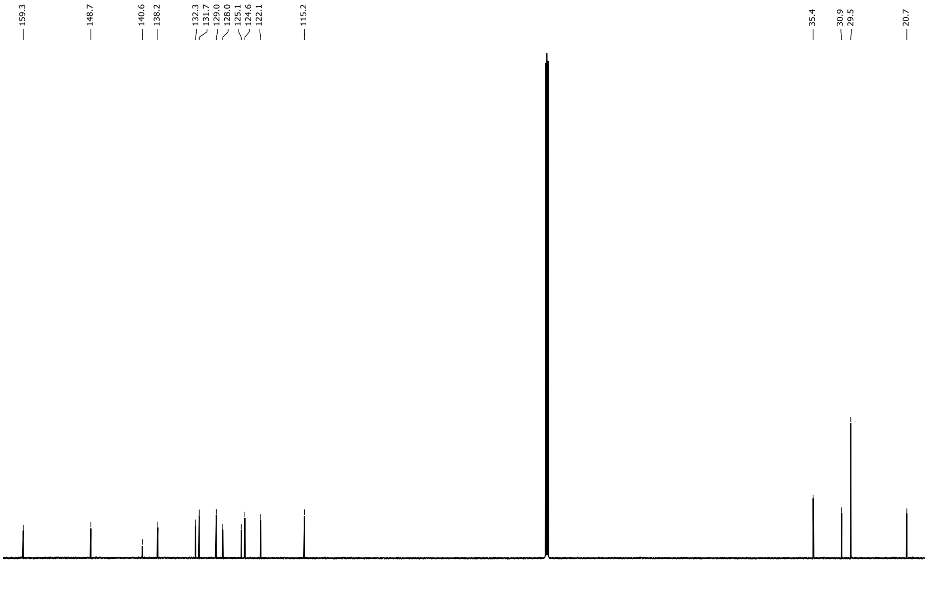

Figure S17 176 MHz ^{13}C-NMR of **1** in $CDCl_3$.

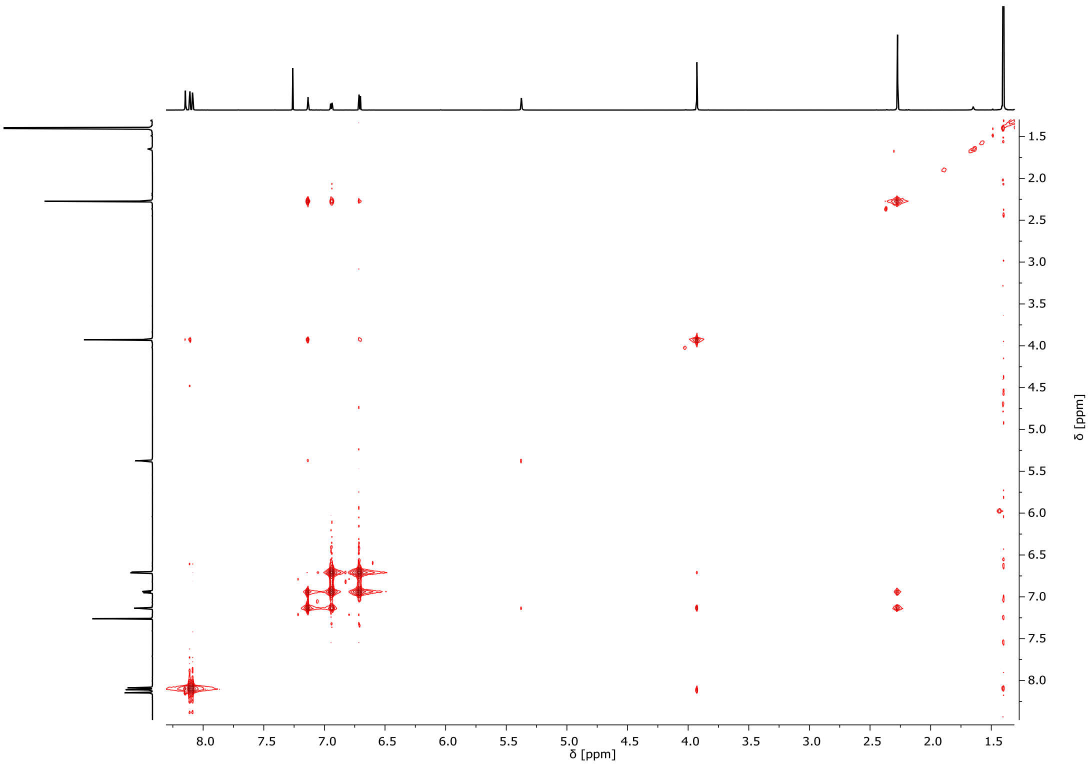

Figure S18 400 MHz ^{1}H-^{1}H COSY spectrum of **1** in $CDCl_3$.

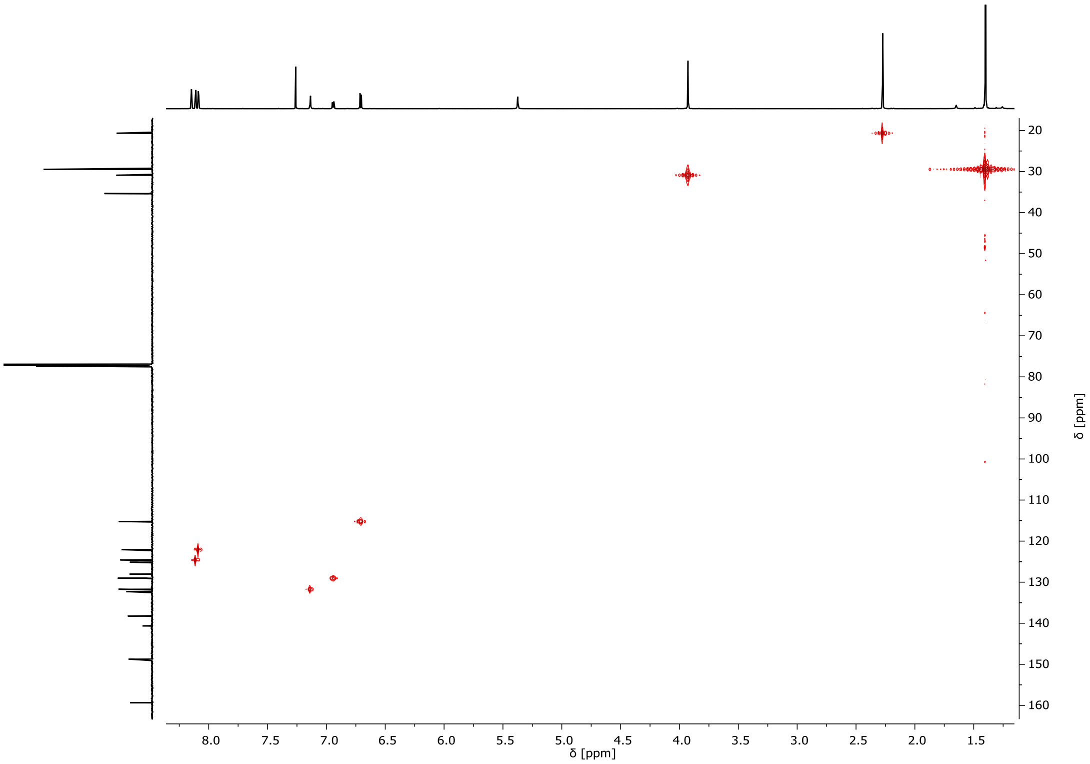

Figure S19 400 MHz ^{1}H-^{13}C Heteronuclear Single Quantum Coherence (HSQC) spectrum of **1** in $CDCl_3$.

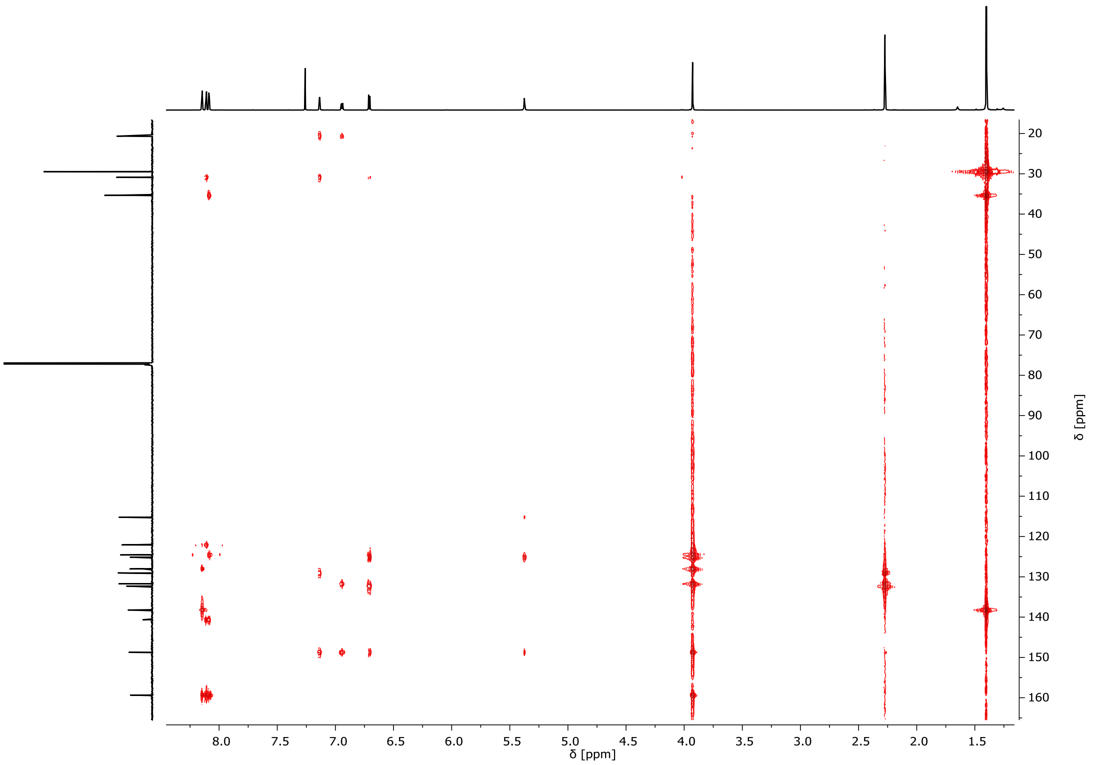

Figure S20 400 MHz ^{1}H-^{13}C Heteronuclear Multiple Bond Correlation (HMBC) spectrum of **1** in $CDCl_3$.

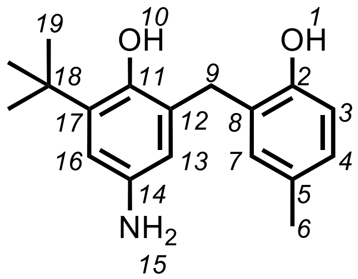

32

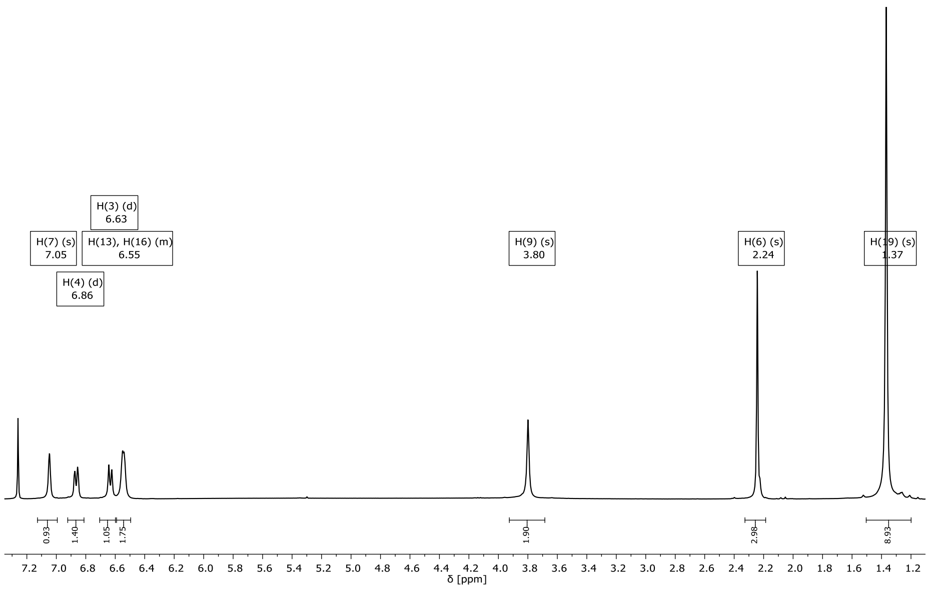

Figure S21 400 MHz ^{1}H-NMR of **32** in $CDCl_3$.

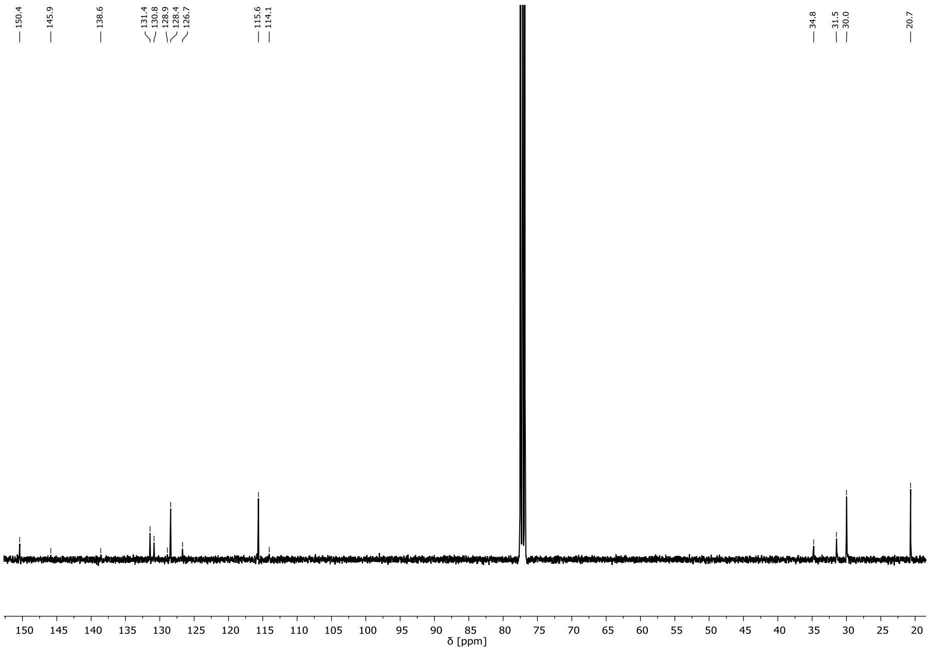

Figure S22 101 MHz ^{13}C-NMR of **32** in $CDCl_3$.

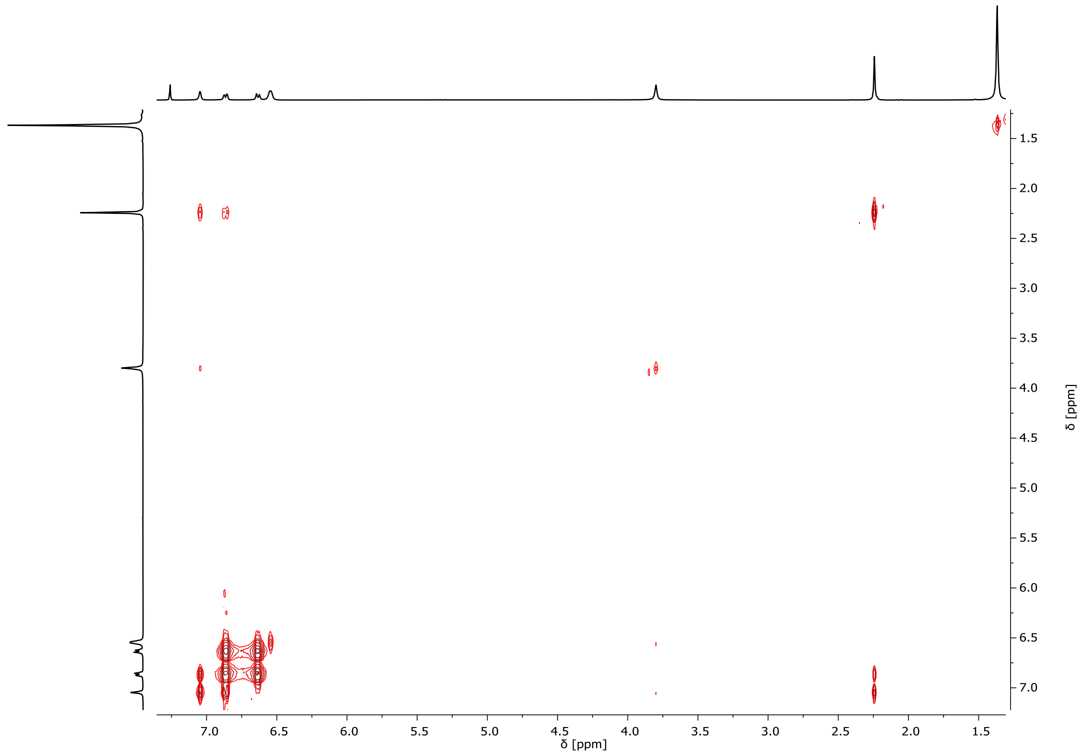

Figure S23 400 MHz ^{1}H-^{1}H COSY spectrum of **32** in $CDCl_3$.

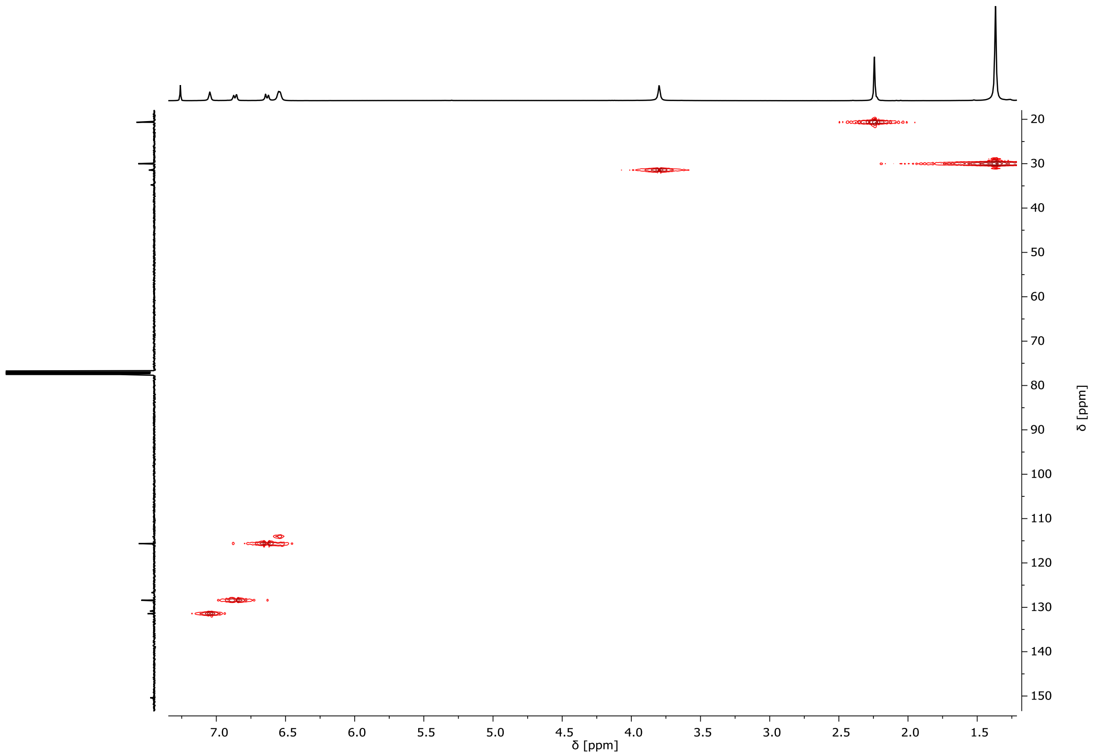

Figure S24 400 MHz ^{1}H-^{13}C Heteronuclear Single Quantum Coherence (HSQC) spectrum of **32** in $CDCl_3$.

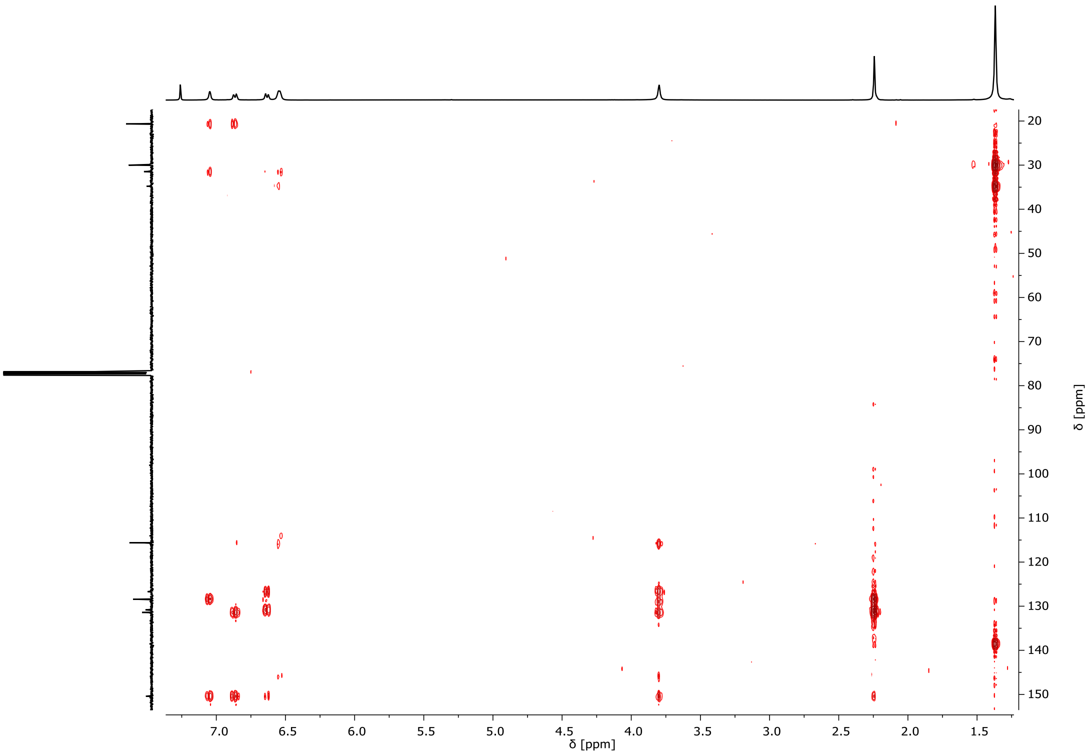

Figure S25 400 MHz ^{1}H-^{13}C Heteronuclear Multiple Bond Correlation (HMBC) spectrum of **32** in $CDCl_3$.

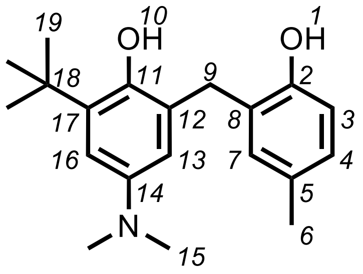

5

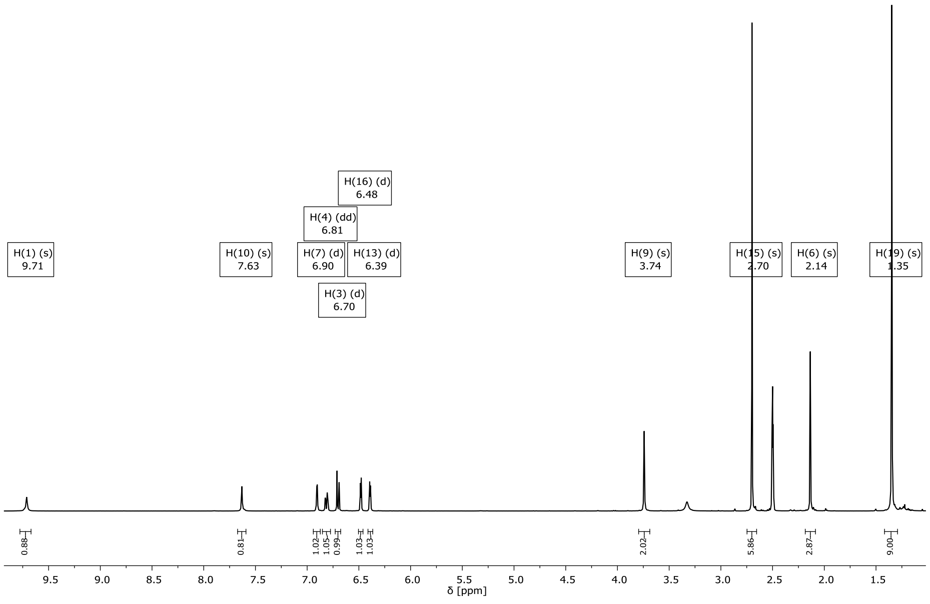

Figure S26 400 MHz ^{1}H-NMR of **5** in d_6-DMSO.

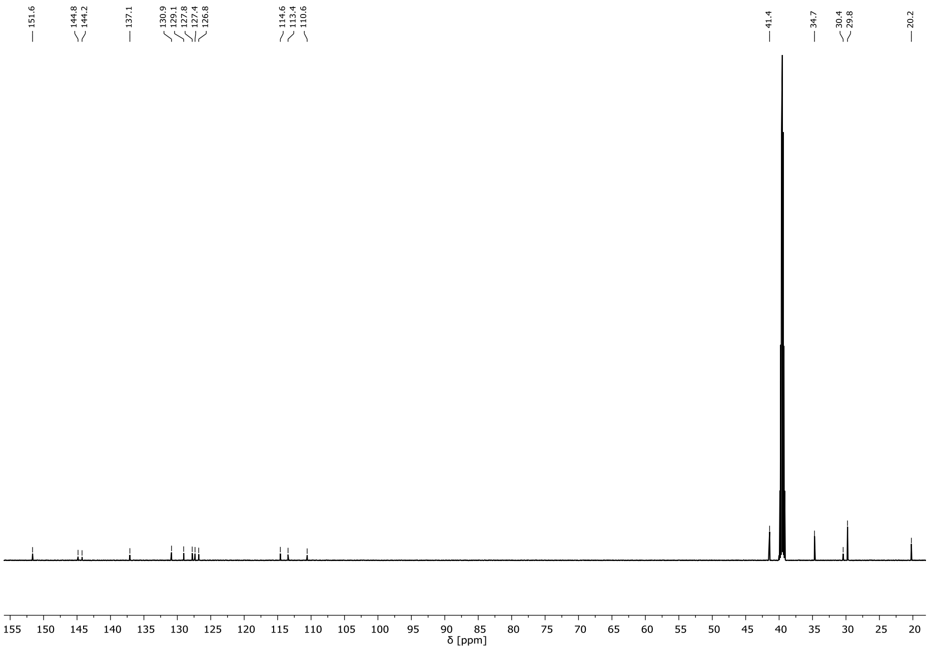

Figure S27 176 MHz ^{13}C-NMR of **5** in d_6-DMSO.

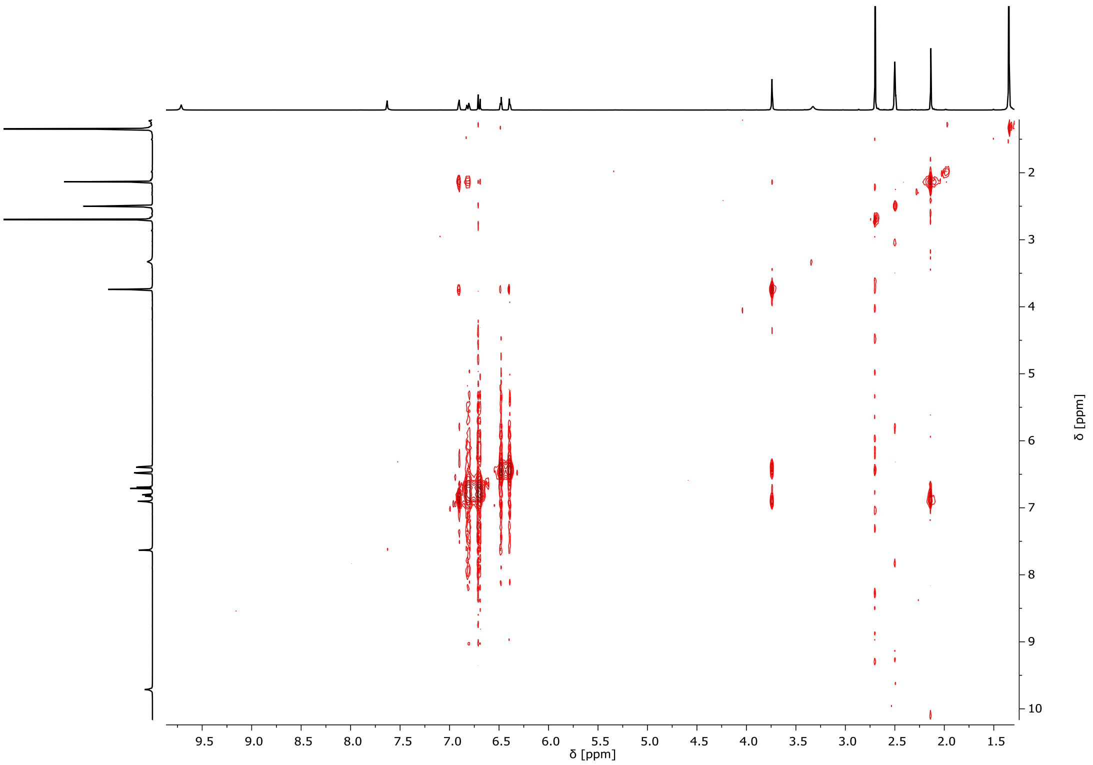

Figure S28 400 MHz ^{1}H-^{1}H COSY spectrum of **5** in d_6-DMSO.

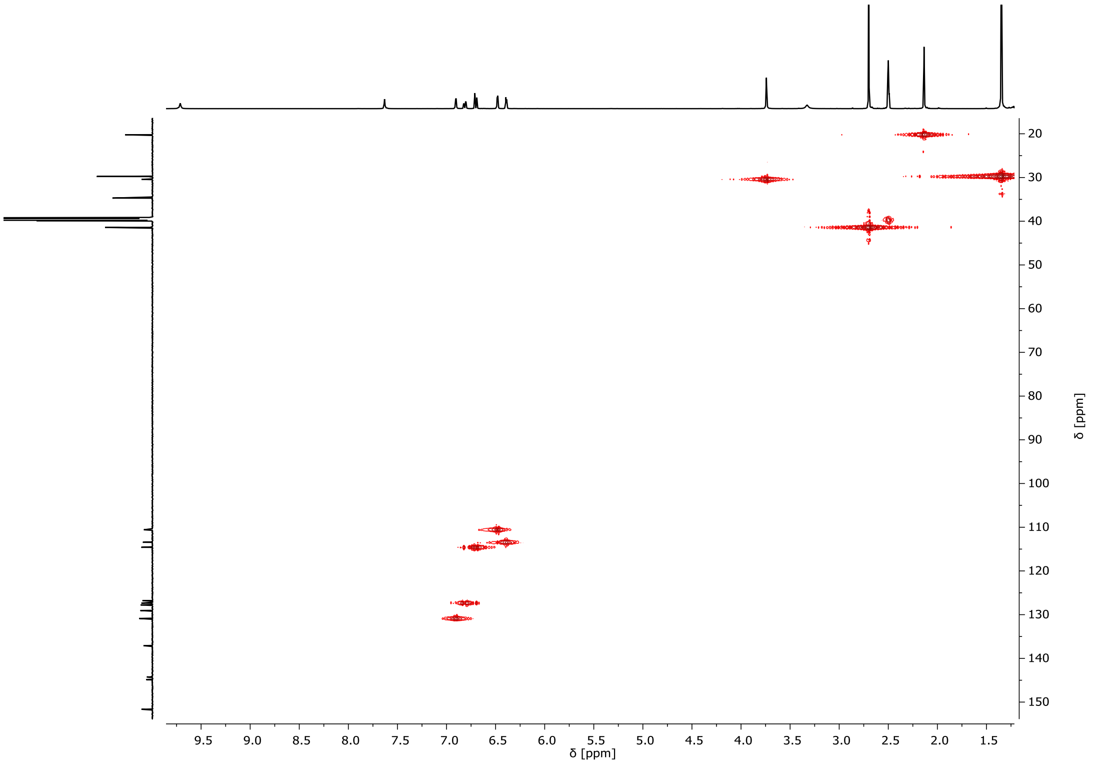

Figure S29 400 MHz ^{1}H-^{13}C Heteronuclear Single Quantum Coherence (HSQC) spectrum of **5** in d_6-DMSO.

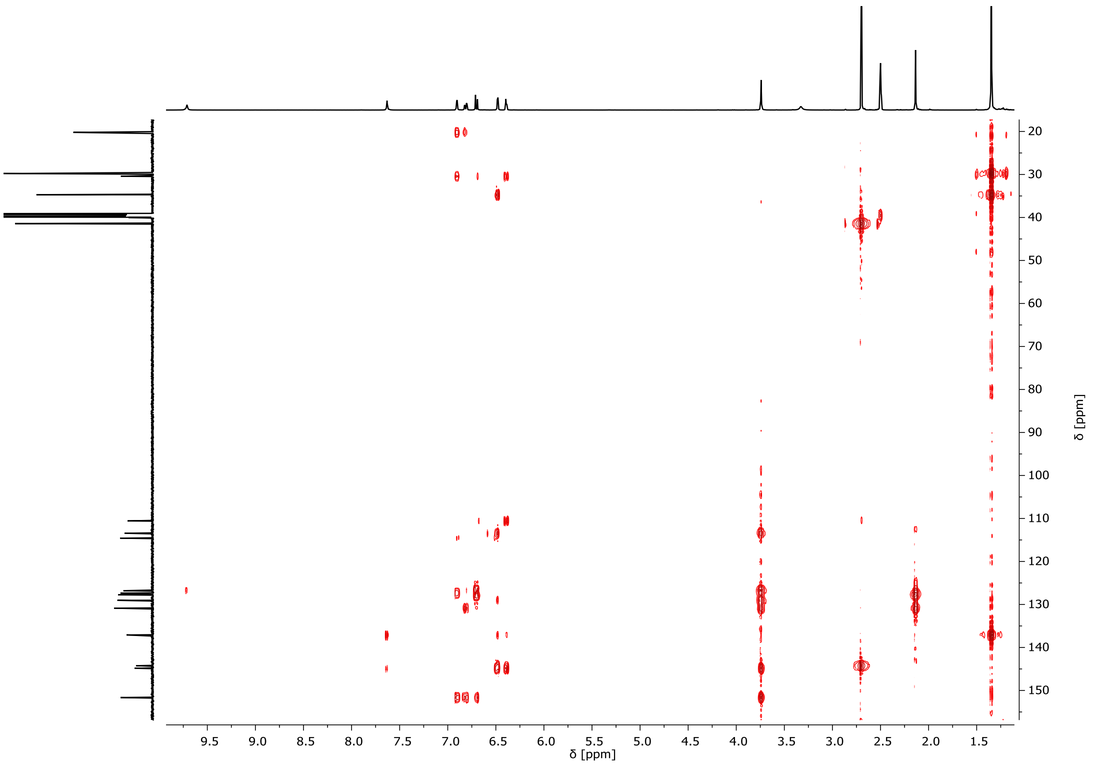

Figure S30 400 MHz ^{1}H-^{13}C Heteronuclear Multiple Bond Correlation (HMBC) spectrum of **5** in d_6-DMSO.

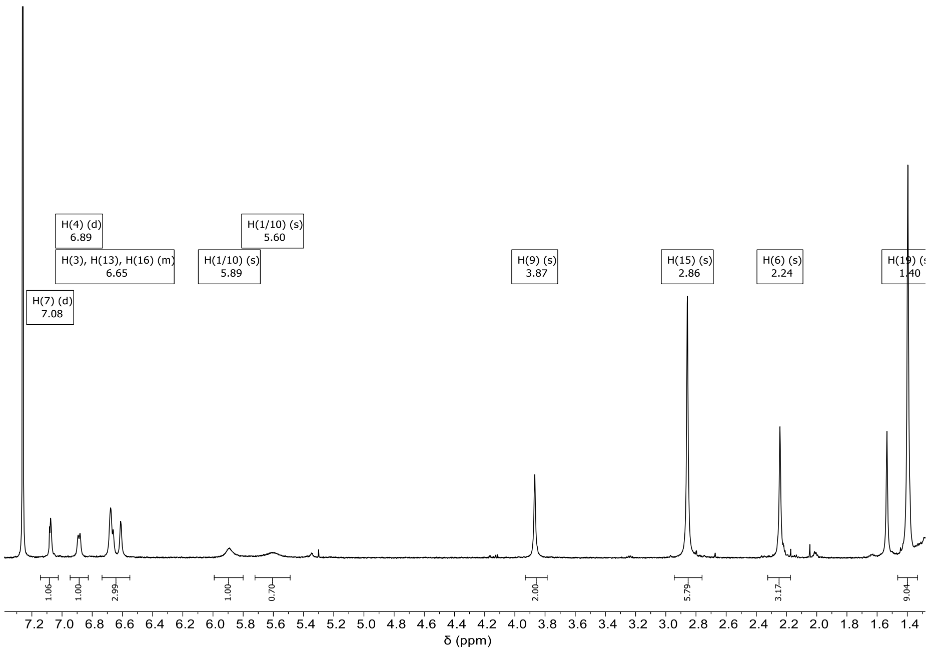

Figure S31 600 MHz ^{1}H-NMR of **5** in $CDCl_3$.

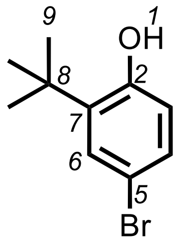

22

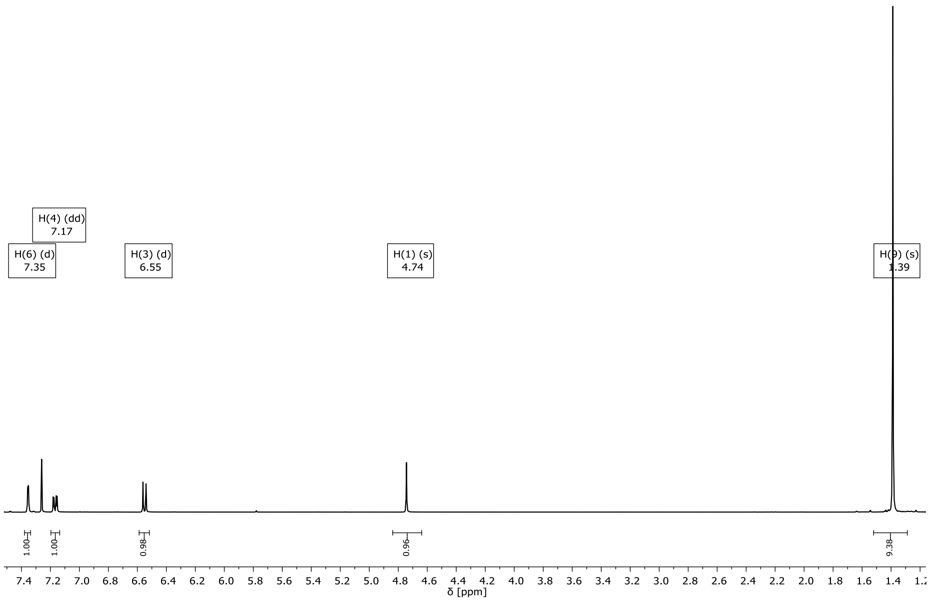

Figure S32 400 MHz ^{1}H-NMR of **22** in $CDCl_3$.

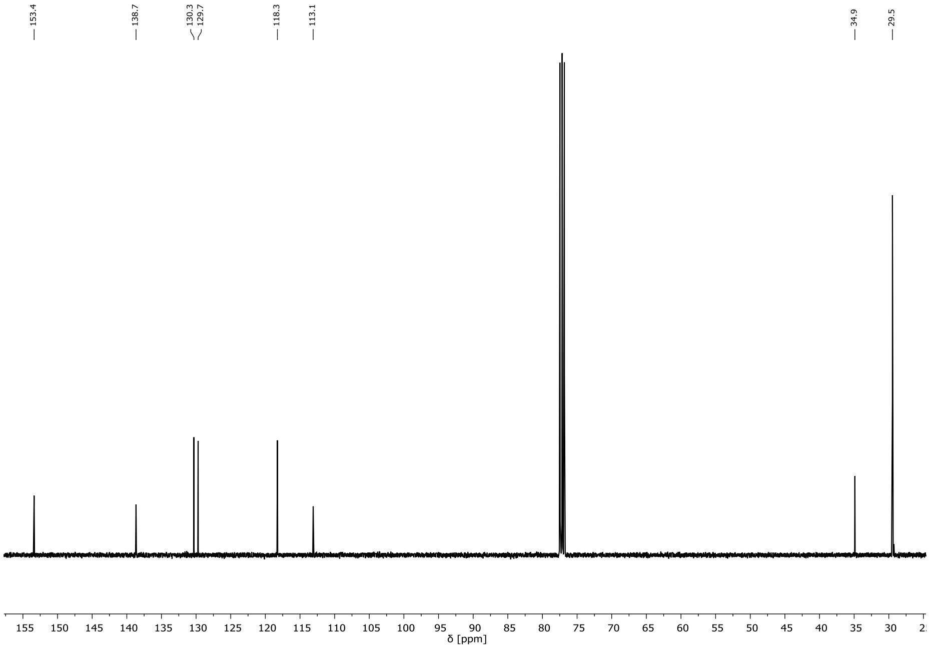

Figure S33 101 MHz ^{13}C-NMR of **22** in $CDCl_3$.

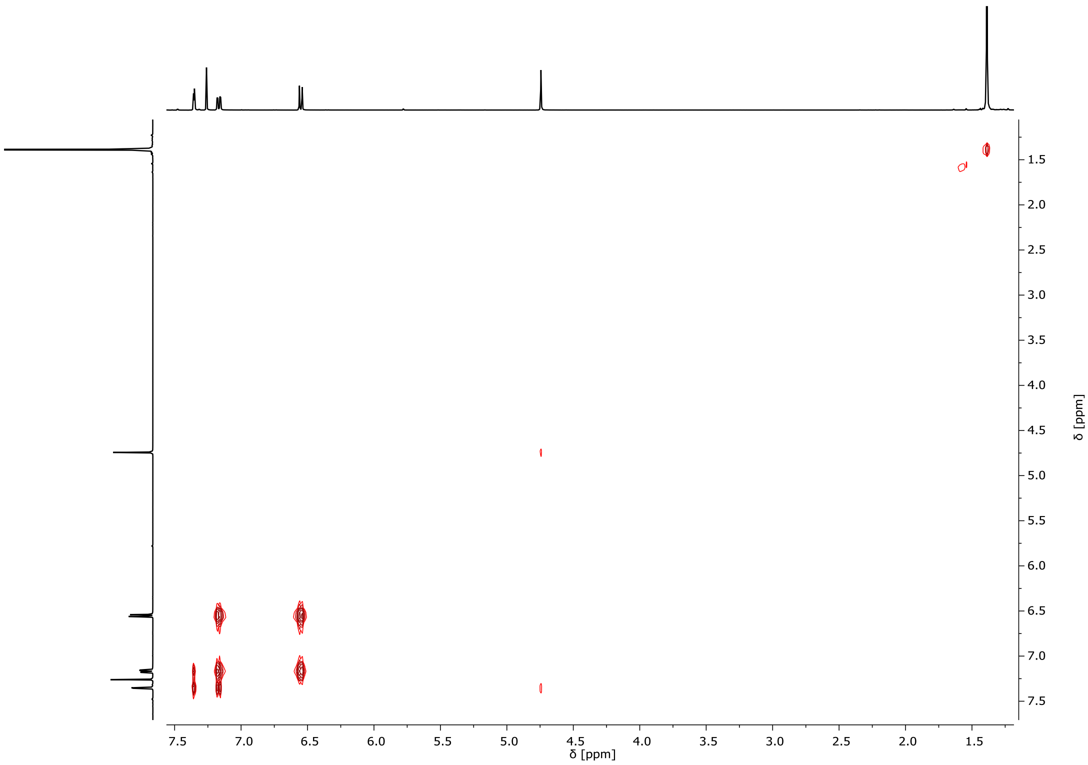

Figure S34 400 MHz ^{1}H-^{1}H COSY spectrum of **22** in $CDCl_3$.

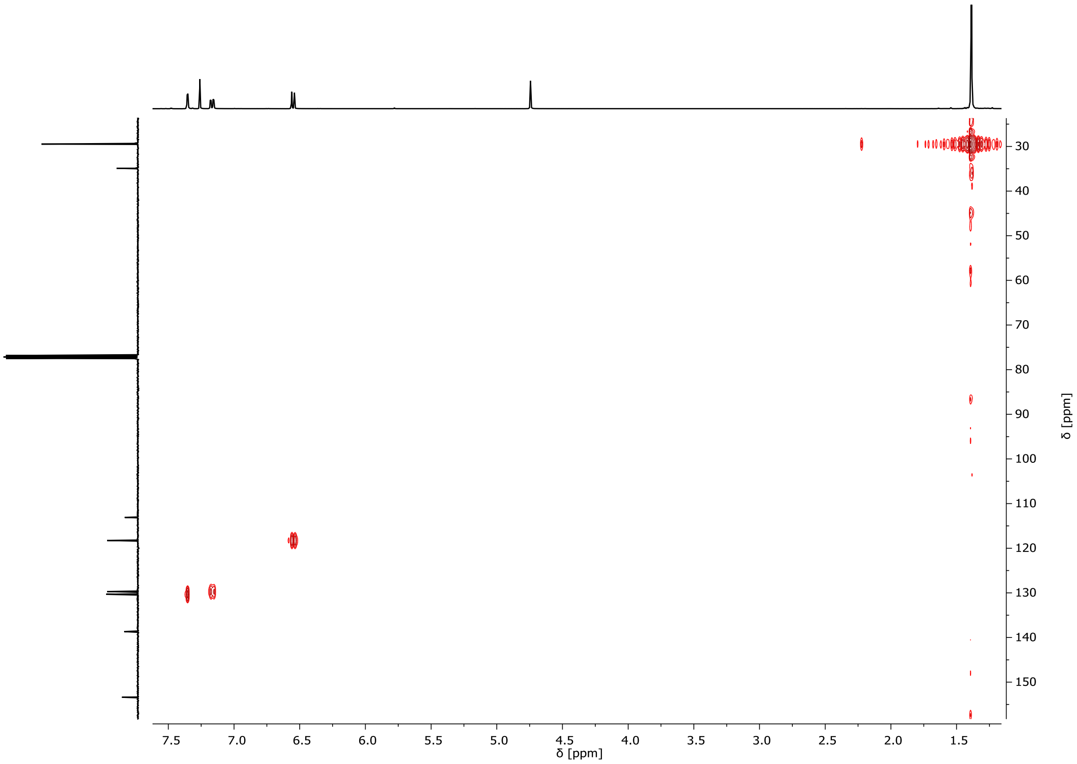

Figure S35 400 MHz ^{1}H-[13]C Heteronuclear Single Quantum Coherence (HSQC) spectrum of **22** in $CDCl_3$.

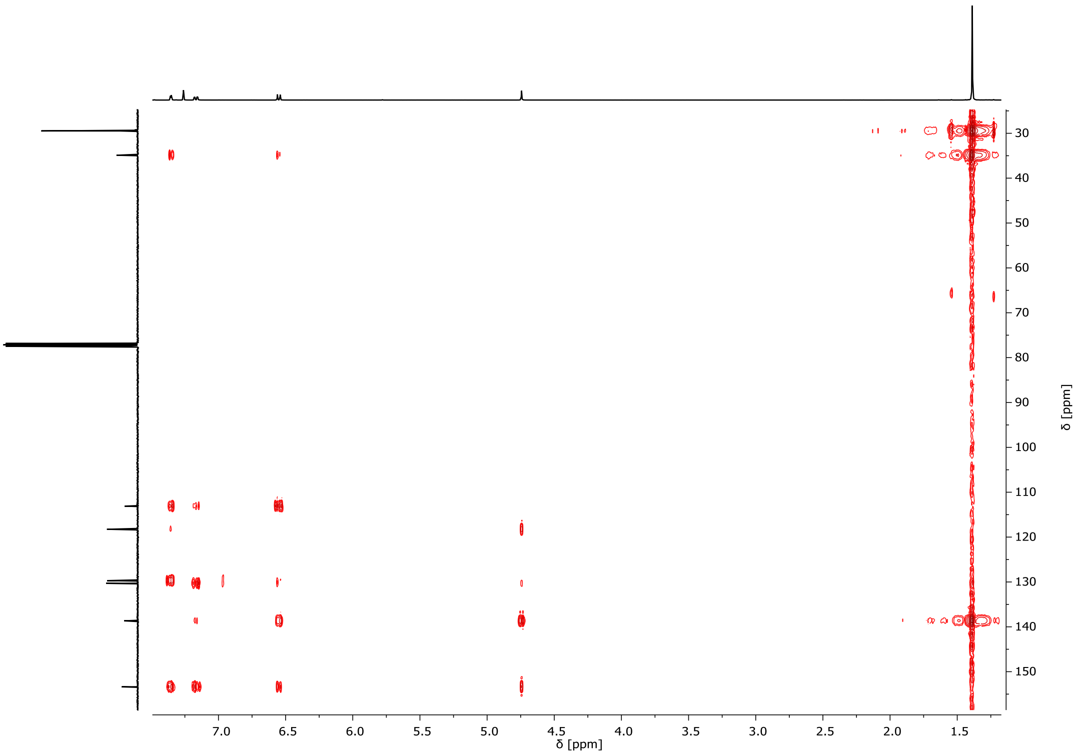

Figure S36 400 MHz ^{1}H-^{13}C Heteronuclear Multiple Bond Correlation (HMBC) spectrum of **22** in $CDCl_3$.

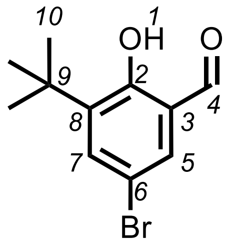

26

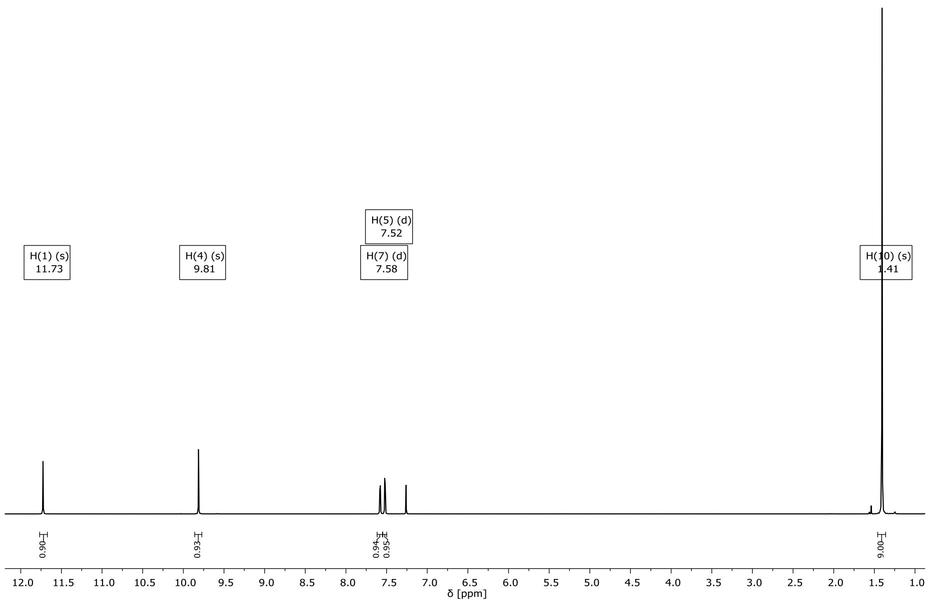

Figure S37 400 MHz ^{1}H-NMR of **26** in $CDCl_3$.

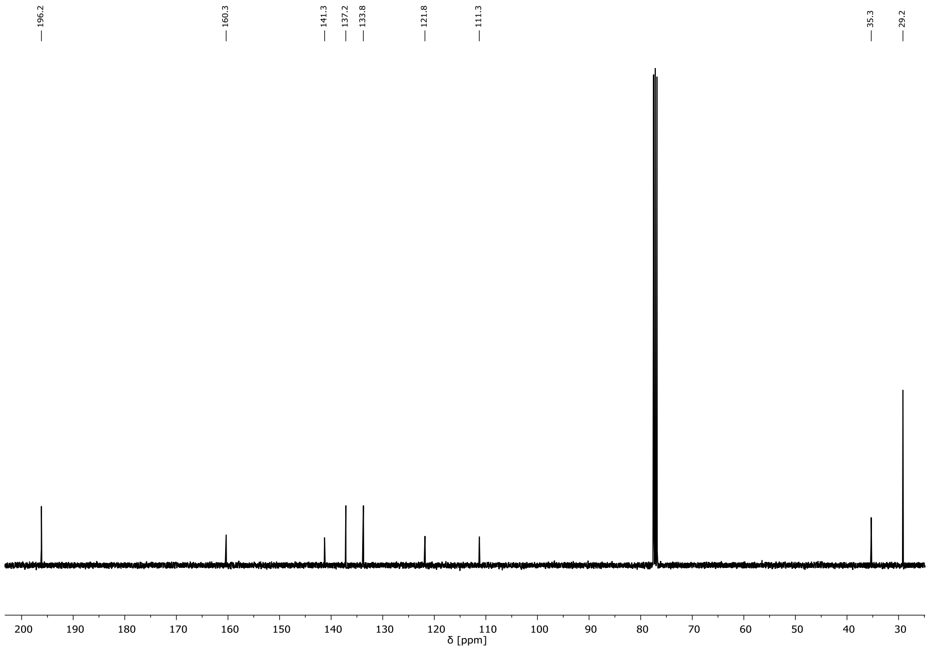

Figure S38 101 MHz ^{13}C-NMR of **26** in $CDCl_3$.

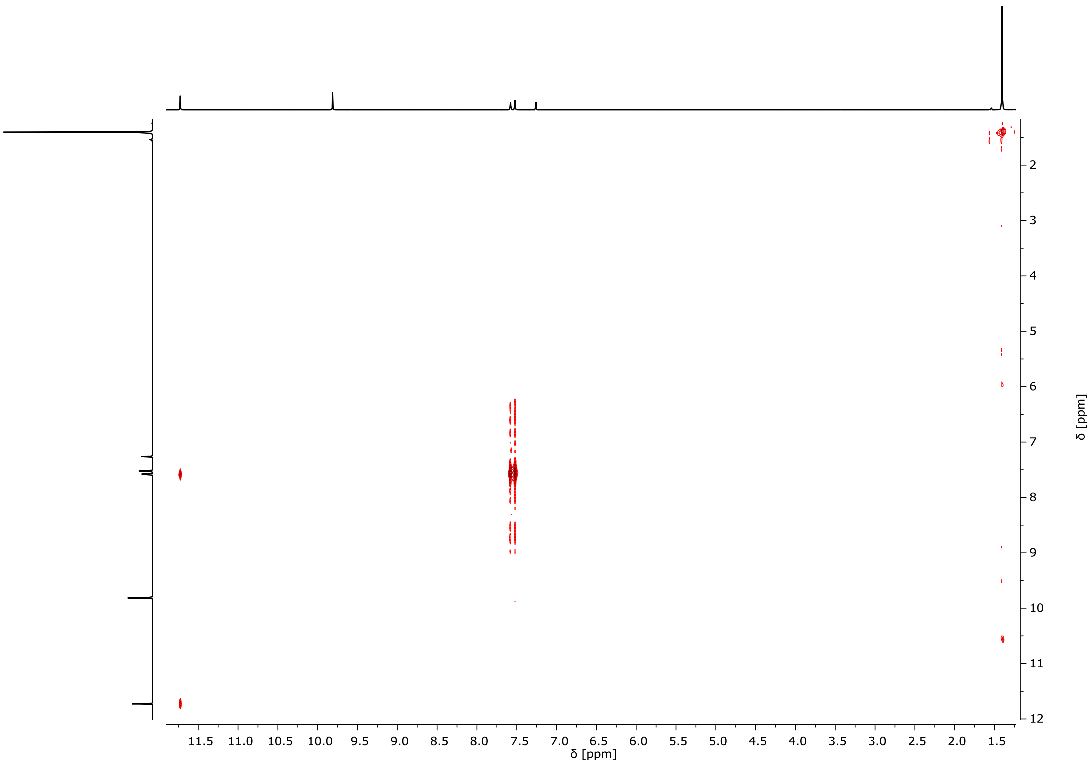

Figure S39 400 MHz ^{1}H-^{1}H COSY spectrum of **26** in $CDCl_3$.

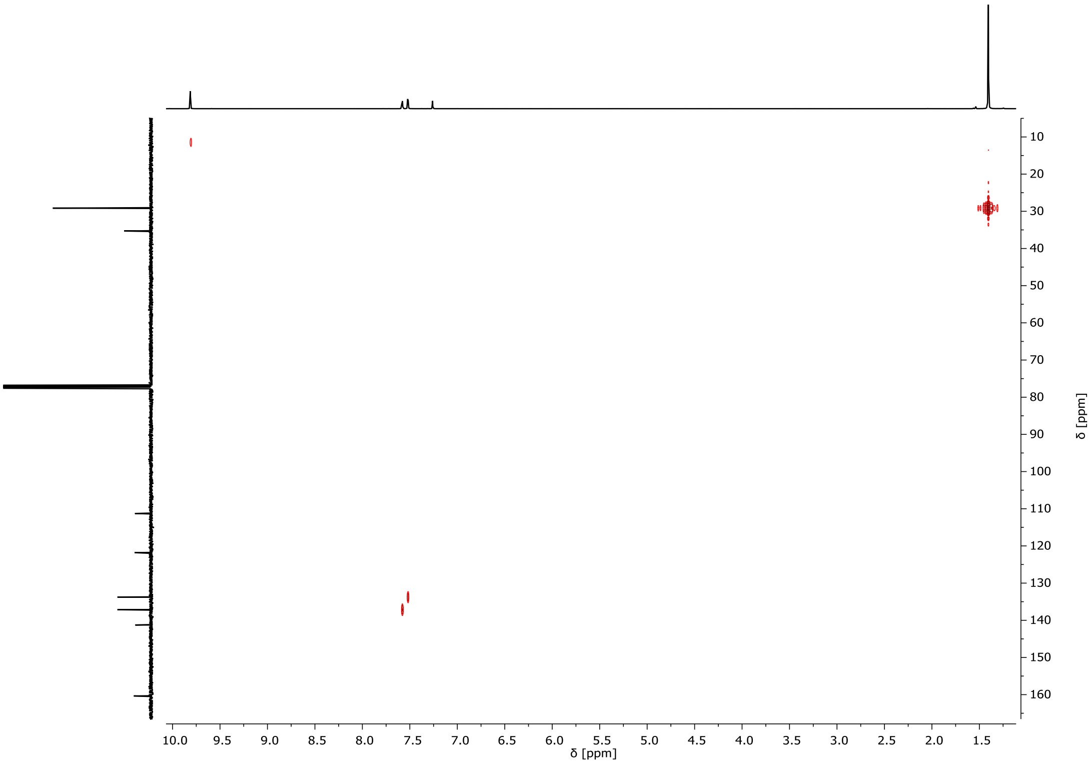

Figure S40 400 MHz ^{1}H-[13]C Heteronuclear Single Quantum Coherence (HSQC) spectrum of **26** in $CDCl_3$.

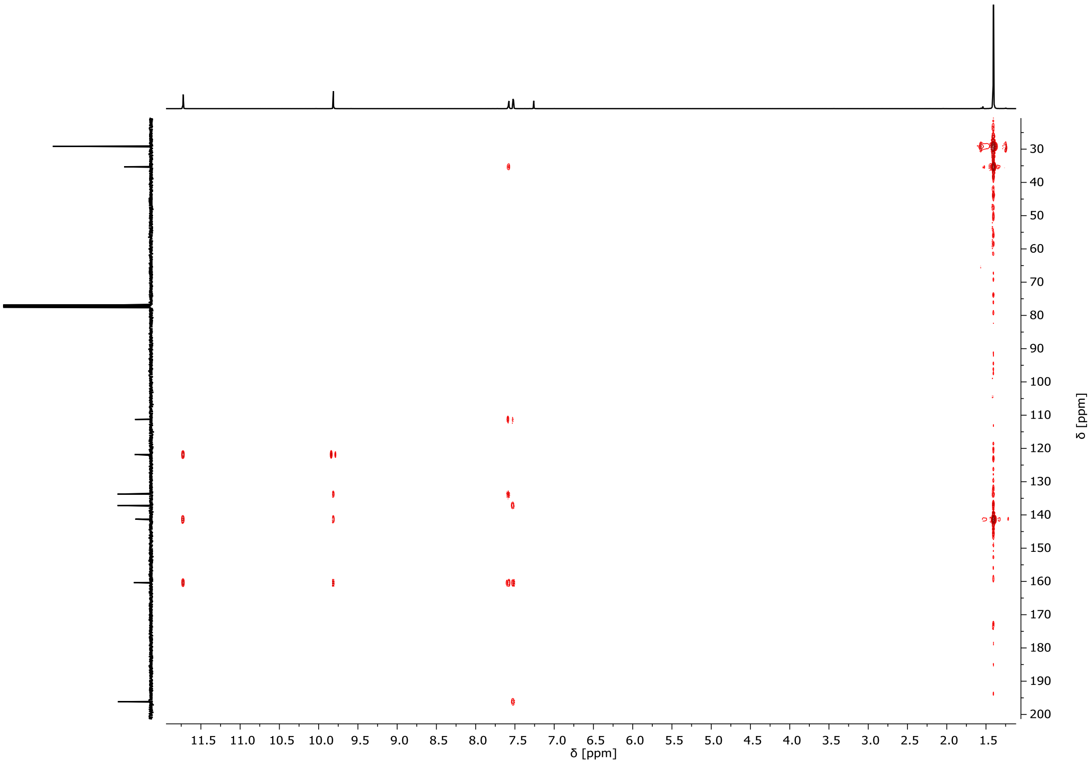

Figure S41 400 MHz ^{1}H-^{13}C Heteronuclear Multiple Bond Correlation (HMBC) spectrum of **26** in $CDCl_3$.

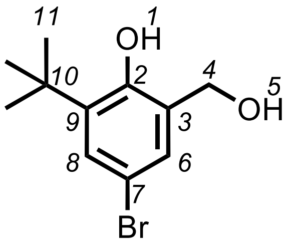

29

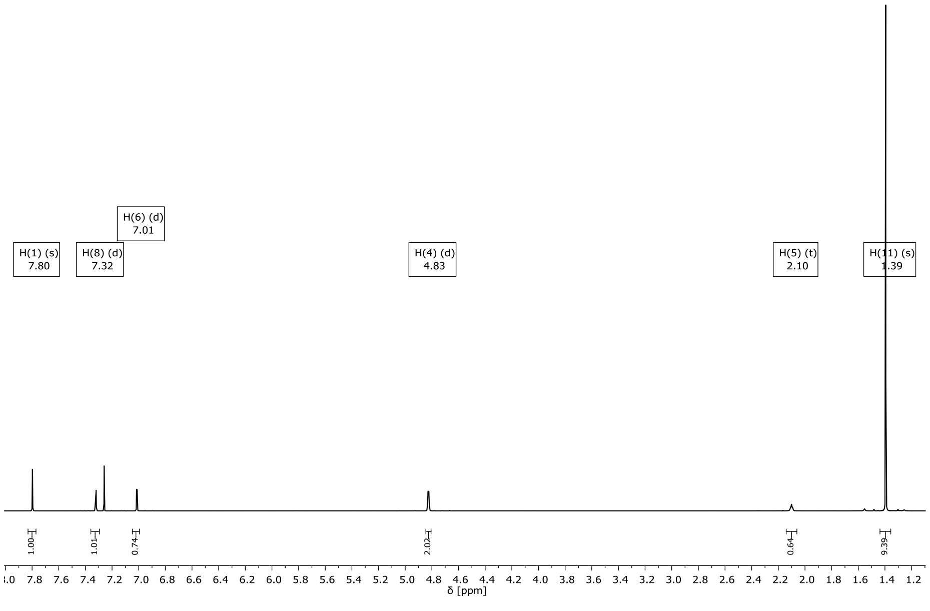

Figure S42 700 MHz ^{1}H-NMR of **29** in $CDCl_3$.

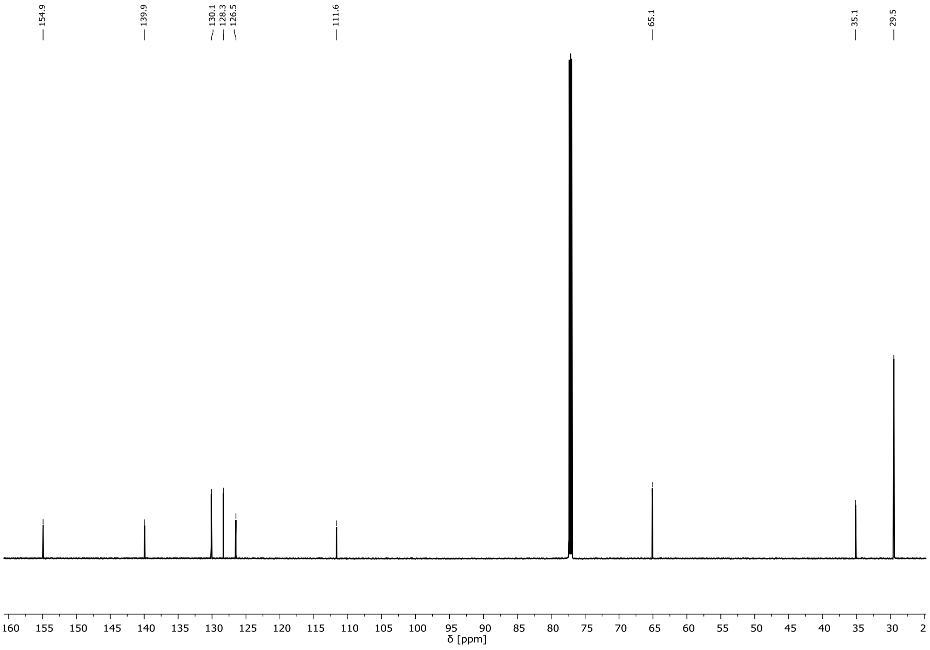

Figure S43 176 MHz ^{13}C-NMR of **29** in $CDCl_3$.

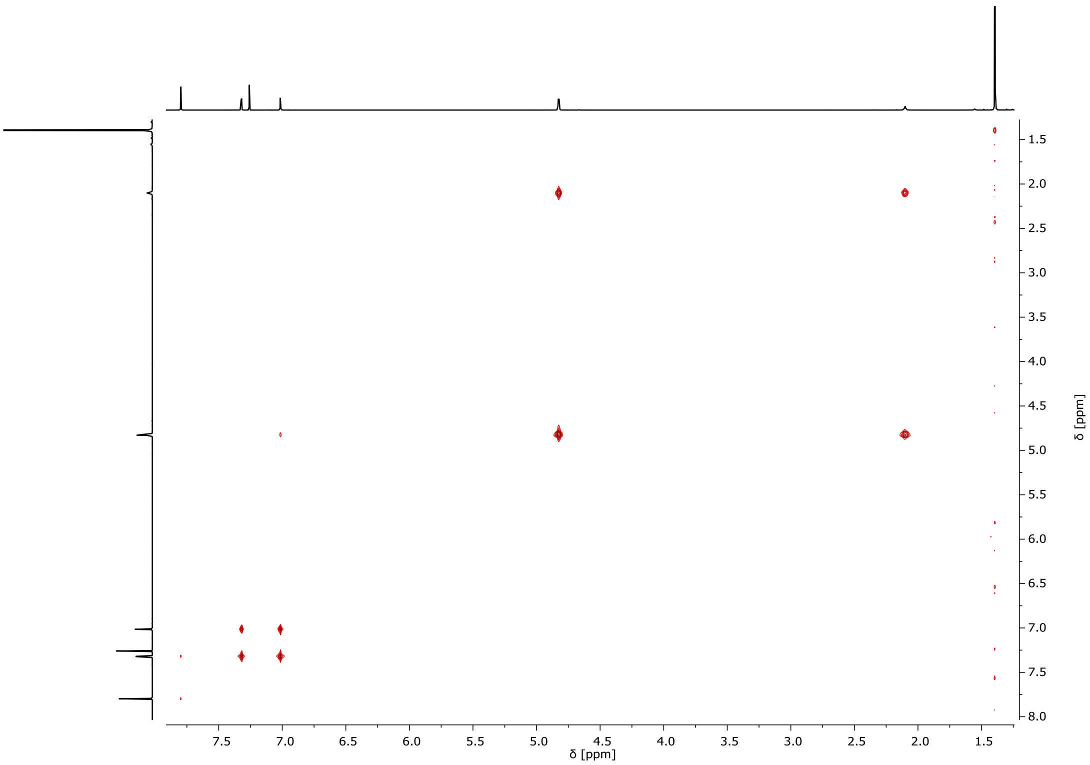

Figure S44 700 MHz ^{1}H-^{1}H COSY spectrum of **29** in $CDCl_3$.

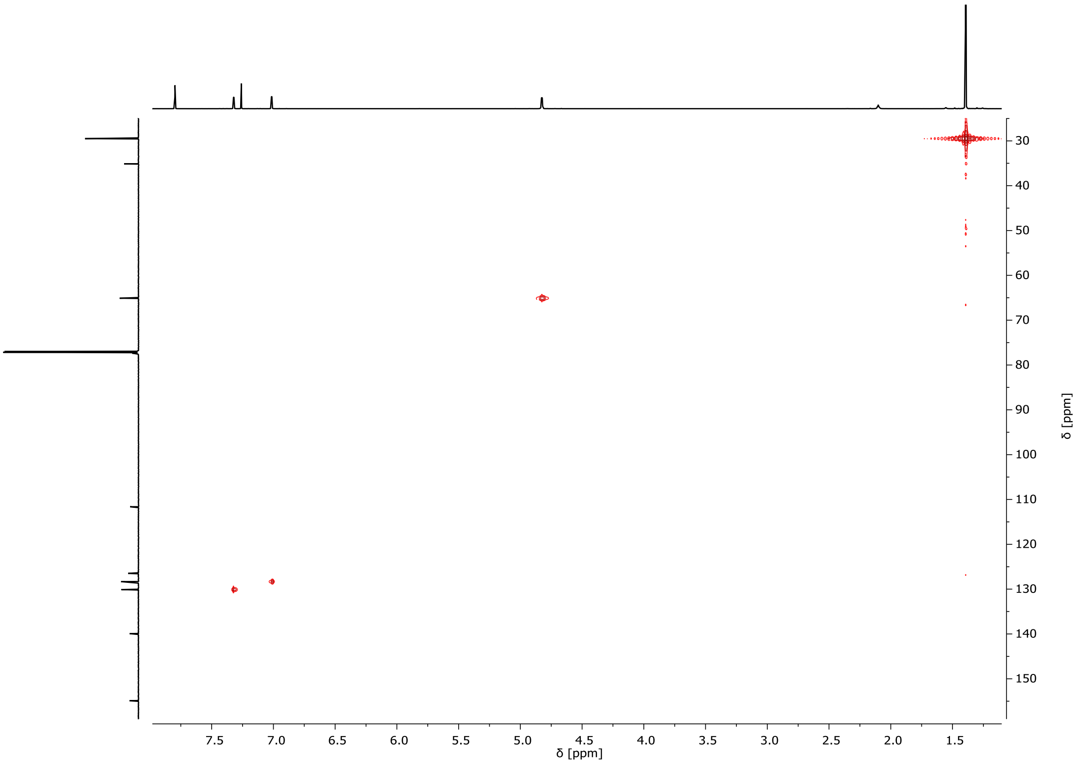

Figure S45 700 MHz ^{1}H-^{13}C Heteronuclear Single Quantum Coherence (HSQC) spectrum of **29** in $CDCl_3$.

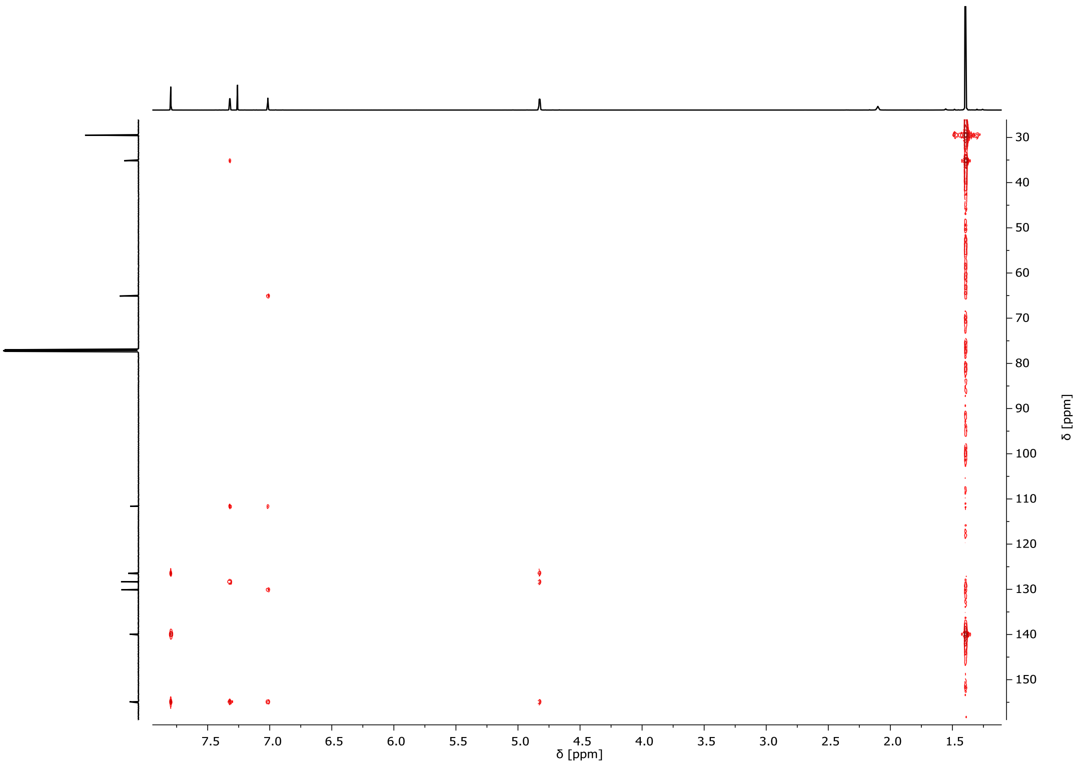

Figure S46 700 MHz ^{1}H-^{13}C Heteronuclear Multiple Bond Correlation (HMBC) spectrum of **29** in $CDCl_3$.

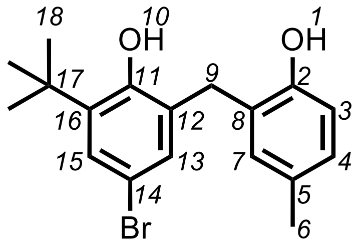

2

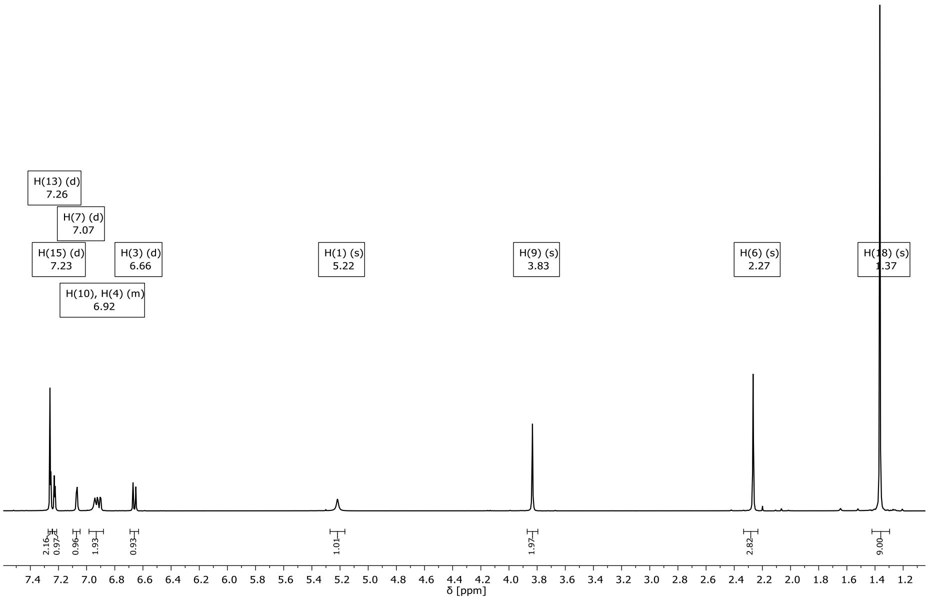

Figure S47 400 MHz ^{1}H-NMR of **2** in $CDCl_3$.

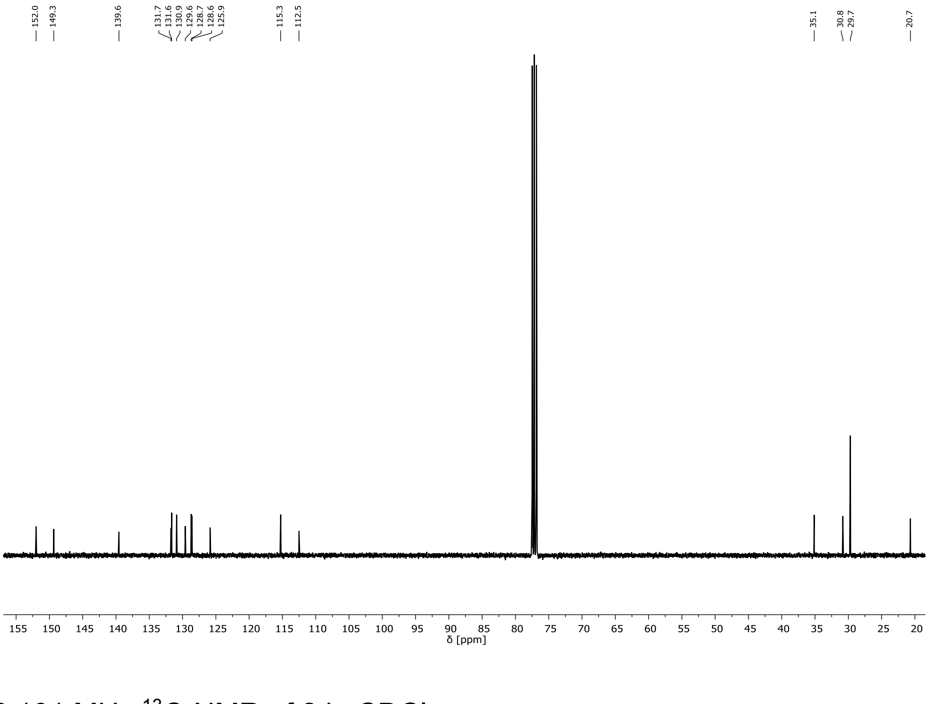

Figure S48 101 MHz ^{13}C-NMR of **2** in $CDCl_3$.

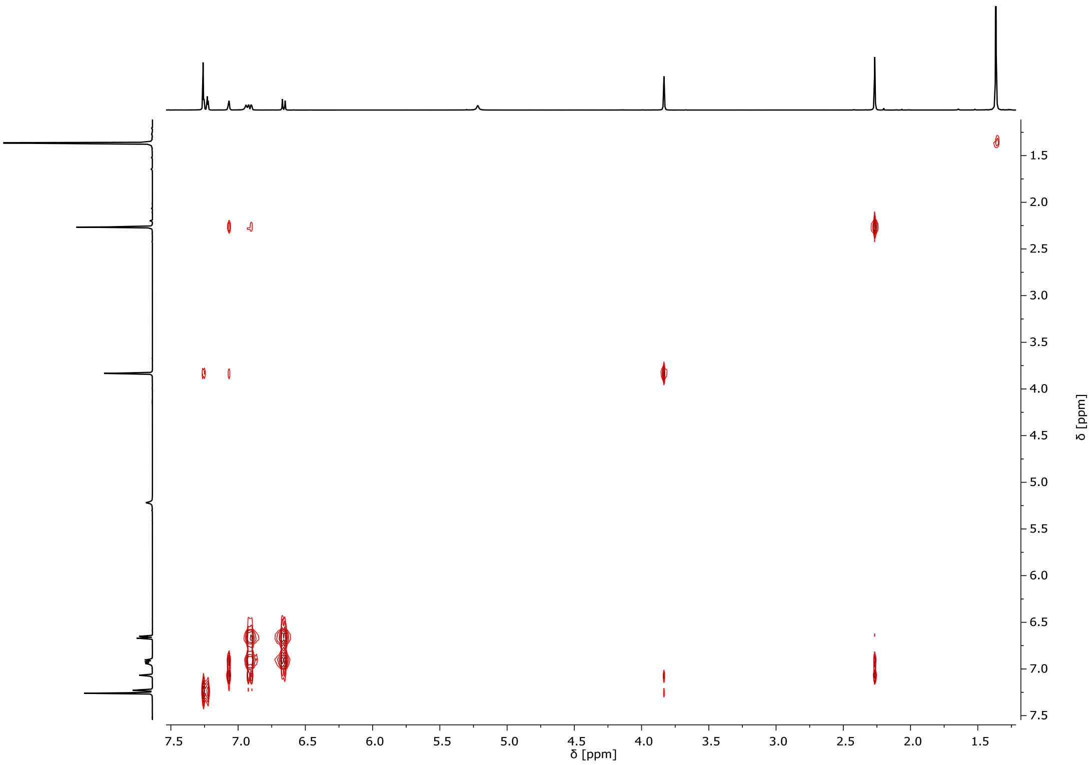

Figure S49 400 MHz ^{1}H-^{1}H COSY spectrum of **2** in $CDCl_3$.

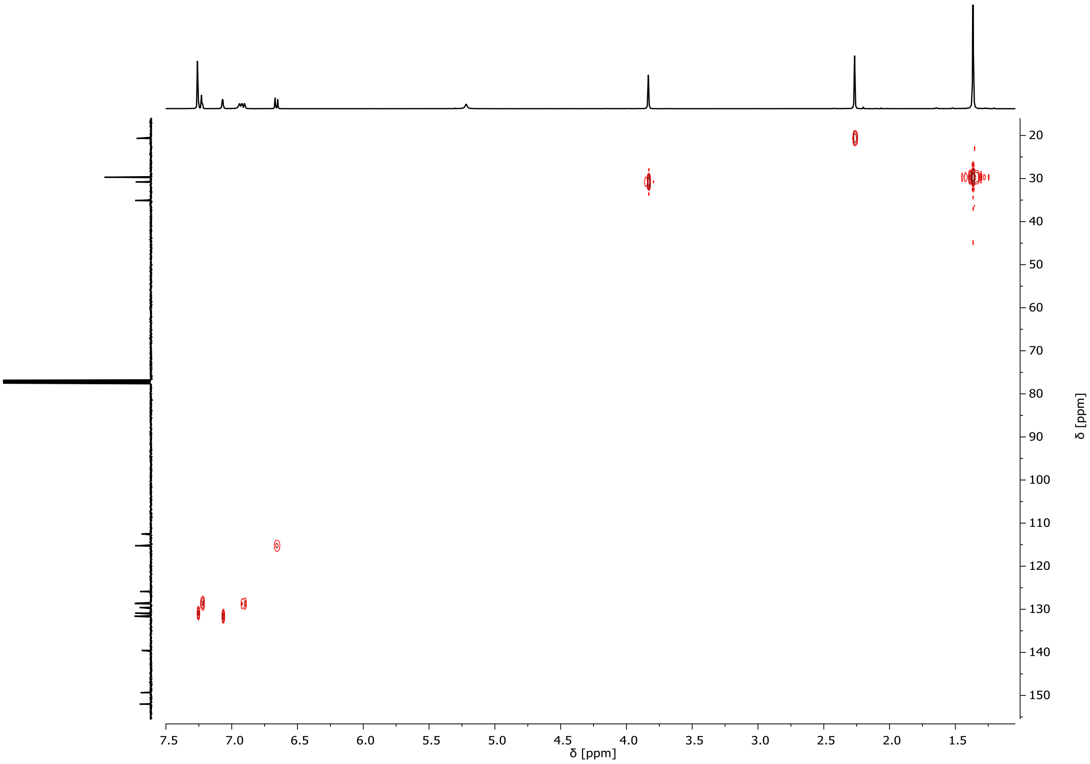

Figure S50 400 MHz ^{1}H-^{13}C Heteronuclear Single Quantum Coherence (HSQC) spectrum of **2** in $CDCl_3$.

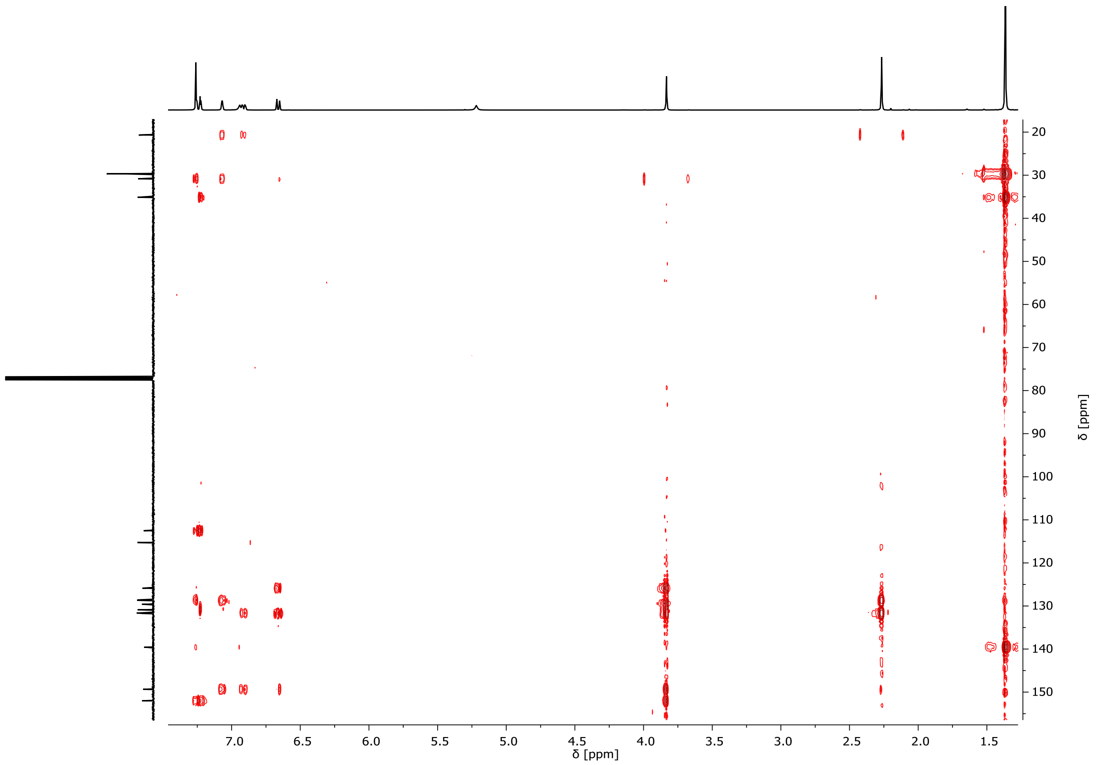

Figure S51 400 MHz ^{1}H-^{13}C Heteronuclear Multiple Bond Correlation (HMBC) spectrum of **2** in $CDCl_3$.

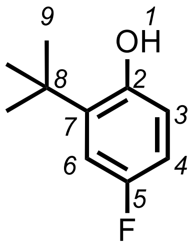

23

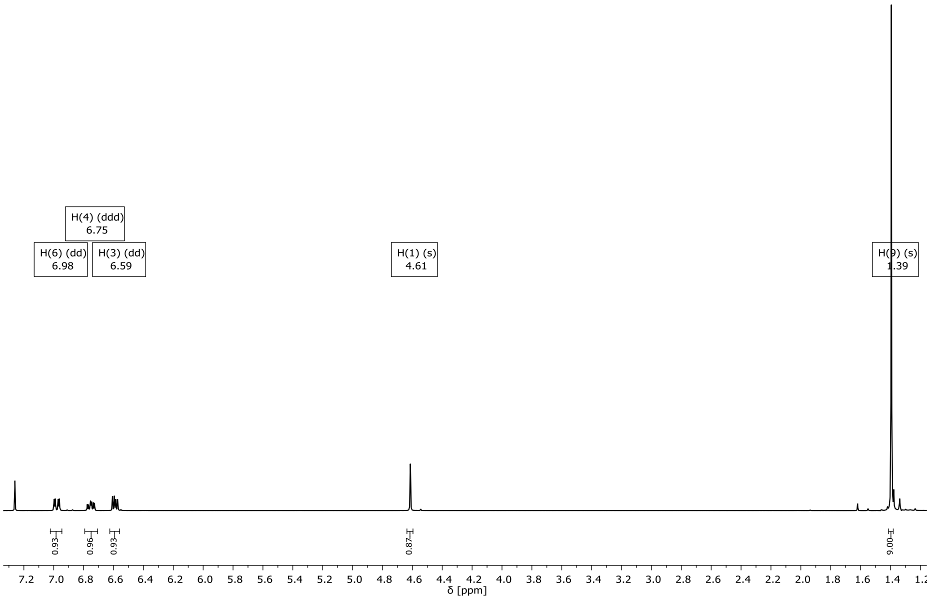

Figure S52 400 MHz ^{1}H-NMR of **23** in $CDCl_3$.

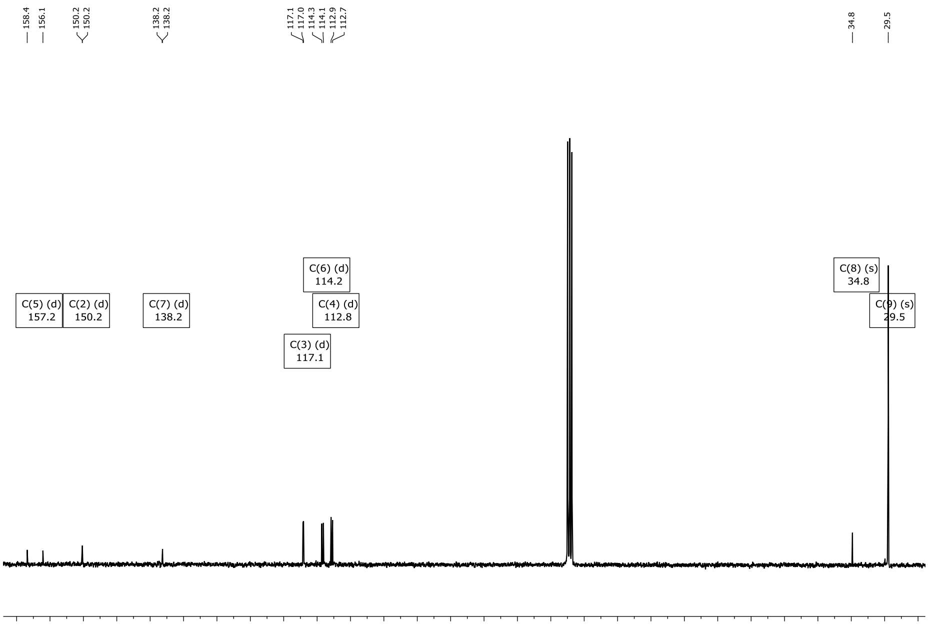

Figure S53 101 MHz ^{13}C-NMR of **23** in $CDCl_3$.

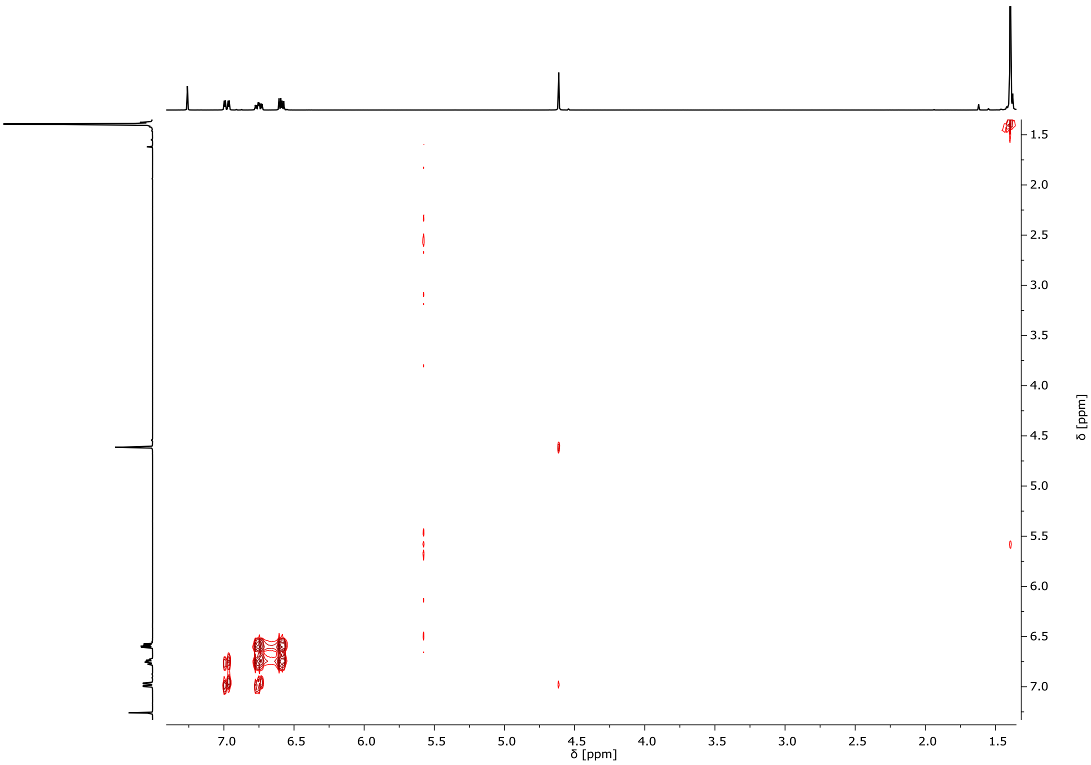

Figure S54 400 MHz ^{1}H-^{1}H COSY spectrum of **23** in $CDCl_3$.

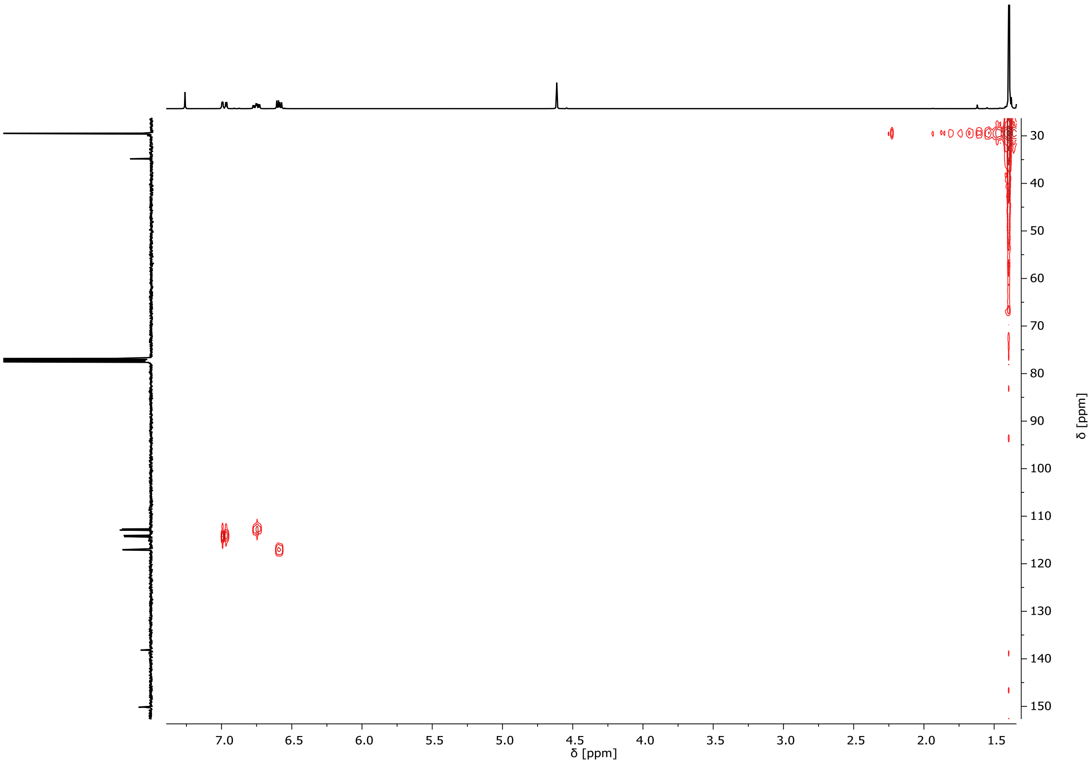

Figure S55 400 MHz ^{1}H-[13]C Heteronuclear Single Quantum Coherence (HSQC) spectrum of **23** in $CDCl_3$.

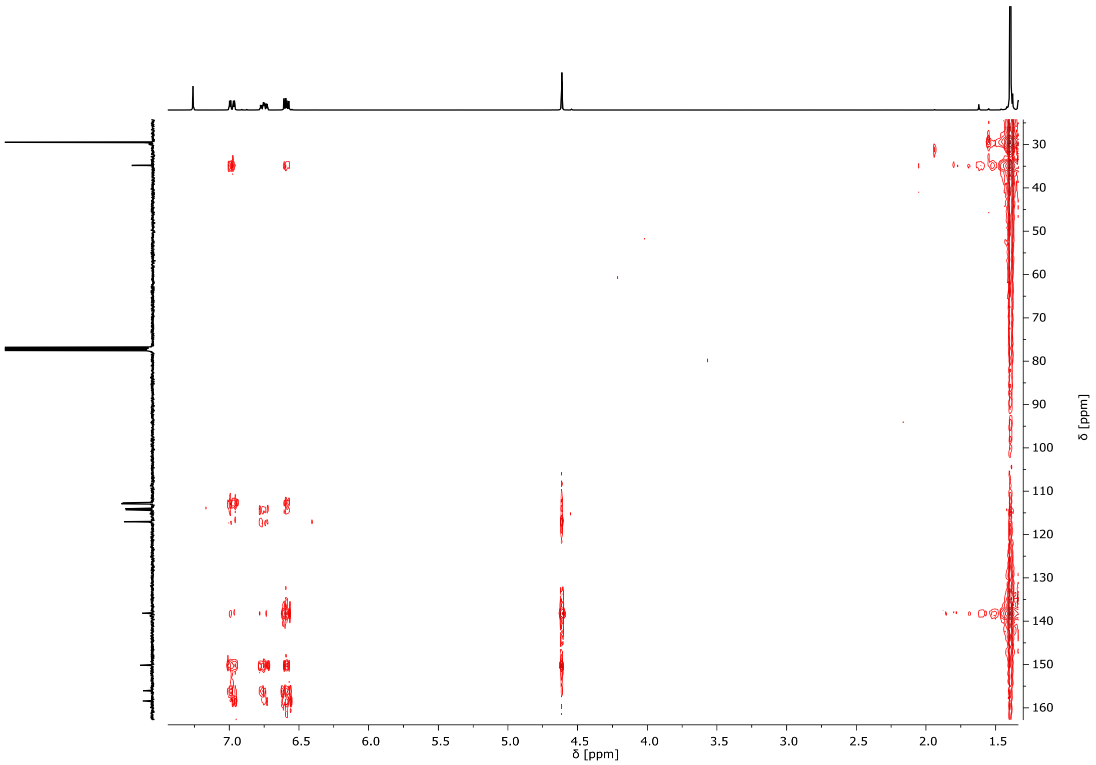

Figure S56 400 MHz ^{1}H-^{13}C Heteronuclear Multiple Bond Correlation (HMBC) spectrum of **23** in $CDCl_3$.

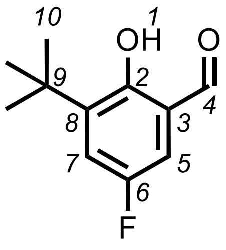

27

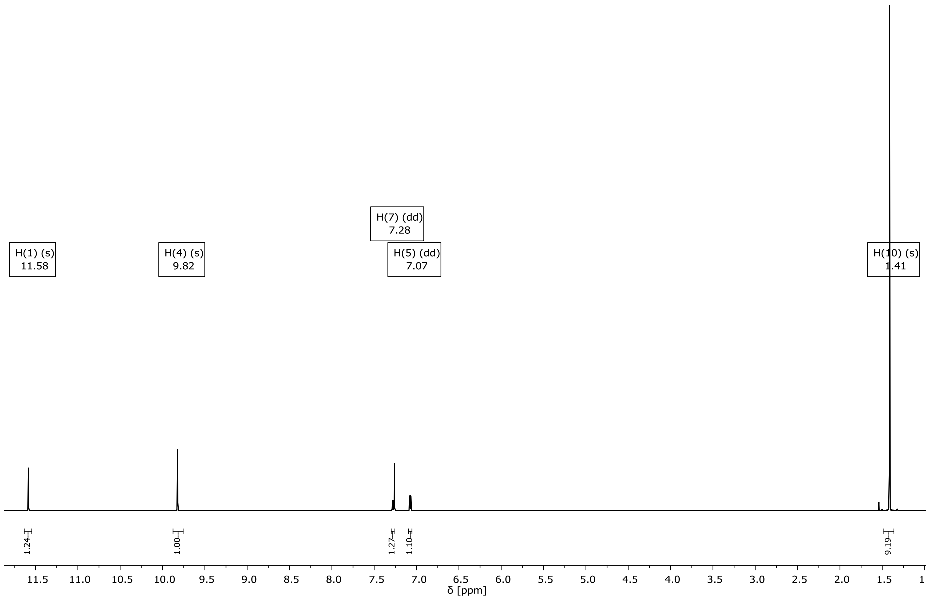

Figure S57 700 MHz ^{1}H-NMR of **27** in $CDCl_3$.

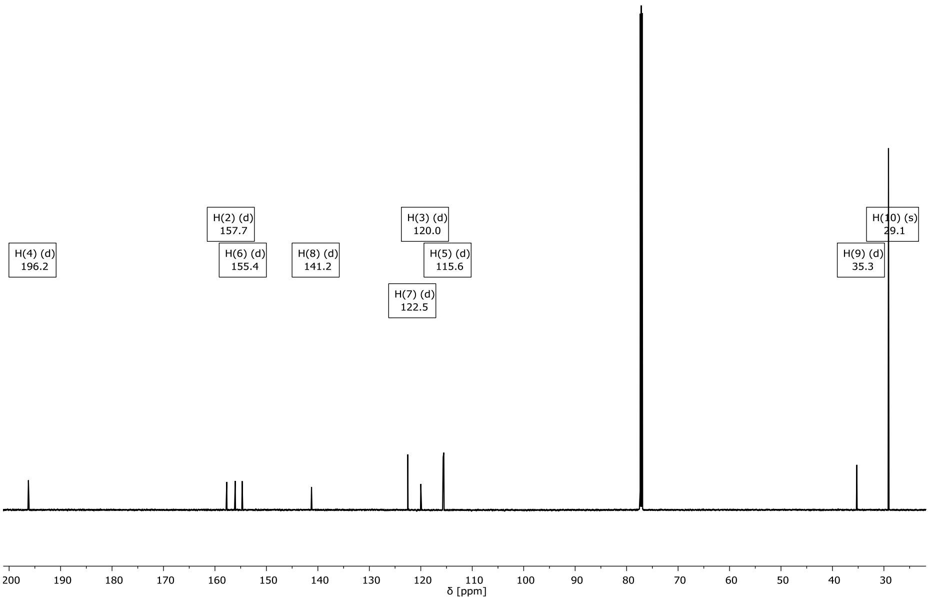

Figure S58 176 MHz ^{13}C-NMR of **27** in $CDCl_3$.

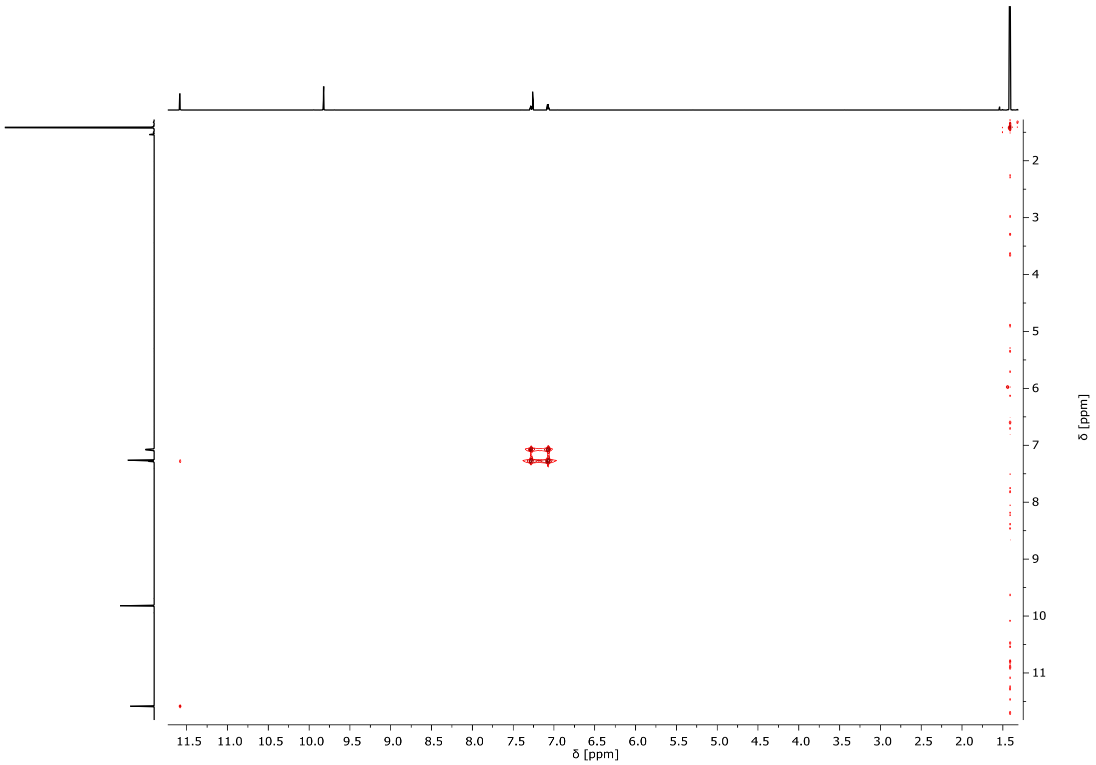

Figure S59 700 MHz ^{1}H-^{1}H COSY spectrum of **27** in $CDCl_3$.

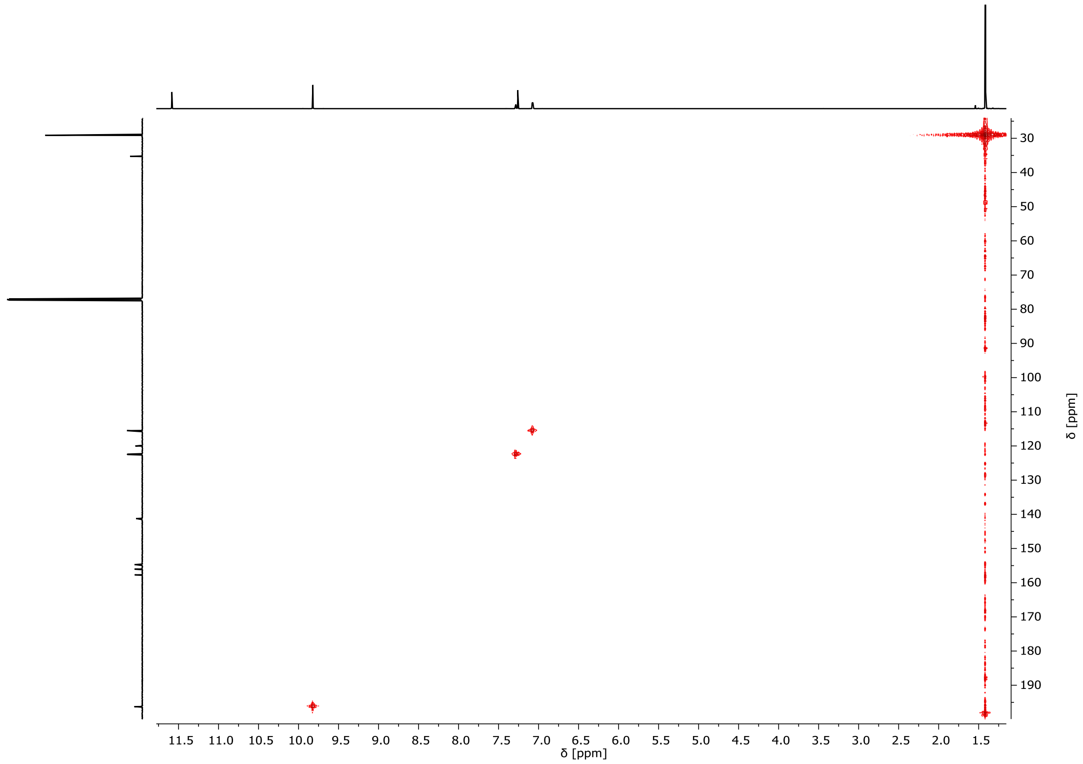

Figure S60 700 MHz ^{1}H-^{13}C Heteronuclear Single Quantum Coherence (HSQC) spectrum of **27** in $CDCl_3$.

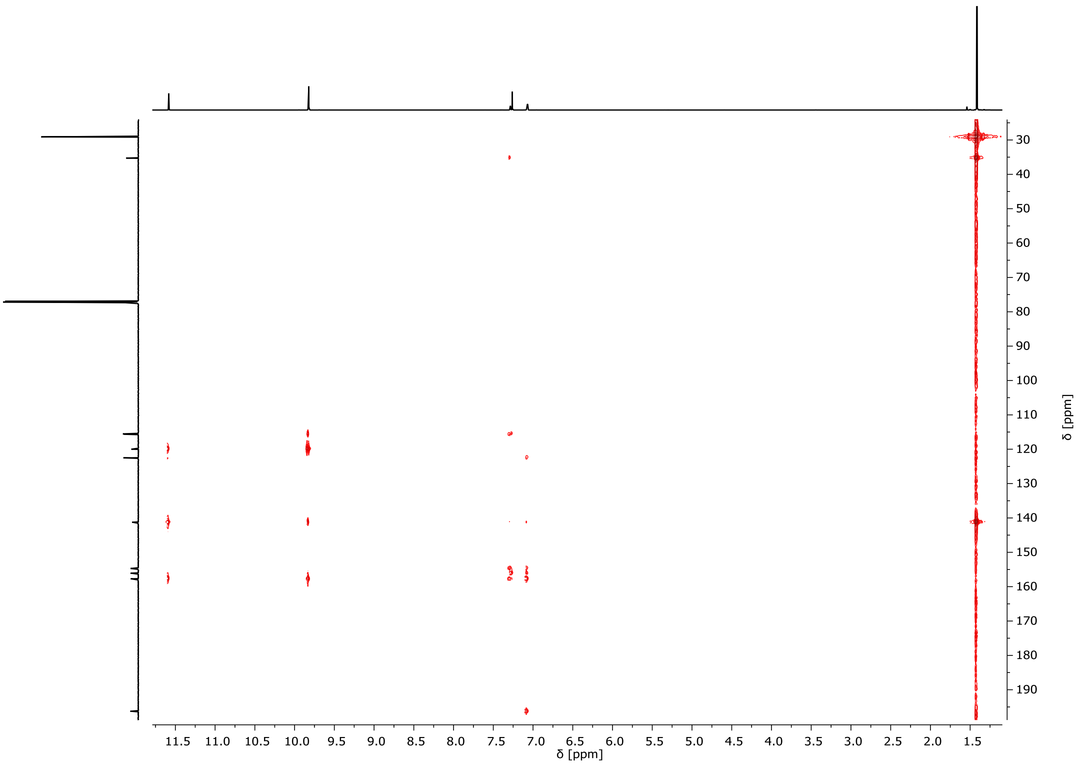

Figure S61 700 MHz ^{1}H-^{13}C Heteronuclear Multiple Bond Correlation (HMBC) spectrum of **27** in $CDCl_3$.

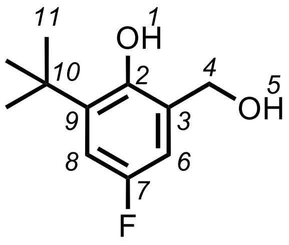

30

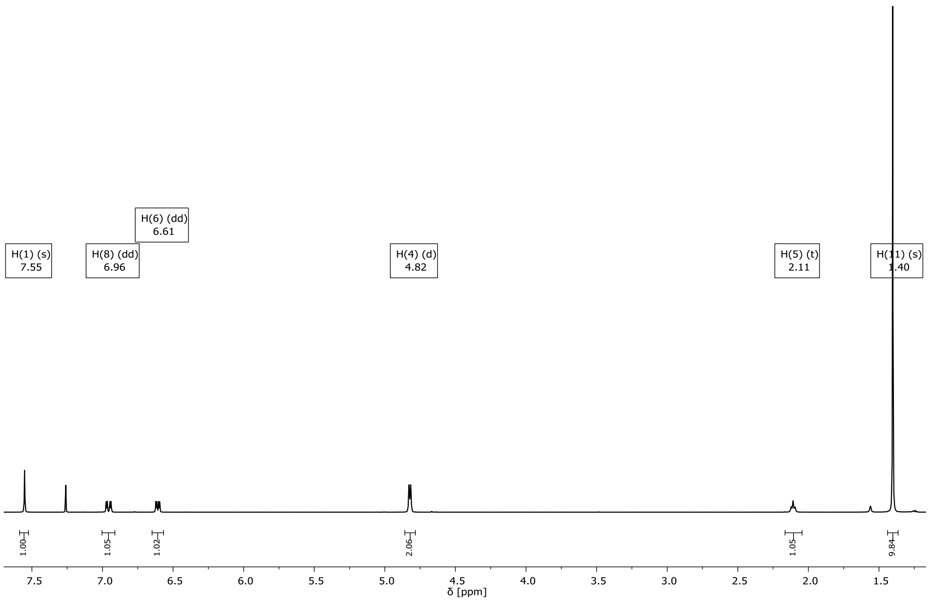

Figure S62 400 MHz ^{1}H-NMR of **30** in $CDCl_3$.

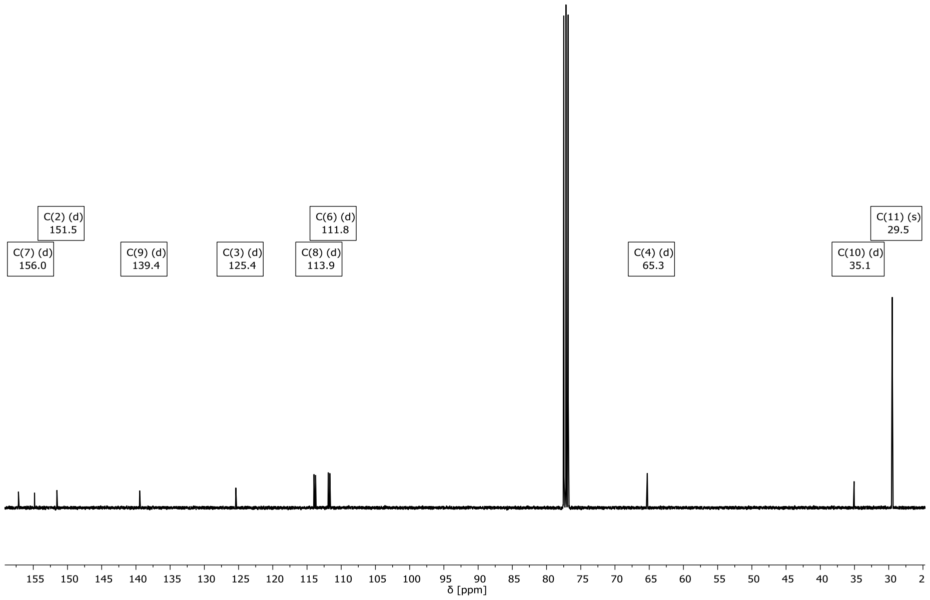

Figure S63 101 MHz ^{13}C-NMR of **30** in $CDCl_3$.

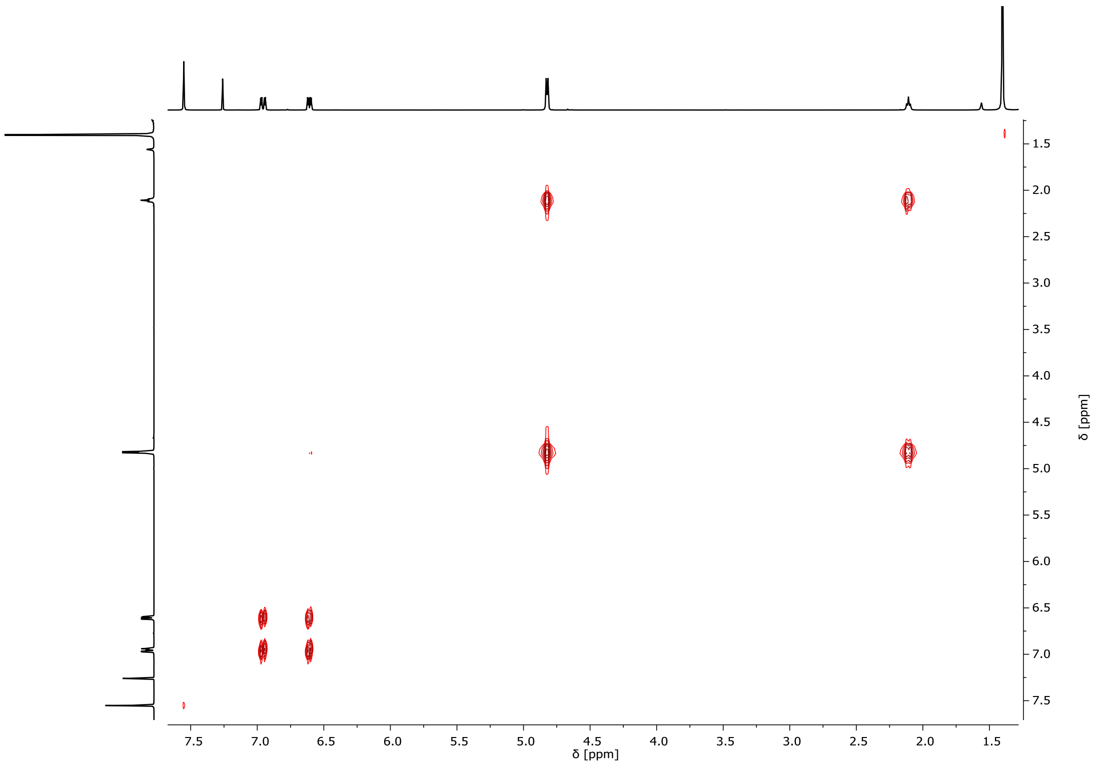

Figure S64 400 MHz ^{1}H-^{1}H COSY spectrum of **30** in $CDCl_3$.

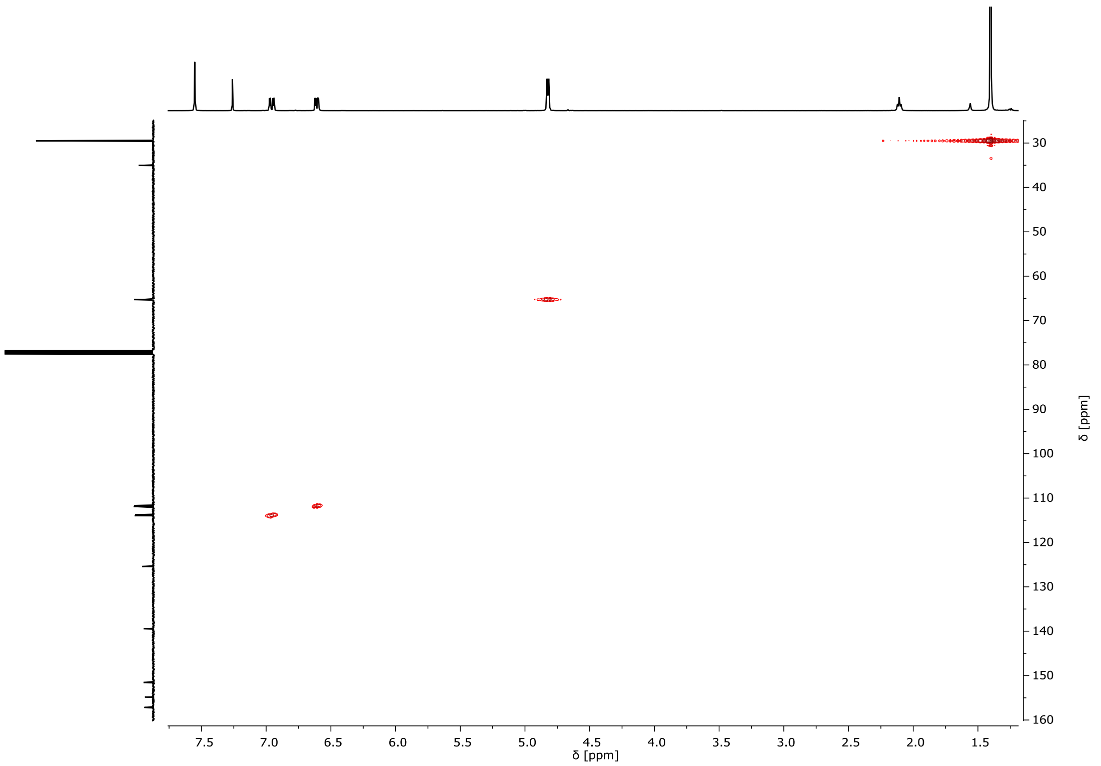

Figure S65 400 MHz ^{1}H-^{13}C Heteronuclear Single Quantum Coherence (HSQC) spectrum of **30** in $CDCl_3$.

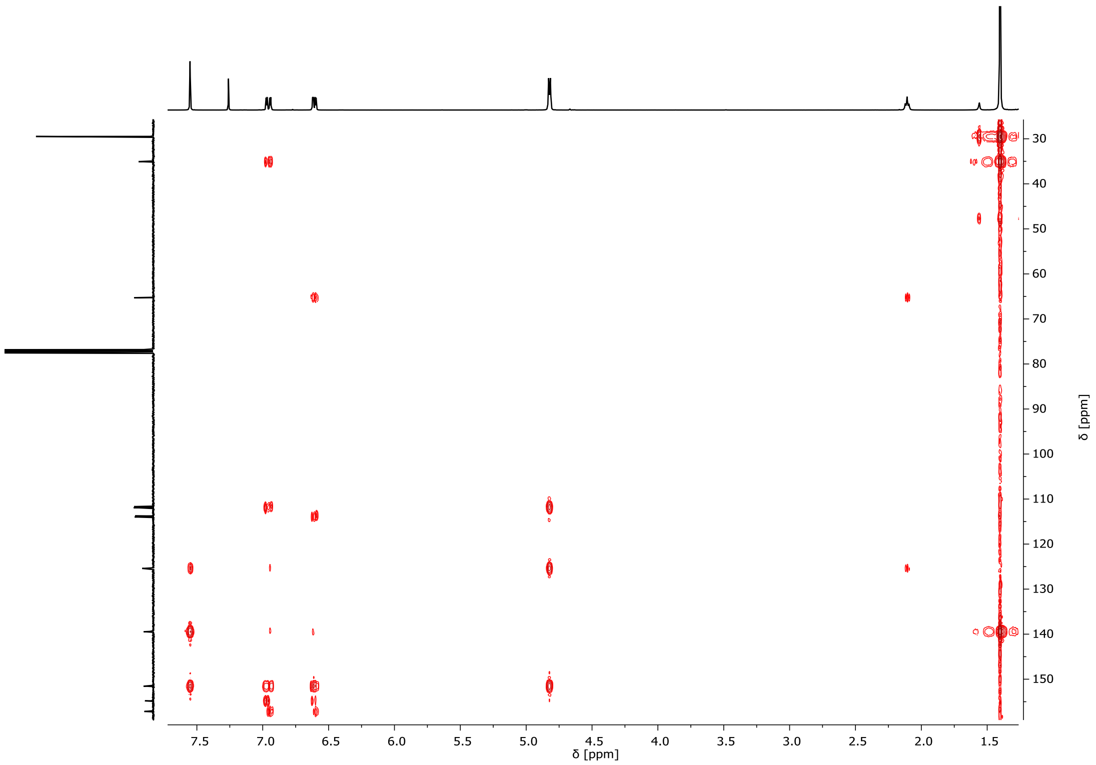

Figure S66 400 MHz ^{1}H-^{13}C Heteronuclear Multiple Bond Correlation (HMBC) spectrum of **30** in $CDCl_3$.

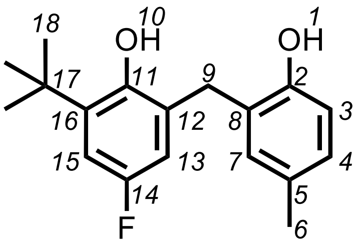

3

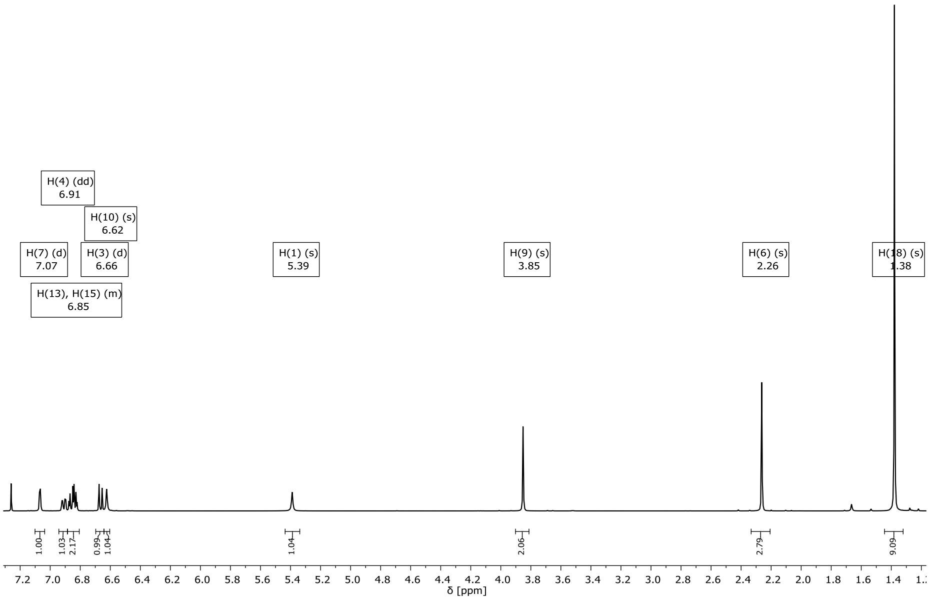

Figure S67 400 MHz ^{1}H-NMR of **3** in $CDCl_3$.

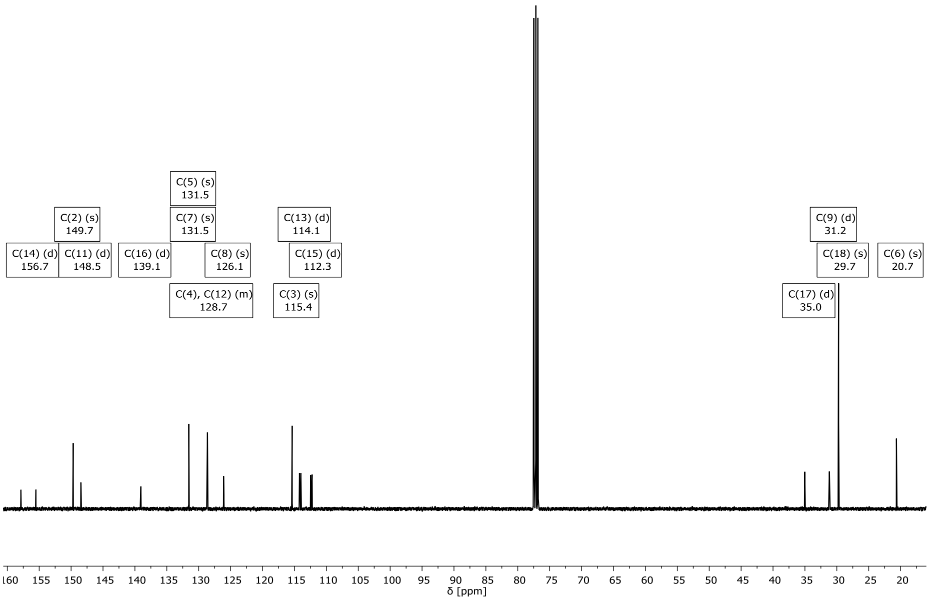

Figure S68 101 MHz ^{13}C-NMR of **3** in $CDCl_3$.

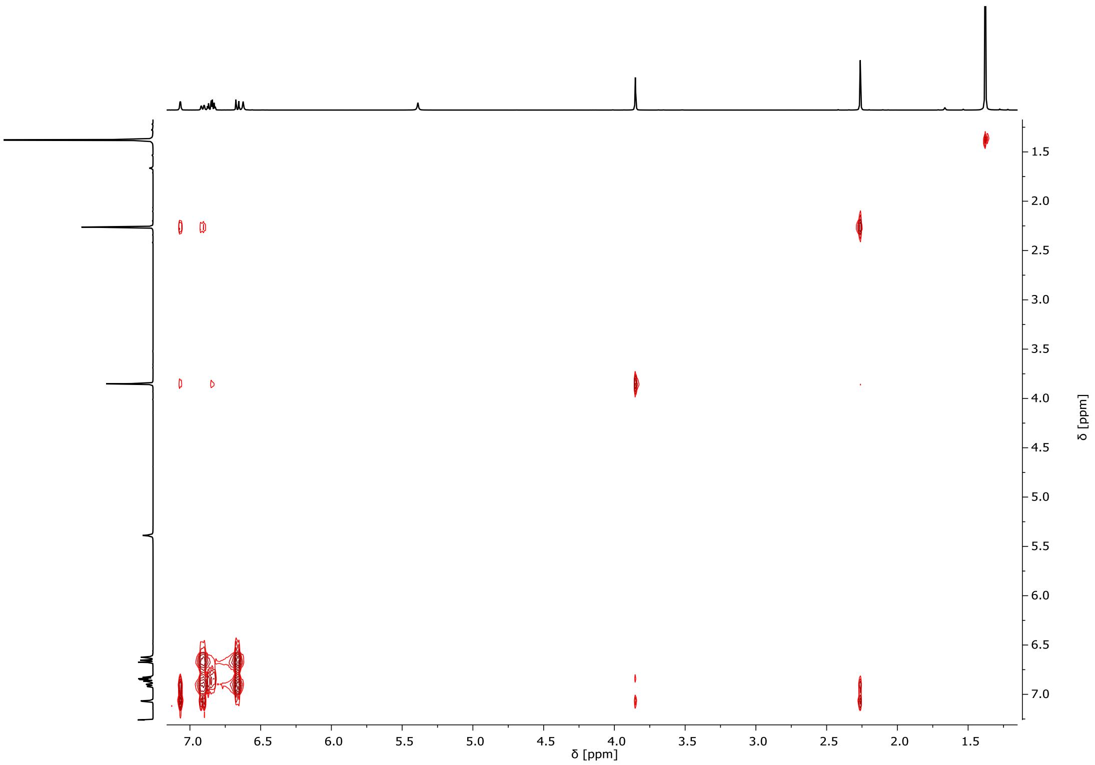

Figure S69 400 MHz ^{1}H-^{1}H COSY spectrum of **3** in $CDCl_3$.

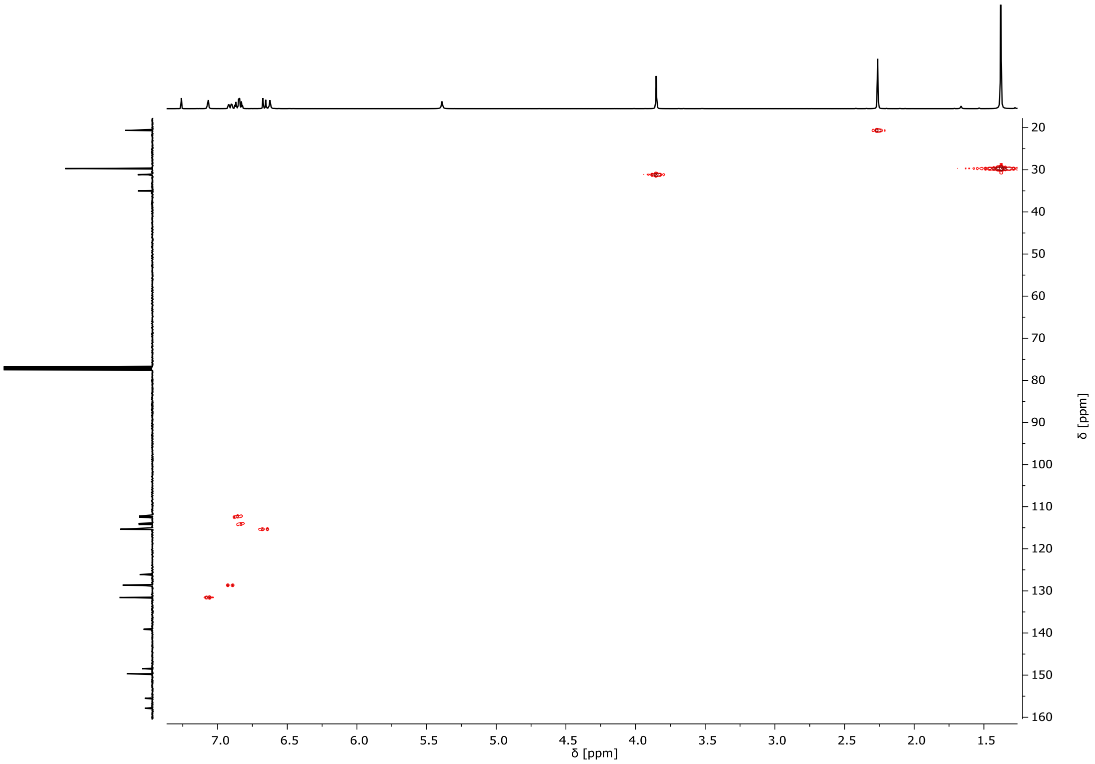

Figure S70 400 MHz ^{1}H-^{13}C Heteronuclear Single Quantum Coherence (HSQC) spectrum of **3** in $CDCl_3$.

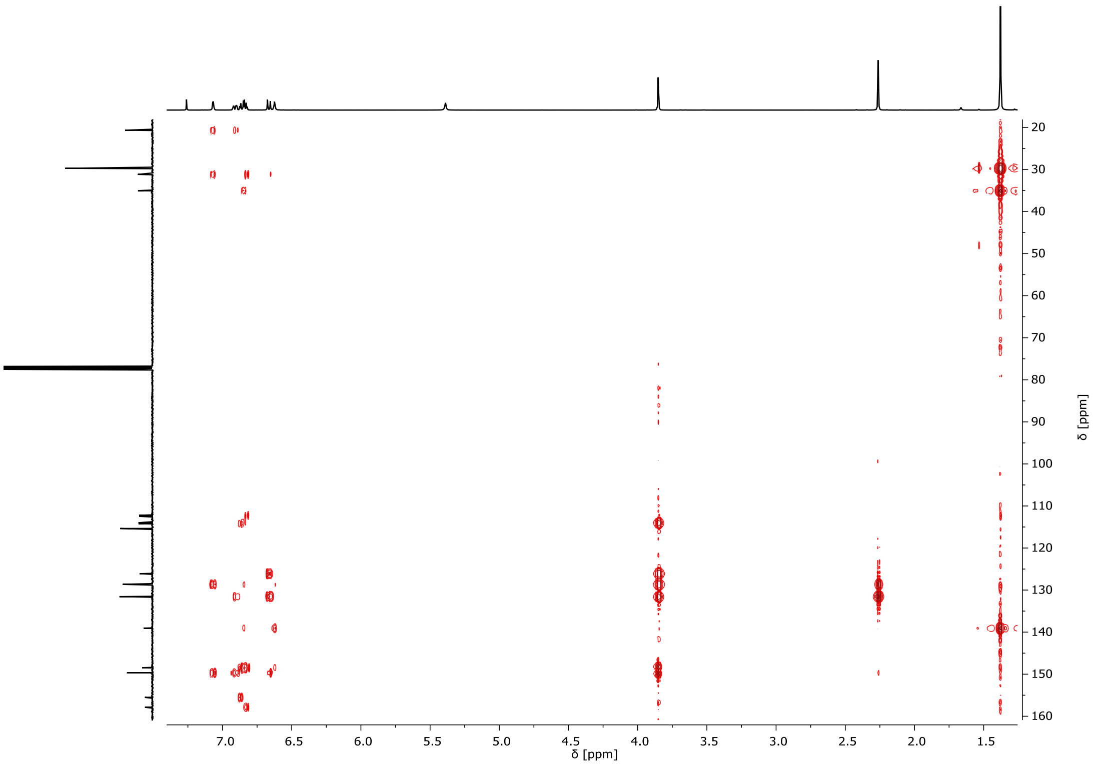

Figure S71 400 MHz ^{1}H-[13]C Heteronuclear Multiple Bond Correlation (HMBC) spectrum of **3** in $CDCl_3$.

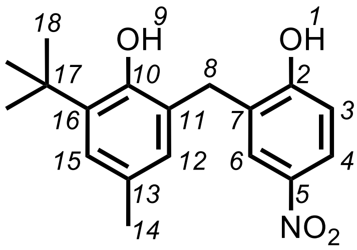

6

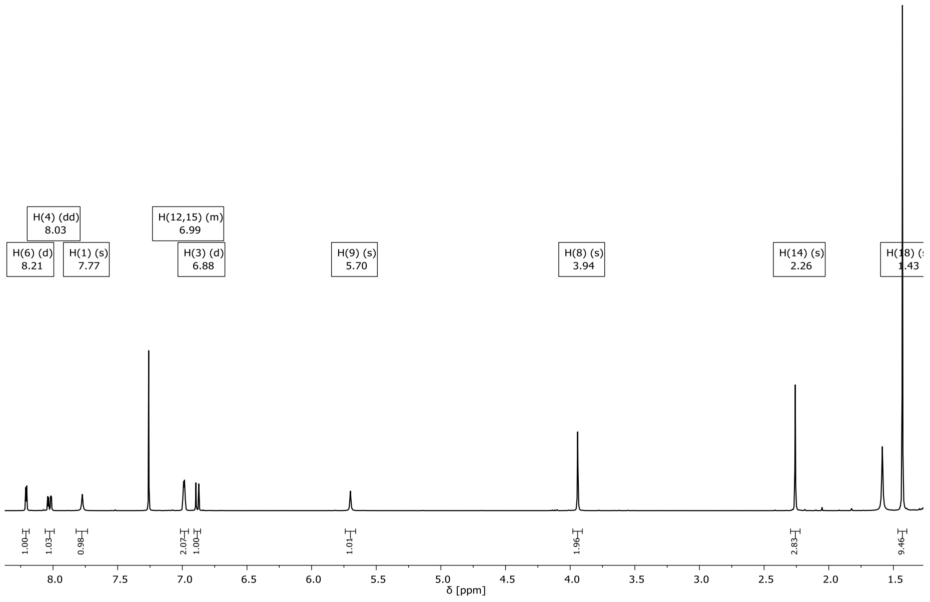

Figure S72 400 MHz ^{1}H-NMR of **6** in $CDCl_3$.

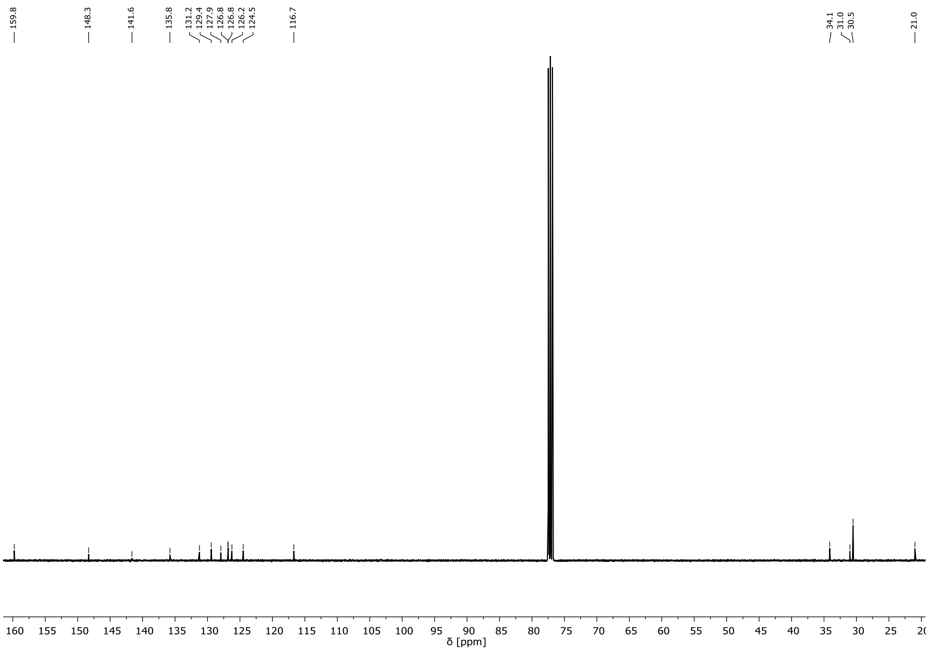

Figure S73 101 MHz ^{13}C-NMR of **6** in $CDCl_3$.

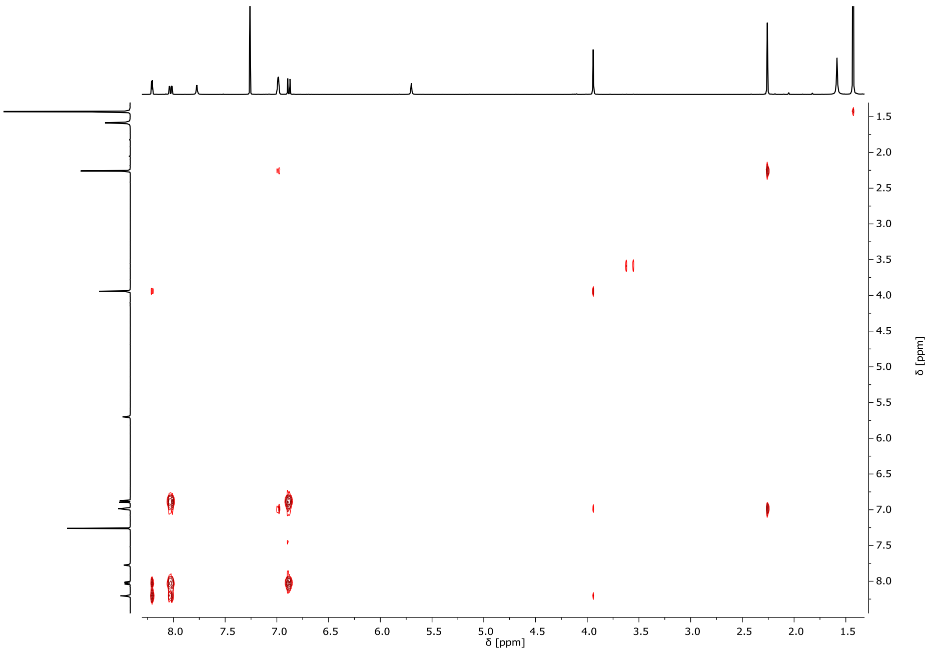

Figure S74 400 MHz ^{1}H-^{1}H COSY spectrum of **6** in $CDCl_3$.

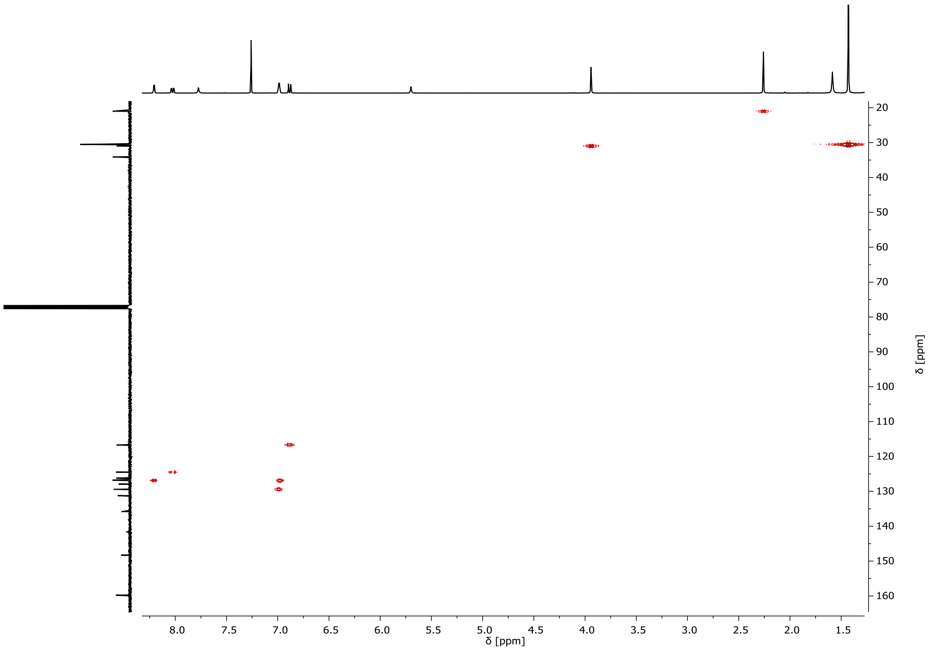

Figure S75 400 MHz ^{1}H-^{13}C Heteronuclear Single Quantum Coherence (HSQC) spectrum of **6** in $CDCl_3$.

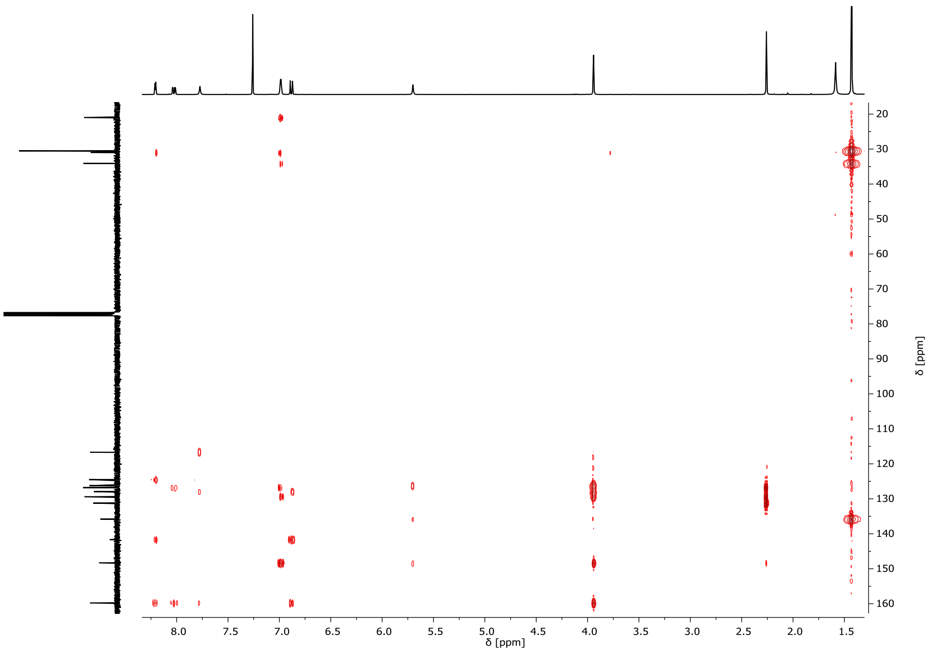

Figure S76 400 MHz ^{1}H-^{13}C Heteronuclear Multiple Bond Correlation (HMBC) spectrum of **6** in $CDCl_3$.

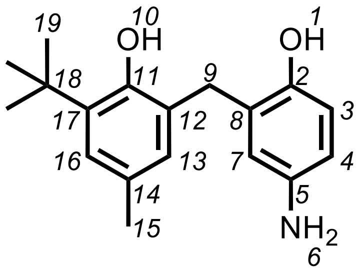

38

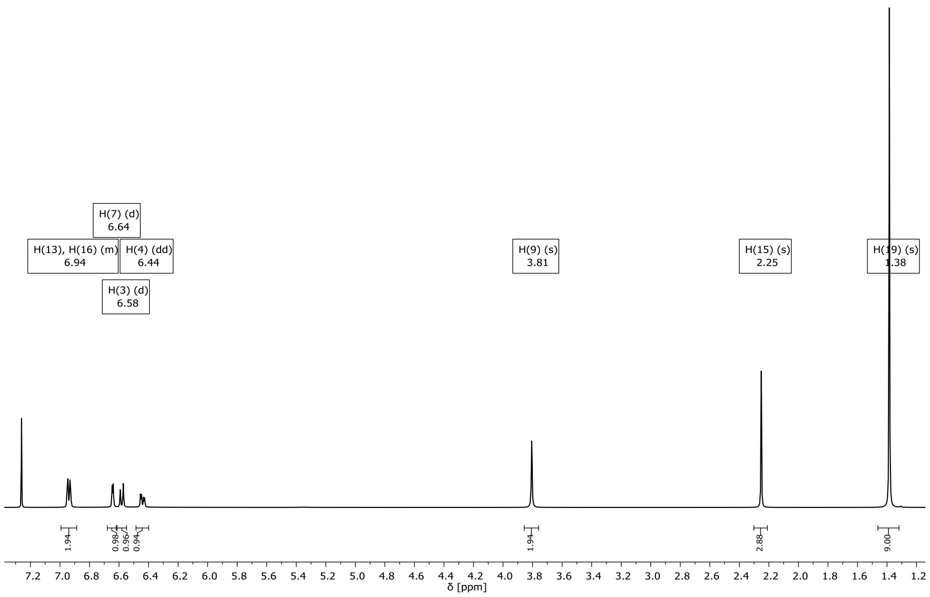

Figure S77 400 MHz ^{1}H-NMR of **38** in $CDCl_3$.

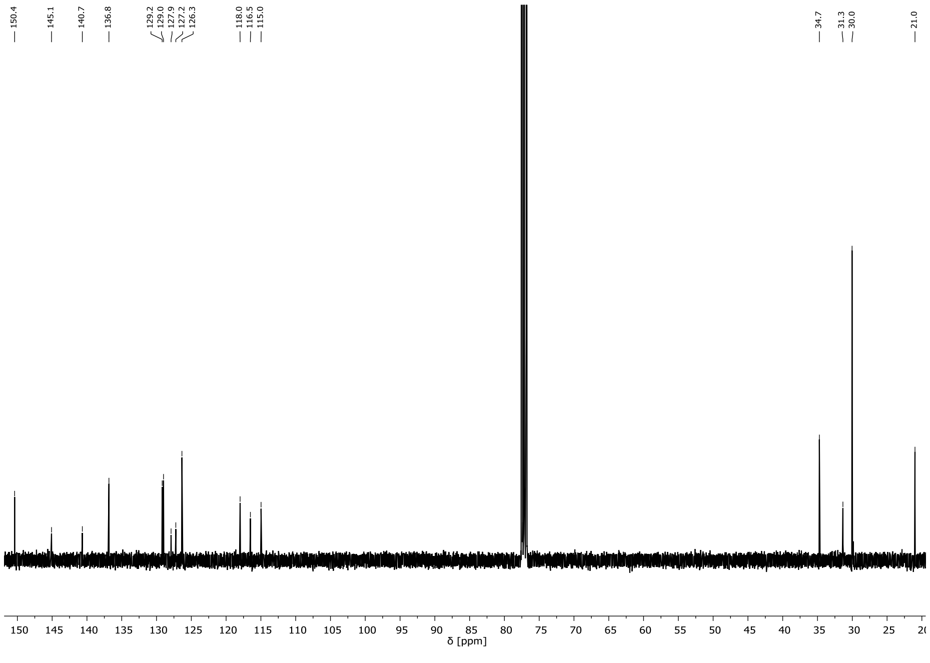

Figure S78 101 MHz ^{13}C-NMR of **38** in $CDCl_3$.

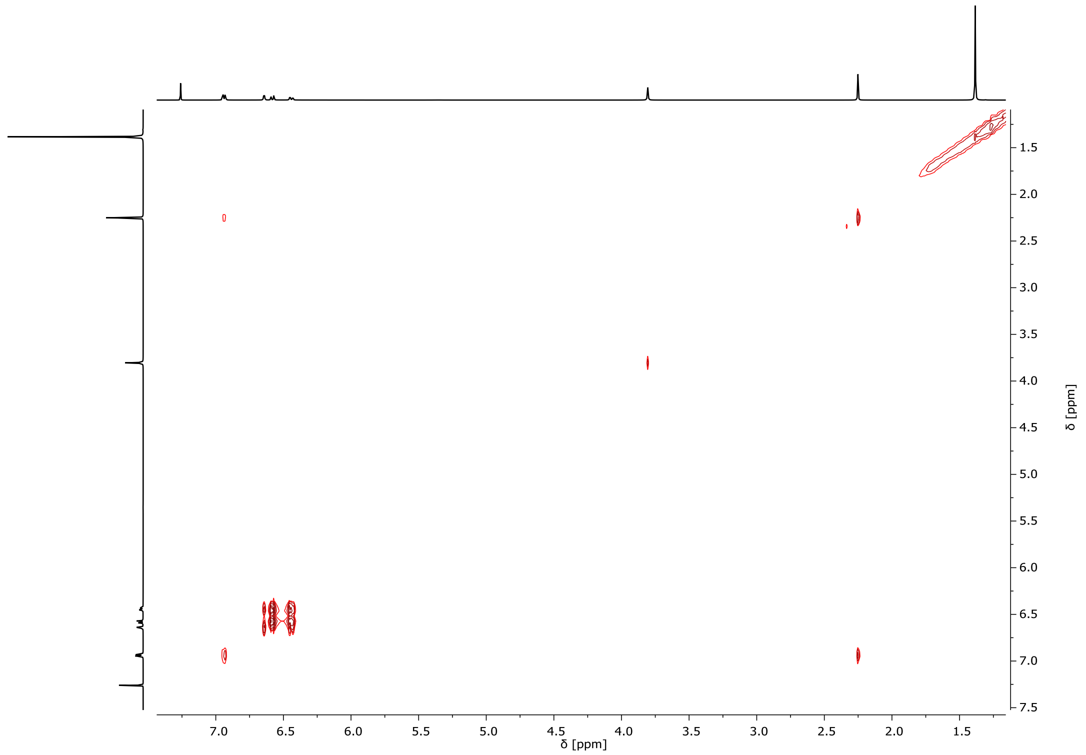

Figure S79 400 MHz ^{1}H-^{1}H COSY spectrum of **38** in $CDCl_3$.

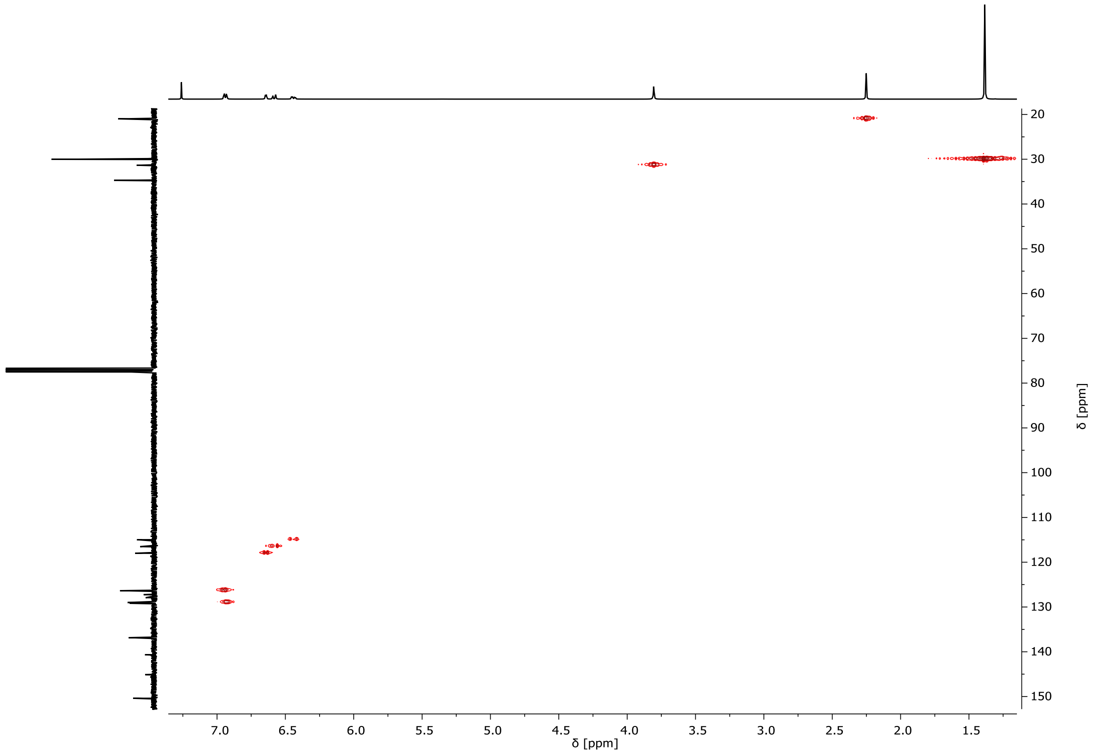

Figure S80 400 MHz ^{1}H-^{13}C Heteronuclear Single Quantum Coherence (HSQC) spectrum of **38** in $CDCl_3$.

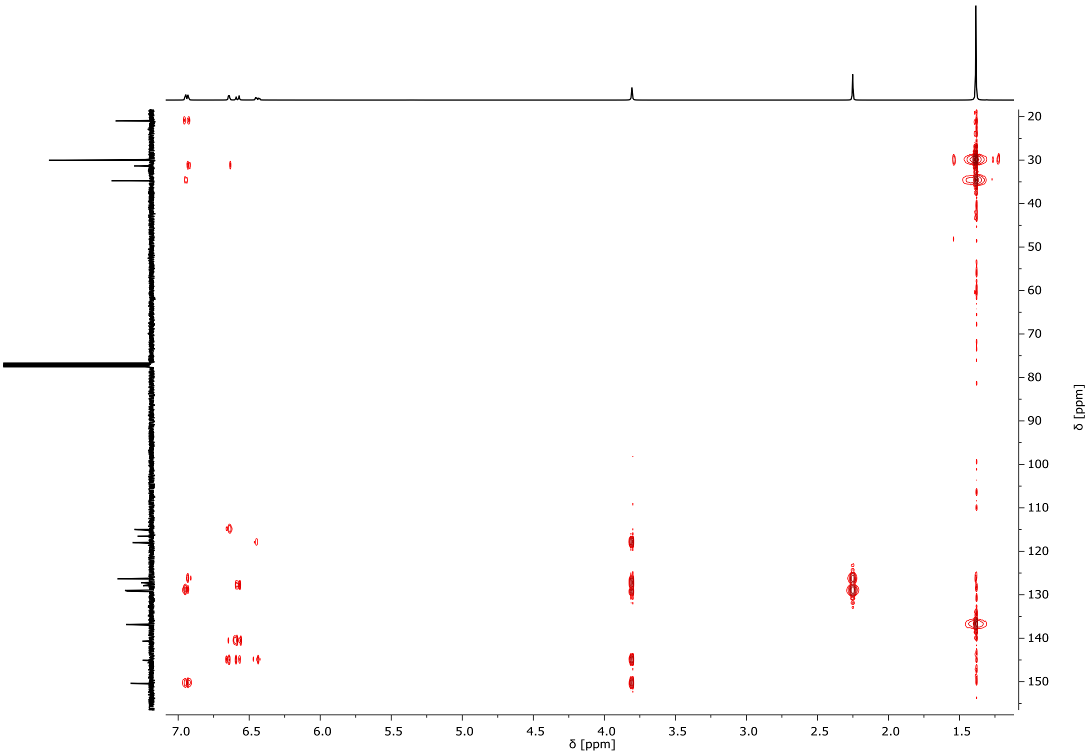

Figure S81 400 MHz ^{1}H-^{13}C Heteronuclear Multiple Bond Correlation (HMBC) spectrum of **38** in $CDCl_3$.

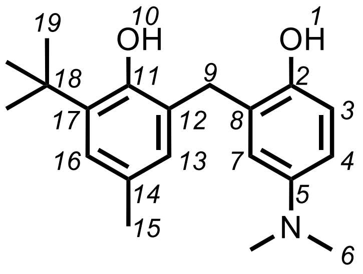

9

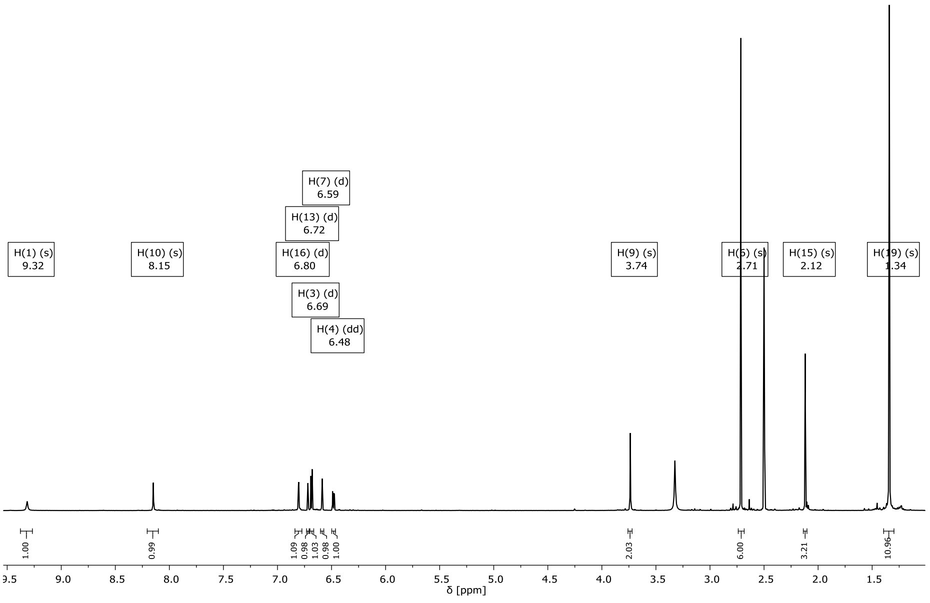

Figure S82 700 MHz ^{1}H-NMR of **9** in d_6-DMSO.

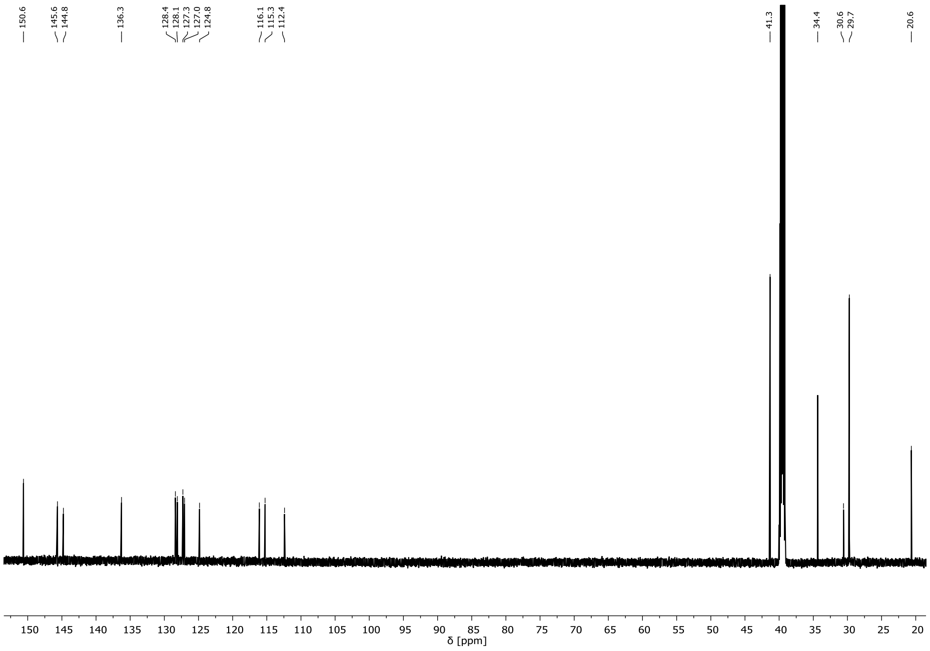

Figure S83 176 MHz ^{13}C-NMR of **9** in d_6-DMSO.

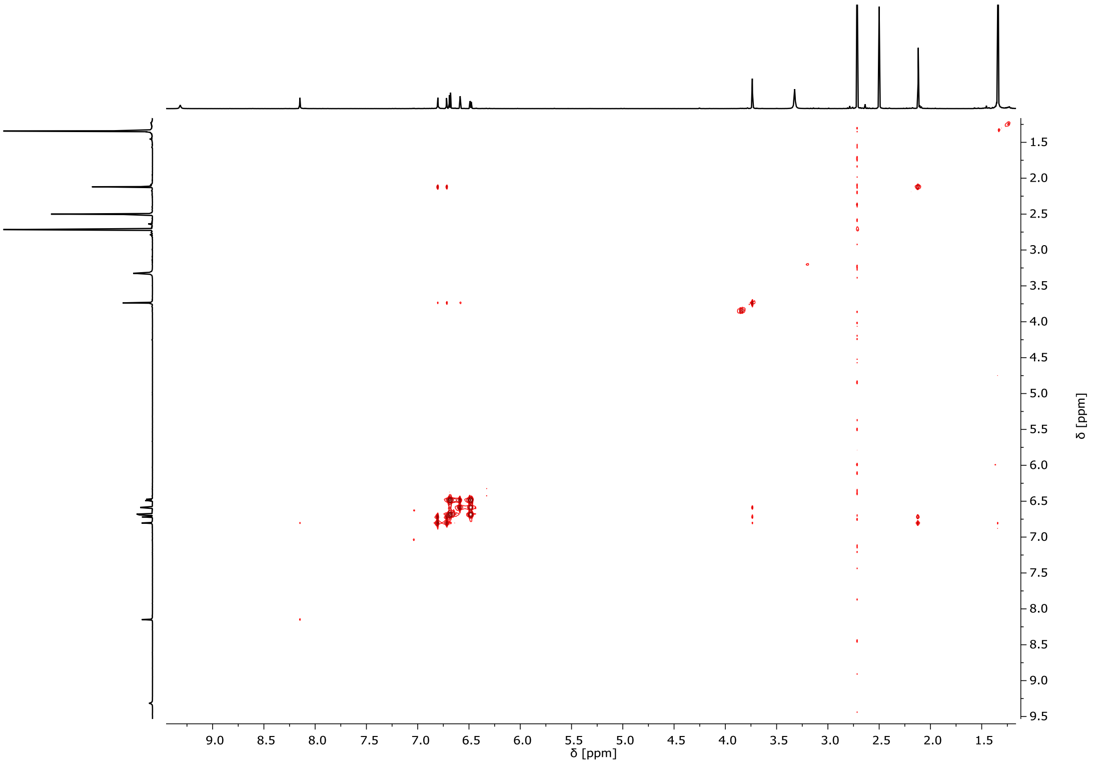

Figure S84 700 MHz ^{1}H-^{1}H COSY spectrum of **9** in d_6-DMSO.

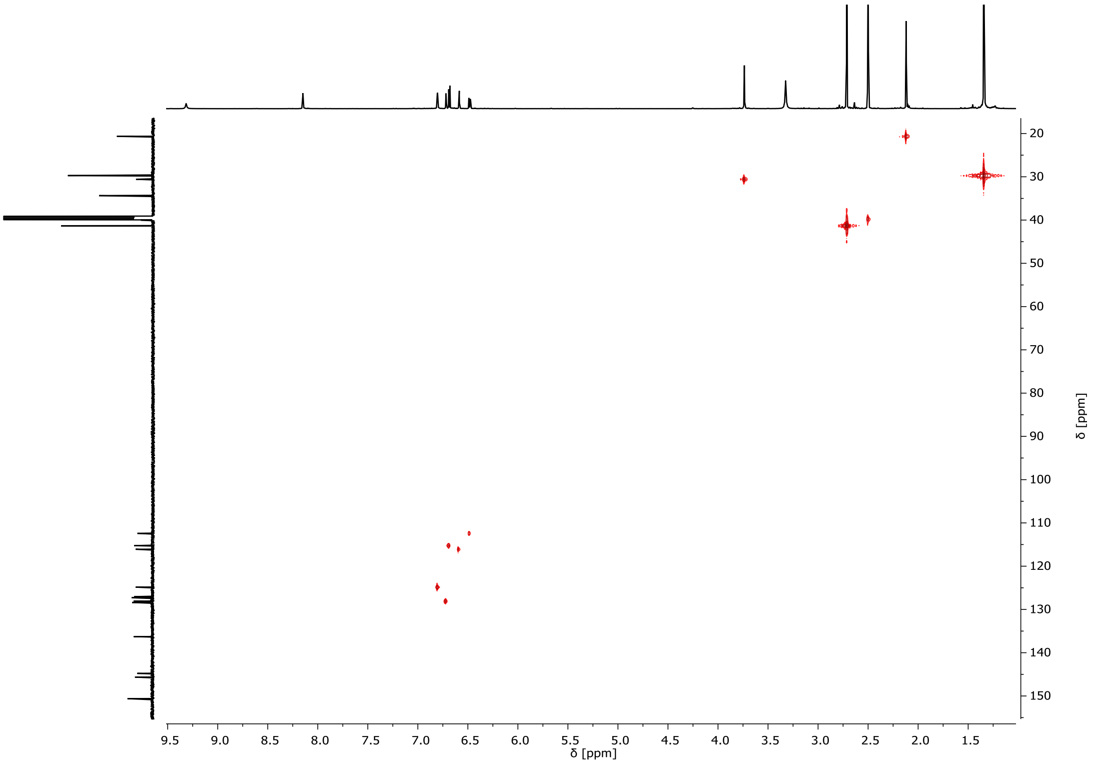

Figure S85 700 MHz ^{1}H-^{13}C Heteronuclear Single Quantum Coherence (HSQC) spectrum of **9** in d_6-DMSO.

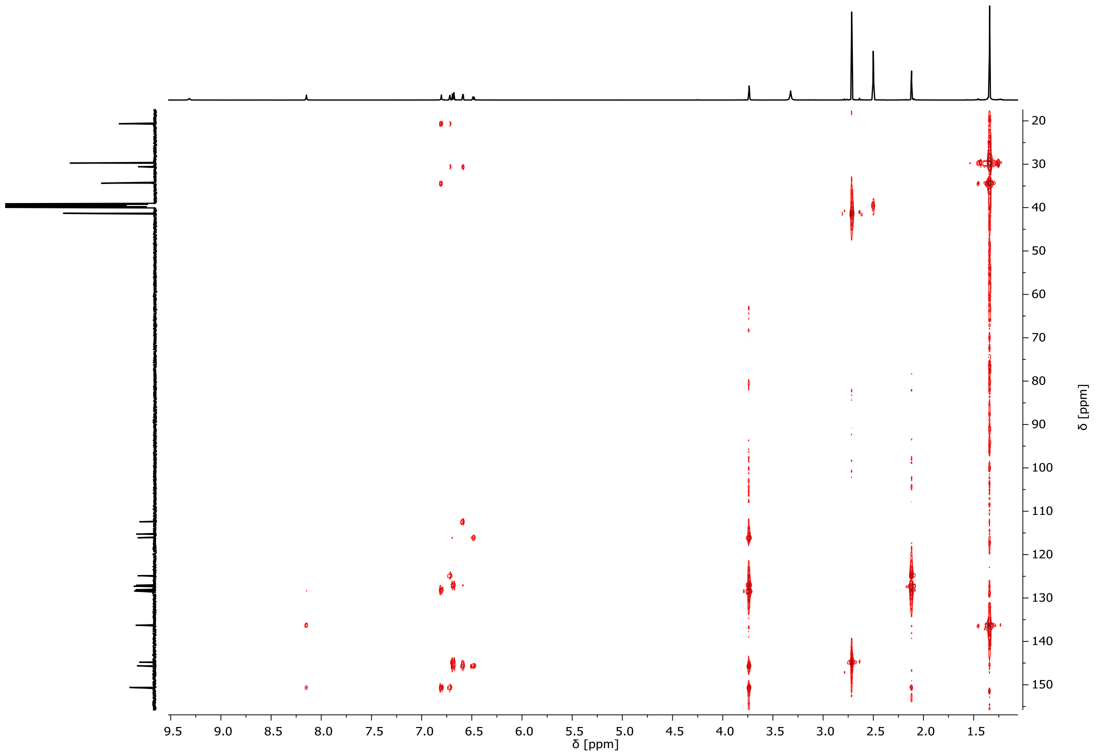

Figure S86 700 MHz ^{1}H-^{13}C Heteronuclear Multiple Bond Correlation (HMBC) spectrum of **9** in d_6-DMSO.

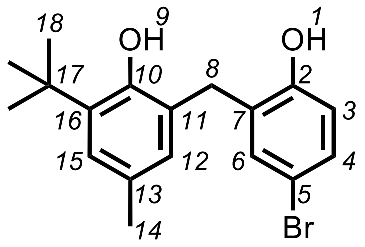

7

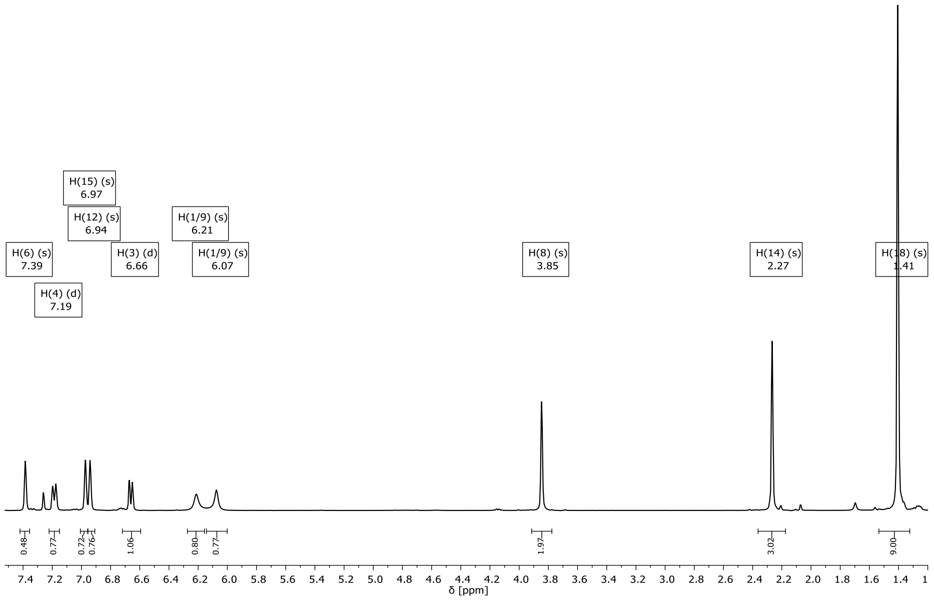

Figure S87 400 MHz ^{1}H-NMR of **7** in $CDCl_3$.

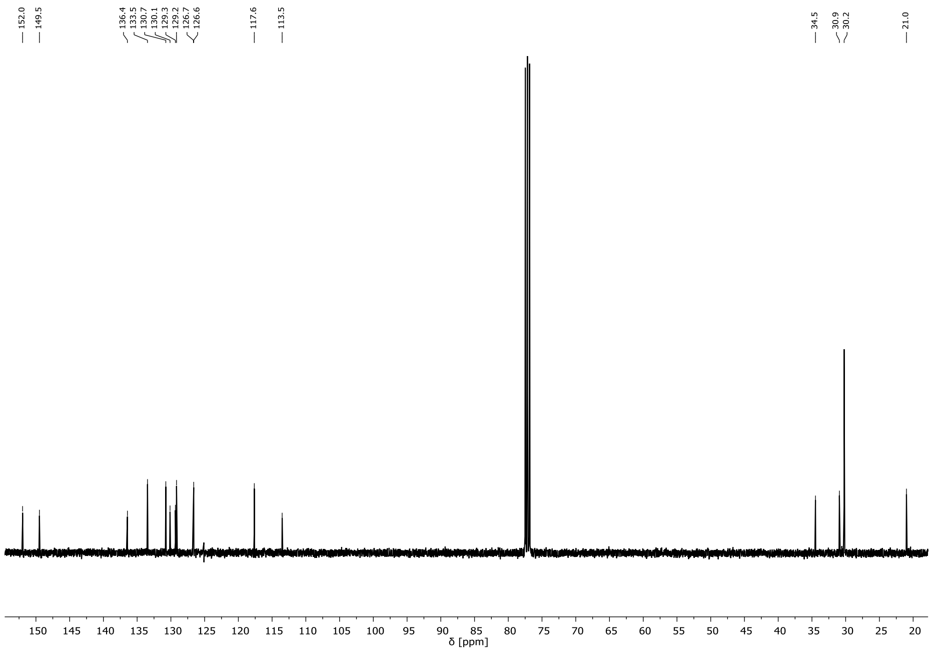

Figure S88 101 MHz ^{13}C-NMR of **7** in $CDCl_3$.

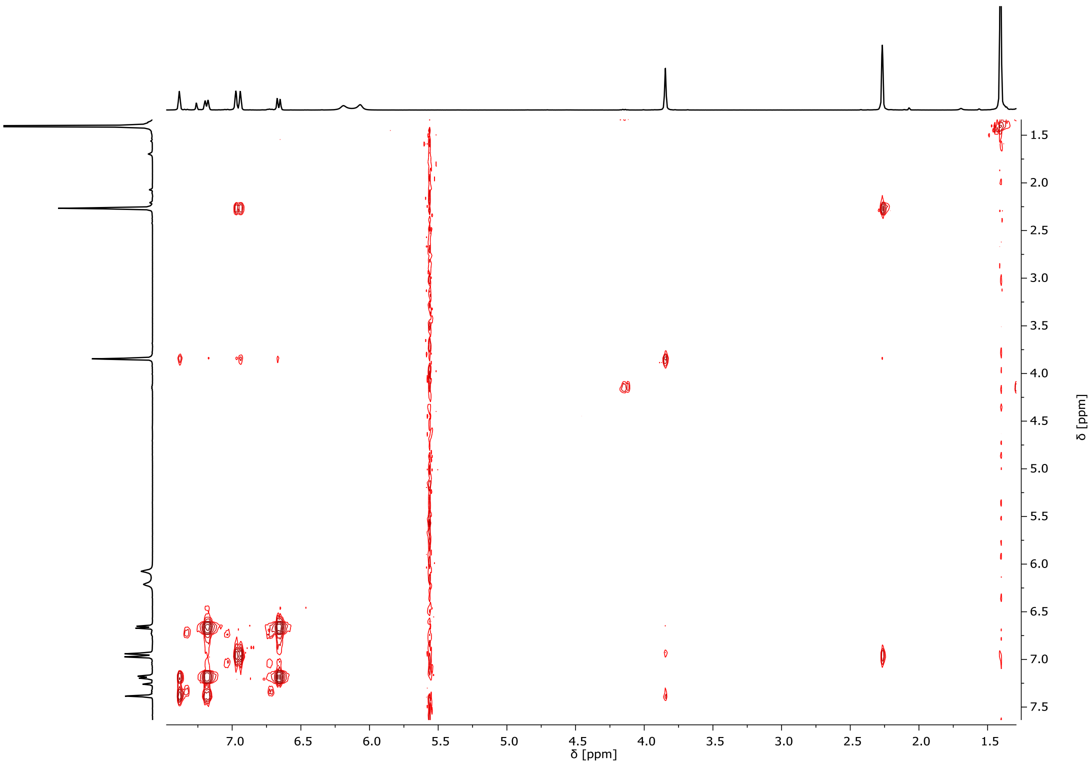

Figure S89 400 MHz ^{1}H-^{1}H COSY spectrum of **7** in $CDCl_3$.

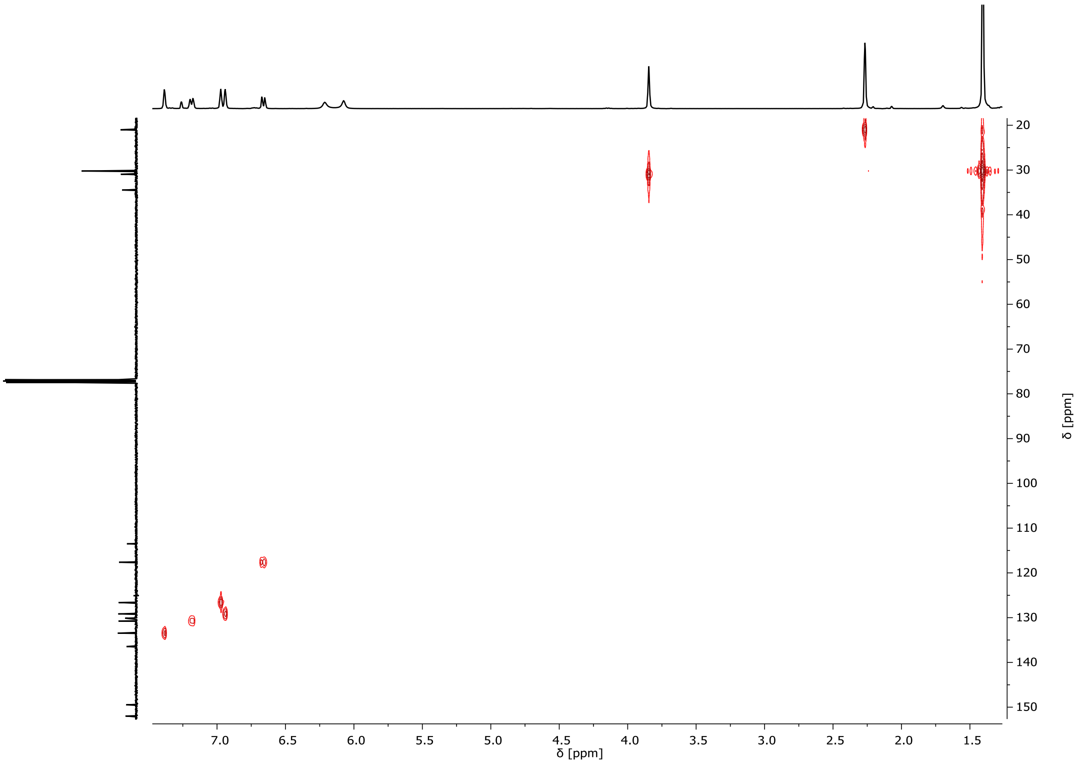

Figure S90 400 MHz ^{1}H-^{13}C Heteronuclear Single Quantum Coherence (HSQC) spectrum of **7** in $CDCl_3$.

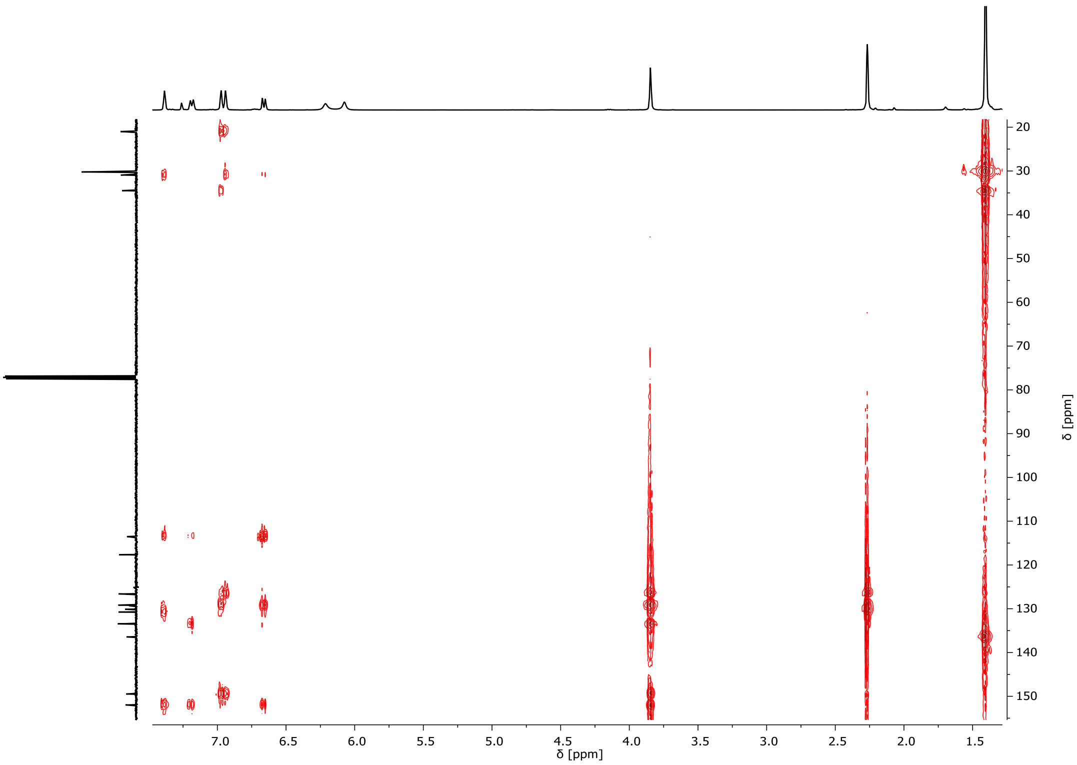

Figure S91 400 MHz ^{1}H-^{13}C Heteronuclear Multiple Bond Correlation (HMBC) spectrum of **7** in $CDCl_3$.

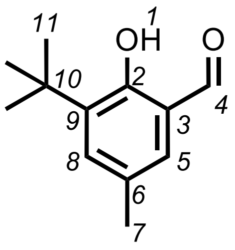

34

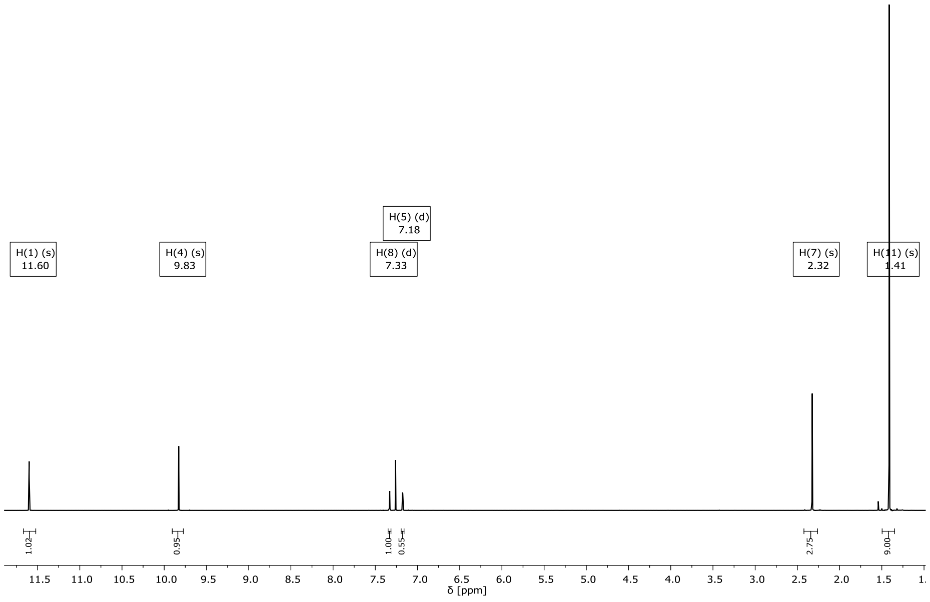

Figure S92 400 MHz ^{1}H-NMR of **34** in $CDCl_3$.

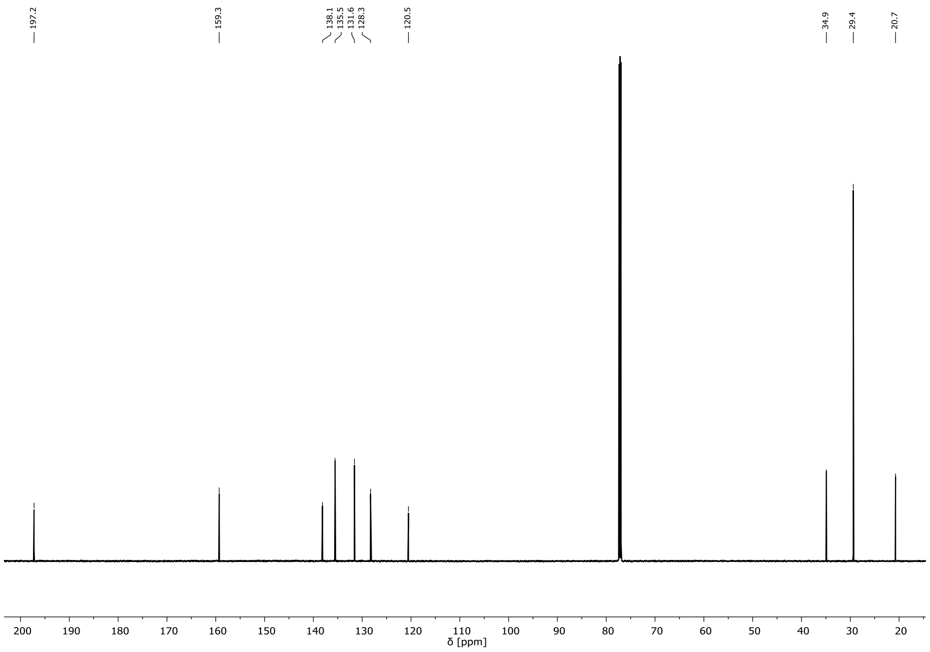

Figure S93 101 MHz ^{13}C-NMR of **34** in $CDCl_3$.

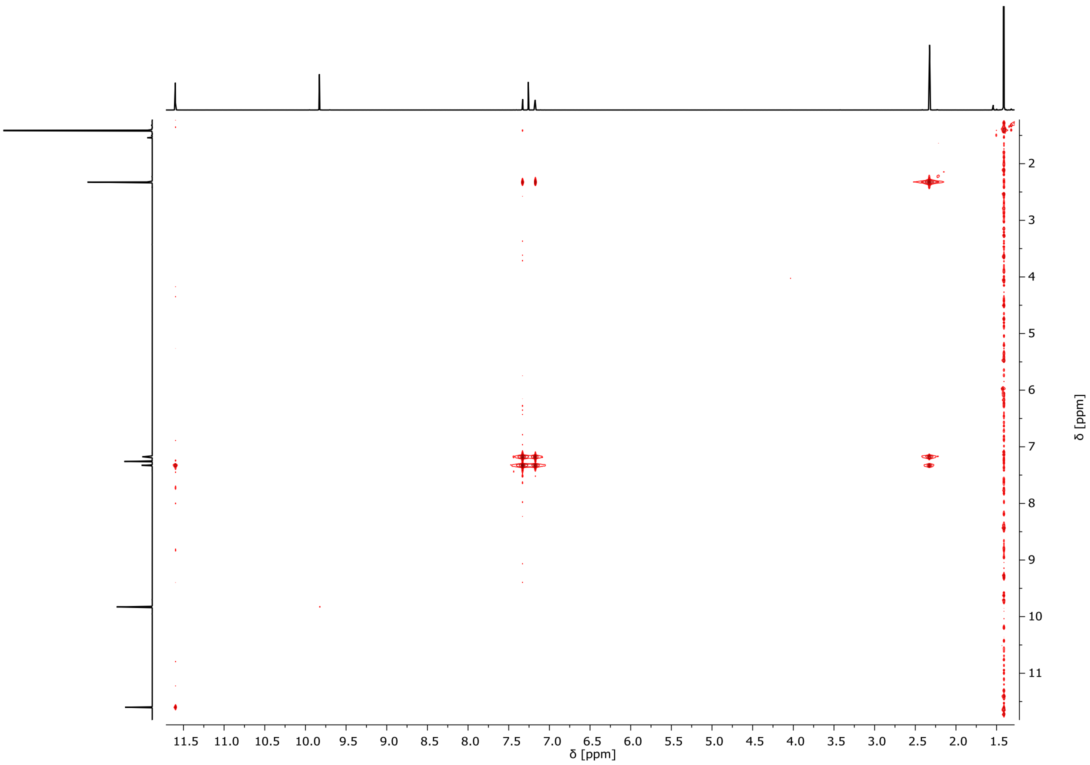

Figure S94 400 MHz ^{1}H-^{1}H COSY spectrum of **34** in $CDCl_3$.

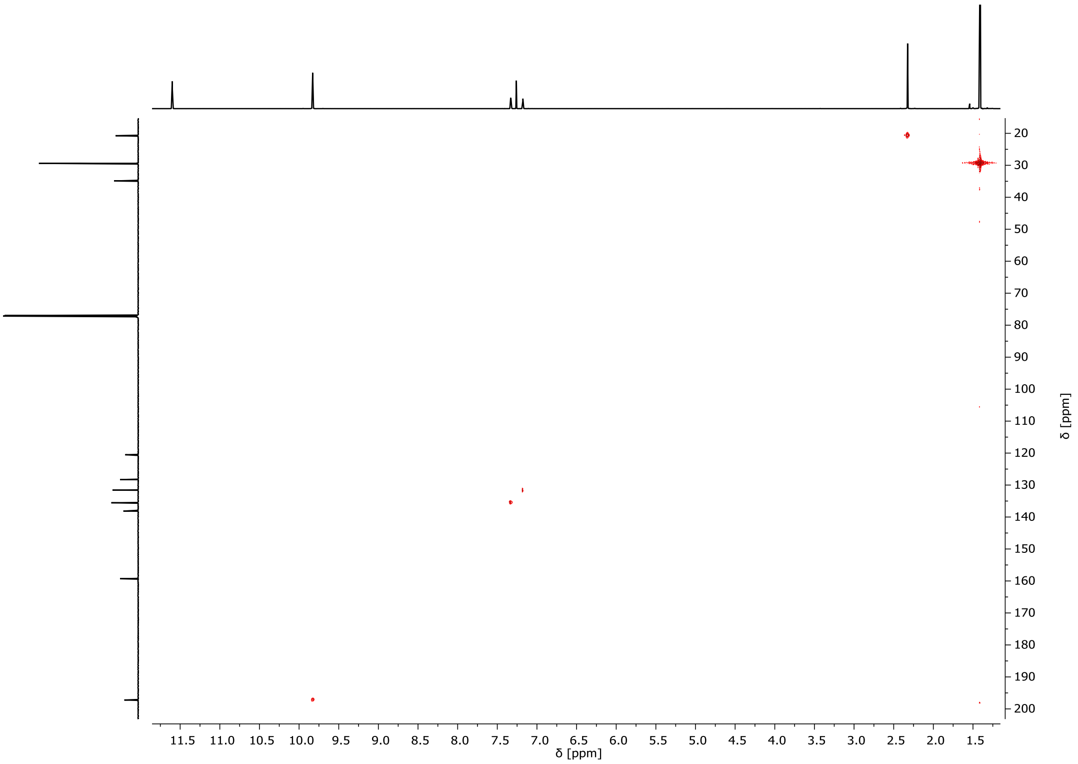

Figure S95 400 MHz ^{1}H-^{13}C Heteronuclear Single Quantum Coherence (HSQC) spectrum of **34** in $CDCl_3$.

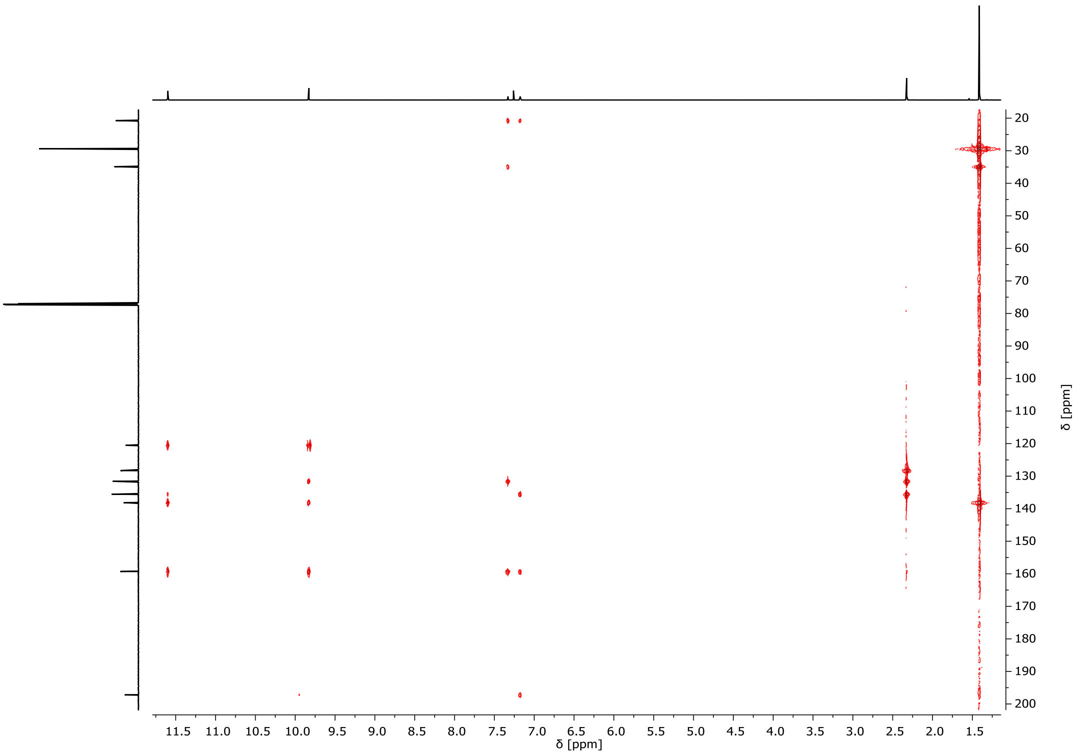

Figure S96 400 MHz ^{1}H-^{13}C Heteronuclear Multiple Bond Correlation (HMBC) spectrum of **34** in $CDCl_3$.

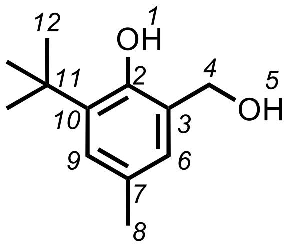

35

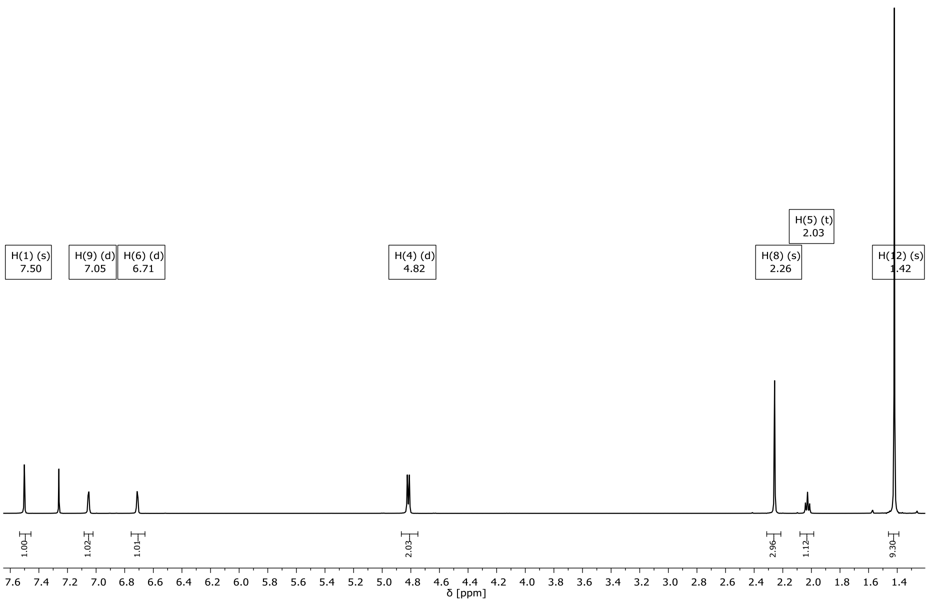

Figure S97 400 MHz ^{1}H-NMR of **35** in $CDCl_3$.

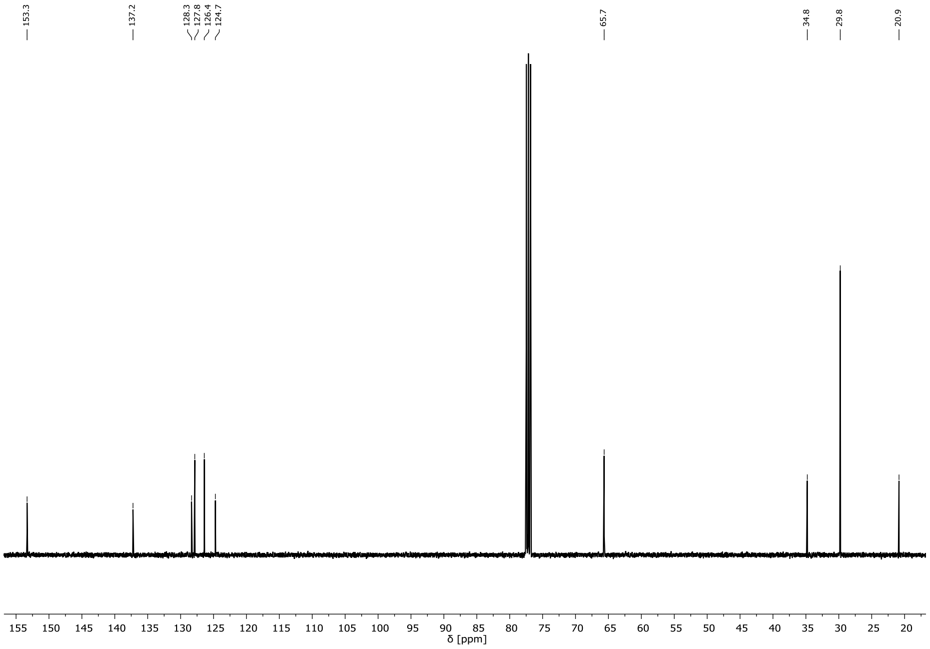

Figure S98 101 MHz ^{13}C-NMR of **35** in $CDCl_3$.

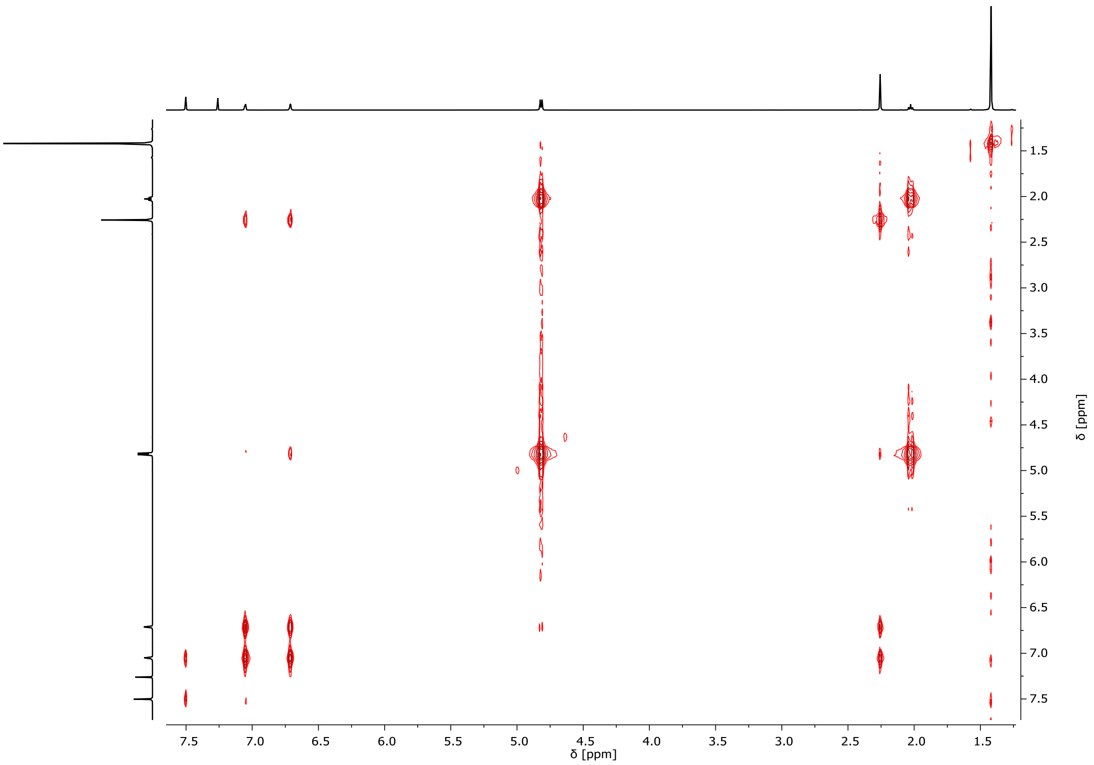

Figure S99 400 MHz ^{1}H-^{1}H COSY spectrum of **35** in $CDCl_3$.

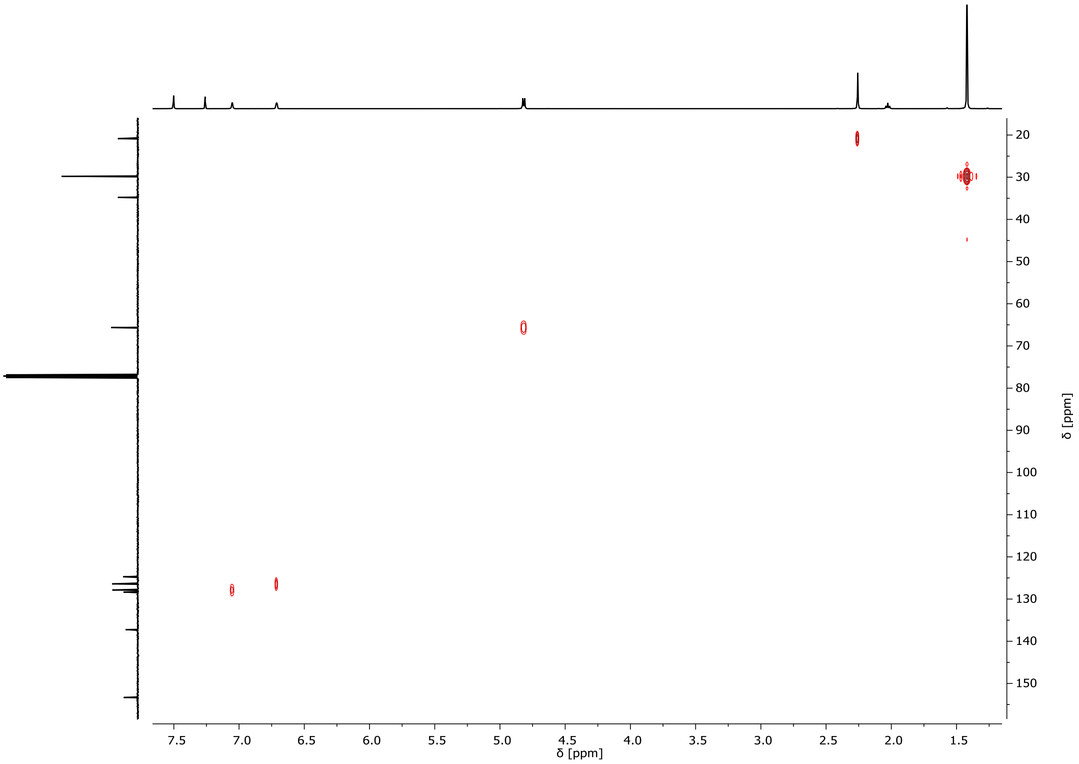

Figure S100 400 MHz ^{1}H-^{13}C Heteronuclear Single Quantum Coherence (HSQC) spectrum of **35** in $CDCl_3$.

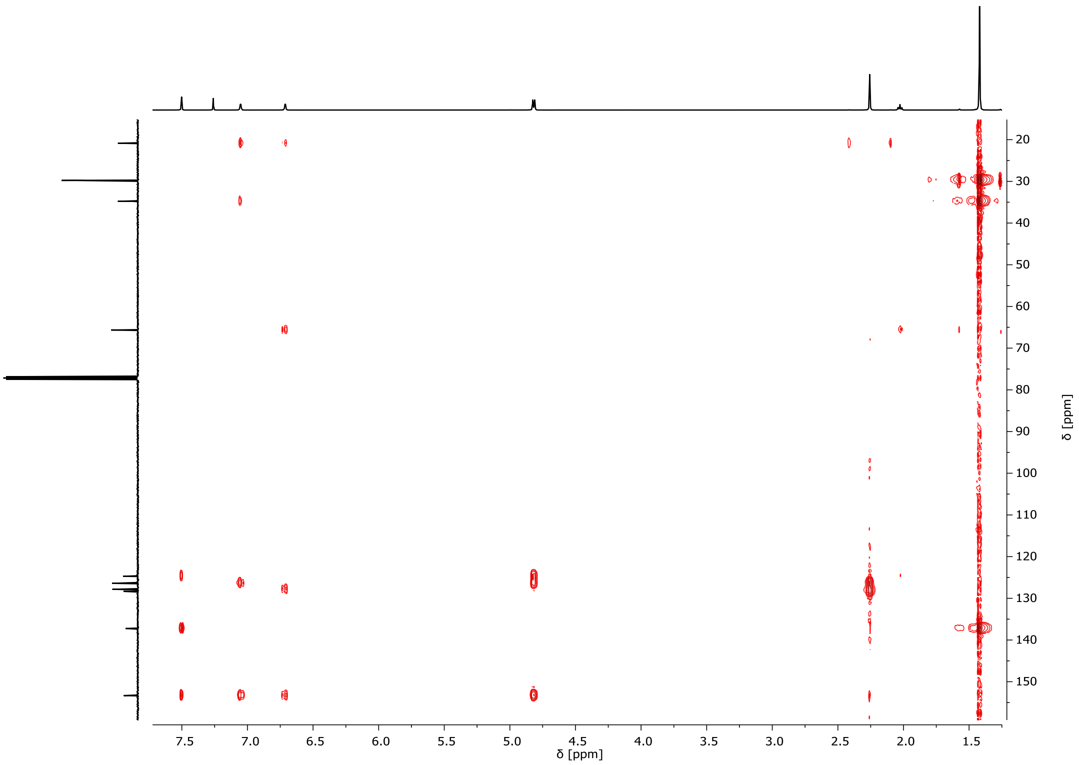

Figure S101 400 MHz ^{1}H-^{13}C Heteronuclear Multiple Bond Correlation (HMBC) spectrum of **35** in $CDCl_3$.

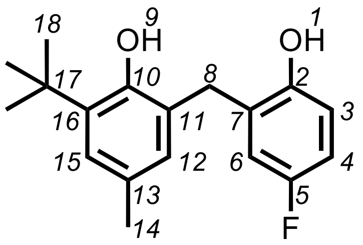

8

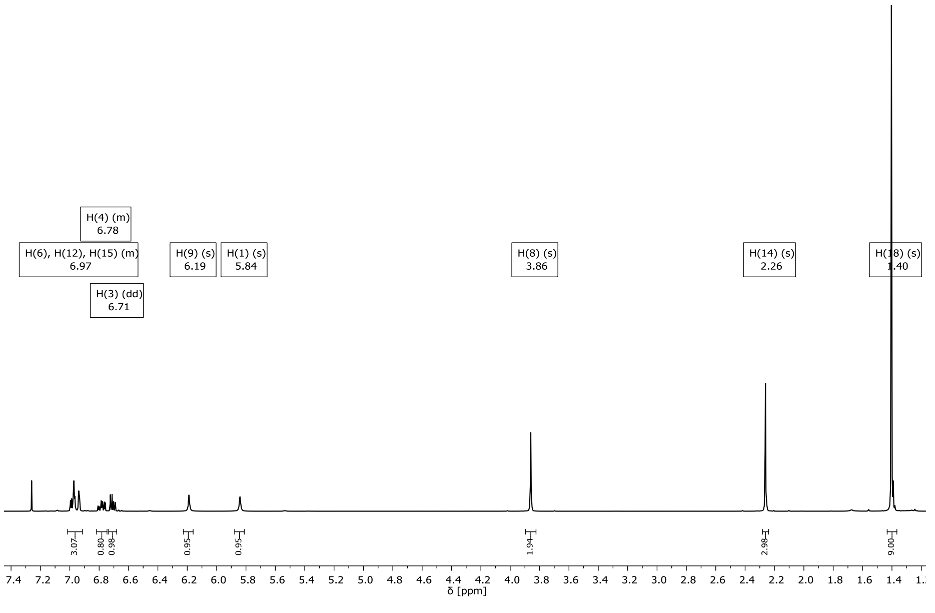

Figure S102 400 MHz ^{1}H-NMR of **8** in $CDCl_3$.

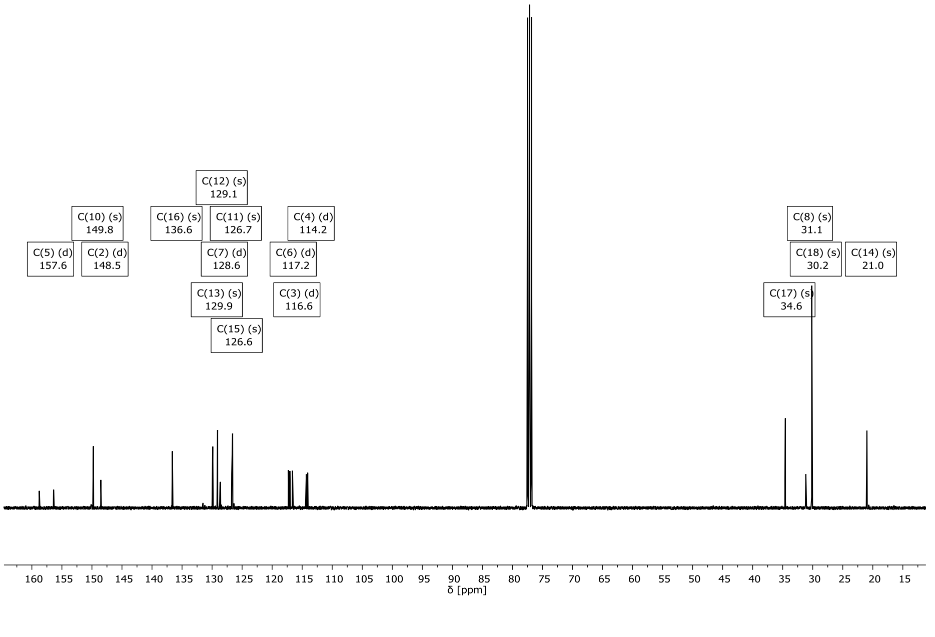

Figure S103 101 MHz ^{13}C-NMR of **8** in $CDCl_3$.

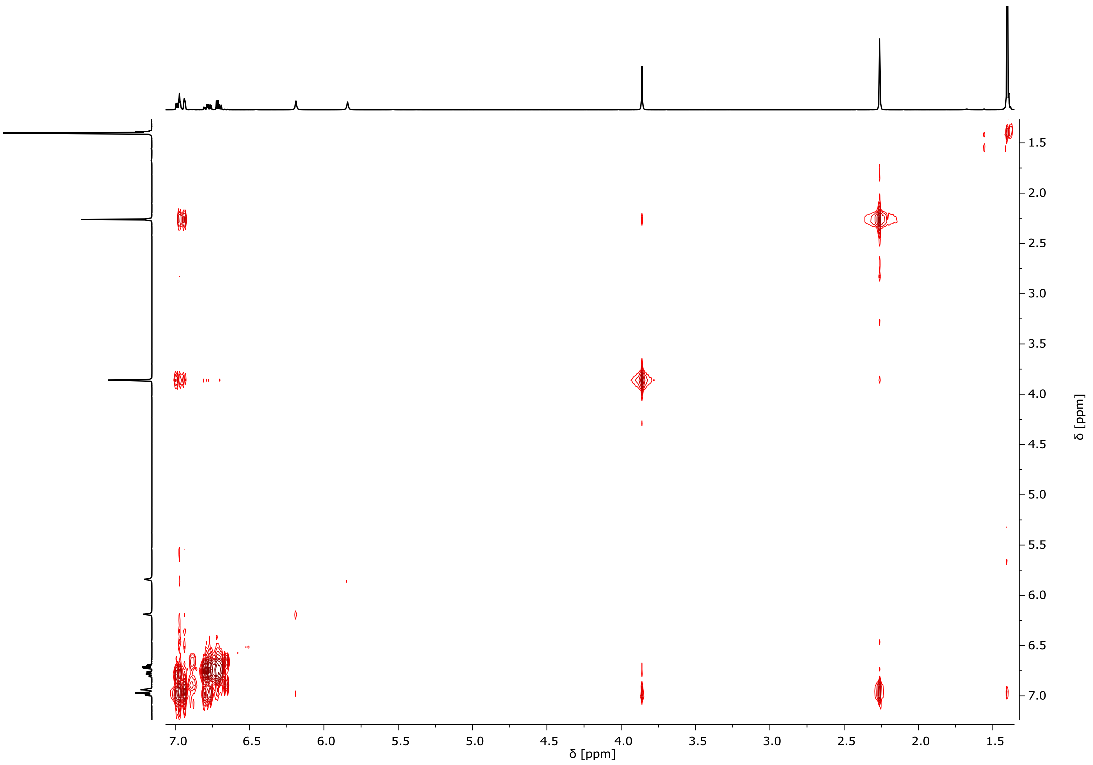

Figure S104 400 MHz ^{1}H-^{1}H COSY spectrum of **8** in $CDCl_3$.

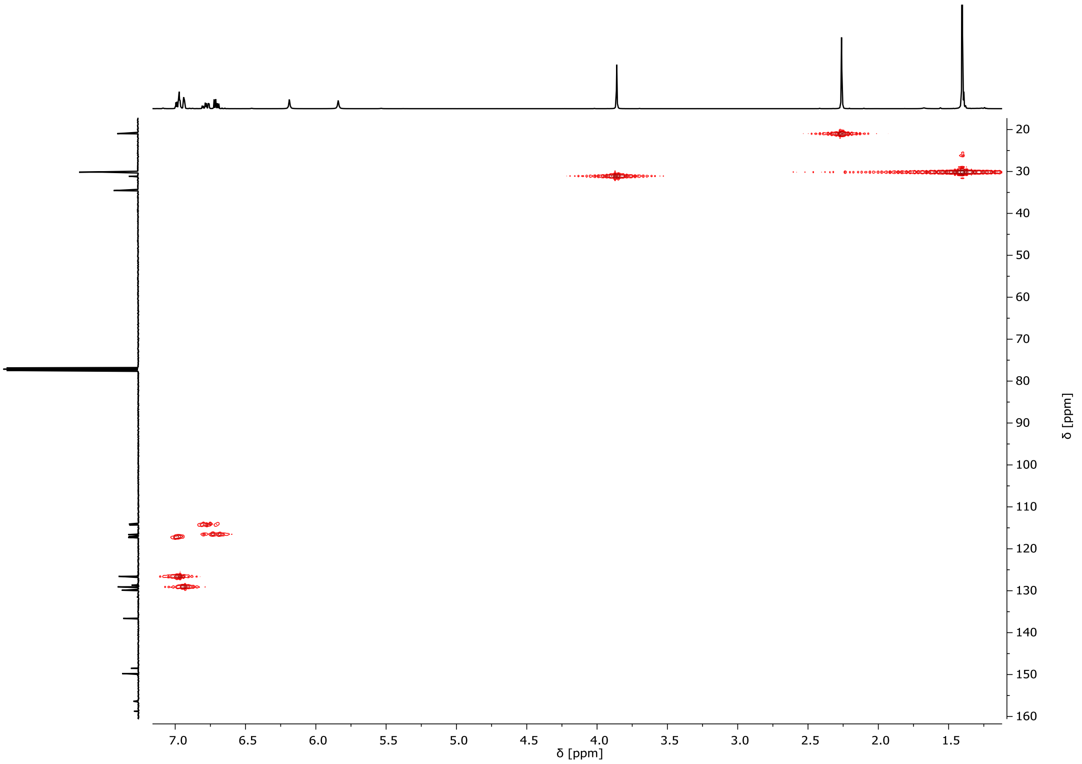

Figure S105 400 MHz ^{1}H-^{13}C Heteronuclear Single Quantum Coherence (HSQC) spectrum of **8** in $CDCl_3$.

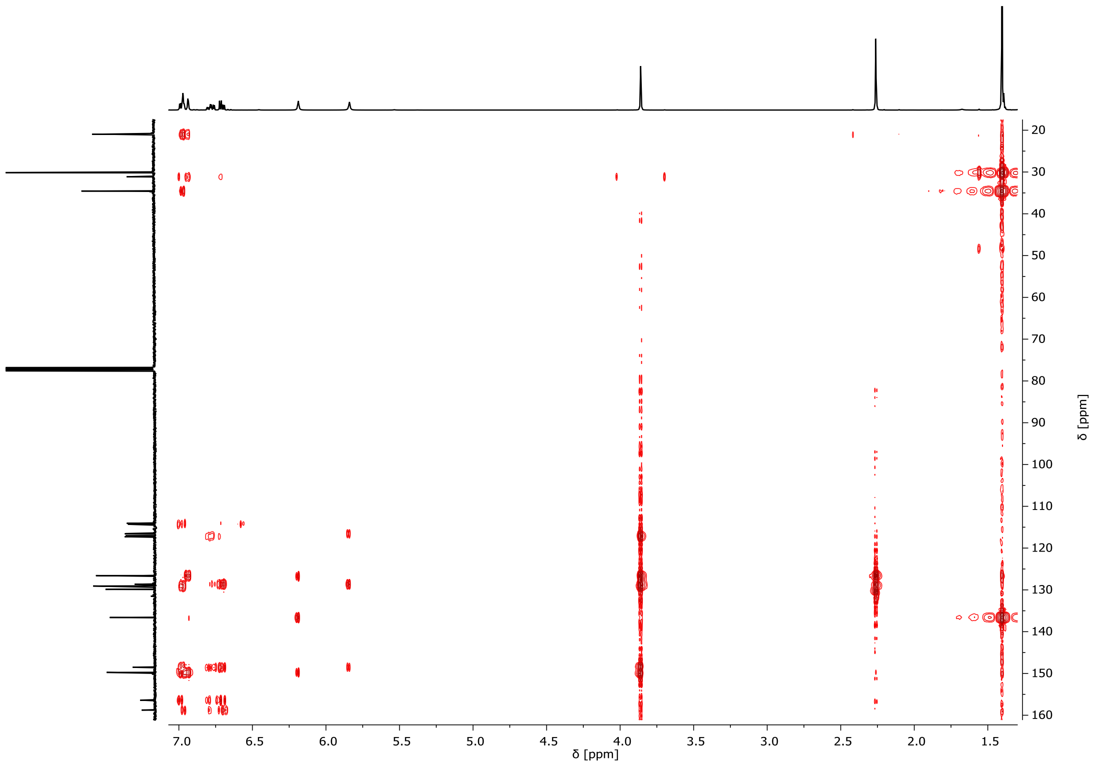

Figure S106 400 MHz ^{1}H-^{13}C Heteronuclear Multiple Bond Correlation (HMBC) spectrum of **8** in $CDCl_3$.

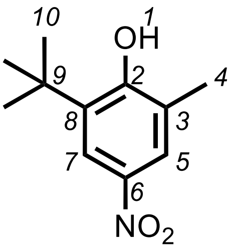

10

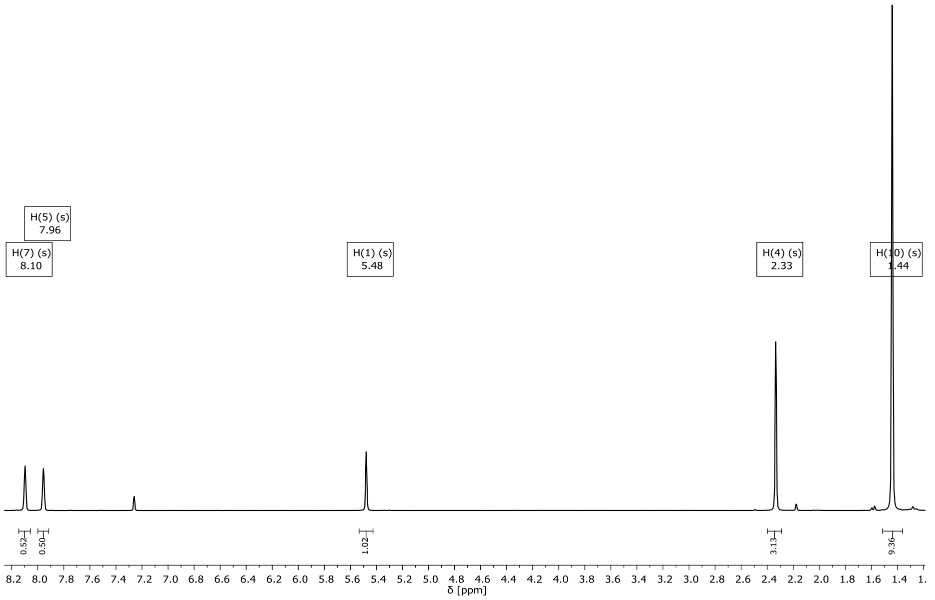

Figure S107 400 MHz ^{1}H-NMR of **10** in CDCl$_3$.

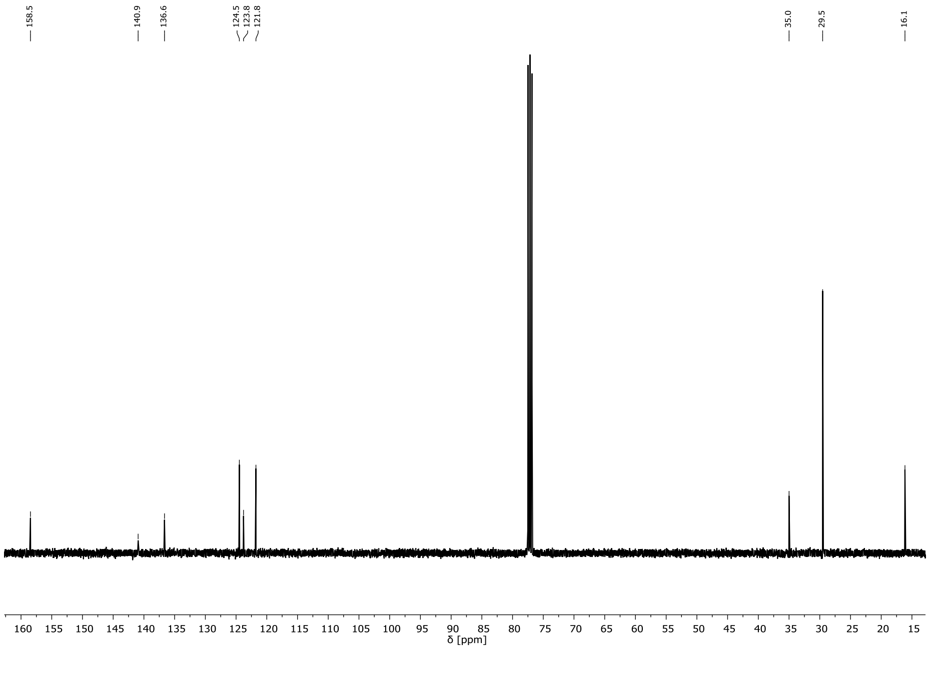

Figure S108 101 MHz ^{13}C-NMR of **10** in CDCl$_3$.

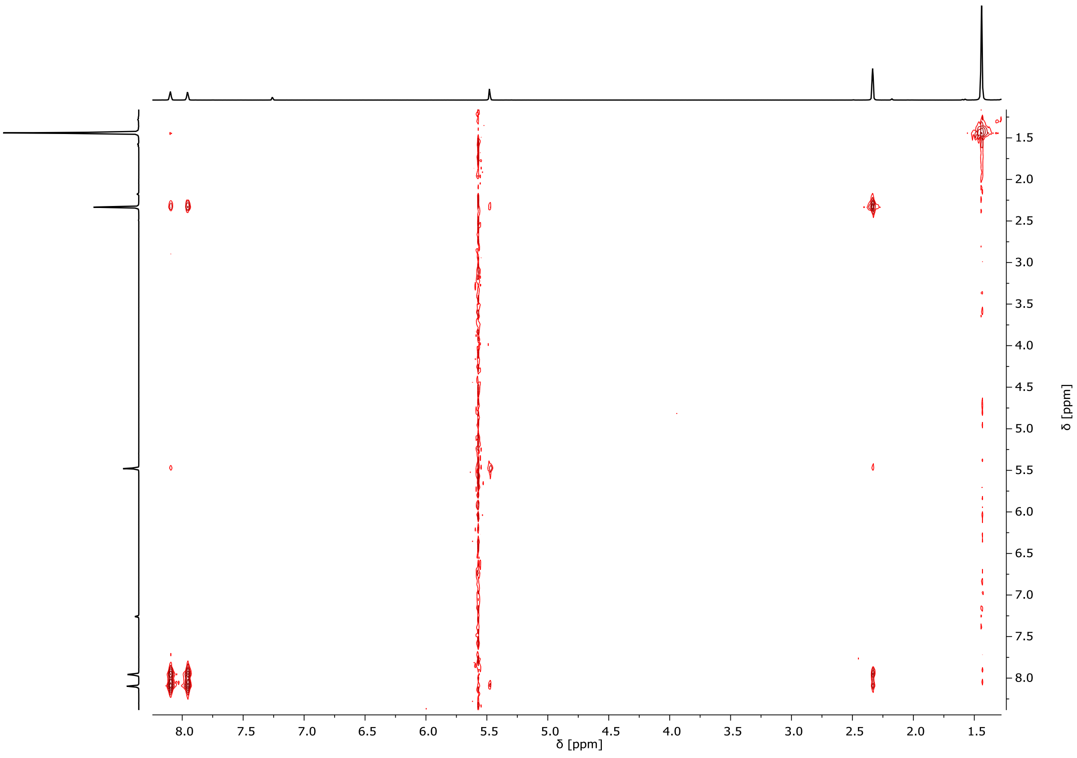

Figure S109 400 MHz ^{1}H-^{1}H COSY spectrum of **10** in $CDCl_3$.

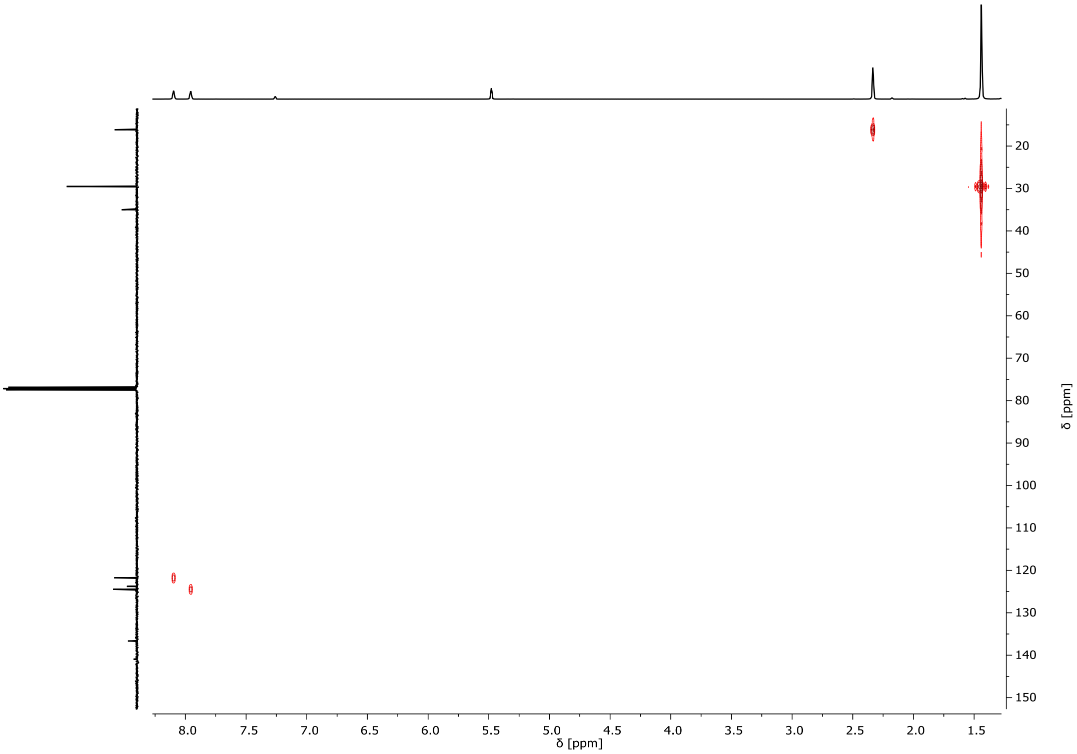

Figure S110 400 MHz ^{1}H-^{13}C Heteronuclear Single Quantum Coherence (HSQC) spectrum of **10** in $CDCl_3$.

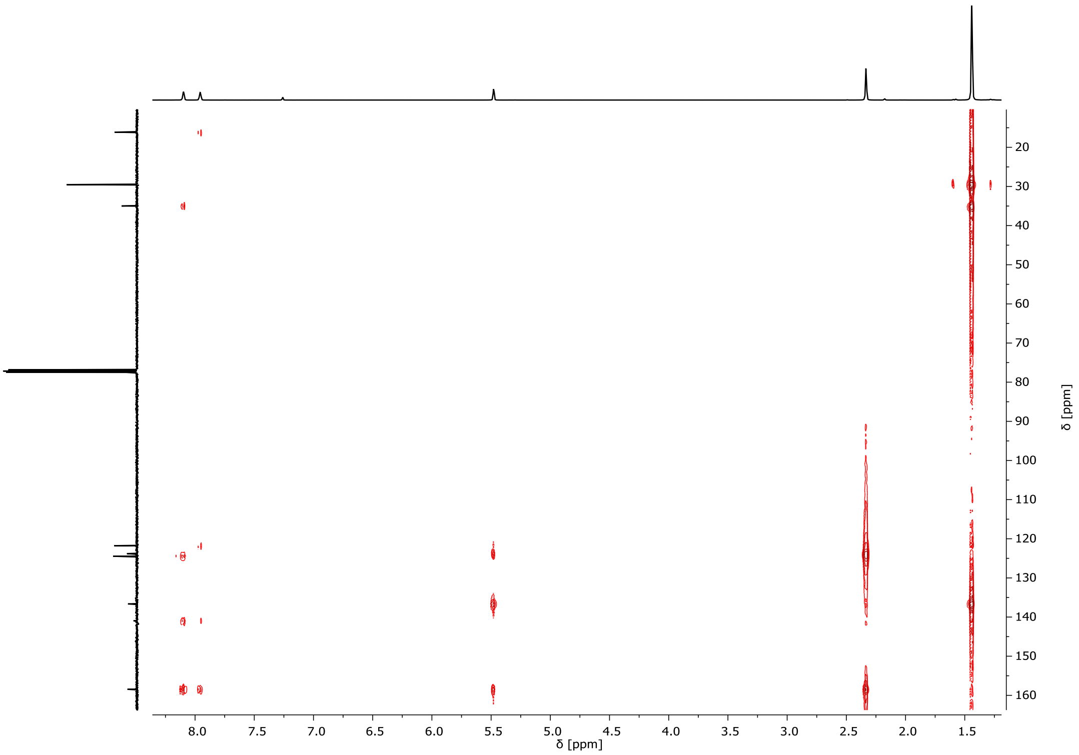

Figure S111 400 MHz ^{1}H-[13]C Heteronuclear Multiple Bond Correlation (HMBC) spectrum of **10** in $CDCl_3$.

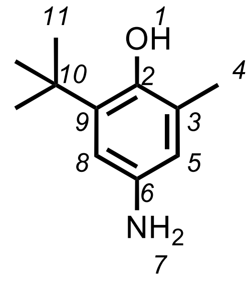

40

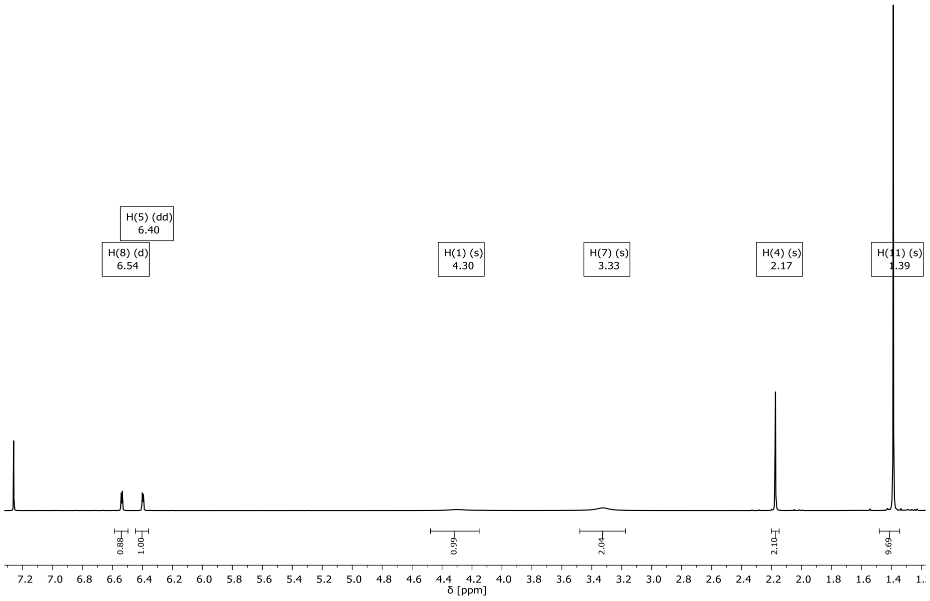

Figure S112 400 MHz ^{1}H-NMR of **40** in $CDCl_3$.

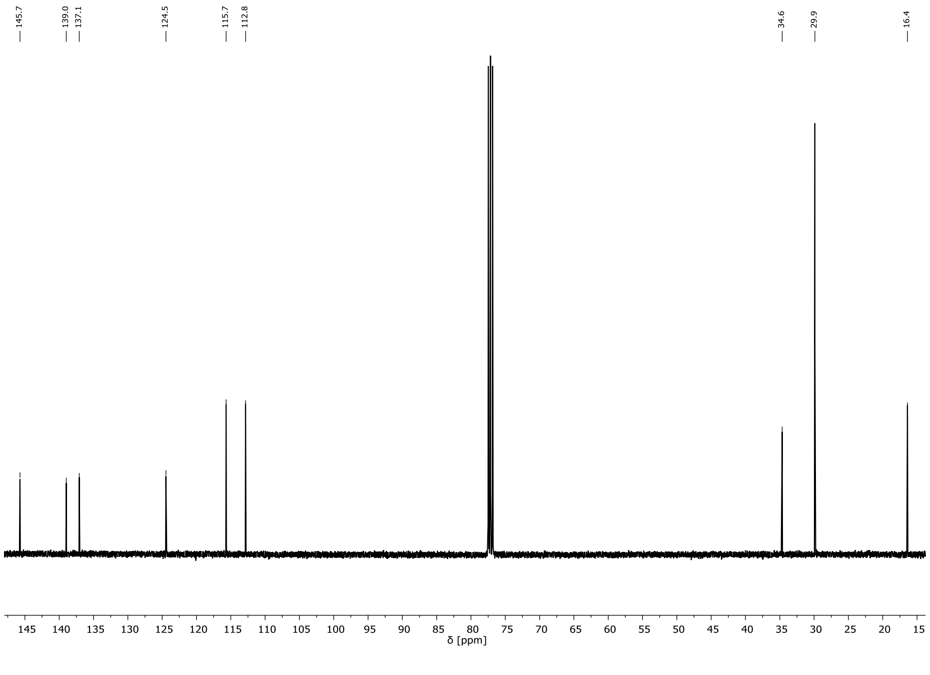

Figure S113 101 MHz ^{13}C-NMR of **40** in $CDCl_3$.

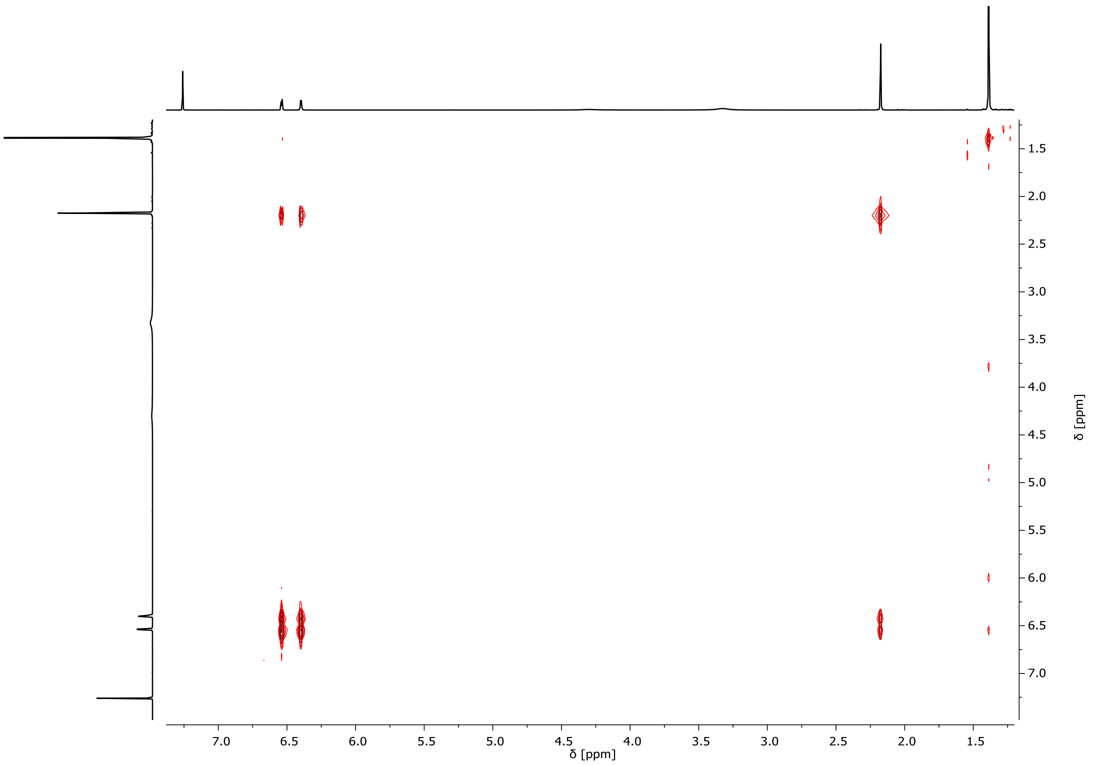

Figure S114 400 MHz ^{1}H-^{1}H COSY spectrum of **40** in $CDCl_3$.

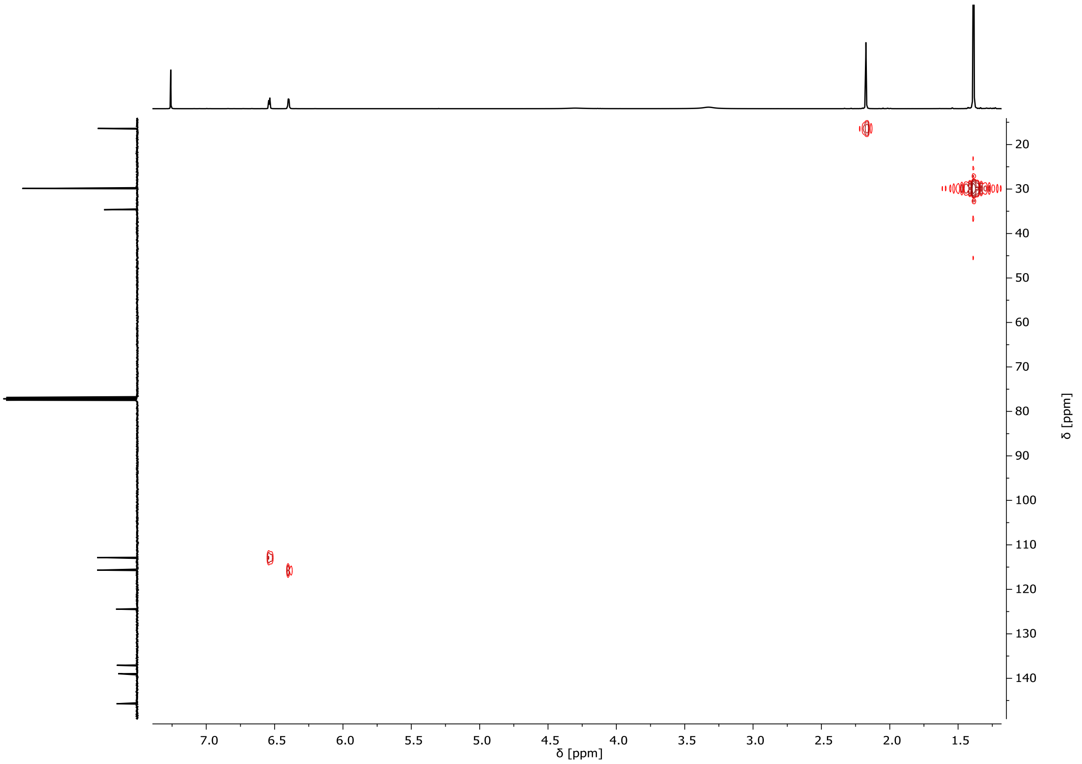

Figure S115 400 MHz ^{1}H-^{13}C Heteronuclear Single Quantum Coherence (HSQC) spectrum of **40** in $CDCl_3$.

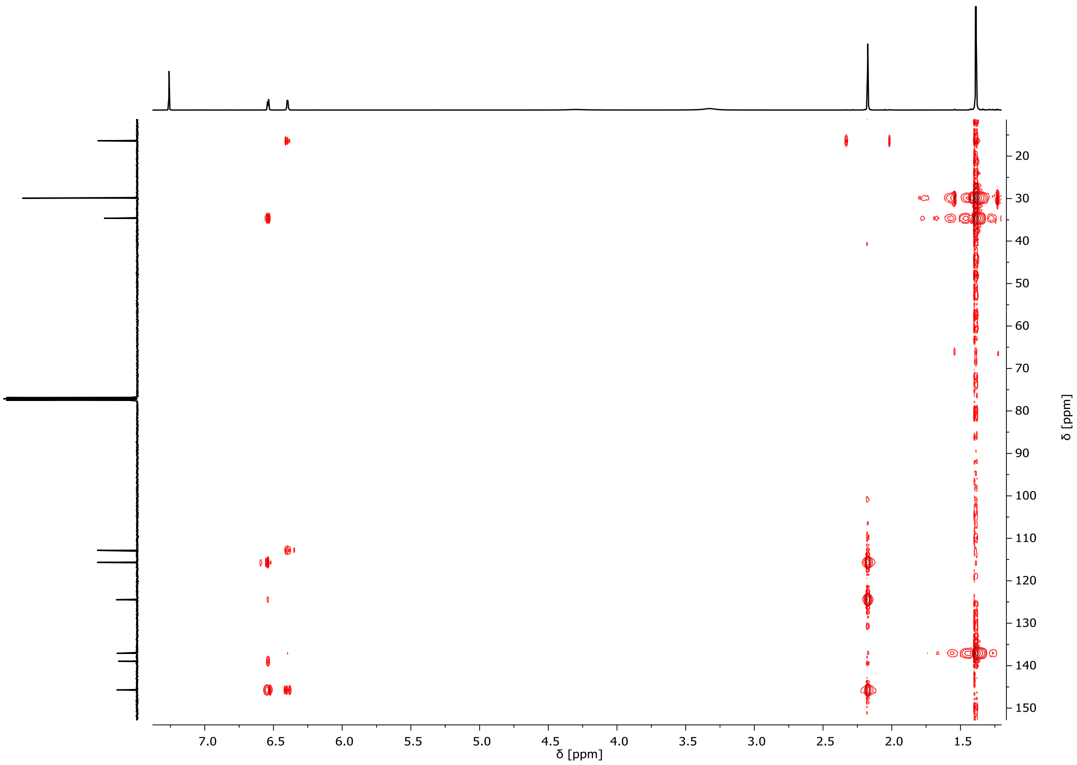

Figure S116 400 MHz ^{1}H-[13]C Heteronuclear Multiple Bond Correlation (HMBC) spectrum of **40** in $CDCl_3$.

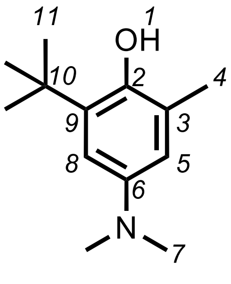

14

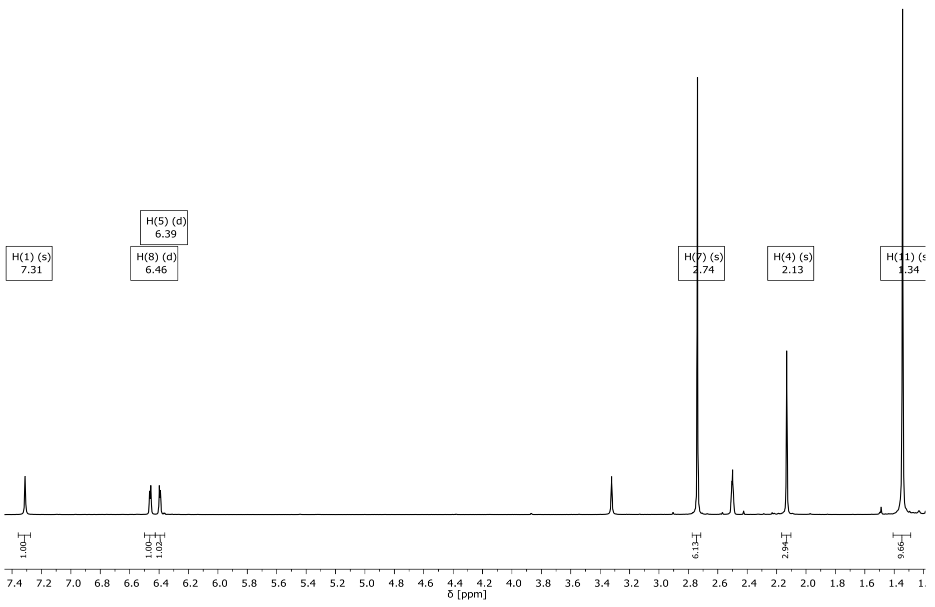

Figure S117 400 MHz ^{1}H-NMR of **14** in d$_6$-DMSO.

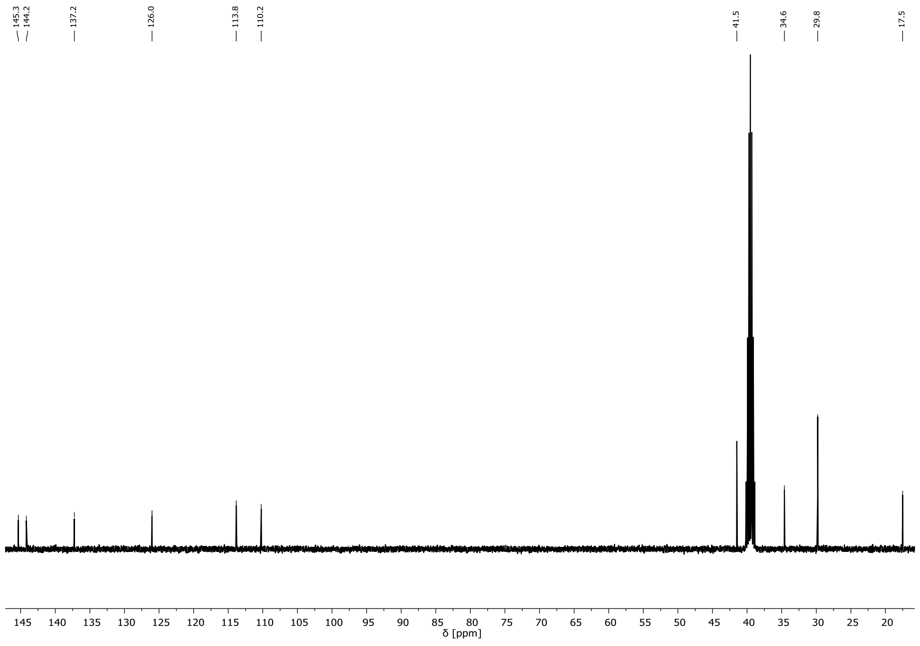

Figure S118 101 MHz ^{13}C-NMR of **14** in d$_6$-DMSO.

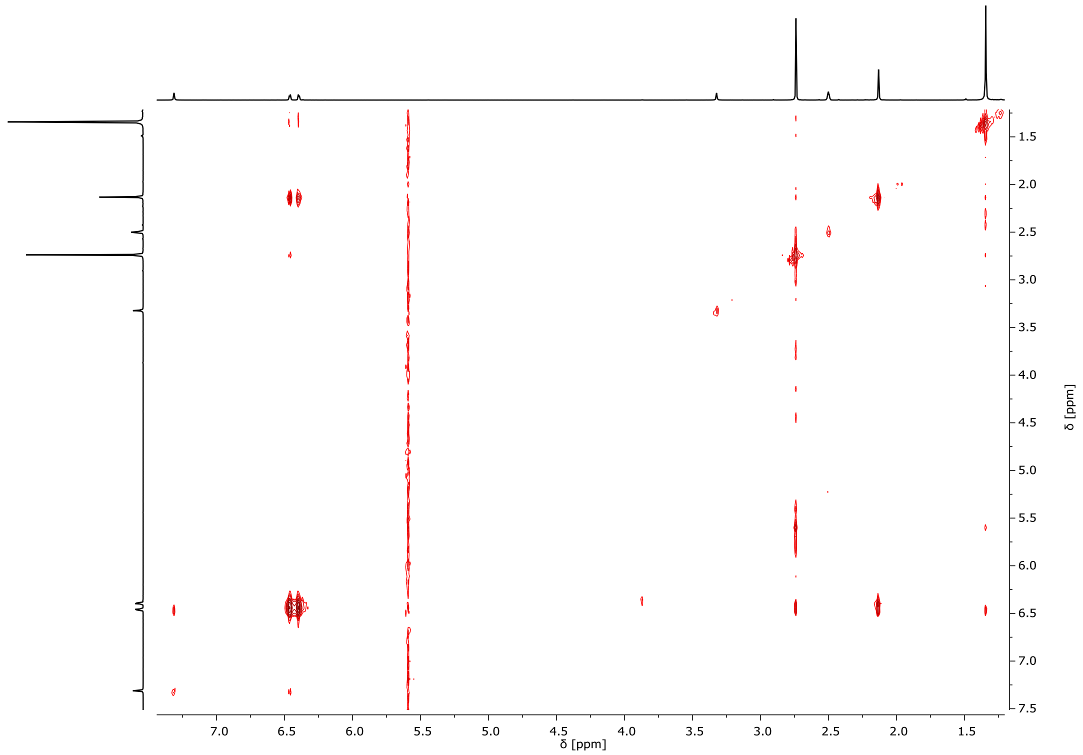

Figure S119 400 MHz ^{1}H-^{1}H COSY spectrum of **14** in d_6-DMSO.

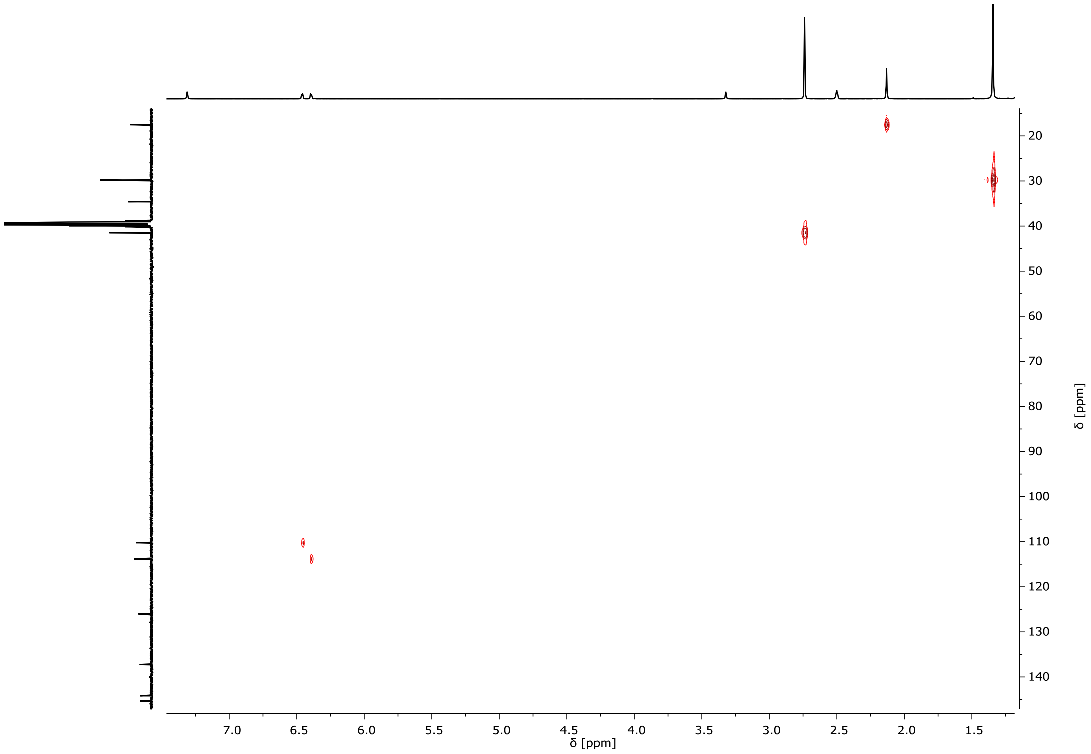

Figure S120 400 MHz ^{1}H-^{13}C Heteronuclear Single Quantum Coherence (HSQC) spectrum of **14** in d_6-DMSO.

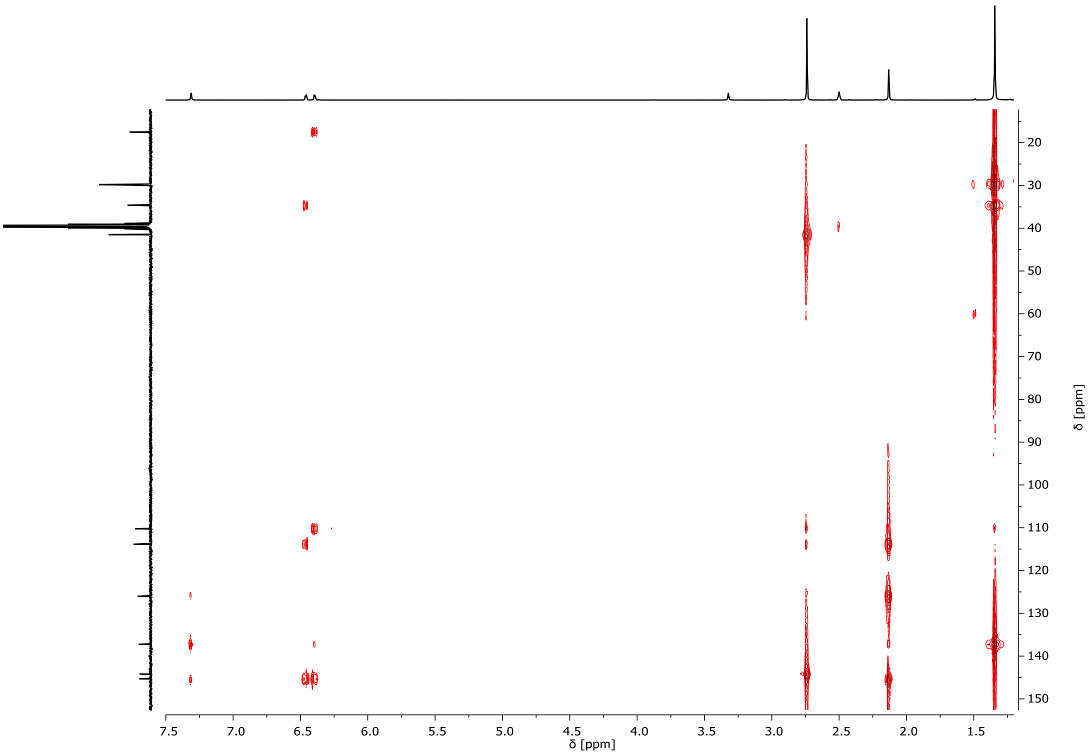

Figure S121 400 MHz ^{1}H-^{13}C Heteronuclear Multiple Bond Correlation (HMBC) spectrum of **14** in d_6-DMSO.

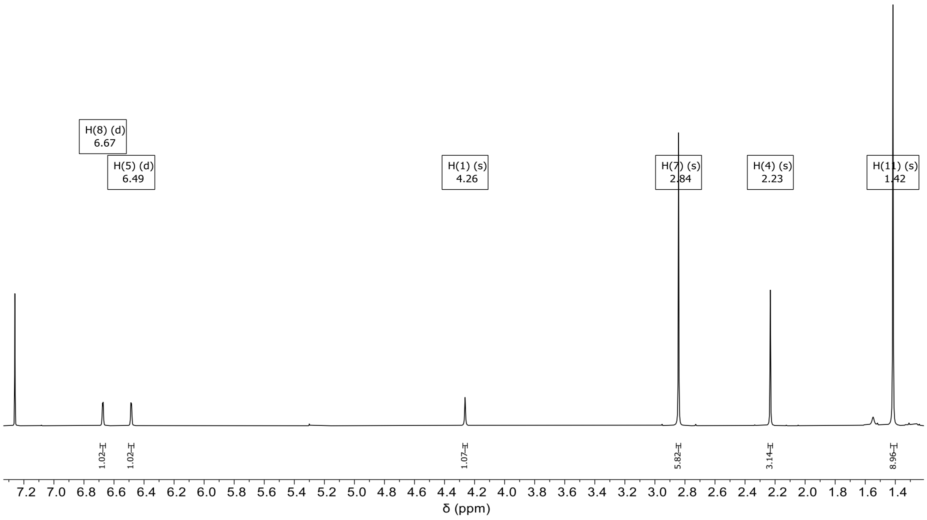

Figure S122 600 MHz ^{1}H-NMR of **14** in $CDCl_3$.

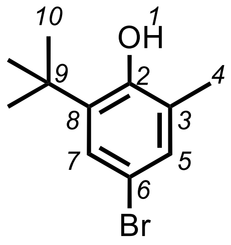

11

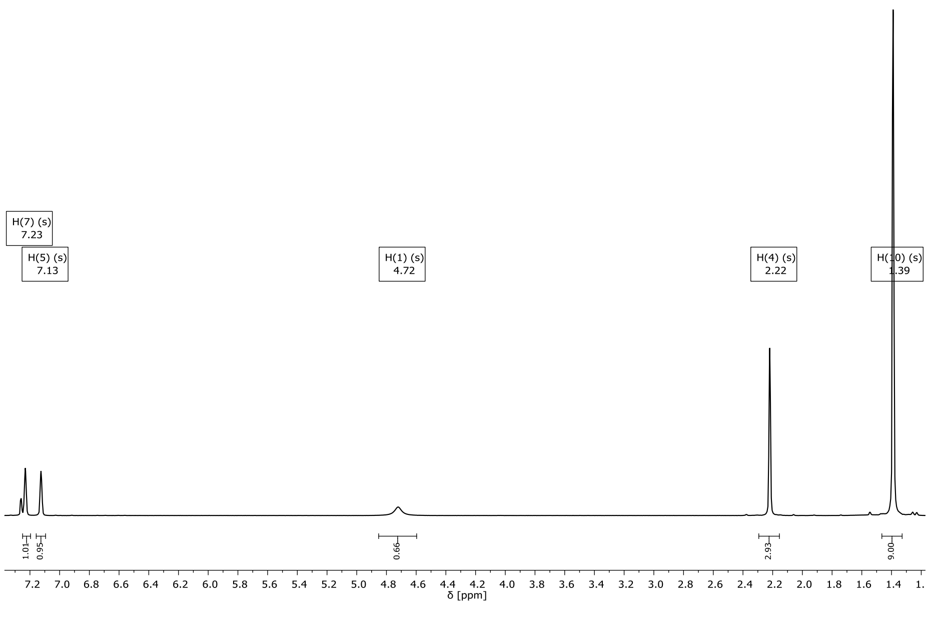

Figure S123 400 MHz ^{1}H-NMR of **11** in $CDCl_3$.

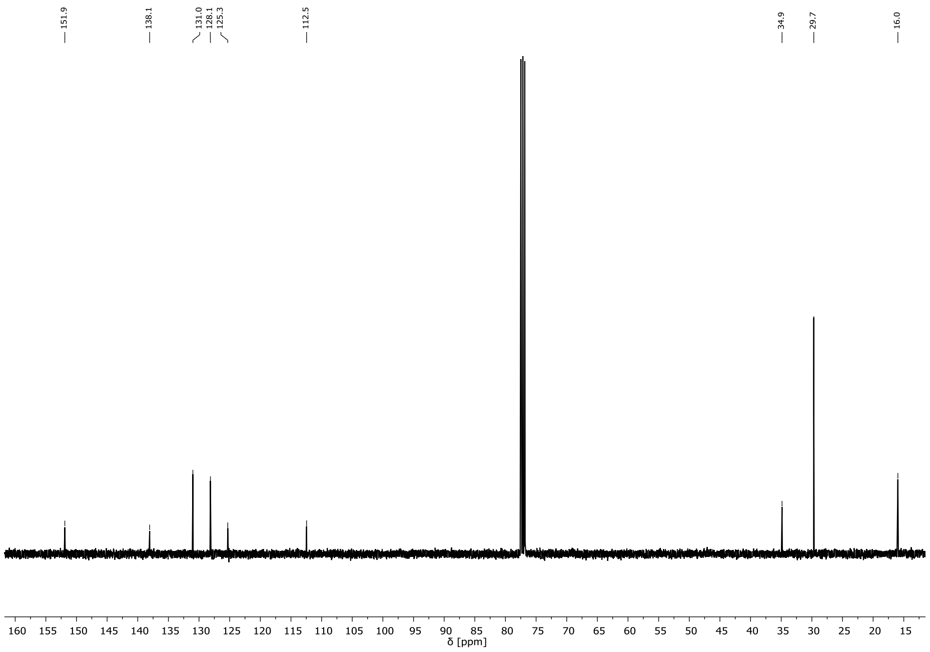

Figure S124 101 MHz ^{13}C-NMR of **11** in $CDCl_3$.

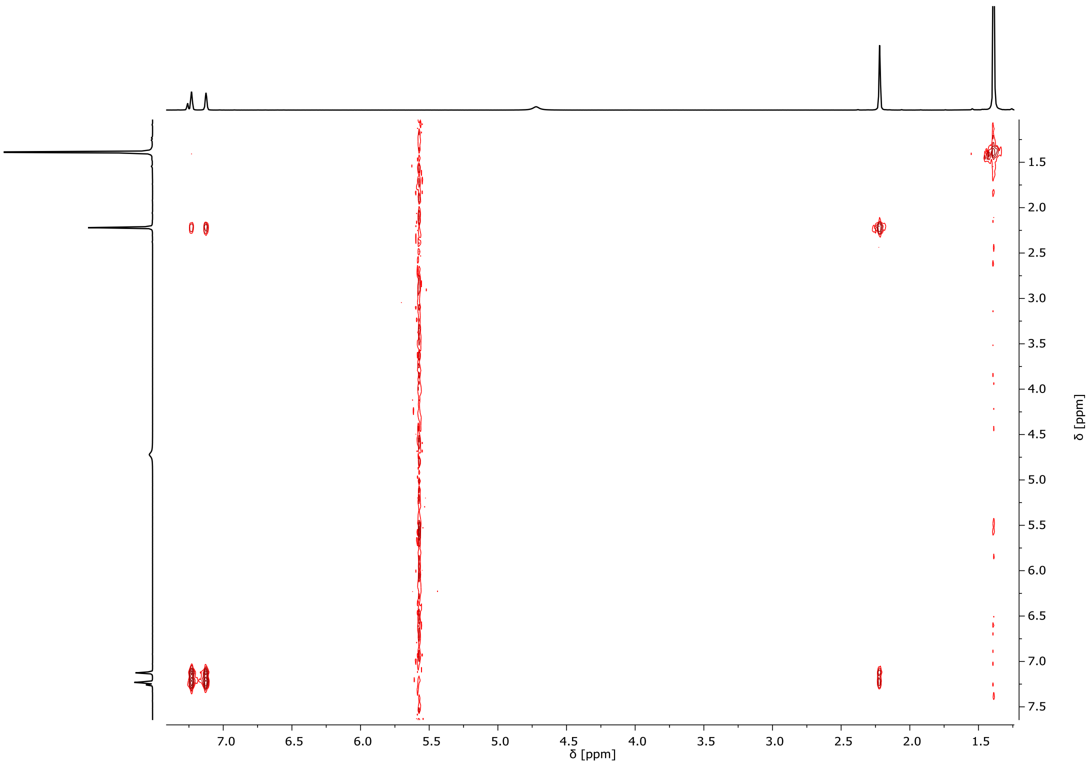

Figure S125 400 MHz ^{1}H-^{1}H COSY spectrum of **11** in $CDCl_3$.

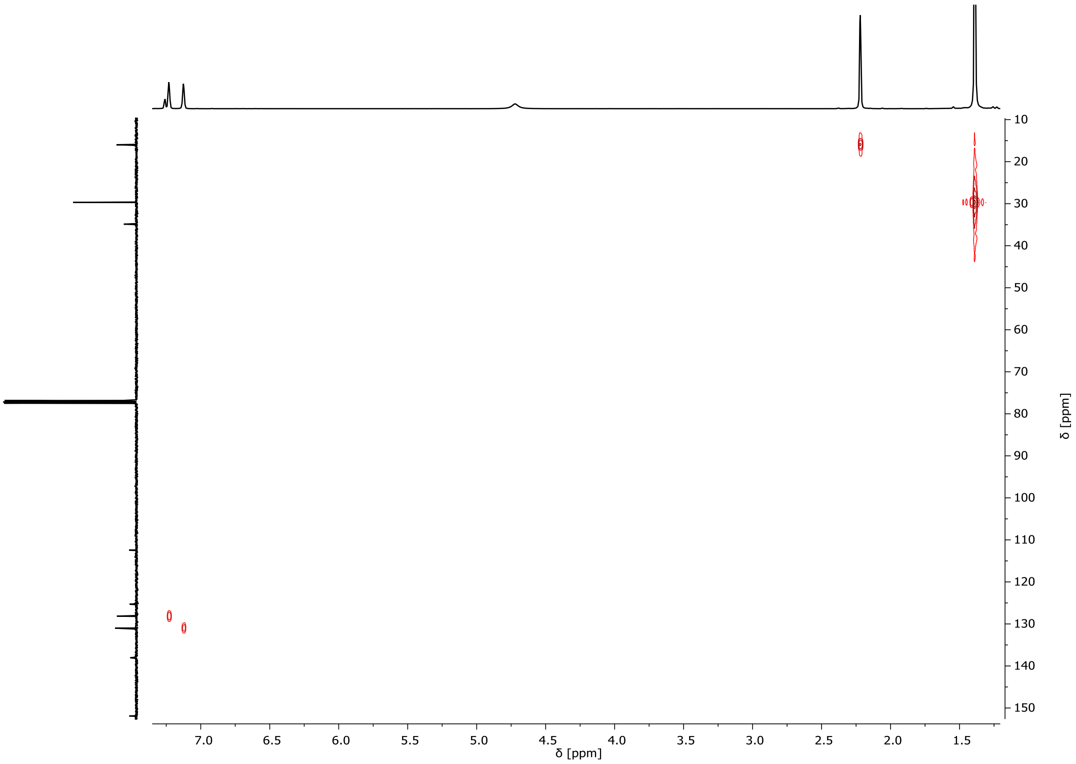

Figure S126 400 MHz ^{1}H-^{13}C Heteronuclear Single Quantum Coherence (HSQC) spectrum of **11** in $CDCl_3$.

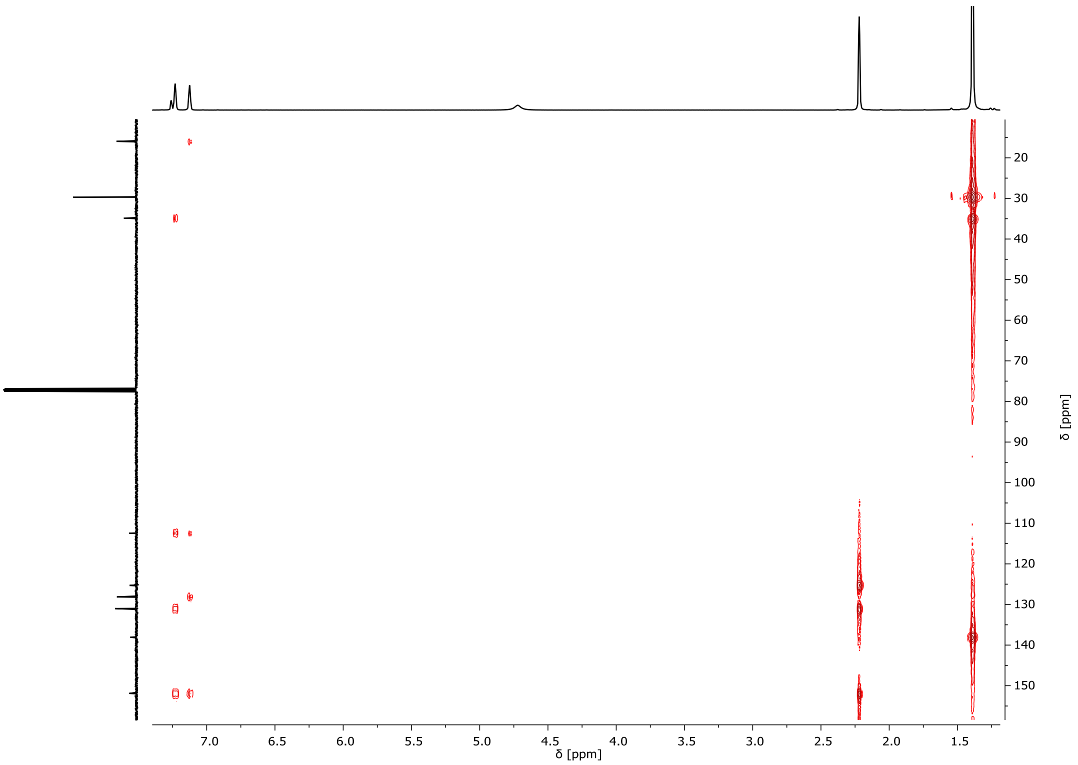

Figure S127 400 MHz ^{1}H-^{13}C Heteronuclear Multiple Bond Correlation (HMBC) spectrum of **11** in $CDCl_3$.

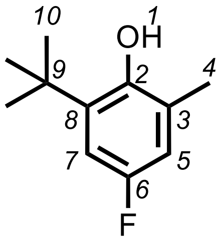

12

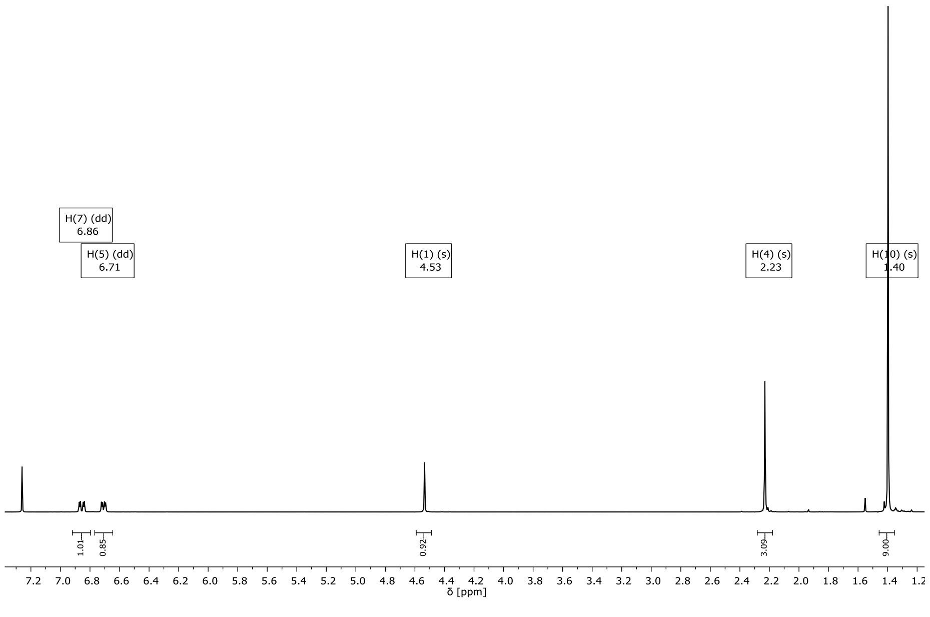

Figure S128 400 MHz ^{1}H-NMR of **12** in $CDCl_3$.

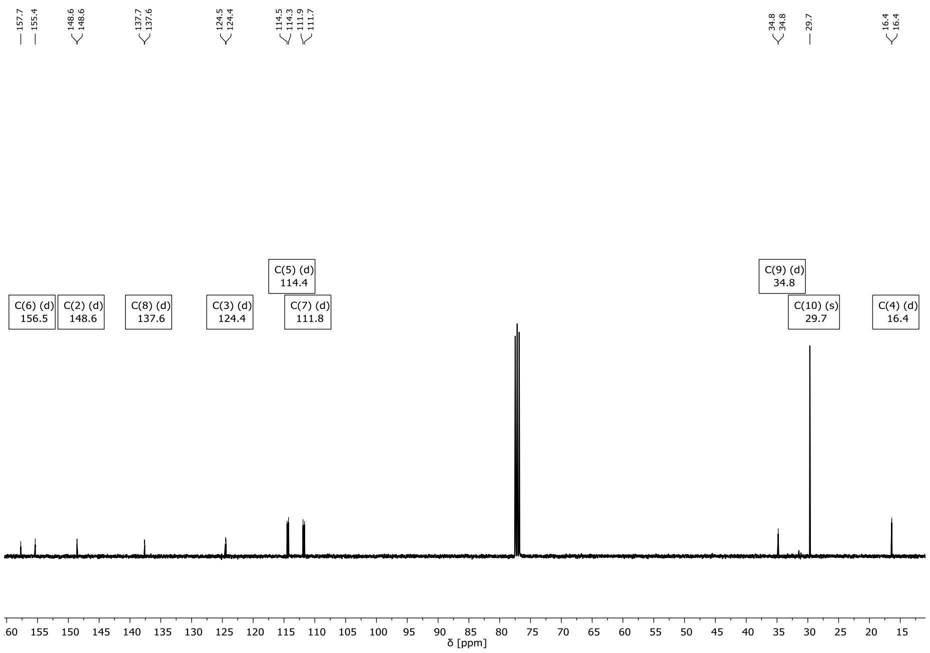

Figure S129 101 MHz ^{13}C-NMR of **12** in $CDCl_3$.

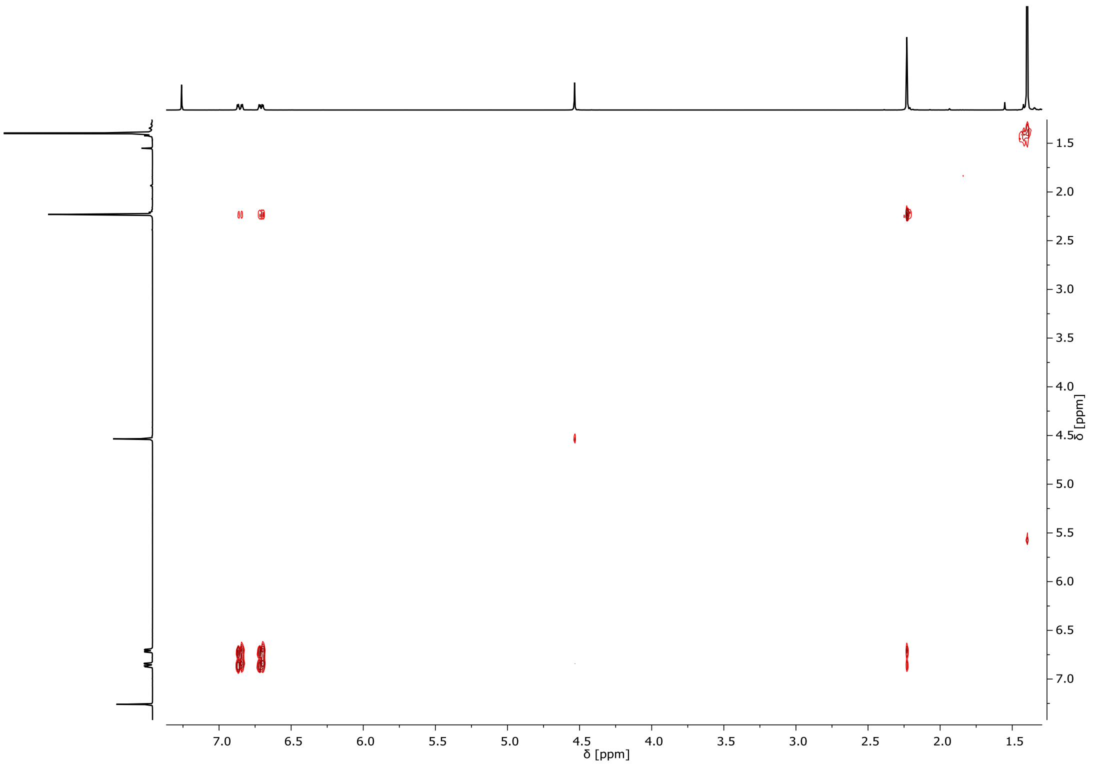

Figure S130 400 MHz ^{1}H-^{1}H COSY spectrum of **12** in $CDCl_3$.

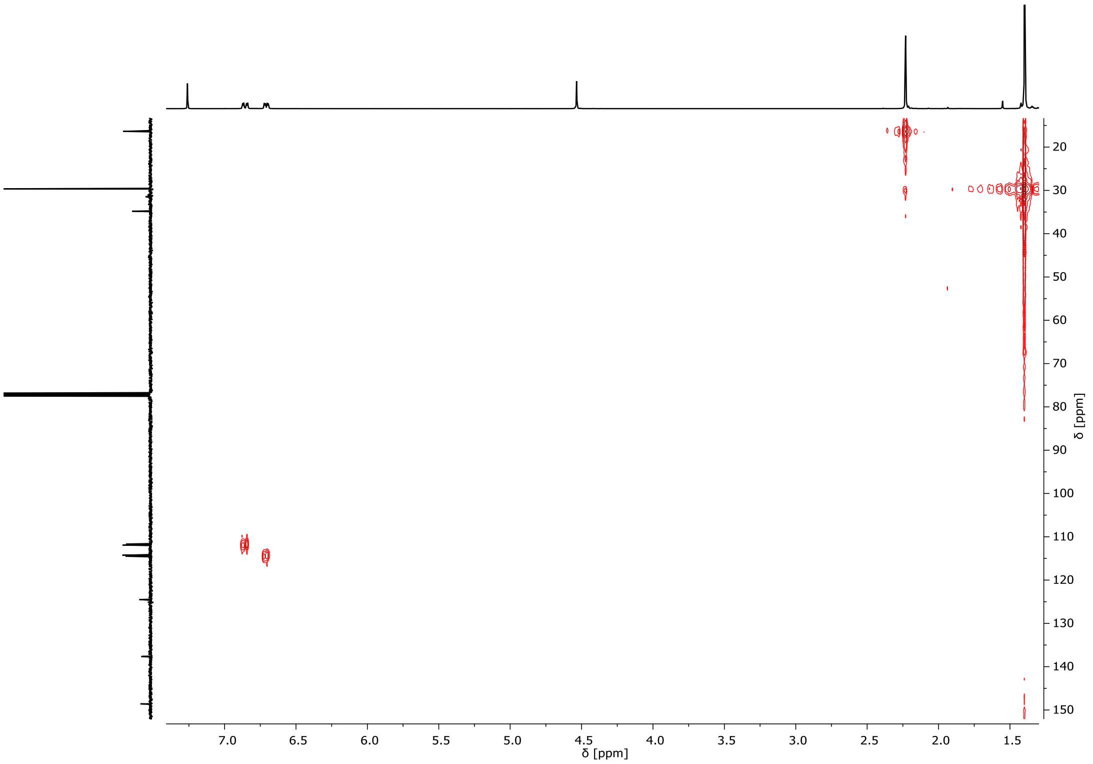

Figure S131 400 MHz ^{1}H-^{13}C Heteronuclear Single Quantum Coherence (HSQC) spectrum of **12** in $CDCl_3$.

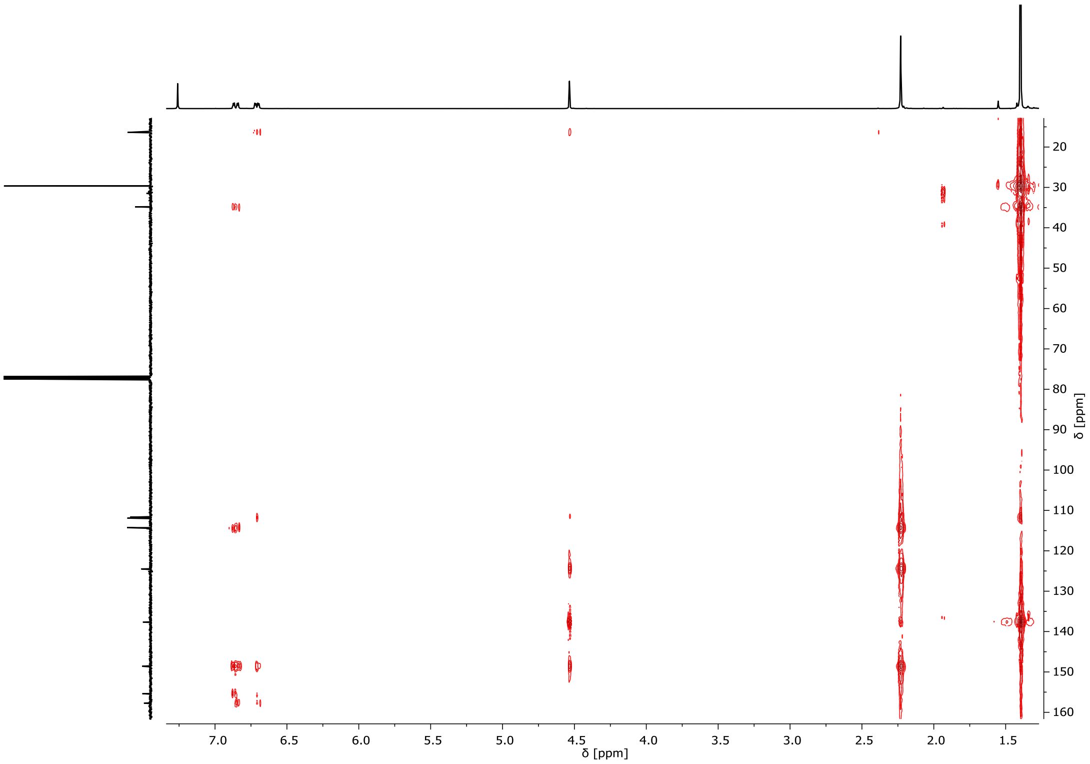

Figure S132 400 MHz ^{1}H-^{13}C Heteronuclear Multiple Bond Correlation (HMBC) spectrum of **12** in $CDCl_3$.

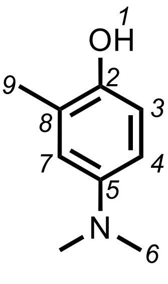

19

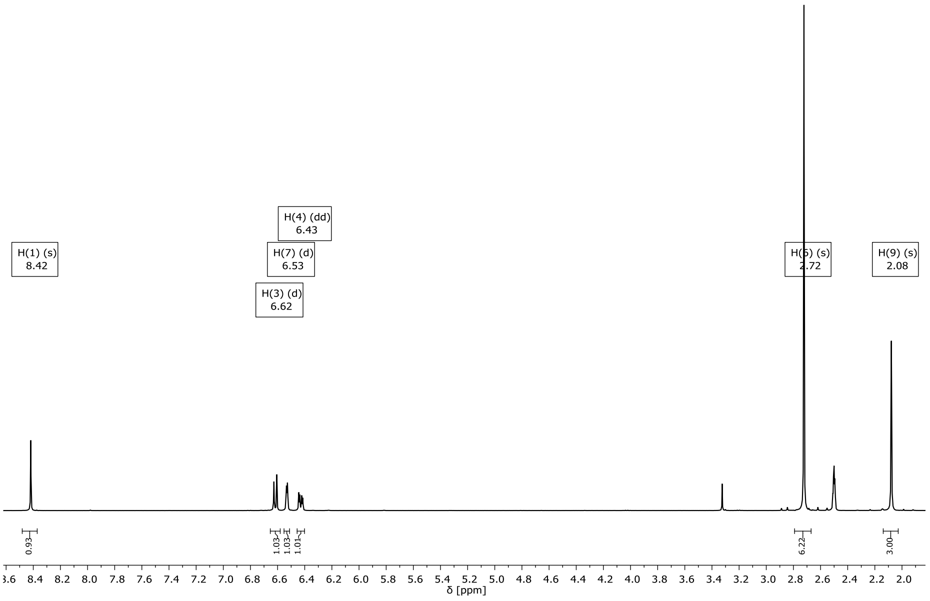

Figure S133 400 MHz ^{1}H-NMR of **19** in d_6-DMSO.

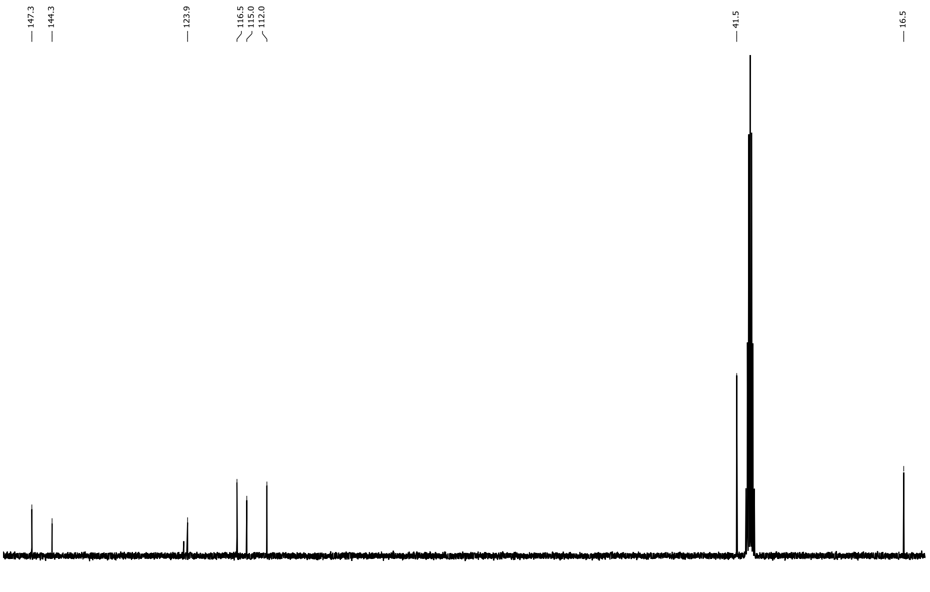

Figure S134 101 MHz ^{13}C-NMR of **19** in d_6-DMSO (artifact at 125.0 ppm).

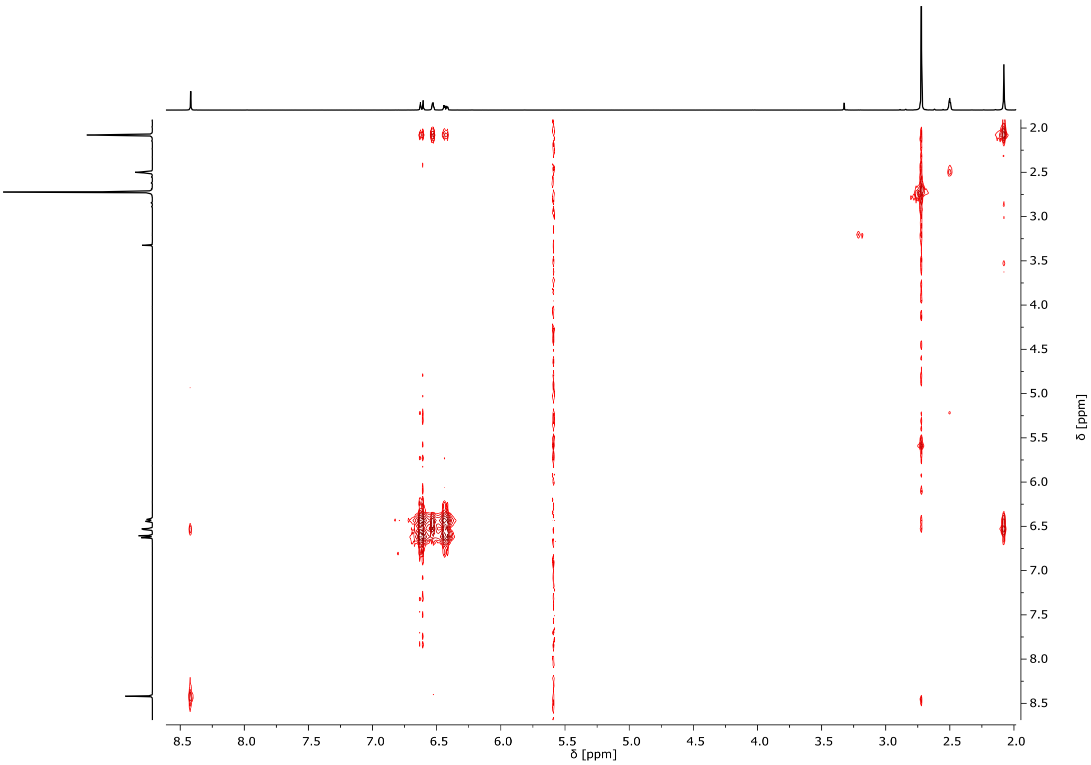

Figure S135 400 MHz ^1H-^1H COSY spectrum of **19** in d_6-DMSO.

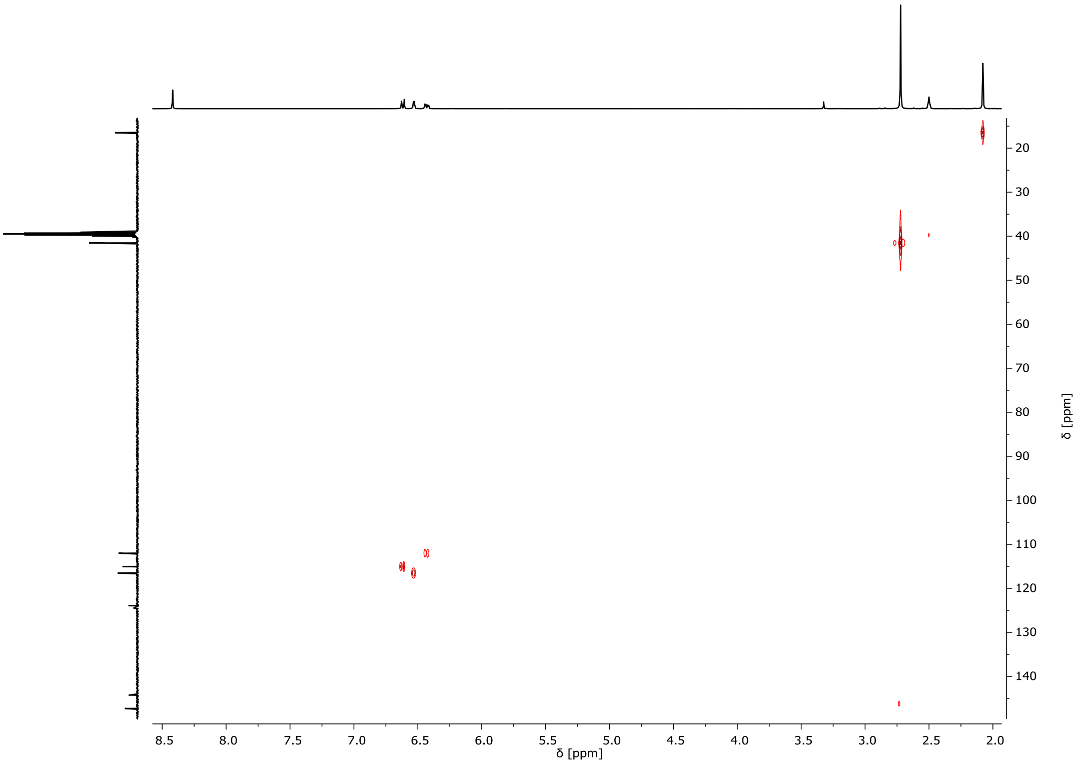

Figure S136 400 MHz ^1H-^{13}C Heteronuclear Single Quantum Coherence (HSQC) spectrum of **19** in d_6-DMSO.

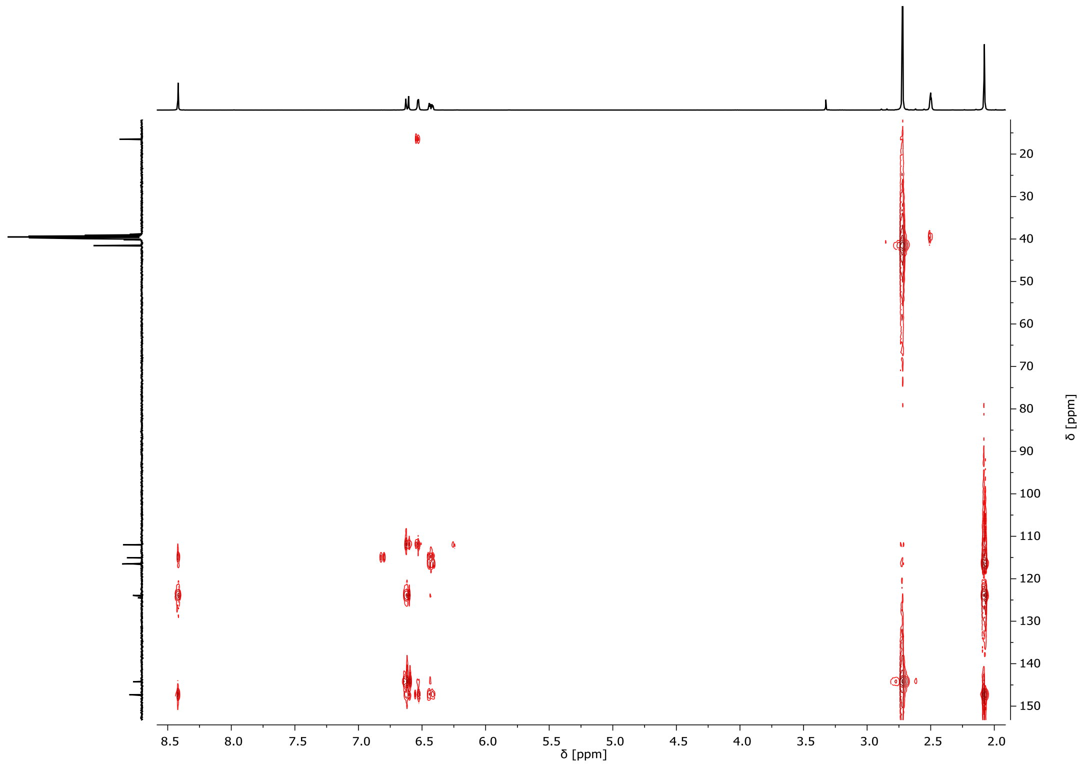

Figure S137 400 MHz ^{1}H-[13]C Heteronuclear Multiple Bond Correlation (HMBC) spectrum of **19** in d_6-DMSO.

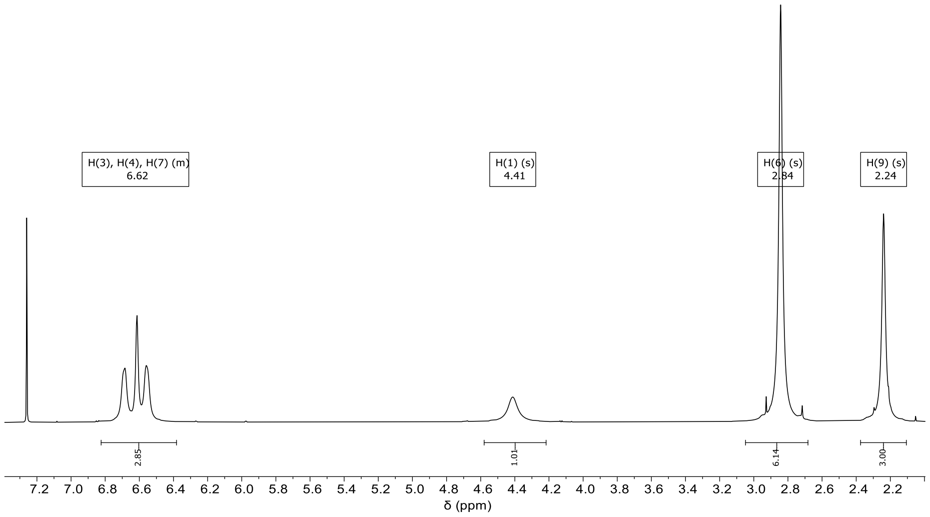

Figure S138 600 MHz ^{1}H-NMR of **19** in $CDCl_3$.

^{1}H-NMR of reference compounds 13, 15-18

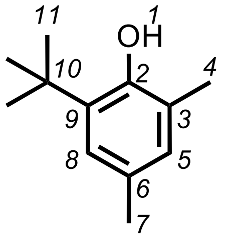

13

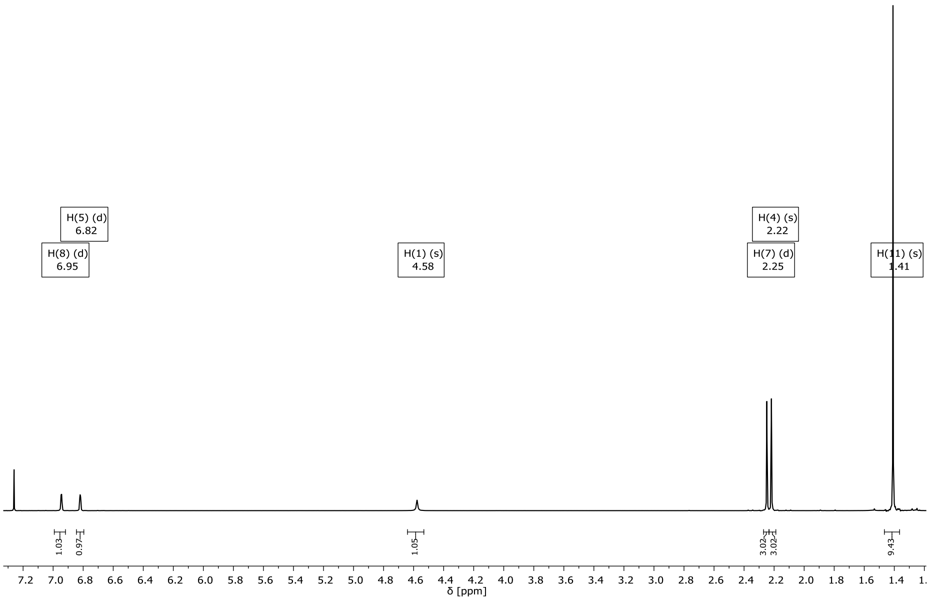

Figure S139 500 MHz ^{1}H-NMR of **13** in $CDCl_3$.

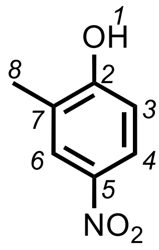

15

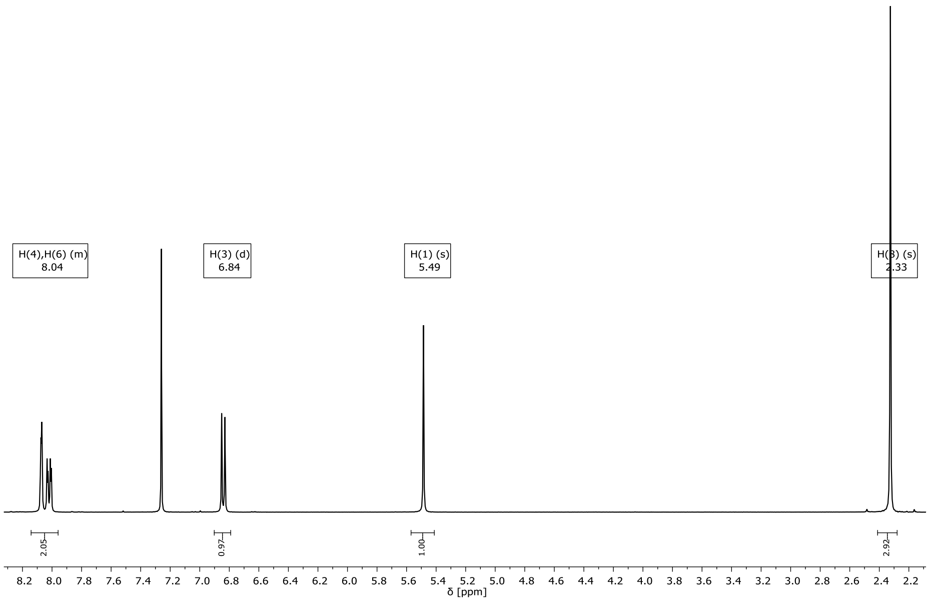

Figure S140 400 MHz ^{1}H-NMR of **15** in $CDCl_3$.

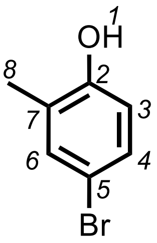

16

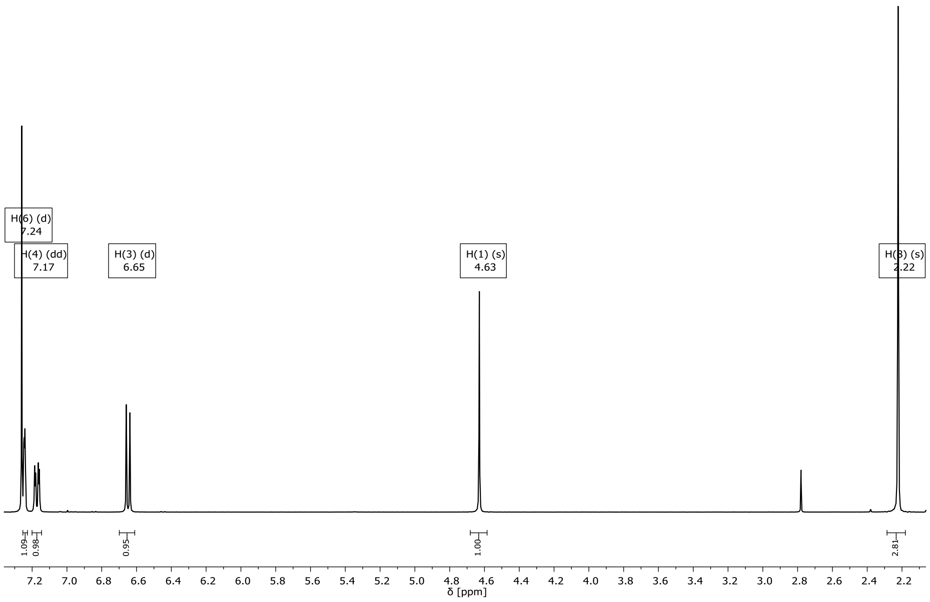

Figure S141 400 MHz ^{1}H-NMR of **16** in $CDCl_3$.

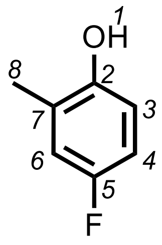

17

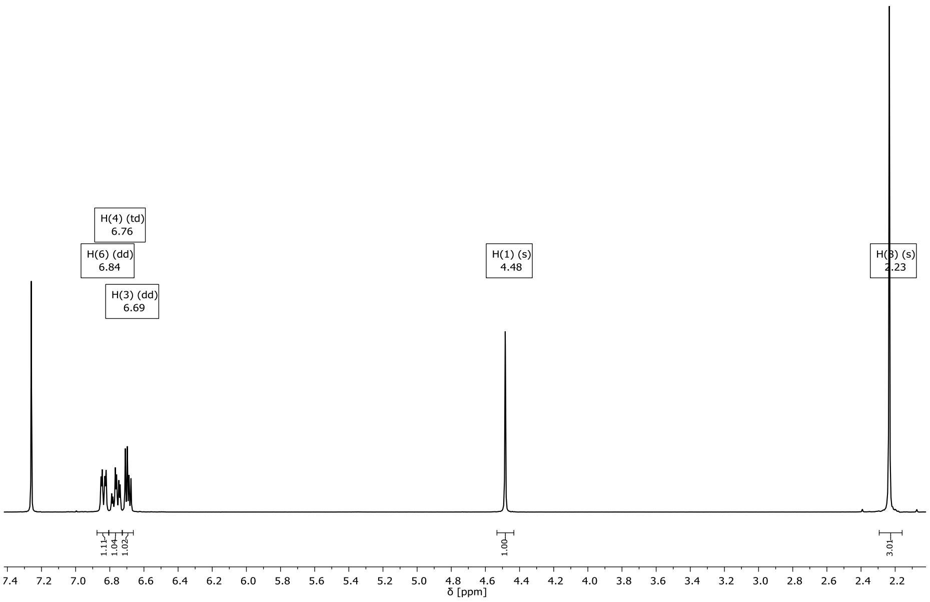

Figure S142 400 MHz ^{1}H-NMR of **17** in $CDCl_3$.

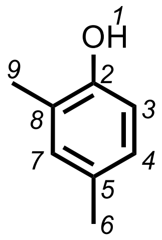

18

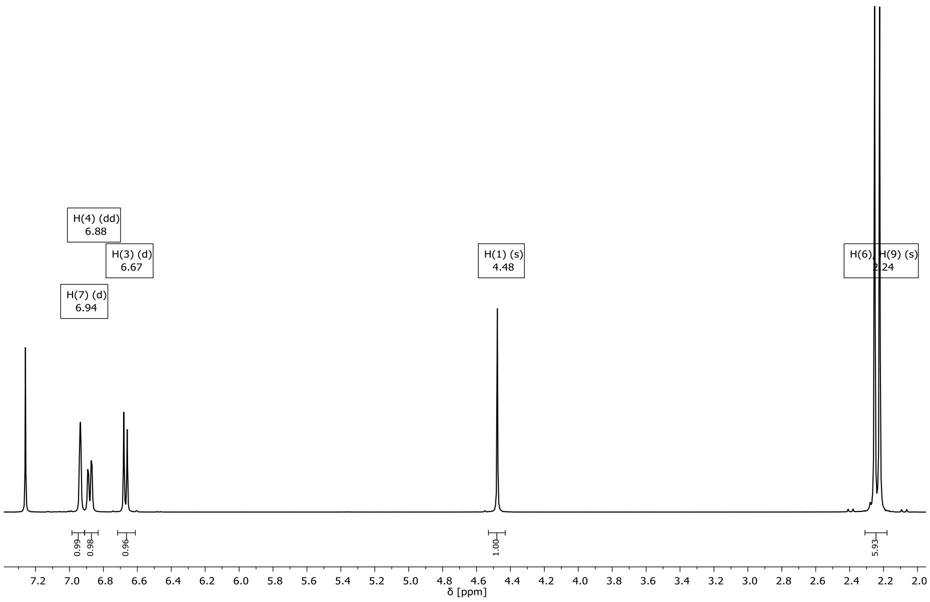

Figure S143 400 MHz ^{1}H-NMR of **18** in $CDCl_3$.

4. Single Crystal X-ray Diffraction

X-ray data were collected on a Bruker D8-QUEST diffractometer, equipped with an Incoatec IμS Cu microsource (λ = 1.5418 Å) and a PHOTON-III detector operating in shutterless mode. The crystal temperature was held at 180(2) K using an Oxford Cryosystems open-flow N_2 Cryostream. The control and processing software was Bruker *APEX4* (ver. 2021.4-0). Structures were solved using SHELXT[13] and refined using SHELXL.[14]

Structure 6: H atoms on the hydroxyl groups were located in the difference Fourier map and refined freely with isotropic displacement parameters.

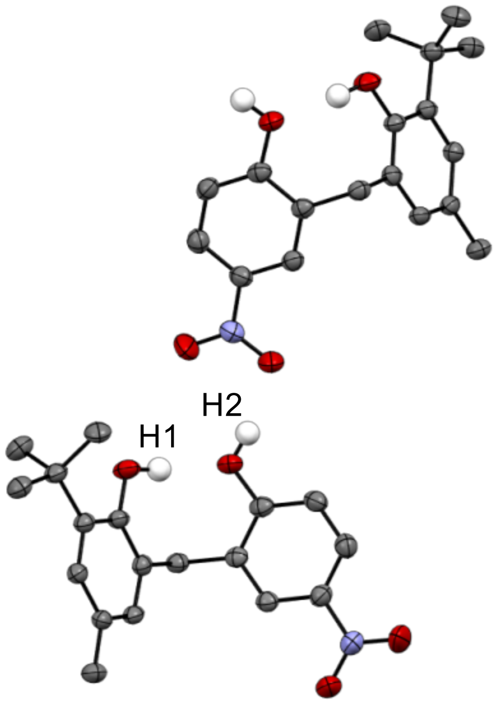

D	H	A	D–H (Å)	H···A (Å)	D···A (Å)	D–H···A (°)
O1	H1	O2	0.92(3)	1.91(3)	2.831(2)	173(3)
O2	H2	O3[a]	0.89(3)	1.89(3)	2.768(2)	168(3)

Symmetry code: (a) 2–x, 1/2+y, 3/2–z

Structure 7: The crystals were thin needles (min dimension = 10 μm) and diffraction at higher angle was weak. I/σ(I) falls below 3.0 around 1.0 Å. The data are integrated to 0.93 Å (where I/σ(I) = 1.5) and the precision of the structure is limited accordingly. The refinement is implemented as a 2-component twin, with twin law [1 0 0 / 0 -1 0 / 0 0 -1], corresponding to a 180° rotation around a*.

The crystal structure of **7** is isomorphous with the previously published **4**.[20]

The H atoms of the OH groups could not be located confidently from the difference Fourier map. They are placed geometrically and refined as riding on the parent O atom. The molecules form 1-D H-bonded chains running along the crystallographic a axis. The O–H···O chains could conceivably run in either direction along the a axis, such that either of the OH groups in the molecule could act as the intramolecular donor. The choice to include OH_b acting as the intramolecular donor is consistent with that established confidently for the isomorphous compound **4** (CSD: WEPTOE).[20]

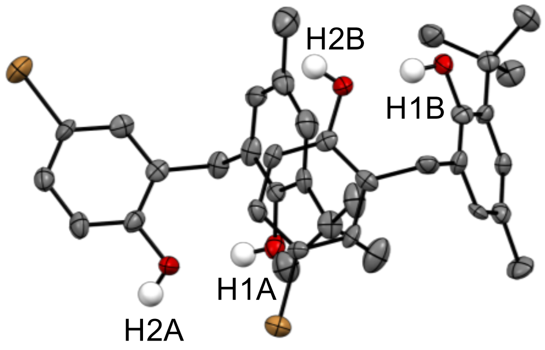

D	H	A	D–H (Å)	H···A (Å)	D···A (Å)	D–H···A (°)
O1A	H1A	O2A	0.84	1.90	2.737(8)	174.2
O2A	H2A	O1B^a	0.84	1.90	2.728(8)	166.8
O1B	H1B	O2B	0.84	1.89	2.735(8)	179.4
O2B	H2B	O1A^b	0.84	1.90	2.735(8)	179.9

Symmetry code: (a) 1+x, 1/2–y, 1/2+z; (b) x, 1/2–y, –1/2+z

Structure 8: H atoms on the hydroxyl groups were located in the difference Fourier map and refined with isotropic displacement parameters, with O–H restrained to 0.84(1) Å. 1-D H-bond chains in the structure are directly comparable to those in **4** and **7**. The firm identification of OH_b as the intramolecular donor within these chains adds further evidence for the assignment chosen in **7**.

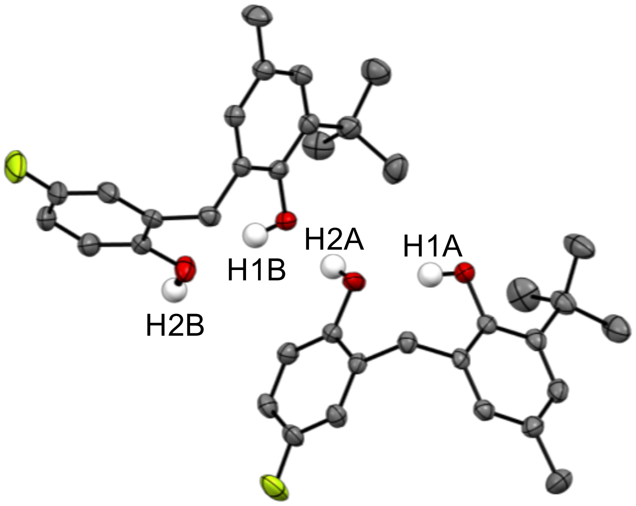

D	H	A	D–H (Å)	H···A (Å)	D···A (Å)	D–H···A (°)
O1A	H1A	O2A	0.834(13)	1.867(13)	2.701(2)	178(3)
O2A	H2A	O1B	0.842(13)	1.876(14)	2.705(2)	168(3)
O1B	H1B	O2B	0.838(13)	1.892(13)	2.729(2)	177(3)
O2B	H2B	O1A[a]	0.838(13)	1.880(13)	2.713(2)	172(3)

Symmetry code: (a) x, y, –1+z

Structure 25: H atoms on the hydroxyl groups were located in the difference Fourier map and refined freely with isotropic displacement parameters.

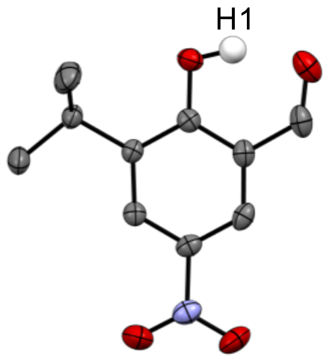

D	H	A	D–H (Å)	H···A (Å)	D···A (Å)	D–H···A (°)
O1	H1	O2	0.97(3)	1.70(3)	2.5991(17)	154(2)

Structure 27: H atoms on the hydroxyl groups were located in the difference Fourier map and refined freely with isotropic displacement parameters.

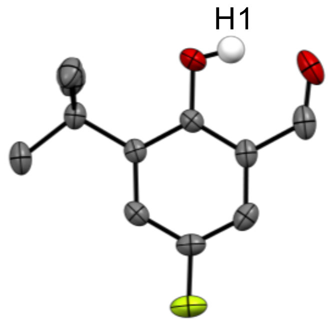

D	H	A	D–H (Å)	H···A (Å)	D···A (Å)	D–H···A (°)
O1	H1	O2	0.87(3)	1.80(3)	2.6025(17)	153(2)

Table S1 Summary of the crystal and refinement details.

	**6**	**7**	**8**
CCDC number	2393239	2393240	2393241
Cambridge data number	CH_B2_0035	CH_B1_0062	CH_B1_0053
Chemical formula	$C_{18}H_{21}NO_4$	$C_{18}H_{21}BrO_2$	$C_{18}H_{21}FO_2$
Formula weight	315.36	349.26	288.35
Temperature / K	180(2)	180(2)	180(2)
Crystal system	orthorhombic	monoclinic	orthorhombic
Space group	P b c a	P 2_1/c	P n a 2_1
a / Å	7.1487(2)	8.9098(7)	22.1057(5)
b / Å	16.3795(5)	22.0225(18)	17.2181(4)
c / Å	27.7255(7)	16.8396(14)	8.6672(2)
alpha / °	90	90	90
beta / °	90	90.031(4)	90
gamma / °	90	90	90
Unit-cell volume / $Å^3$	3246.44(16)	3304.2(5)	3298.89(13)
Z	8	8	8
Calc. density / g cm^{-3}	1.290	1.404	1.161
F(000)	1344	1440	1232
Radiation type	Cu Kα	Cu Kα	Cu Kα
Absorption coefficient / mm^{-1}	0.744	3.399	0.664
Crystal size / mm^3	0.18 x 0.16 x 0.02	0.10 x 0.01 x 0.01	0.24 x 0.10 x 0.08
2-Theta range / °	6.38-133.36	5.25-112.24	6.51-133.25
Completeness to max 2-theta	0.999	0.996	0.999
No. of reflections measured	40017	24860	31917
No. of independent reflections	2875	4331	5004
R(int)	0.1139	0.1308	0.0385
No. parameters / restraints	220 / 0	388 / 0	403 / 5
Final R1 values ($I > 2\sigma(I)$)	0.0451	0.0560	0.0328
Final wR(F^2) values (all data)	0.1244	0.1378	0.0882
Goodness-of-fit on F^2	1.038	1.003	1.062
Largest difference peak & hole / e $Å^{-3}$	0.257, -0.208	0.434, -1.028	0.115, -0.136
Flack parameter			-0.11(8)

	**25**	**27**
CCDC number	2393238	2393237
Cambridge data number	CH_B1_0060	CH_B2_0046
Chemical formula	$C_{11}H_{13}NO_4$	$C_{11}H_{13}FO_2$
Formula weight	223.22	196.21
Temperature / K	180(2)	180(2)
Crystal system	orthorhombic	monoclinic
Space group	P b c a	P 2_1/m
a / Å	9.8547(3)	8.4301(6)
b / Å	12.0275(3)	6.6538(4)
c / Å	18.1774(5)	8.8774(6)
alpha / °	90	90
beta / °	90	94.239(3)
gamma / °	90	90
Unit-cell volume / $Å^3$	2154.52(10)	496.59(6)
Z	8	2
Calc. density / g cm^{-3}	1.376	1.312
F(000)	944	208
Radiation type	Cu Kα	Cu Kα
Absorption coefficient / mm^{-1}	0.886	0.847
Crystal size / mm^3	0.10 x 0.08 x 0.08	0.20 x 0.14 x 0.08
2-Theta range / °	12.59-133.28	9.99-132.97
Completeness to max 2-theta	0.997	0.993
No. of reflections measured	19848	6978
No. of independent reflections	1901	943
R(int)	0.0452	0.0280
No. parameters / restraints	152 / 0	87 / 0
Final R1 values ($I > 2\sigma(I)$)	0.0417	0.0332
Final wR(F^2) values (all data)	0.1252	0.0910
Goodness-of-fit on F^2	1.065	1.113
Largest difference peak & hole / e $Å^{-3}$	0.537, -0.205	0.224, -0.151

5. NMR Experiments

^{1}H-NMR Titration – General Procedure

A diluted solution of the host (5 mL) in *n*-octane is prepared from the stock solution of the receptor in *n*-octane. 600 μL of the host solution is titrated with a solution of the guest (G) containing also the host at the same concentration in *n*-octane. A ^{1}H-NMR spectrum with WET[15] or presat[16] solvent suppression is recorded for every point of the titration on a 500 MHz spectrometer. The chemical shifts of the protons of the host are monitored upon addition of various concentrations of guest. The observed chemical shift (δ_{obs}, ppm, Equation 1) is a weighted average of the chemical shifts of the free host (δ_H, ppm) and the host-guest complex ($\delta_{H\cdot G}$, ppm):

$$\delta_{obs} = \delta_H X_H + \delta_{H\cdot G} X_{H\cdot G} \qquad (1)$$

where X_H and $X_{H\cdot G}$ are the mole fractions of H (X_H = [H]/[H]$_0$) and of H·G ($X_{H\cdot G}$ = [H·G]/[H]$_0$), respectively. [H]$_0$ is the initial concentration of H ([H]$_0$=[H·G]+ [H]) and [G]$_0$ is the initial concentration of G ([G]$_0$=[H·G]+ [G]). Since X_H=1 - $X_{H\cdot G}$, equation (1) can be rearranged:

$$\delta_{obs} = \delta_H(1 - X_{H\cdot G}) + \delta_{H\cdot G} X_{H\cdot G} \qquad (2)$$

$$\frac{\delta_{obs}-\delta_H}{\delta_{HG}-\delta_H} = X_{H\cdot G} = \frac{[HG]}{[H]_0} \qquad (3)$$

Given that the association constant K_a for the 1:1 H·G complex is [H·G]/[H][G] and [H]$_0$=[H·G]+ [H], equation (3) can be written as

$$\frac{\delta_{obs}-\delta_H}{\delta_{HG}-\delta_H} = \frac{K_a[G]}{1+K_a[G]} \qquad (4)$$

and [G] can be determined using equation (5) by making iteratively guesses of K_a and solving for [G] until the theoretical isotherm matches the experimental data:

$$K_a[G]^2 + (K_a[H]_0 - K_a[G]_0 + 1)[G] - [G]_0 = 0 \qquad (5)$$

A Microsoft Excel spreadsheet with purpose-written VBA macros was used to solve equations (4) and (5).[17]

Each titration was repeated three times, fitted with equations (4) and (5), and an average value of the association constant along with its standard error (with 95% confidence) is reported in Table 2.

Donor	Acceptor
	Quin
**5**	$(1.0 \pm 0.1)x10^{4}$ M^{-1}
**9**	$(7.0 \pm 0.5)x10^{3}$ M^{-1}
**14**	13 ± 4 M^{-1}
**19**	$(1.4 \pm 0.2)x10^{2}$ M^{-1}

Table S2 Association constants (M^{-1}) for formation of 1:1 complexes measured by NMR spectroscopy titrations in *n*-octane at 298 K. Errors are the standard error of the mean of three independent experiments.

^{1}H-NMR Dilution – General Procedure

A diluted solution of the host (2 mL) in *n*-octane is prepared from the stock solution of the receptor in *n*-octane. Increasing volumes of the host solution were added to 600 μL of *n*-octane. Alternatively, to 600 μL of a diluted solution of the host, increasing volumes of *n*-octane were added. A ^{1}H-NMR spectrum with WET[17] or presat[18] solvent suppression is recorded after each addition.

Molecule 1

Dilution Experiment of 1 in *n*-octane

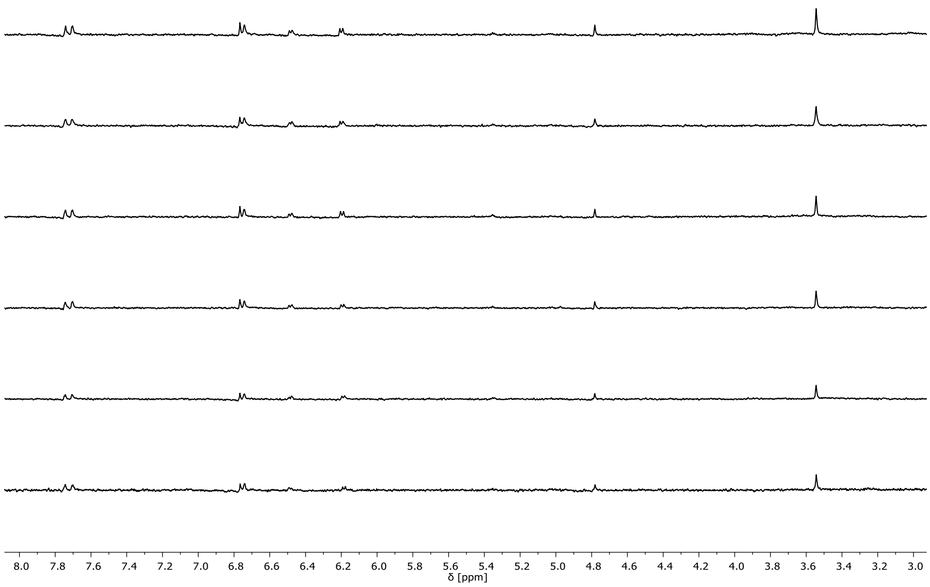

Figure S144 NMR dilution of **1** in *n*-octane (500 MHz ^{1}H-NMR spectra with WET solvent suppression, at the following concentrations of **1** (from bottom to top) 0.086 mM, 0.107 mM, 0.139 mM, 0.155 mM, 0.173 mM, 0.179 mM, 0.183 mM).

Titration of 1 with quinuclidine in *n*-octane

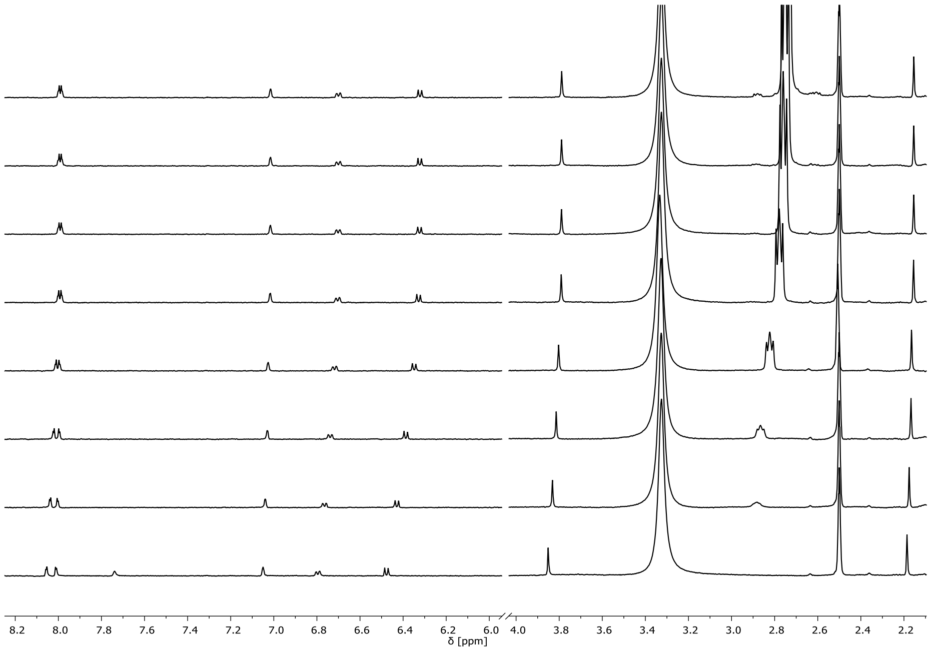

Figure S145 500 MHz ^{1}H-NMR titration of **1** (0.142 mM) with quinuclidine in *n*-octane (500 MHz ^{1}H-NMR spectra with WET solvent suppression, H_2O at 3.32 ppm; DMSO at 2.50 ppm).

Molecule 2

Dilution Experiment of 2 in *n*-octane

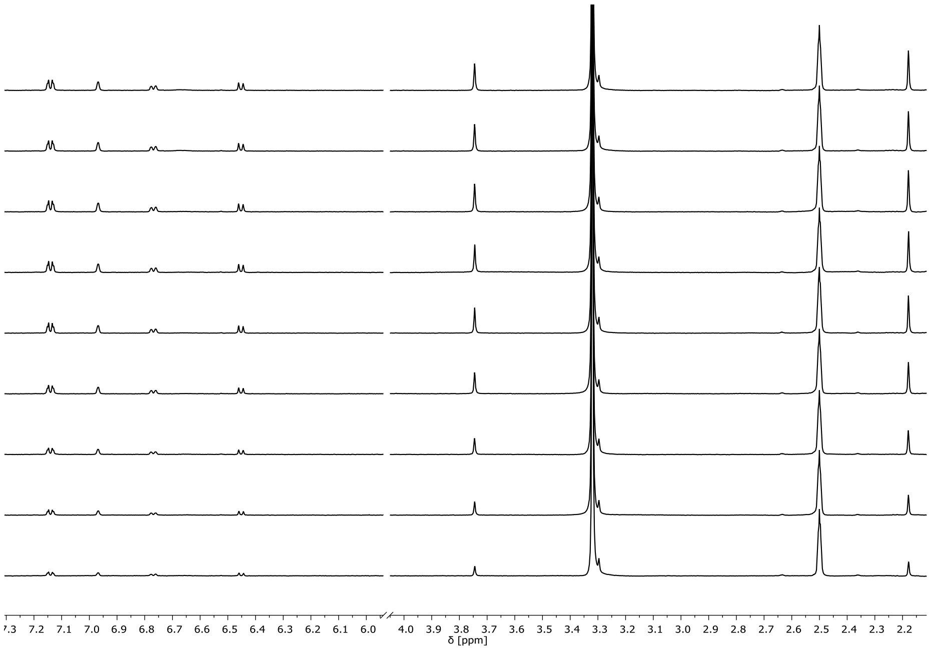

Figure S146 NMR dilution of **2** in *n*-octane (500 MHz ^{1}H-NMR spectra with WET solvent suppression, at the following concentrations of **2** (from bottom to top) 0073 mM, 0.101 mM, 0.125 mM, 0.163 mM, 0.181 mM, 0.202 mM, 0.209 mM, 0.214 mM).

Titration of 2 with quinuclidine in *n*-octane

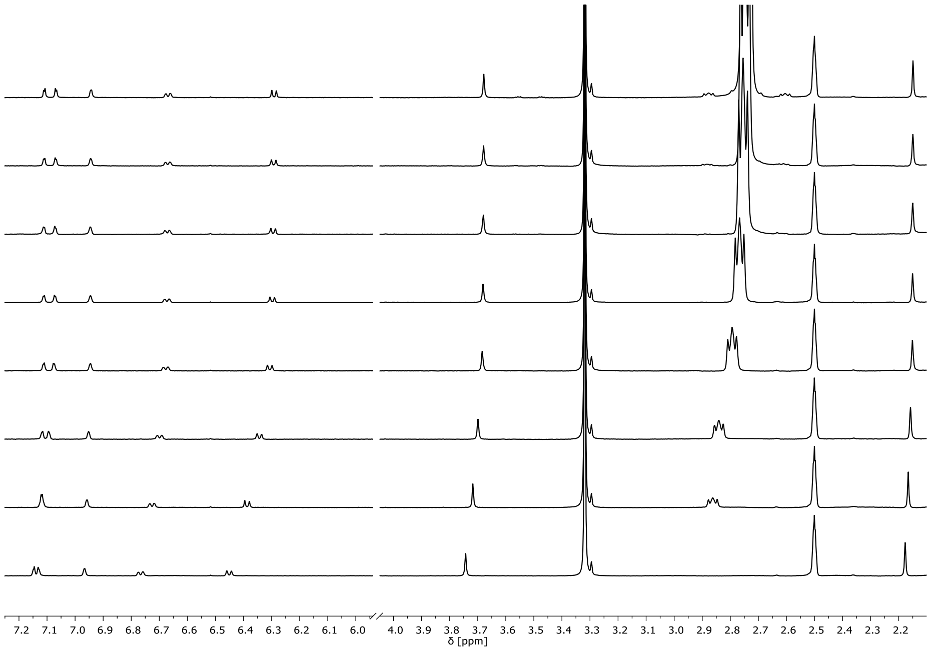

Figure S147 500 MHz ^{1}H-NMR titration of **2** (0.214 mM) with quinuclidine in *n*-octane (500 MHz ^{1}H-NMR spectra with WET solvent suppression, H_2O at 3.32 ppm; DMSO at 2.50 ppm).

Molecule 3

Dilution Experiment of 3 in *n*-octane

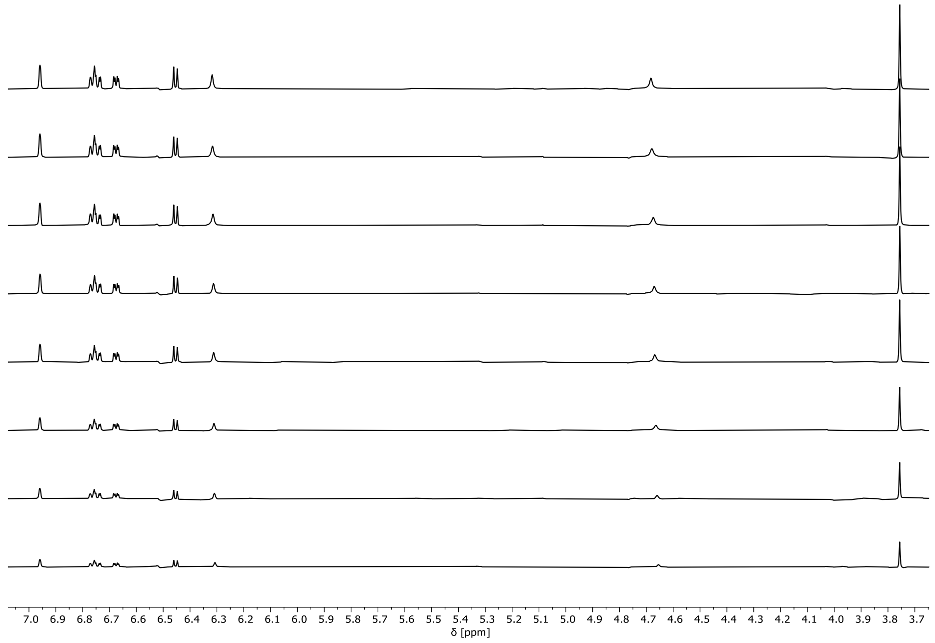

Figure S148 NMR dilution of **3** in *n*-octane (600 MHz ^{1}H-NMR spectra with WET solvent suppression, at the following concentrations of **3** (from bottom to top) 0.069 mM, 0.094 mM, 0.117 mM, 0.152 mM, 0.169 mM, 0.189 mM, 0.195 mM, 0.200 mM).

Titration of 3 with quinuclidine in *n*-octane

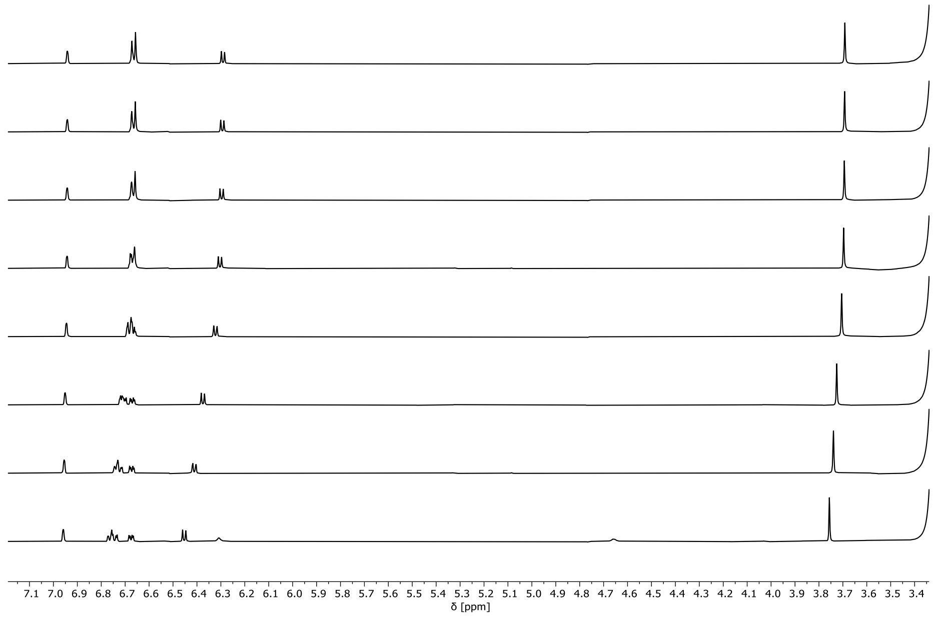

Figure S149 600 MHz ^{1}H-NMR titration of **3** (0.200 mM) with quinuclidine in *n*-octane (600 MHz ^{1}H-NMR spectra with WET solvent suppression, H_2O at 3.32 ppm).

Molecule 5

Dilution Experiment of 5 in *n*-octane

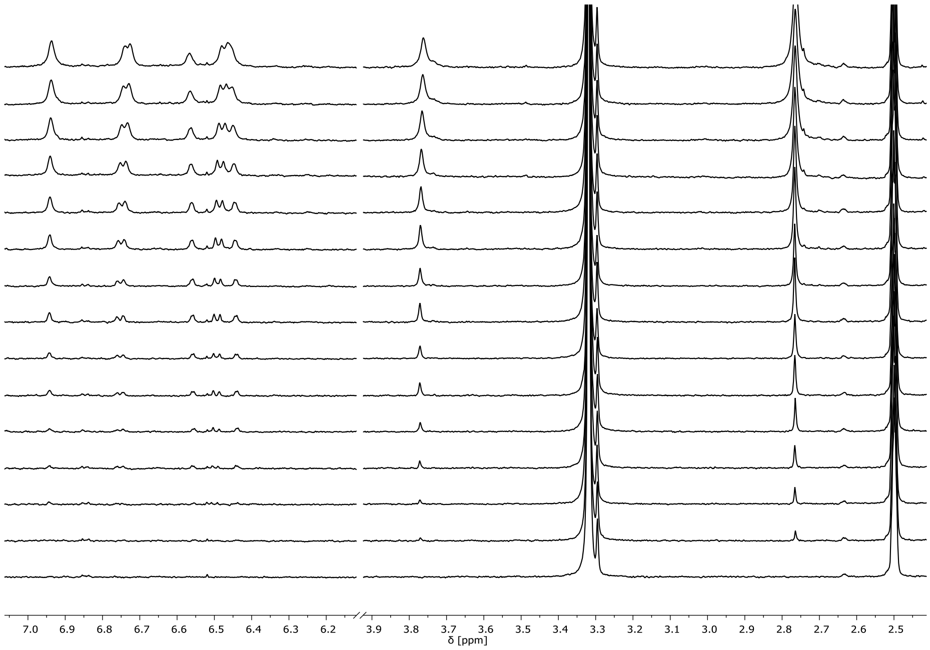

Figure S150 NMR dilution of **5** in *n*-octane (600 MHz ^{1}H-NMR spectra with presat solvent suppression, at the following concentrations of **5** (from bottom to top) 0 mM, 0.014 mM, 0.028 mM, 0.056 mM, 0.083 mM, 0.136 mM, 0.187 mM, 0.285 mM, 0.378 mM, 0.547 mM, 0.698 mM, .0.956 mM, 1.349 mM, 1.747 mM, 2.209 mM).

Titration of 5 with quinuclidine in *n*-octane

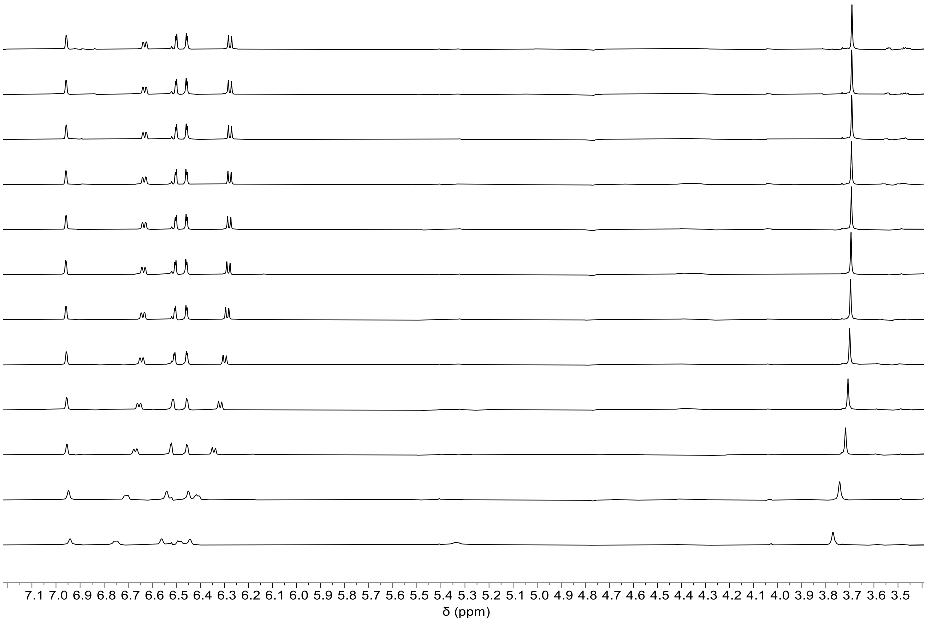

Figure S151 600 MHz ^{1}H-NMR titration of **5** (0.200 mM) with quinuclidine in *n*-octane (600 MHz ^{1}H-NMR spectra with WET solvent suppression).

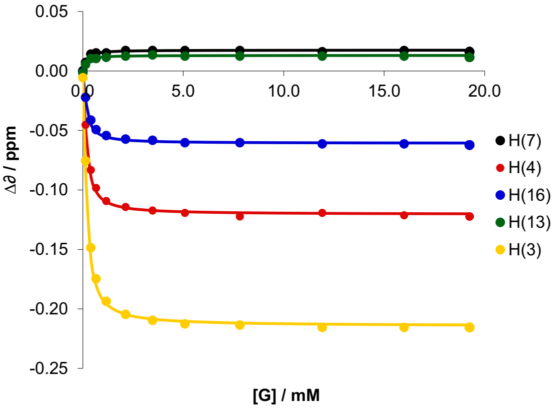

Figure S152 Fitting of the data from the NMR titration of **5** with quinuclidine in *n*-octane (Figure S151) with a 1:1 binding model.

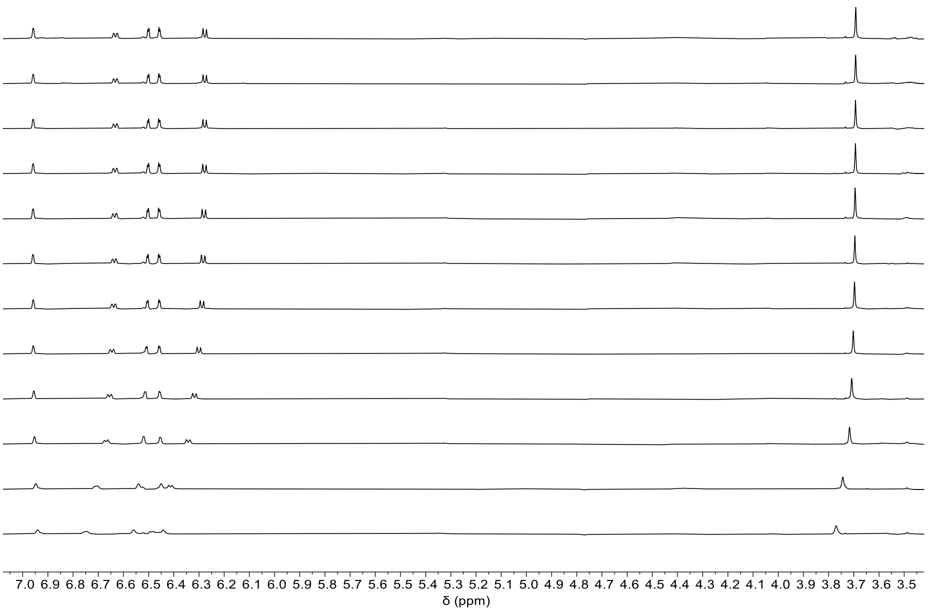

Figure S153 600 MHz ^{1}H-NMR titration of **5** (0.200 mM) with quinuclidine in *n*-octane (600 MHz ^{1}H-NMR spectra with WET solvent suppression).

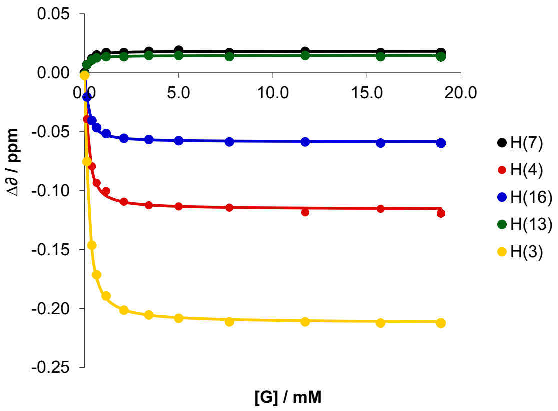

Figure S154 Fitting of the data from the NMR titration of **5** with quinuclidine in *n*-octane (Figure S153) with a 1:1 binding model.

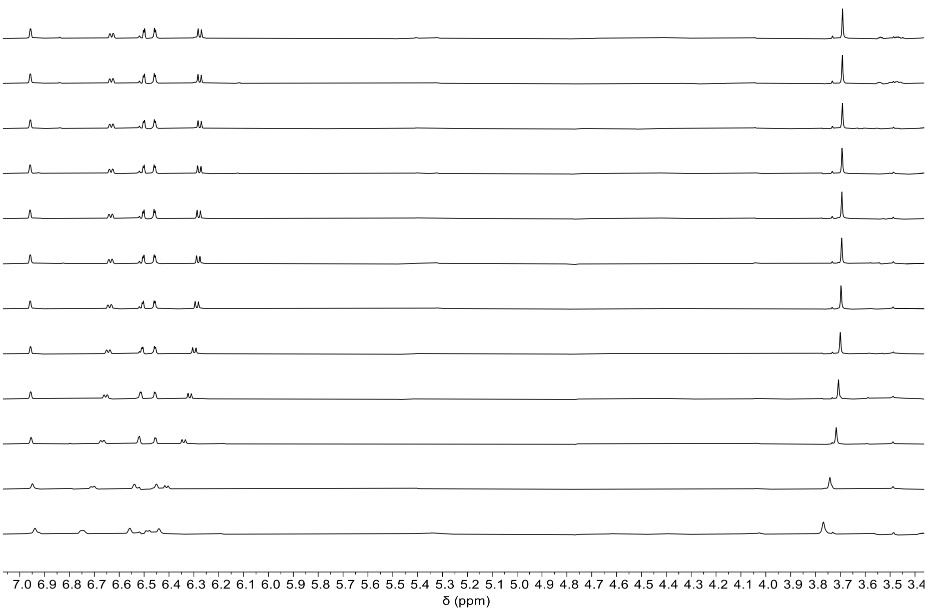

Figure S155 600 MHz ^{1}H-NMR titration of **5** (0.200 mM) with quinuclidine in *n*-octane (600 MHz ^{1}H-NMR spectra with WET solvent suppression).

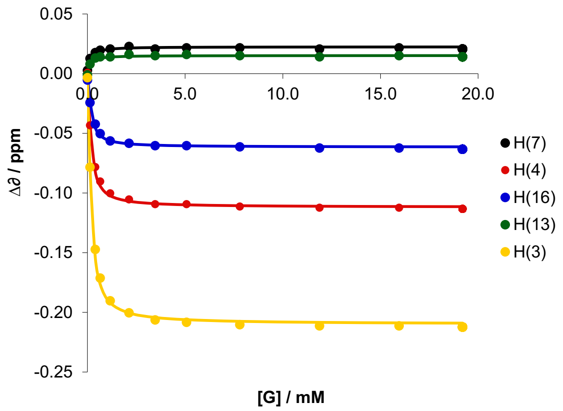

Figure S156 Fitting of the data from the NMR titration of **5** with quinuclidine in *n*-octane (Figure S155) with a 1:1 binding model.

Molecule 6

Dilution Experiment of 6 in *n*-octane

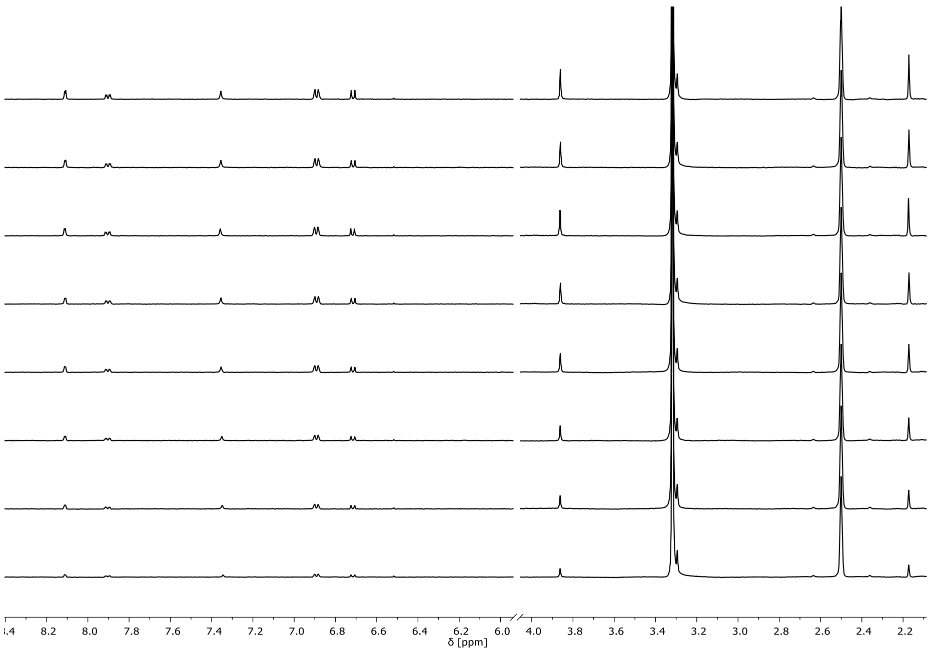

Figure S157 NMR dilution of **6** in *n*-octane (500 MHz ^{1}H-NMR spectra with WET solvent suppression, at the following concentrations of **6** (from bottom to top) 0.052 mM, 0.072 mM, 0.089 mM, 0.115 mM, 0.128 mM, 0.144 mM, 0.148 mM, 0.152 mM).

Titrations of 6 with quinuclidine in *n*-octane

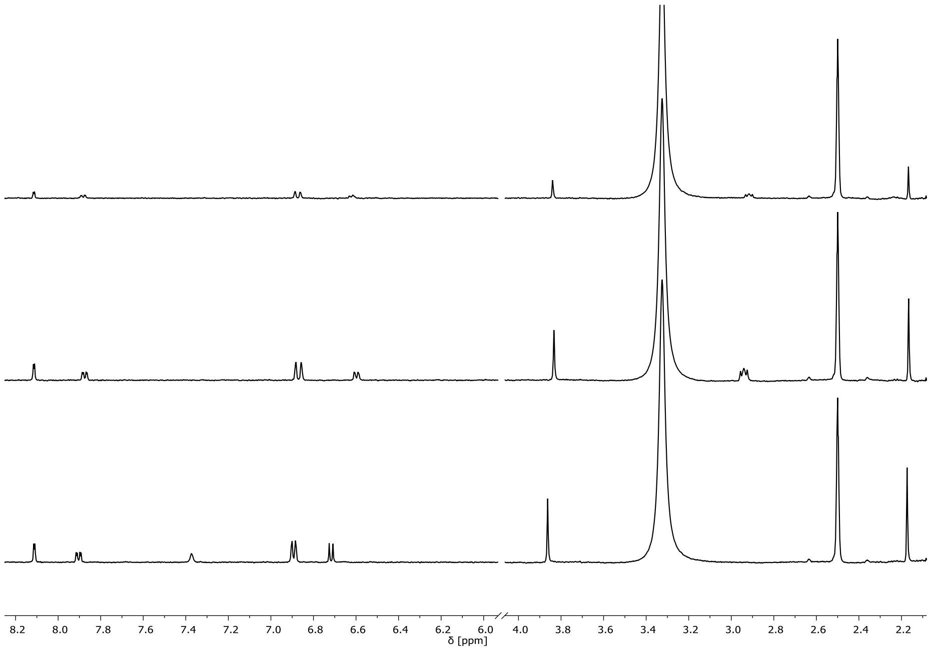

Figure S158 500 MHz ^{1}H-NMR titration of **6** (0.152 mM) with quinuclidine in *n*-octane (500 MHz ^{1}H-NMR spectra with WET solvent suppression, H_2O at 3.32 ppm; DMSO at 2.50 ppm).

Molecule 7

Dilution Experiment of 7 in *n*-octane

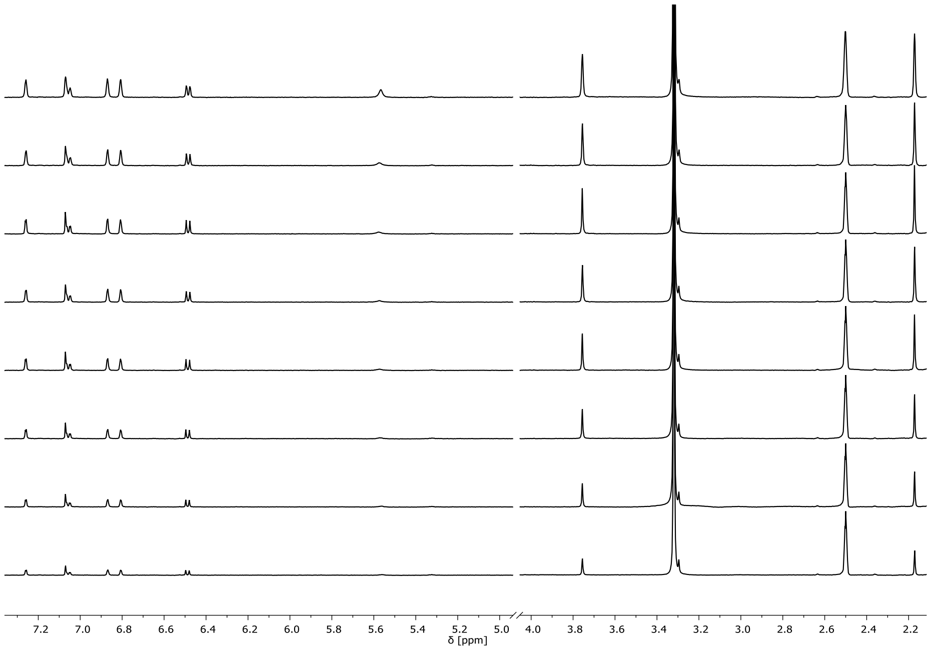

Figure S159 NMR dilution of **7** in *n*-octane (500 MHz ^{1}H-NMR spectra with WET solvent suppression, at the following concentrations of **7** (from bottom to top) 0.091 mM, 0.125 mM, 0.154 mM, 0.201 mM, 0.224 mM, 0.250 mM, 0.259 mM, 0.265 mM).

Titrations of 7 with quinuclidine in *n*-octane

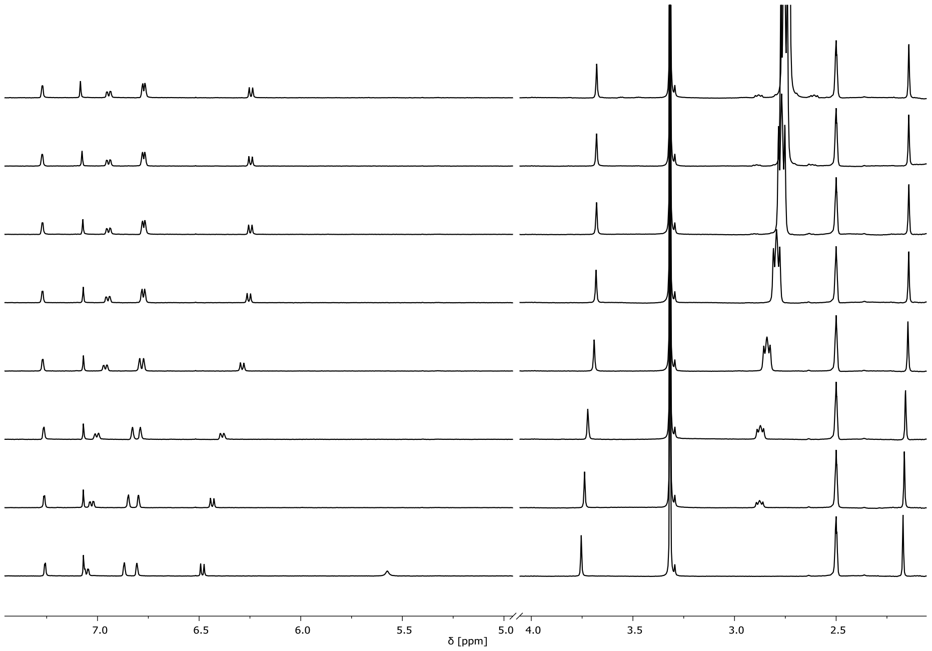

Figure S160 500 MHz ^{1}H-NMR titration of **7** (0.265 mM) with quinuclidine in *n*-octane (500 MHz ^{1}H-NMR spectra with WET solvent suppression, H_2O at 3.32 ppm; DMSO at 2.50 ppm).

Molecule 8

Dilution Experiment of 8 in *n*-octane

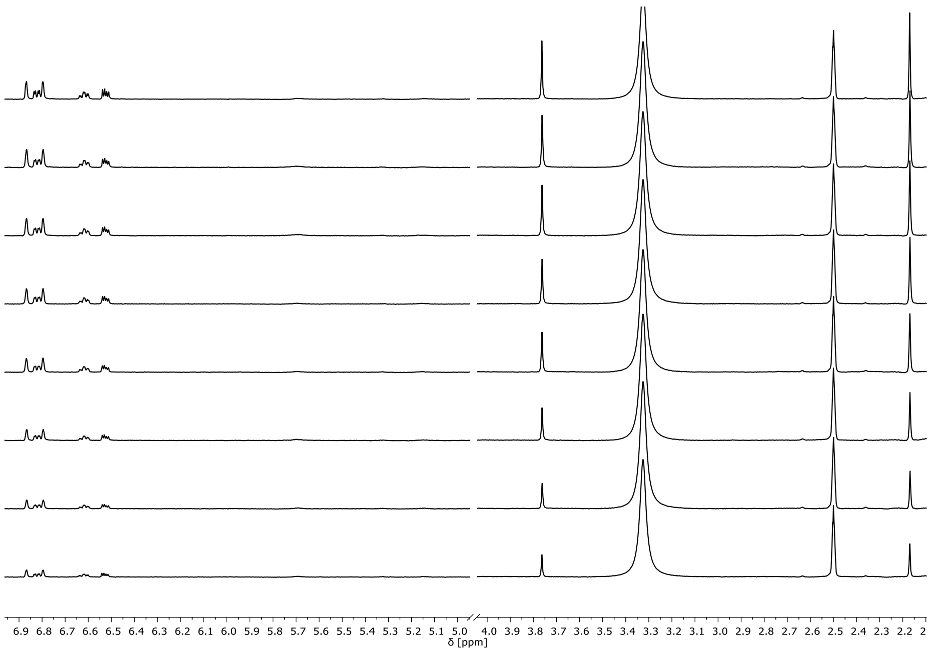

Figure S161 NMR dilution of **8** in *n*-octane (500 MHz ^{1}H-NMR spectra with WET solvent suppression, at the following concentrations of **8** (from bottom to top) 0.106 mM, 0.146 mM, 0.179 mM, 0.234 mM, 0.260 mM, 0.291 mM, 0.300 mM, 0.308 mM).

Titrations of 8 with quinuclidine in *n*-octane

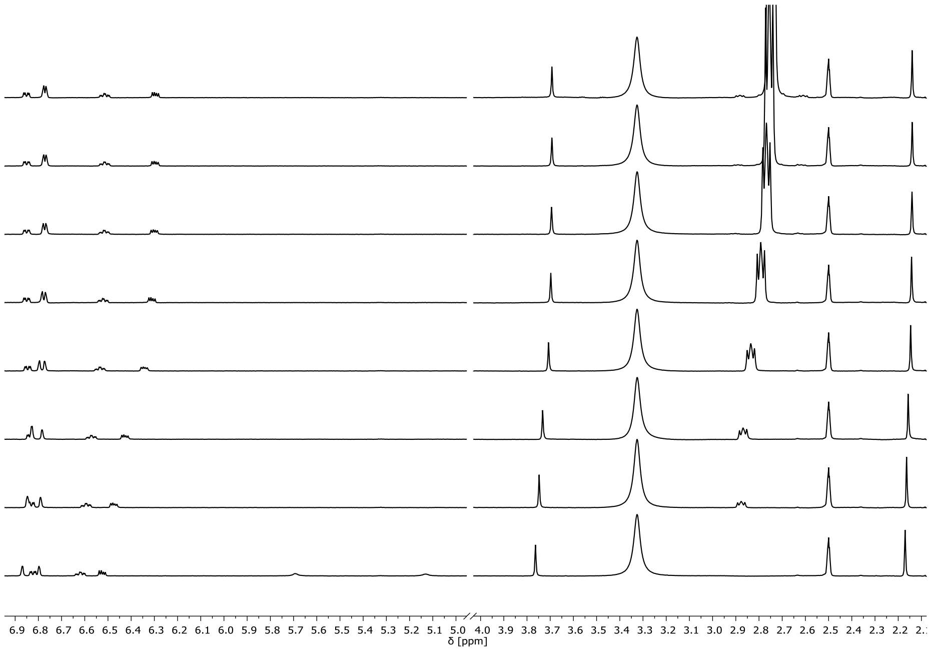

Figure S162 500 MHz ^{1}H-NMR titration of **8** (0.308 mM) with quinuclidine in *n*-octane (500 MHz ^{1}H-NMR spectra with WET solvent suppression, H_2O at 3.32 ppm; DMSO at 2.50 ppm).

Molecule 9

Dilution Experiment of 9 in *n*-octane

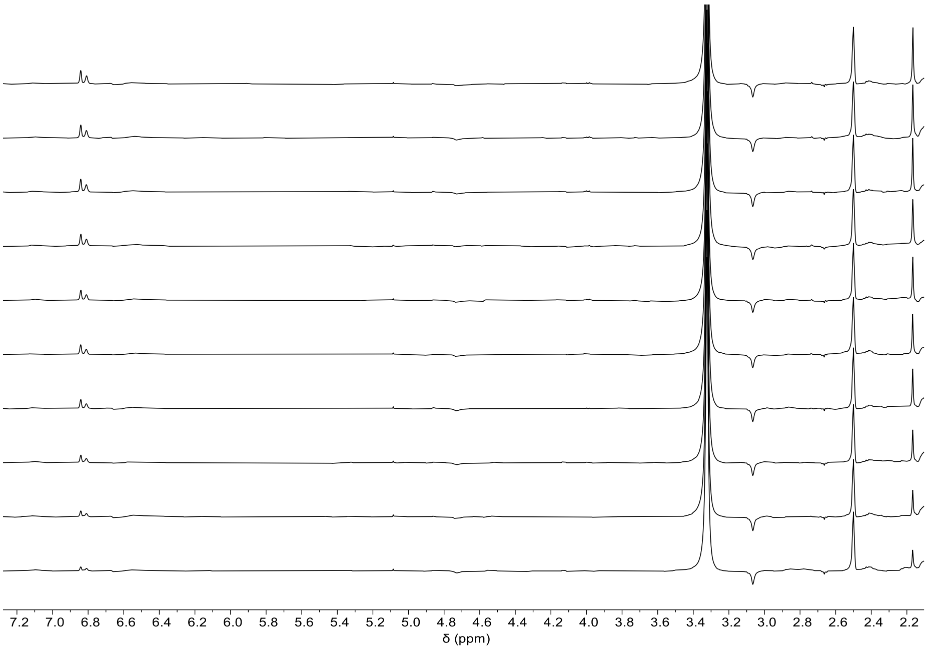

Figure S163 NMR dilution of **9** in *n*-octane (500 MHz ^{1}H-NMR spectra with WET solvent suppression, H_2O at 3.32 ppm; DMSO at 2.50 ppm, at the following concentrations of **9** (from bottom to top) 0.086 mM, 0.118 mM, 0.146 mM, 0.172 mM, 0.190 mM, 0.211 mM, 0.224 mM, 0.238 mM, 0.246 mM, 0.250 mM).

Titration of 9 with quinuclidine in *n*-octane

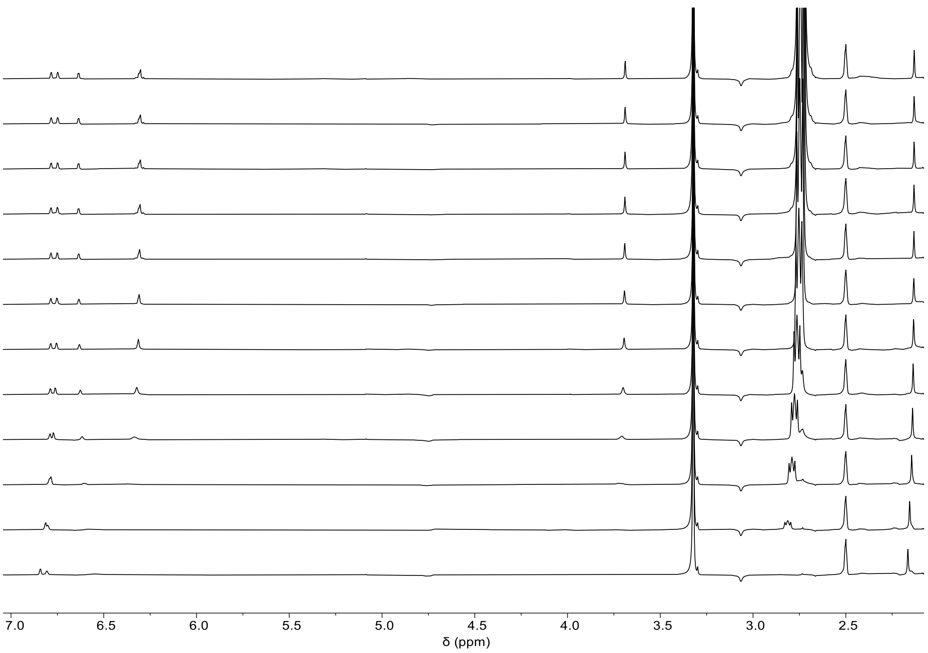

Figure S164 500 MHz ^{1}H-NMR titration of **9** (0.250 mM) with quinuclidine in *n*-octane (600 MHz ^{1}H-NMR spectra with WET solvent suppression, H_2O at 3.32 ppm; DMSO at 2.50 ppm).

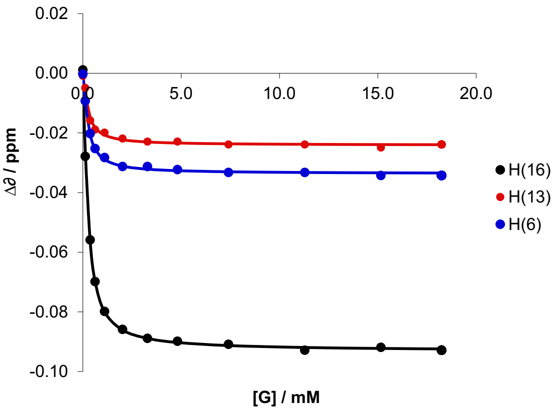

Figure S165 Fitting of the data from the NMR titration of **5** with quinuclidine in *n*-octane (Figure S164) with a 1:1 binding model.

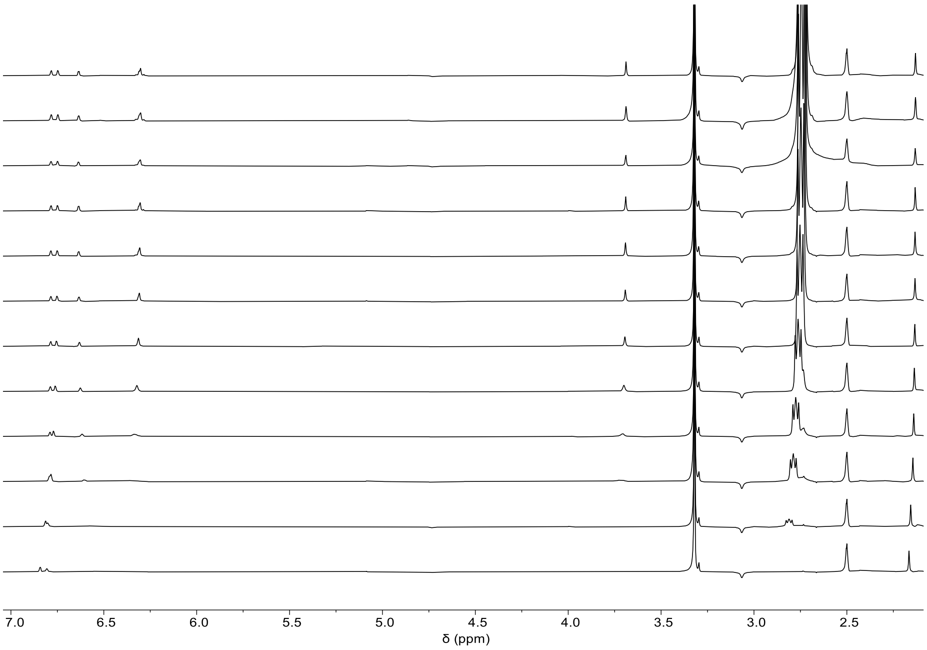

Figure S166 500 MHz ^{1}H-NMR titration of **9** (0.250 mM) with quinuclidine in *n*-octane (600 MHz ^{1}H-NMR spectra with WET solvent suppression, H_2O at 3.32 ppm; DMSO at 2.50 ppm).

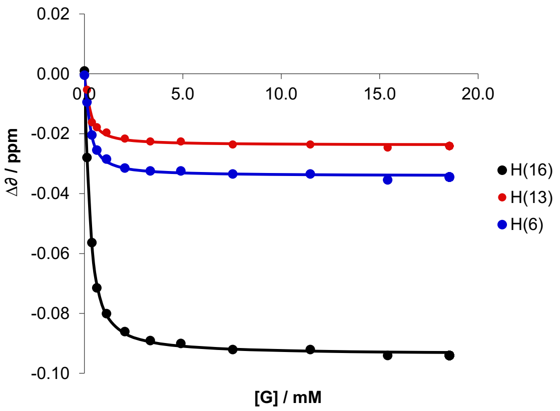

Figure S167 Fitting of the data from the NMR titration of **5** with quinuclidine in *n*-octane (Figure S166) with a 1:1 binding model.

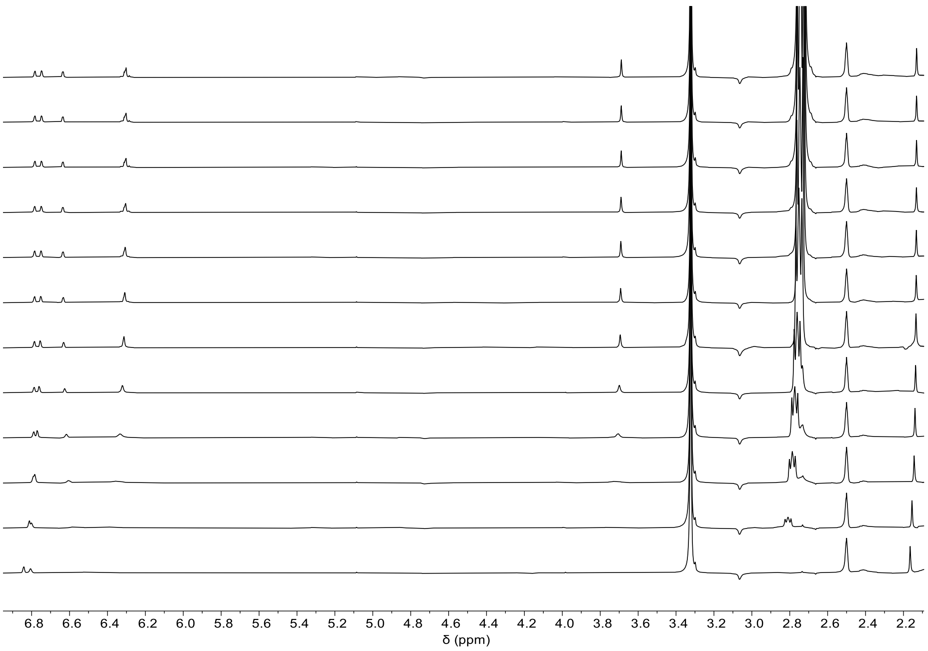

Figure S168 500 MHz ^{1}H-NMR titration of **9** (0.250 mM) with quinuclidine in *n*-octane (600 MHz ^{1}H-NMR spectra with WET solvent suppression, H_2O at 3.32 ppm; DMSO at 2.50 ppm).

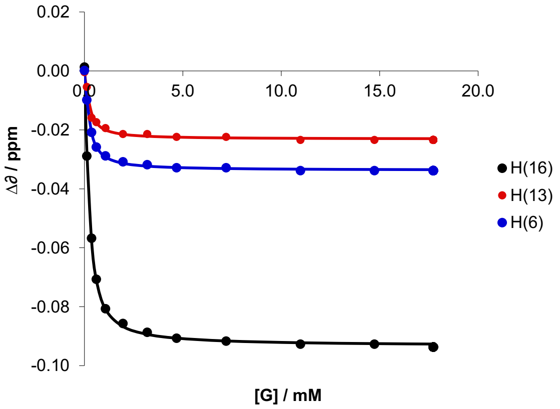

Figure S169 Fitting of the data from the NMR titration of **5** with quinuclidine in *n*-octane (Figure S168) with a 1:1 binding model.

Molecule 14

Dilution Experiment of 14 in *n*-octane

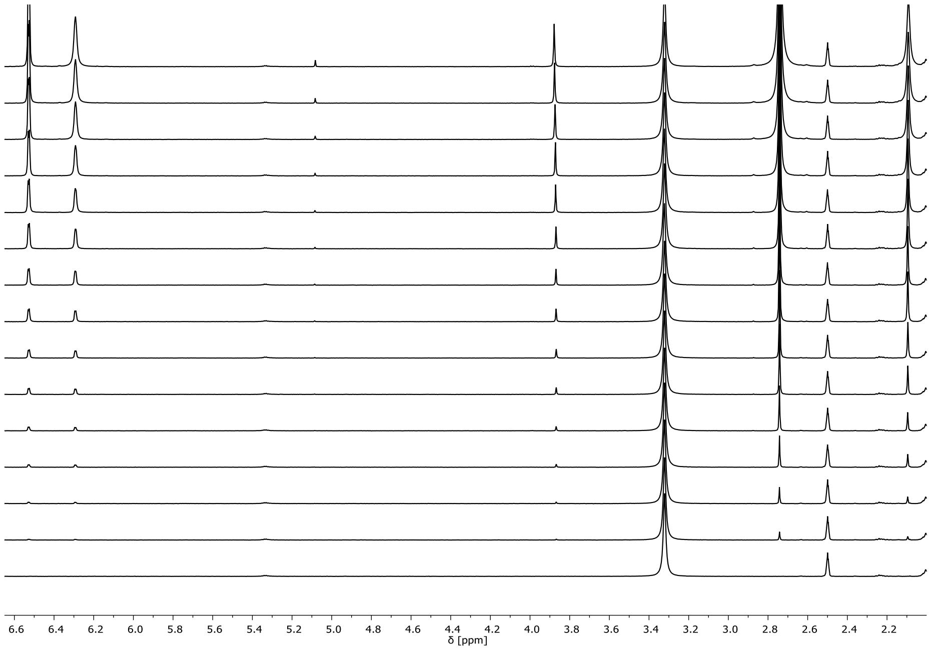

Figure S170 NMR dilution of **14** in *n*-octane (500 MHz ^{1}H-NMR spectra with presat solvent suppression, at the following concentrations of **14** (from bottom to top) 0 mM, 0.033 mM, 0.066 mM, 0.132 mM, 0.196 mM, 0.321 mM, 0.443 mM, 0.674 mM, 0.892 mM, 1.292 mM, 1.649 mM, 2.260 mM, 3.188 mM, 4.129 mM, 5.221 mM).

Titrations of 14 with quinuclidine in *n*-octane

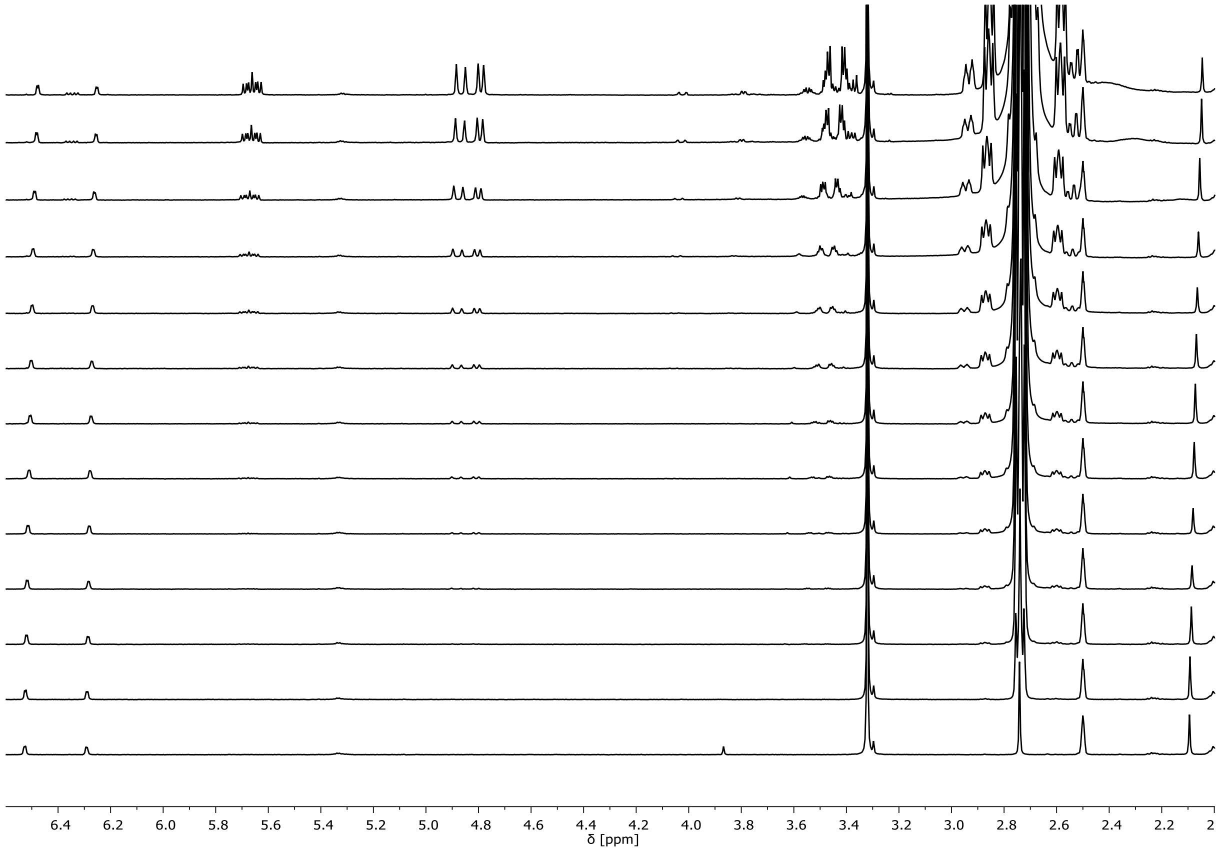

Figure S171 500 MHz ^{1}H-NMR titration of **14** (0.321 mM) with quinuclidine in *n*-octane (500 MHz ^{1}H-NMR spectra with presat solvent suppression, H_2O at 3.32 ppm; DMSO at 2.50 ppm).

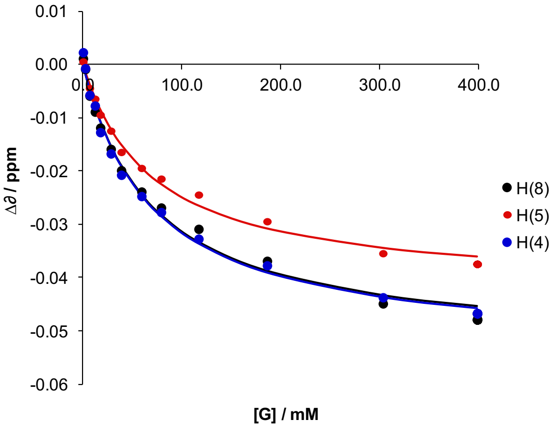

Figure S172 Fitting of the data from the NMR titration of **14** with quinuclidine in *n*-octane (Figure S171) with a 1:1 fitting model.

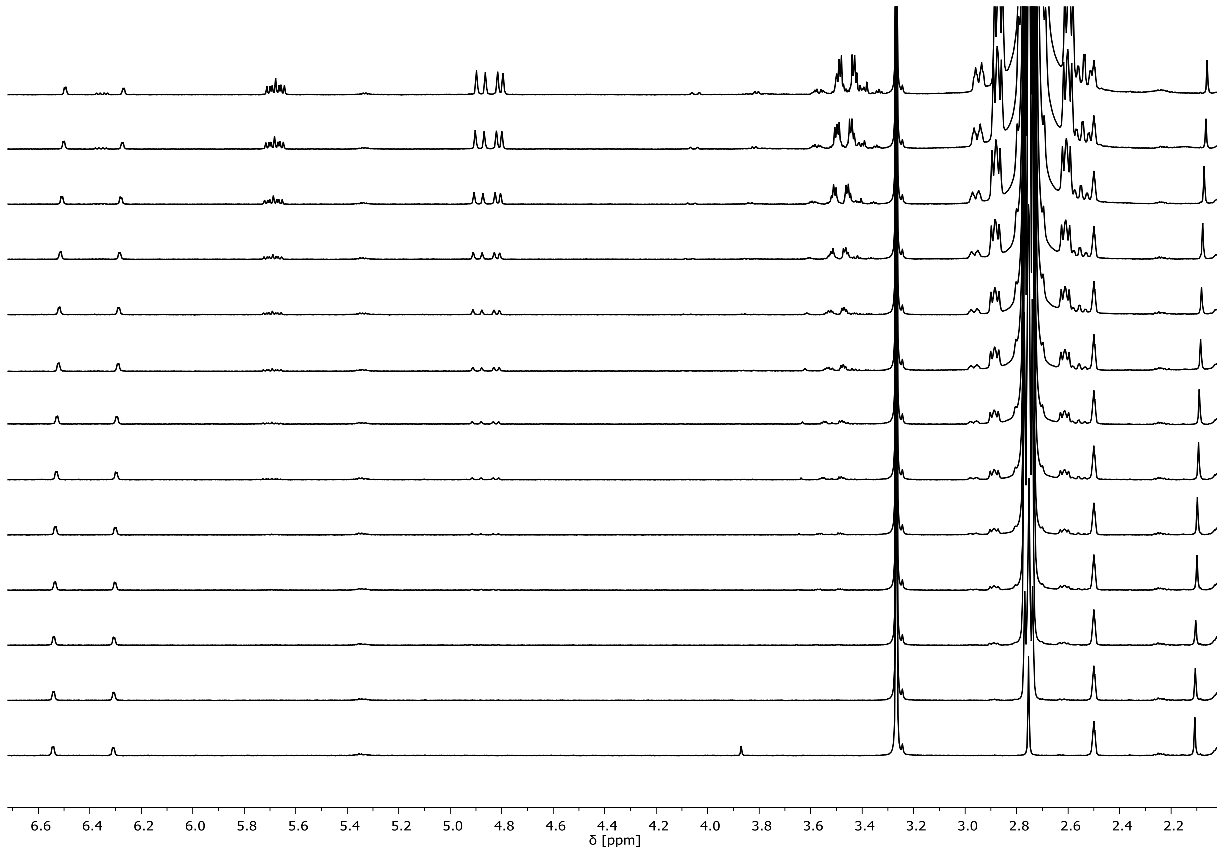

Figure S173 500 MHz ^{1}H-NMR titration of **14** (0.321 mM) with quinuclidine in *n*-octane (500 MHz ^{1}H-NMR spectra with presat solvent suppression, H_2O at 3.32 ppm; DMSO at 2.50 ppm).

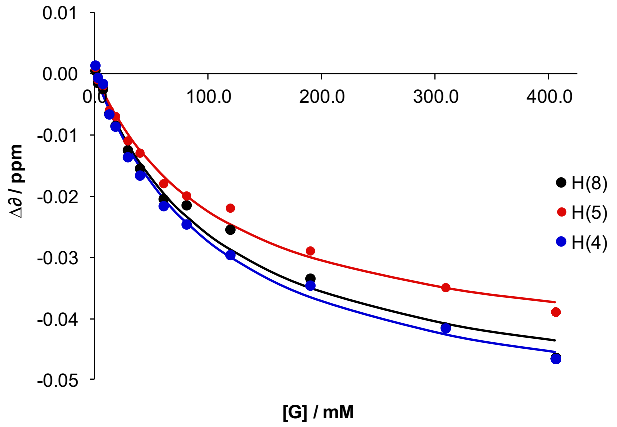

Figure S174 Fitting of the data from the NMR titration of **14** with quinuclidine in *n*-octane (Figure S173) with a 1:1 fitting model.

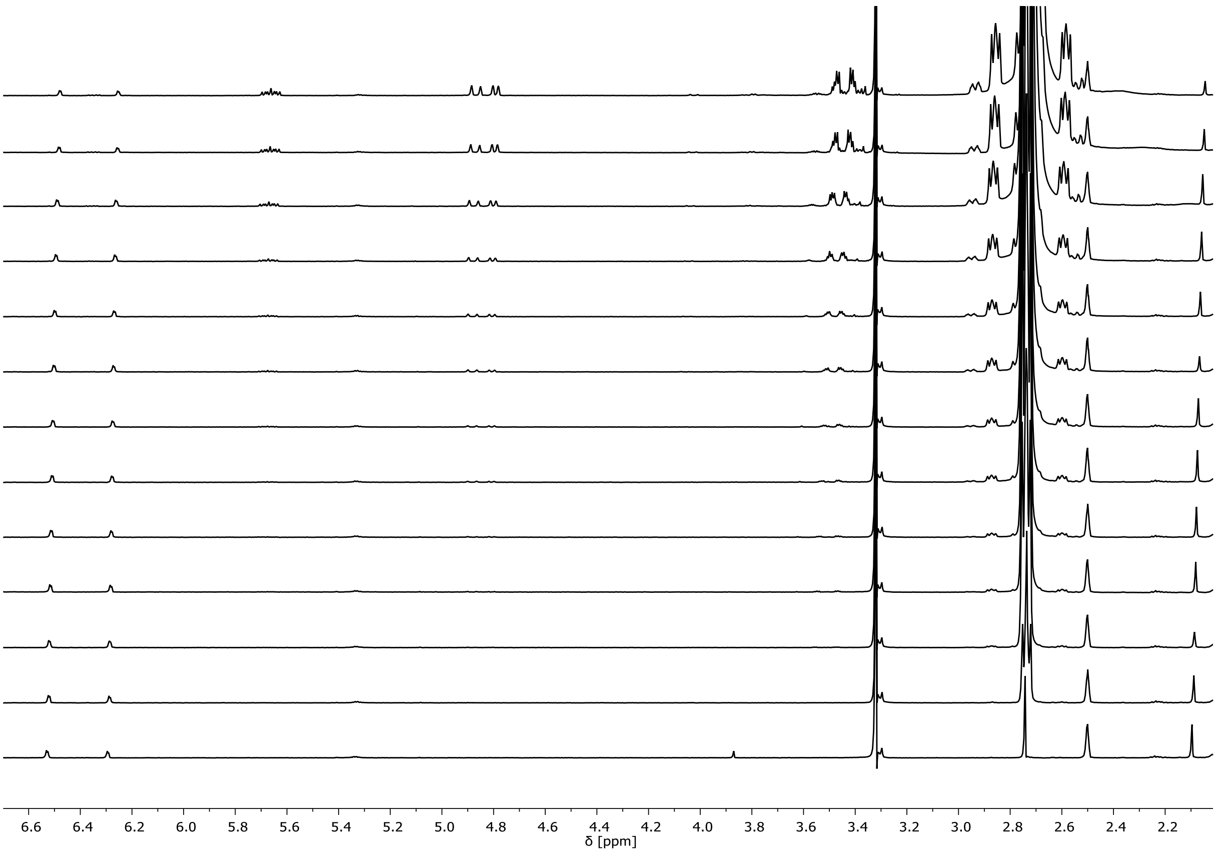

Figure S175 500 MHz ^{1}H-NMR titration of **14** (0.321 mM) with quinuclidine in *n*-octane (500 MHz ^{1}H-NMR spectra with presat solvent suppression, H_2O at 3.32 ppm; DMSO at 2.50 ppm).

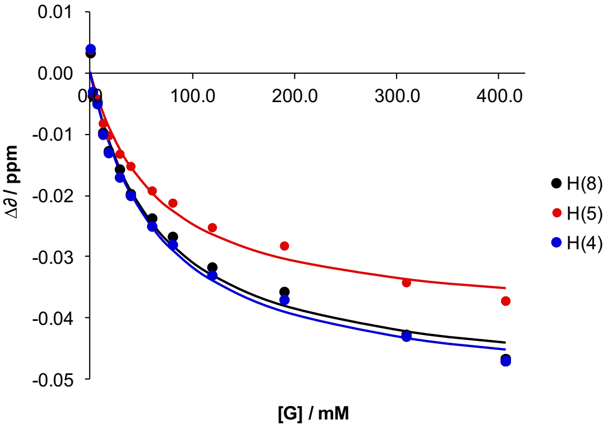

Figure S176 Fitting of the data from the NMR titration of **14** with quinuclidine in *n*-octane (Figure S175) with a 1:1 fitting model.

Molecule 19

Dilution Experiment of 19 in *n*-octane

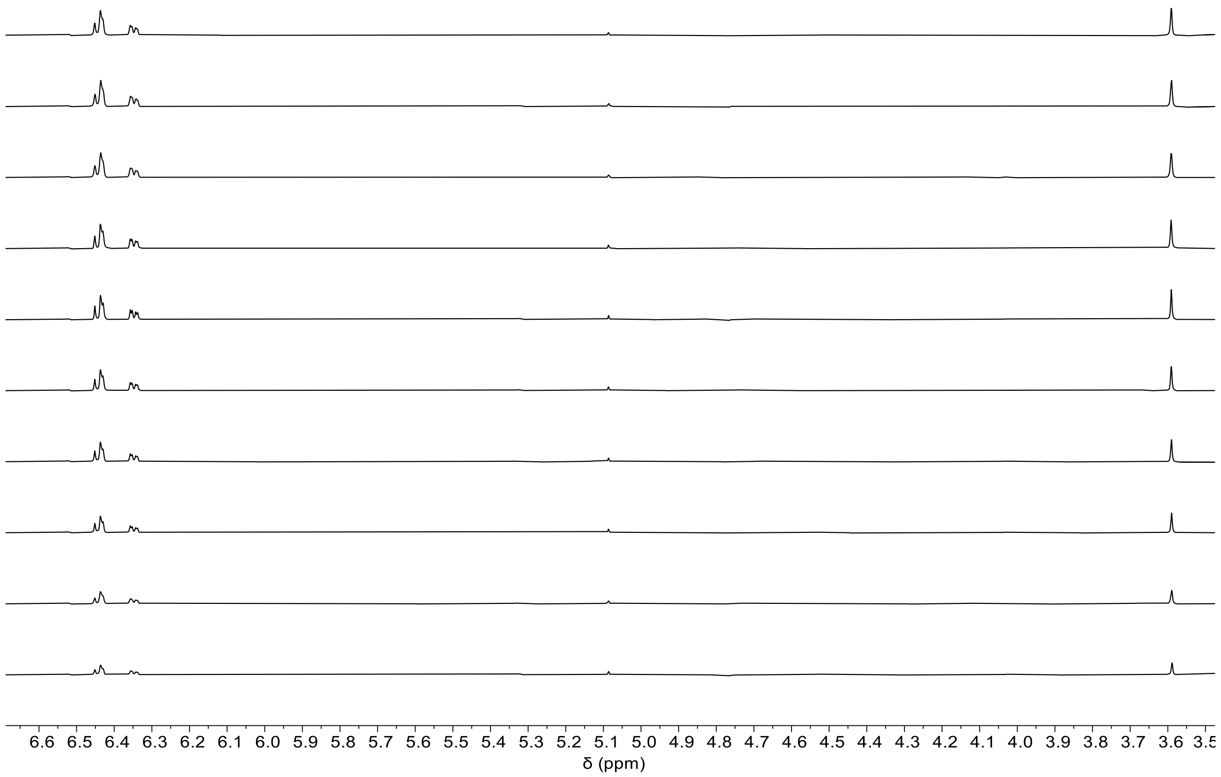

Figure S177 NMR dilution of **19** in *n*-octane (600 MHz ^{1}H-NMR spectra with WET solvent suppression, at the following concentrations of **19** (from bottom to top) 0.086 mM, 0.118 mM, 0.146 mM, 0.172 mM, 0.190 mM, 0.211 mM, 0.224 mM, 0.238 mM, 0.246 mM, 0.250 mM).

Titration of 19 with quinuclidine in *n*-octane

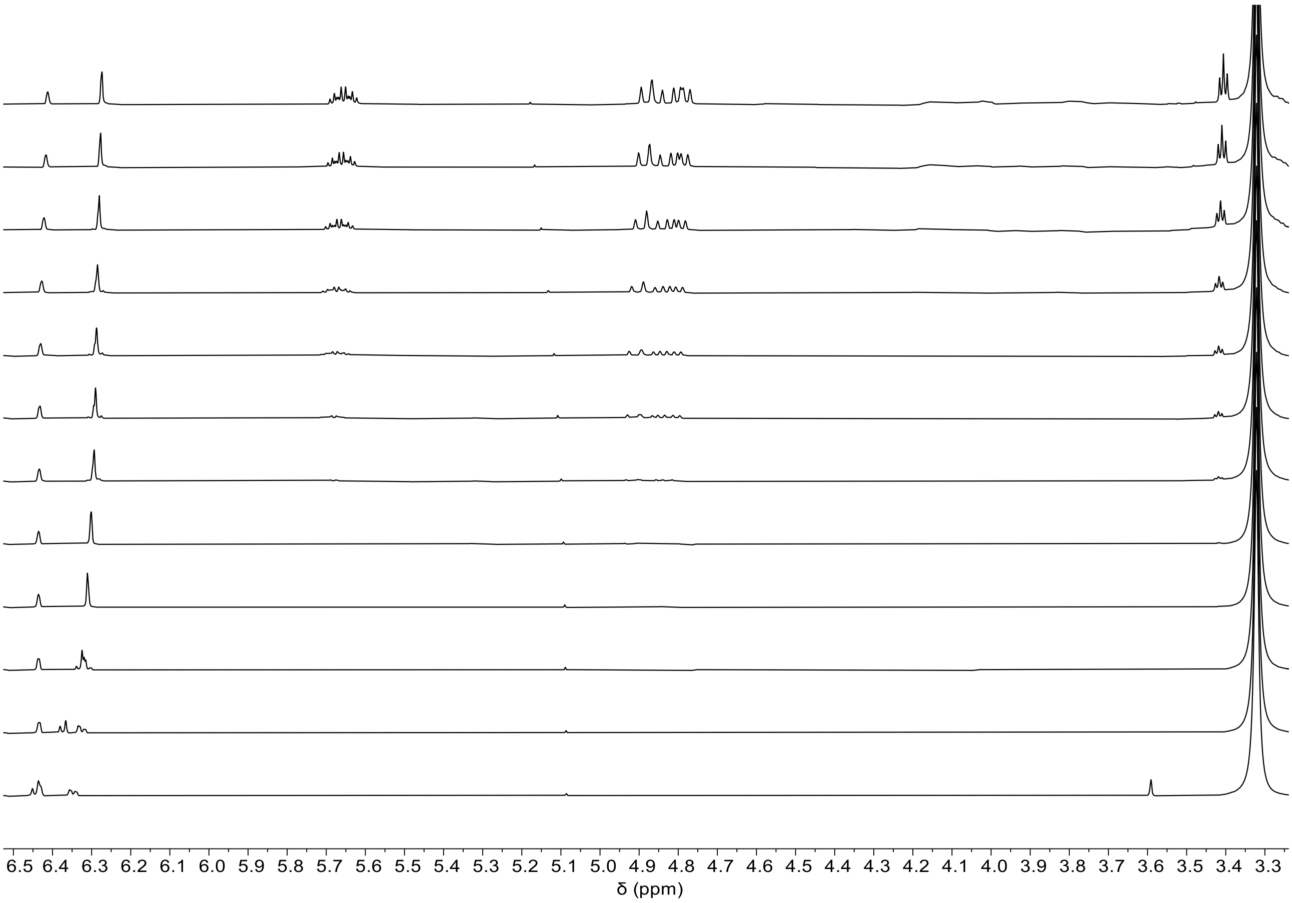

Figure S178 600 MHz ^{1}H-NMR titration of **19** (0.200 mM) with quinuclidine in *n*-octane (600 MHz ^{1}H-NMR spectra with WET solvent suppression).

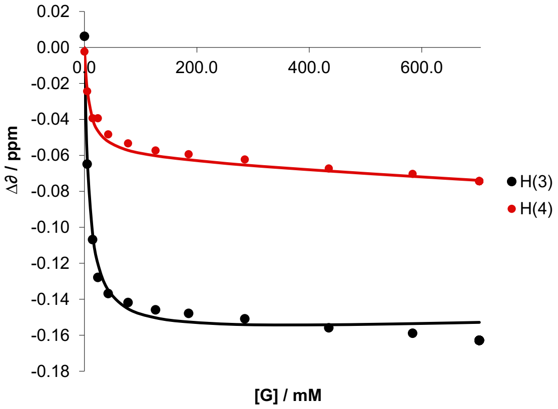

Figure S179 Fitting of the data from the NMR titration of **19** with quinuclidine in *n*-octane (Figure S178) with a 1:1 binding model+non-specific.

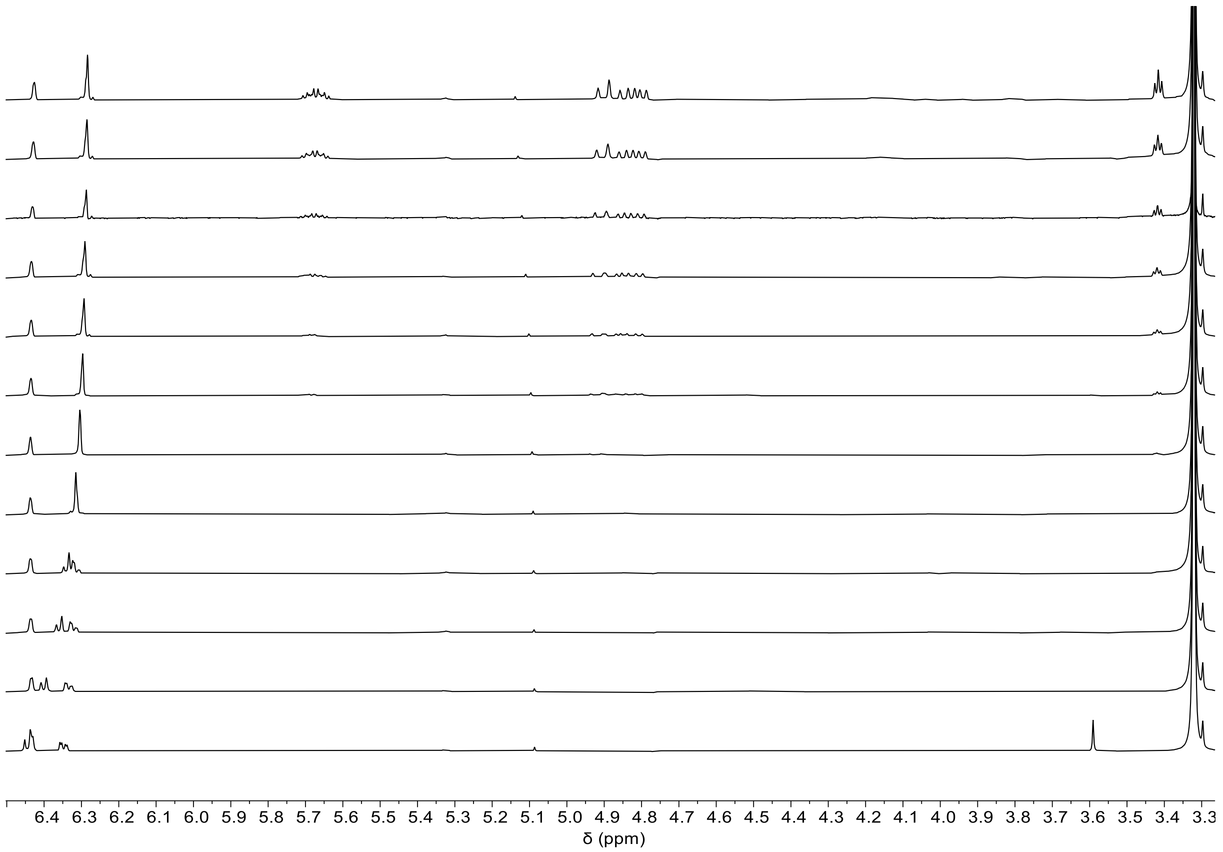

Figure S180 600 MHz ^{1}H-NMR titration of **19** (0.200 mM) with quinuclidine in *n*-octane (600 MHz ^{1}H-NMR spectra with WET solvent suppression).

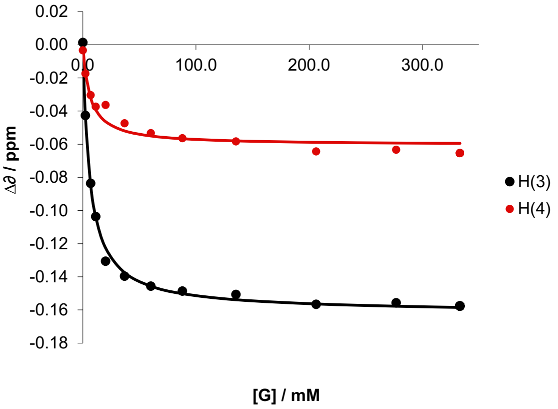

Figure S181 Fitting of the data from the NMR titration of **19** with quinuclidine in *n*-octane (Figure S180) with a 1:1 binding model+non-specific.

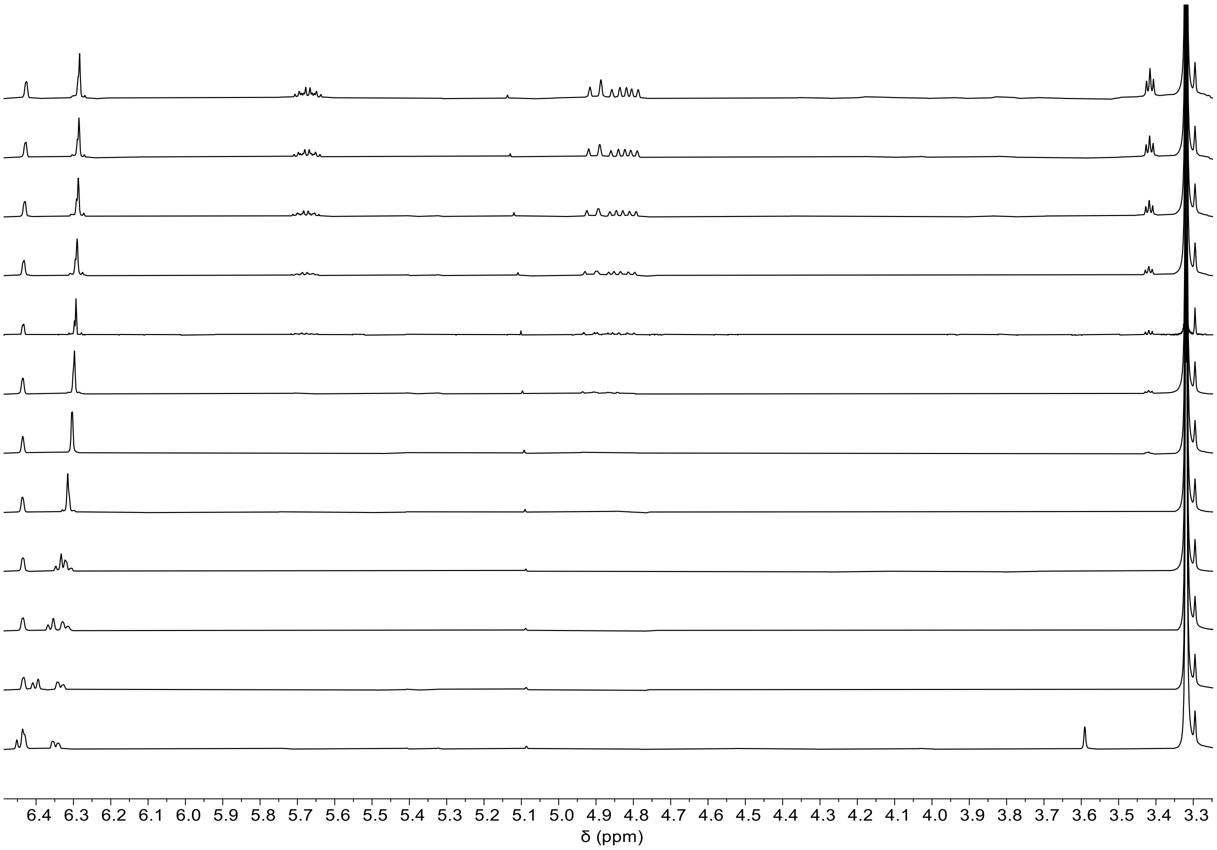

Figure S182 600 MHz ^{1}H-NMR titration of **19** (0.200 mM) with quinuclidine in *n*-octane (600 MHz ^{1}H-NMR spectra with WET solvent suppression).

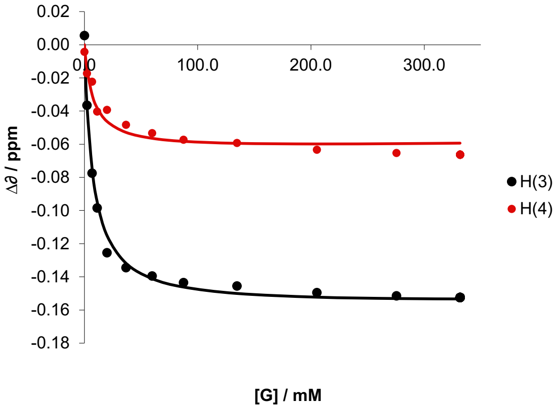

Figure S183 Fitting of the data from the NMR titration of **19** with quinuclidine in *n*-octane (Figure S182) with a 1:1 binding model+non-specific.

6. UV-Vis Experiments

UV-Vis Titration – General Procedure

A diluted solution of the host (5 mL) in *n*-octane is prepared from the stock solution of the receptor in *n*-octane. 2 mL of the host solution is titrated with a solution of the guest containing also the host at the same concentration in *n*-octane. A UV-Vis spectrum is recorded for every point of the titration. Analogous to the NMR titrations, equation (6) can be written as

$$\frac{A_{obs}-A_0}{A_f-A_0} = \frac{K_a[G]}{1+K_a[G]} \quad (6)$$

where A_{obs} (a.u.) is the observed absorbance, A_0 (a.u.) is the initial absorbance, A_f (a.u.) is the final absorbance, K_a is the association constant and [G] is the concentration of free guest. [G] can be determined using equation (7) by making iteratively guesses of K_a and solving for [G] until the theoretical isotherm matches the experimental data:

$$K_a[G]^2 + (K_a[H]_0 - K_a[G]_0 + 1)[G] - [G]_0 = 0 \quad (7)$$

where $[H]_0$ and $[G]_0$ are the total concentrations of the host and guest, respectively. A Microsoft Excel spreadsheet with purpose-written VBA macros was used to solve equations (6) and (7) fitting the experimentally measured absorbance at specified wavelengths.[17]

Each titration was repeated three times, fitted with equations (6) and (7), and an average value of the association constant along with its standard error (with 95% confidence) is reported in Table S3.

UV-Vis Dilution – General Procedure

A diluted solution of the host (5 mL) in *n*-octane is prepared from the stock solution of the receptor in *n*-octane. Increasing volumes of *n*-octane were added to 2 mL or 1.5 mL of the host solution. A UV-Vis spectrum is recorded after each addition.

Donor	Acceptor<br><br>Quin	Donor	Acceptor<br><br>Quin
**1**	(1.1 ± 0.3)x10^{5}	**11**	93 ± 14
**2**	(1.9 ± 0.1)x10^{4}	**12**	78 ± 9
**3**	(1.8 ± 0.2)x10^{4}	**15**	(8.3 ± 0.1)x10^{3}
**6**	(1.2 ± 0.7)x10^{5}	**16**	(8.3 ± 0.4)x10^{2}
**7**	(3.3 ± 0.2)x10^{4}	**17**	(6.1 ± 0.1)x10^{2}
**8**	(1.6 ± 0.1)x10^{4}	**43**	(2.4 ± 0.2)x10^{2}
**10**	(4.9 ± 0.2)x10^{2}		

Table S3 Association constants (M^{-1}) for formation of 1:1 complexes measured by UV-Vis spectroscopy titrations in *n*-octane at 298 K. Errors are the standard error of the mean of three independent experiments. (Compound **43** is 2,2'-methylenebis(6-*tert*-butyl-4-methylphenol)).

Molecule 1

Dilution Experiment of 1 in *n*-octane

(a)

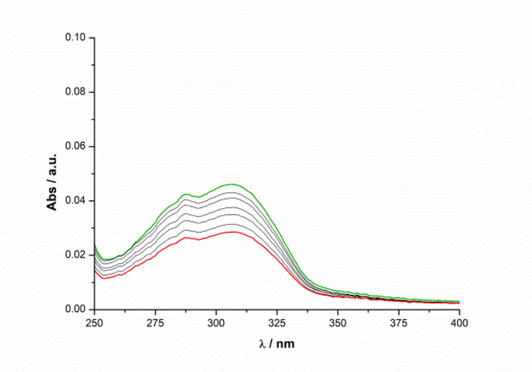

(b)

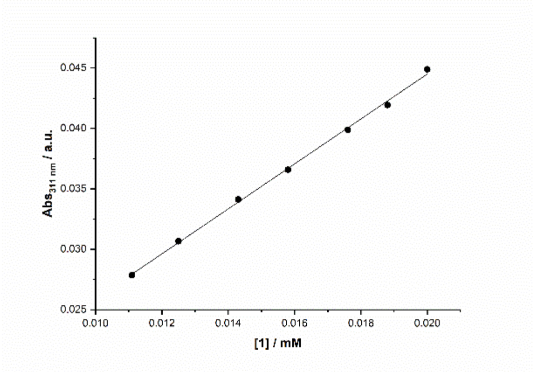

Figure S184 (a) UV-Vis spectra of **1** in *n*-octane at decreasing concentrations (from 0.020 mM in green to 0.011 mM in red), and (b) plot of the absorbance of **1** at 311 nm versus the concentration of **1** and its linear fitting.

Titrations of 1 with quinuclidine in *n*-octane

(a)

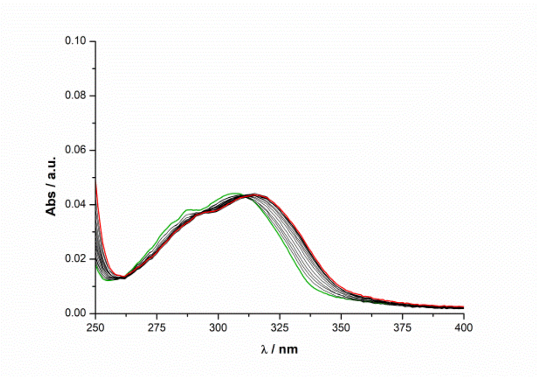

(b)

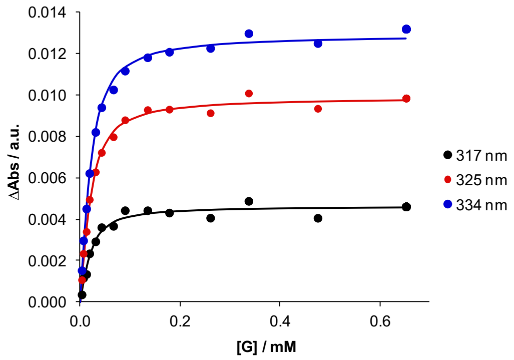

(c)

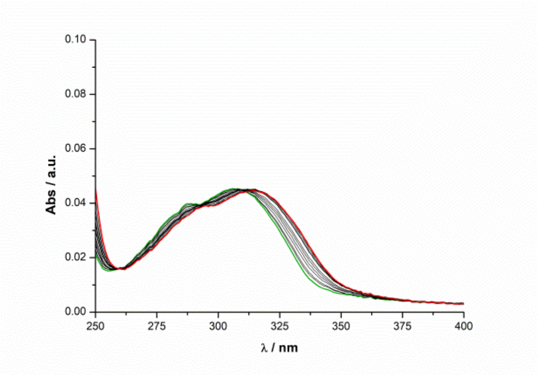

(d)

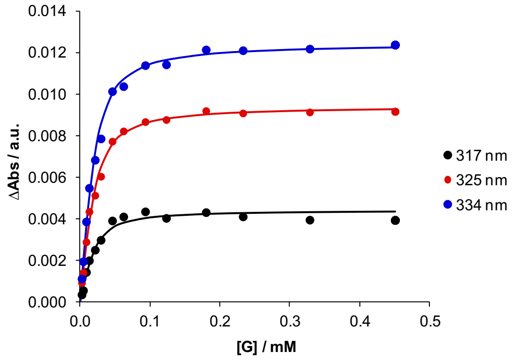

(e)

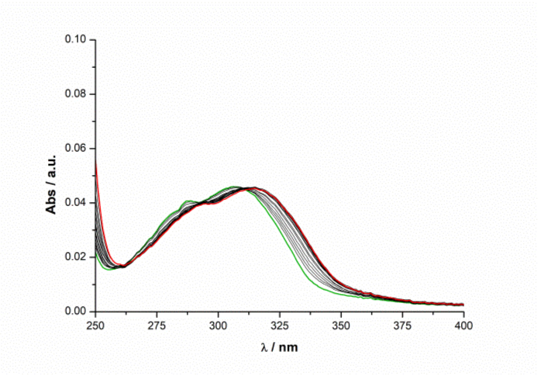

(f)

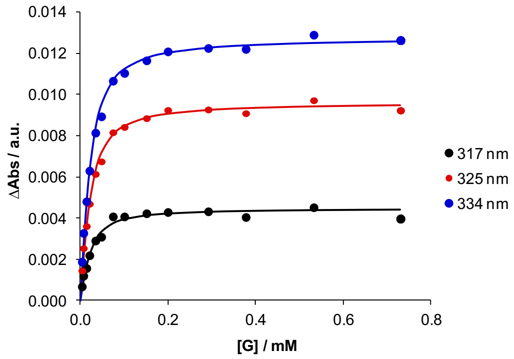

Figure S185 (a, c, e) UV-Vis spectra of **1** (0.020 mM in green) in *n*-octane at increasing concentrations of quinuclidine (from green to red), and (b, d, f) plot of the absorbance of **1** at 317 nm, 325 nm, 334 nm versus the concentration of **1** and its fittings to a 1:1 binding model.

Molecule 2

Dilution Experiment of 2 in *n*-octane

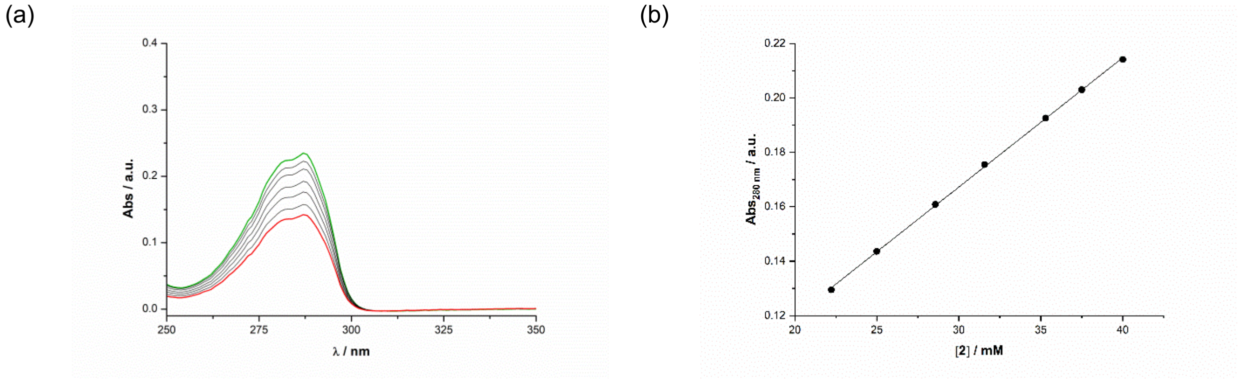

Figure S186 (a) UV-Vis spectra of **2** in *n*-octane at decreasing concentrations (from 0.040 mM in green to 0.025 mM in red), and (b) plot of the absorbance of **2** at 280 nm versus the concentration of **2** and its linear fitting.

Titrations of 2 with quinuclidine in *n*-octane

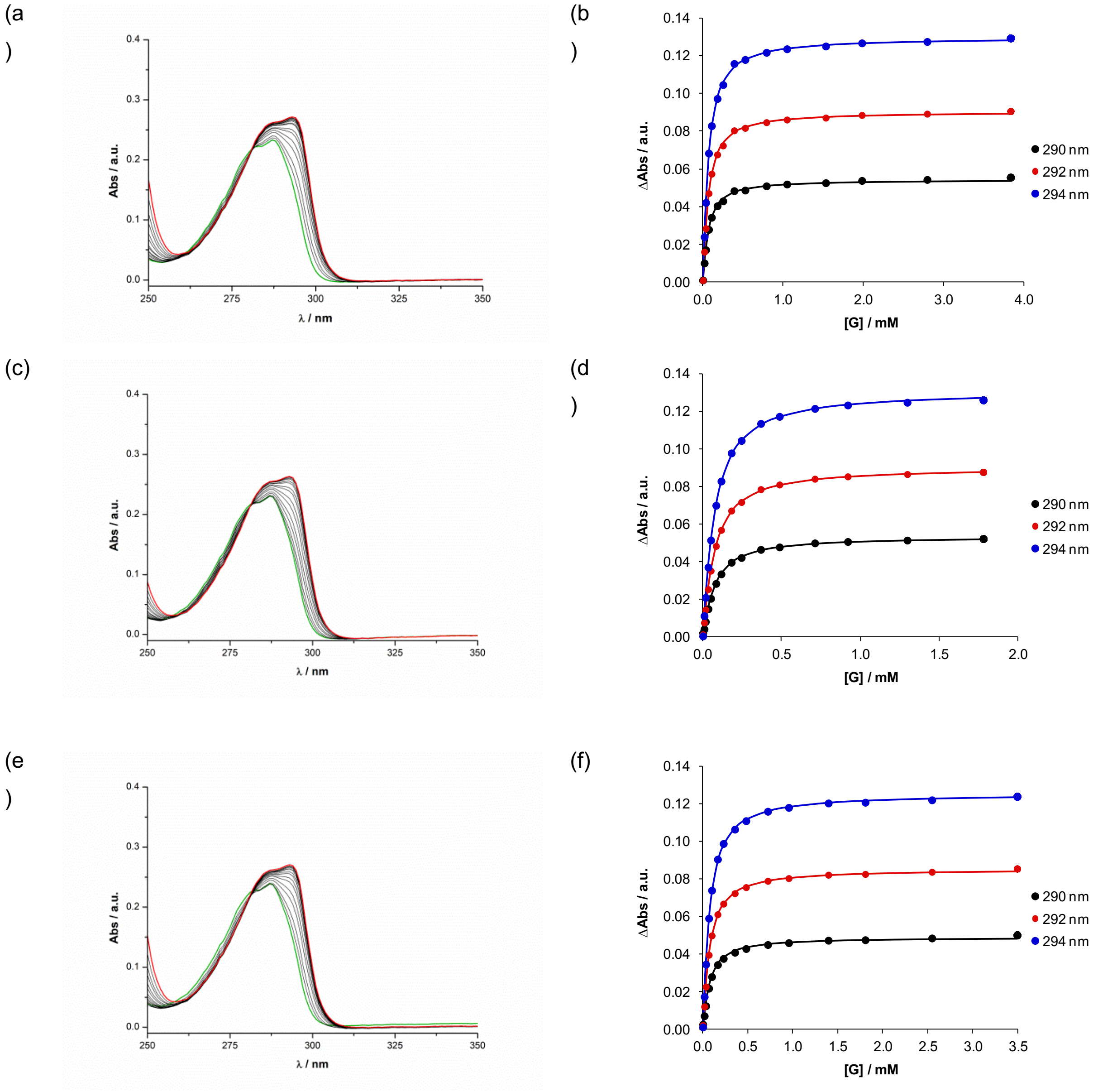

Figure S187 (a, c, e) UV-Vis spectra of **2** (0.040 mM in green) in *n*-octane at increasing concentrations of quinuclidine (from green to red), and (b, d, f) plot of the absorbance of **2** at 290 nm, 292 nm, 294 nm versus the concentration of **2** and its fittings to a 1:1 binding model.

Molecule 3

Dilution Experiment of 3 in *n*-octane

(a) (b)

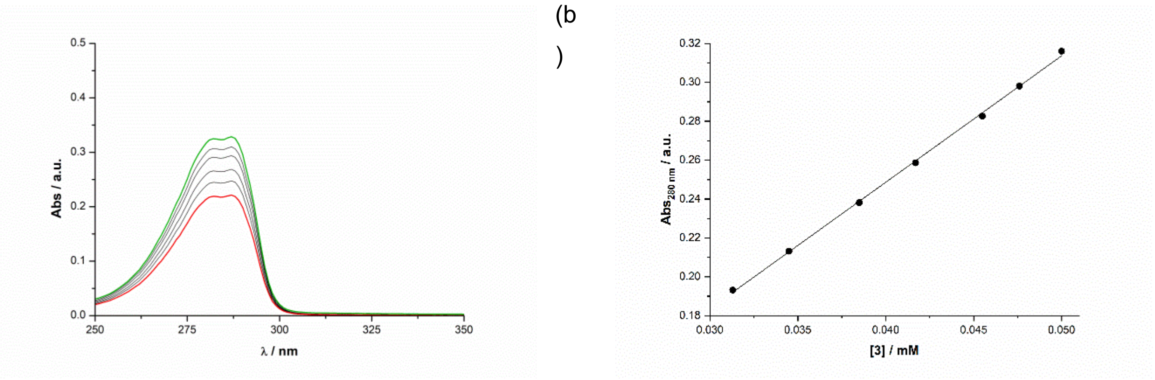

Figure S188 (a) UV-Vis spectra of **3** in *n*-octane at decreasing concentrations (from 0.050 mM in green to 0.031 mM in red), and (b) plot of the absorbance of **3** at 280 nm versus the concentration of **3** and its linear fitting.

Titrations of 3 with quinuclidine in *n*-octane

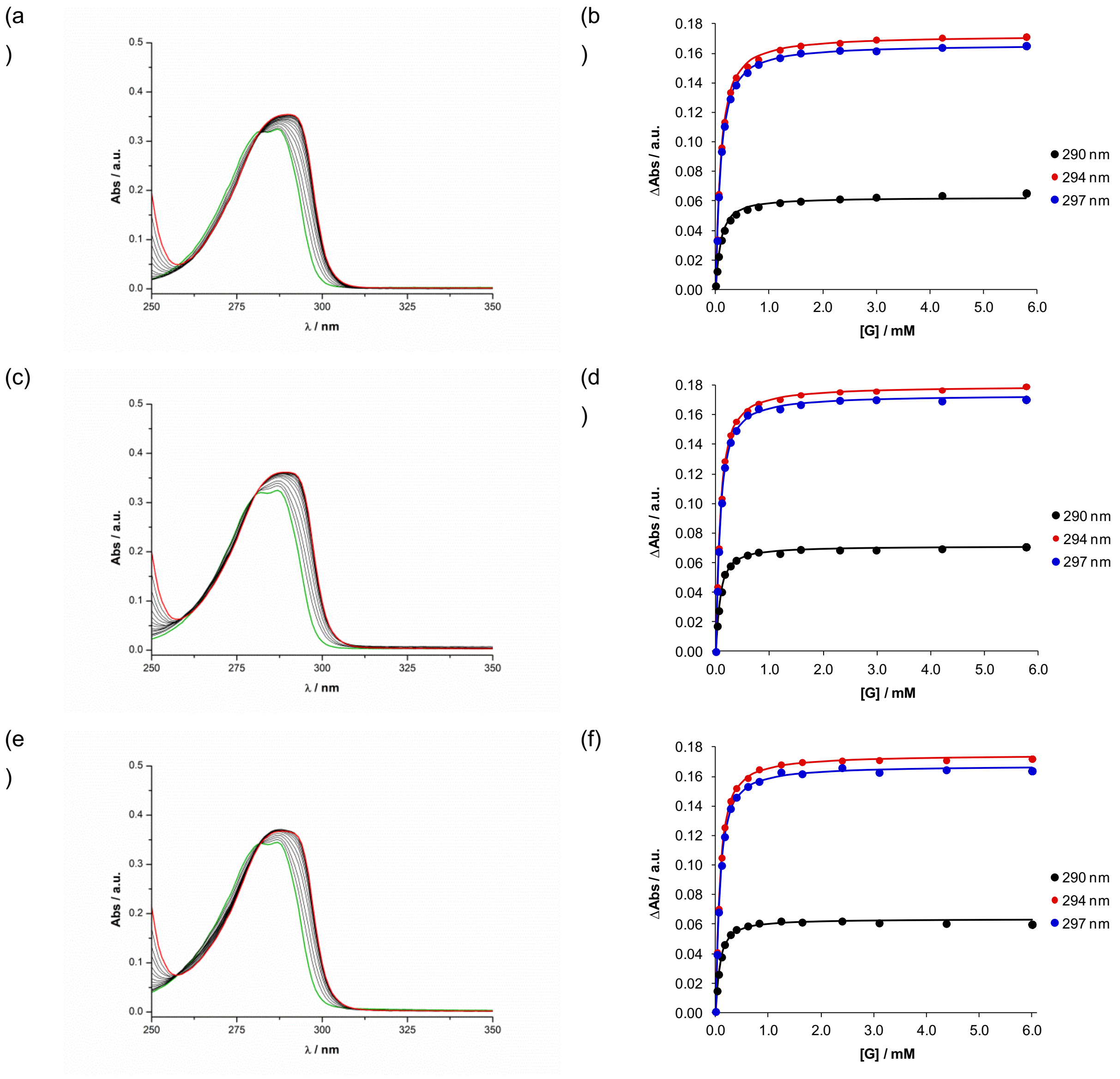

Figure S189 (a, c, e) UV-Vis spectra of **3** (0.050 mM in green) in *n*-octane at increasing concentrations of quinuclidine (from green to red), and (b, d, f) plot of the absorbance of **3** at 290 nm, 294 nm, 297 nm versus the concentration of **3** and its fittings to a 1:1 binding model.

Molecule 6

Dilution Experiment of 6 in *n*-octane

(a
)

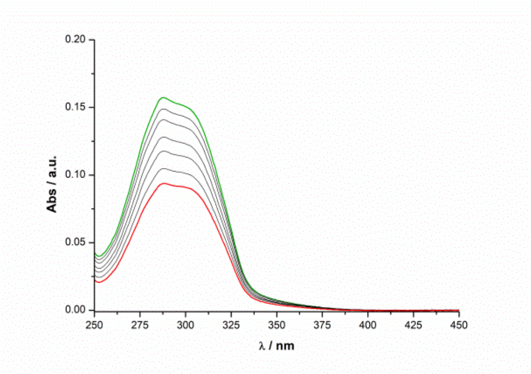

(b
)

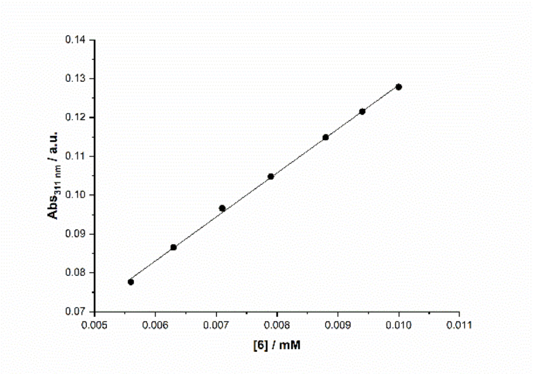

Figure S190 (a) UV-Vis spectra of **6** in *n*-octane at decreasing concentrations (from 0.010 mM in green to 0.006 mM in red), and (b) plot of the absorbance of **6** at 311 nm versus the concentration of **6** and its linear fitting.

Titrations of 6 with quinuclidine in *n*-octane

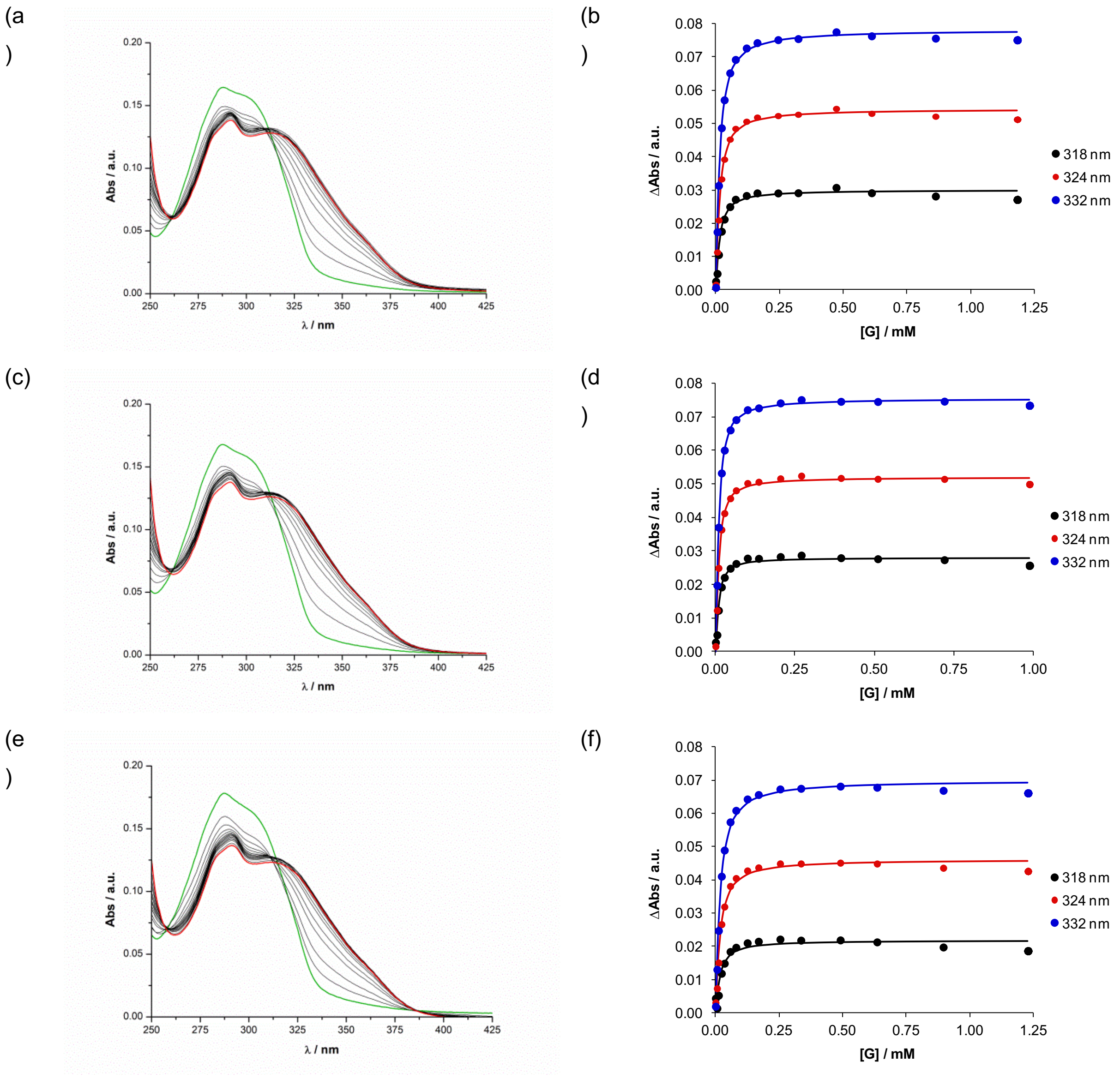

Figure S191 (a, c, e) UV-Vis spectra of **6** (0.010 mM in green) in *n*-octane at increasing concentrations of quinuclidine (from green to red), and (b, d, f) plot of the absorbance of **6** at 318 nm, 324 nm, 332 nm versus the concentration of **6** and its fittings to a 1:1 binding model.

Molecule 7

Dilution Experiment of 7 in *n*-octane

(a
)

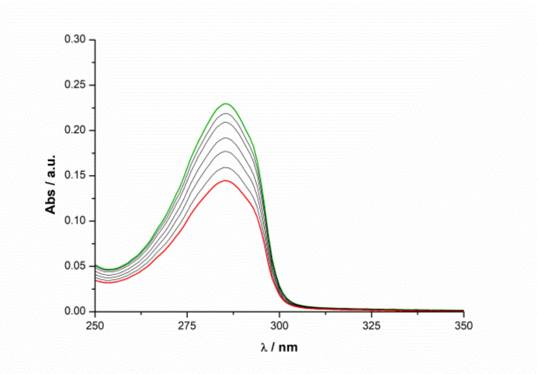

(b
)

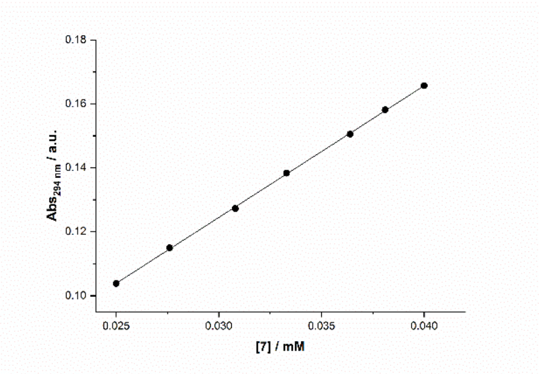

Figure S192 (a) UV-Vis spectra of **7** in *n*-octane at decreasing concentrations (from 040 mM in green to 0.025 mM in red), and (b) plot of the absorbance of **7** at 294 nm versus the concentration of **7** and its linear fitting.

Titrations of 7 with quinuclidine in *n*-octane

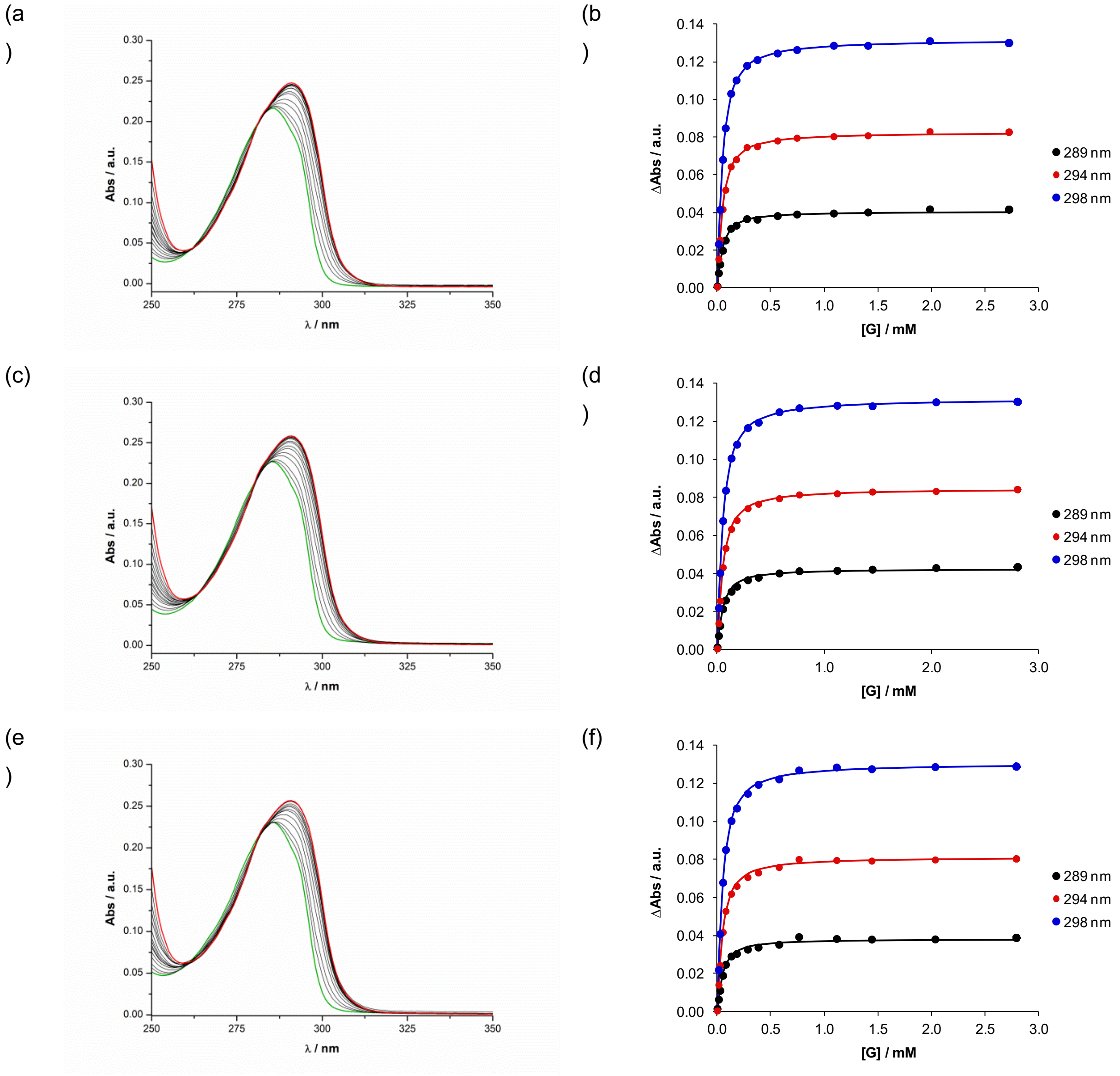

Figure S193 (a, c, e) UV-Vis spectra of **7** (0.040 mM in green) in *n*-octane at increasing concentrations of quinuclidine (from green to red), and (b, d, f) plot of the absorbance of **7** at 289 nm, 294 nm, 298 nm versus the concentration of **7** and its fittings to a 1:1 binding model.

Molecule 8

Dilution Experiment of 8 in *n*-octane

(a)

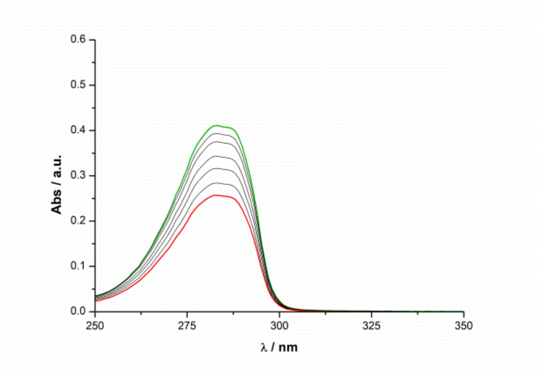

(b)

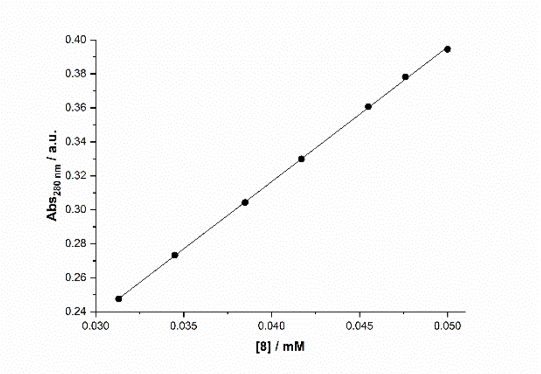

Figure S194 (a) UV-Vis spectra of **8** in *n*-octane at decreasing concentrations (from 0.050 mM in green to 0.031 mM in red), and (b) plot of the absorbance of **8** at 280 nm versus the concentration of **8** and its linear fitting.

Titrations of 8 with quinuclidine in *n*-octane

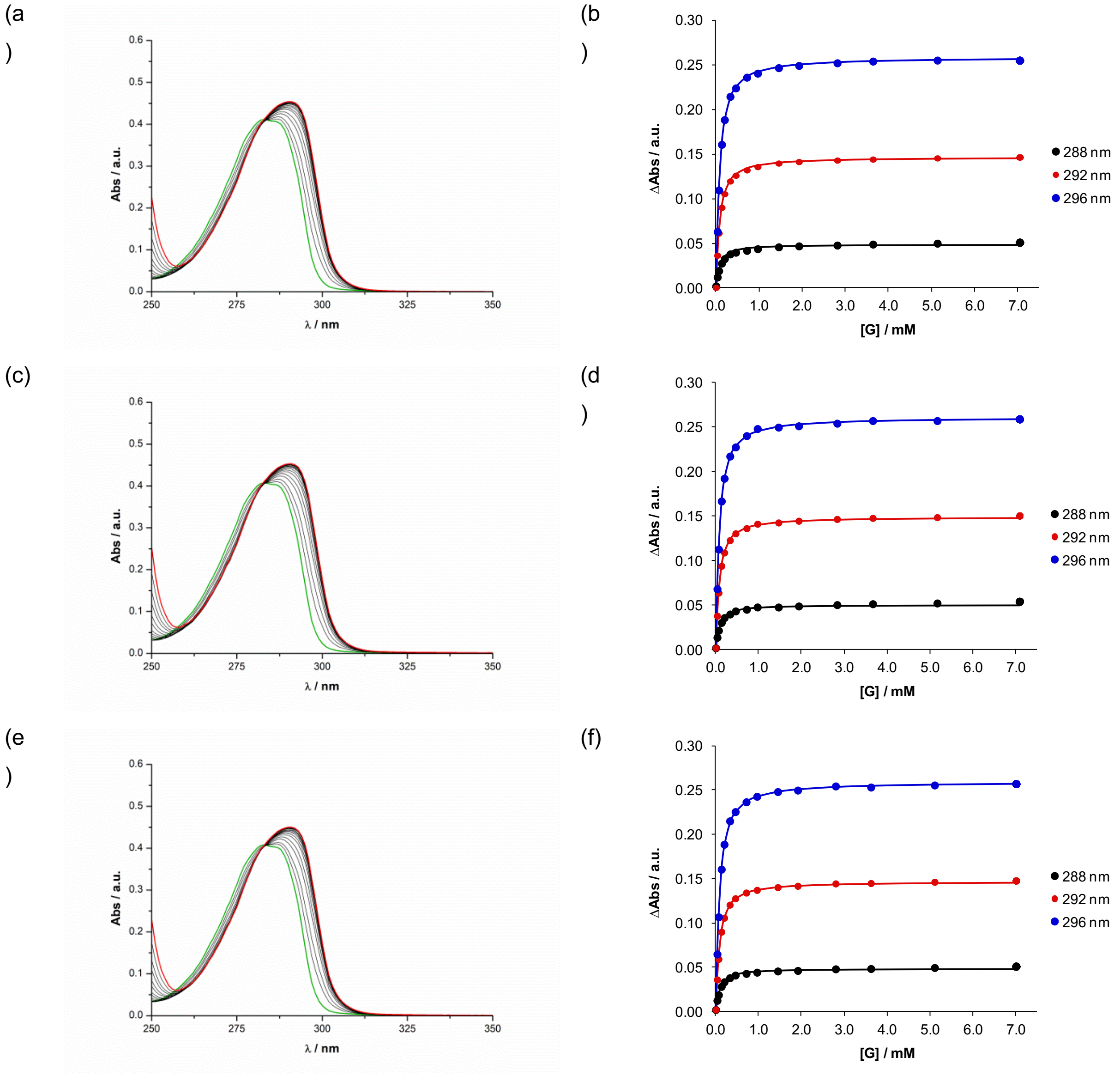

Figure S195 (a, c, e) UV-Vis spectra of **8** (0.050 mM in green) in *n*-octane at increasing concentrations of quinuclidine (from green to red), and (b, d, f) plot of the absorbance of **8** at 288 nm, 292 nm, 296 nm versus the concentration of **8** and its fittings to a 1:1 binding model.

Molecule 10

Dilution Experiment of 10 in *n*-octane

(a)

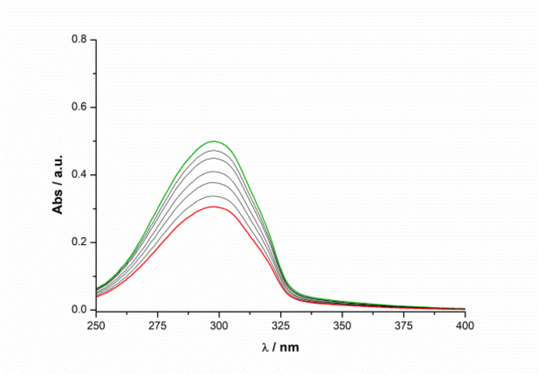

(b)

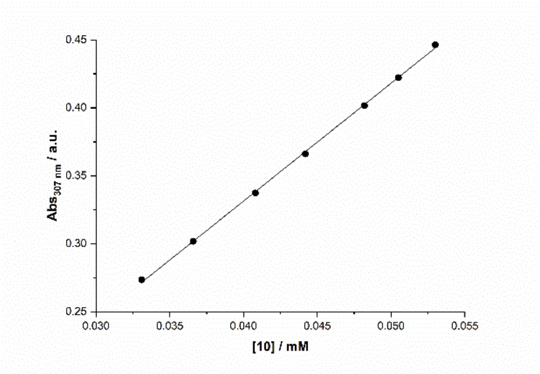

Figure S196 (a) UV-Vis spectra of **10** in *n*-octane at decreasing concentrations (from 0.053 mM in green to 0.033 mM in red), and (b) plot of the absorbance of **10** at 307 nm versus the concentration of **10** and its linear fitting.

Titrations of 10 with quinuclidine in *n*-octane

(a
)

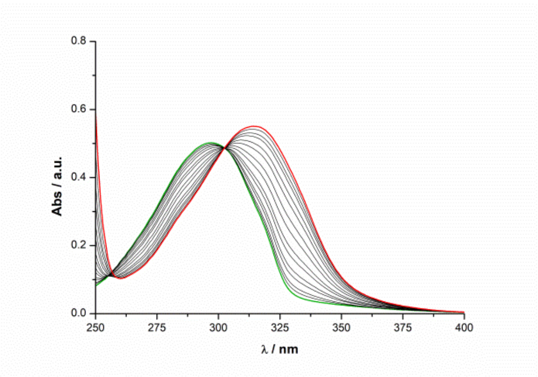

(b
)

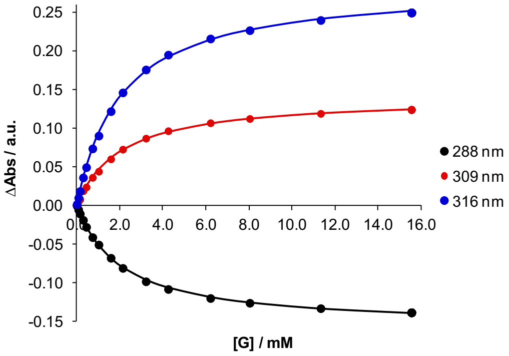

(c)

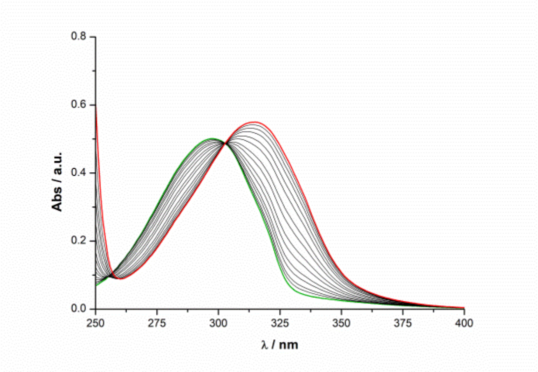

(d
)

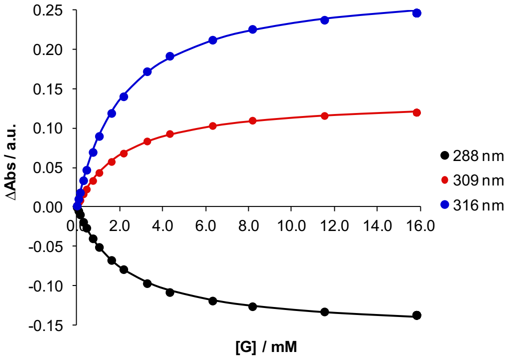

(e
)

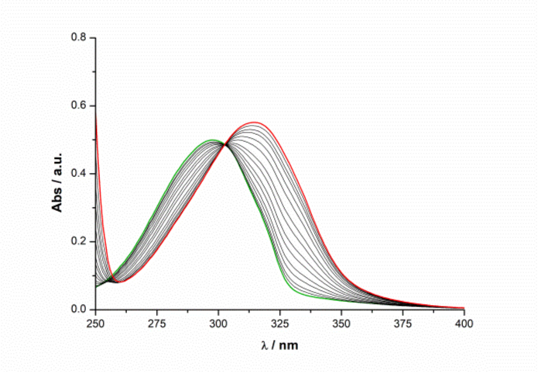

(f)

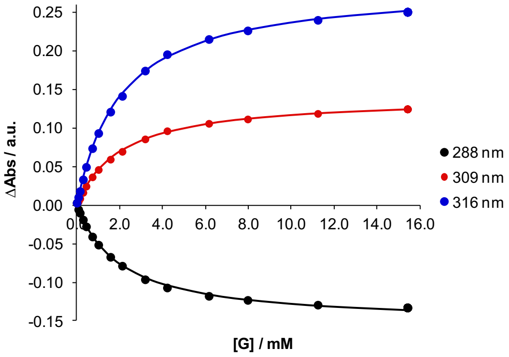

Figure S197 (a, c, e) UV-Vis spectra of **10** (0.053 mM in green) in *n*-octane at increasing concentrations of quinuclidine (from green to red), and (b, d, f) plot of the absorbance of **10** at 288 nm, 309 nm, 316 nm versus the concentration of **10** and its fittings to a 1:1 binding model.

Molecule 11

Dilution Experiment of 11 in *n*-octane

(a) (b)

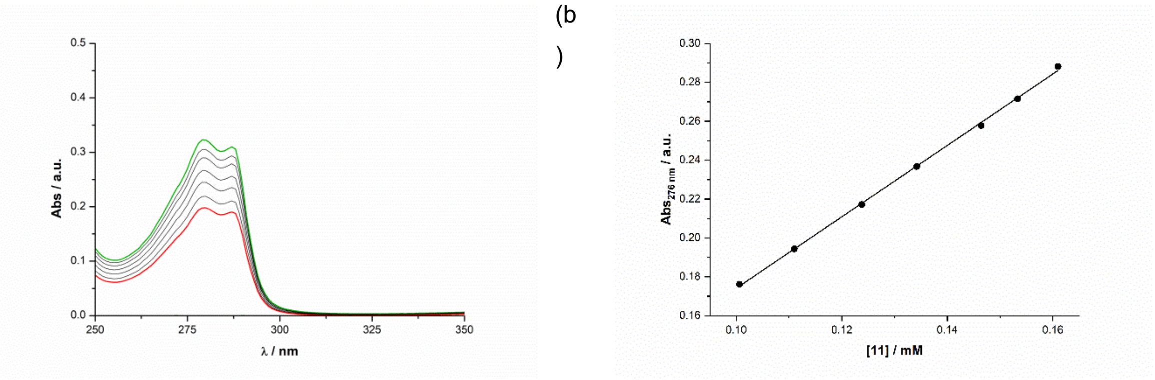

Figure S198 (a) UV-Vis spectra of **11** in *n*-octane at decreasing concentrations (from 0.161 mM in green to 0.101 mM in red), and (b) plot of the absorbance of **11** at 276 nm versus the concentration of **11** and its linear fitting.

Titrations of 11 with quinuclidine in *n*-octane

(a
)

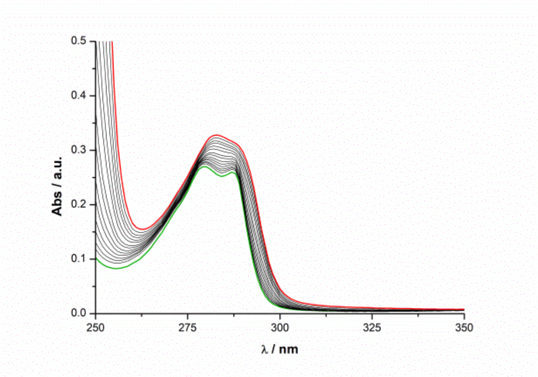

(b
)

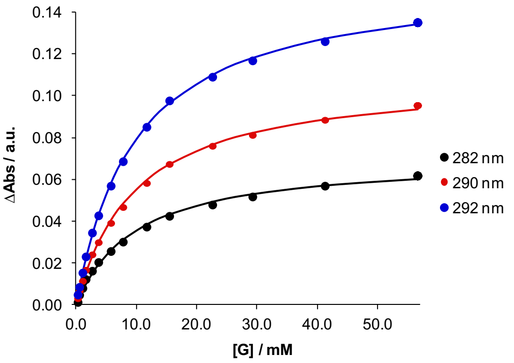

(c)

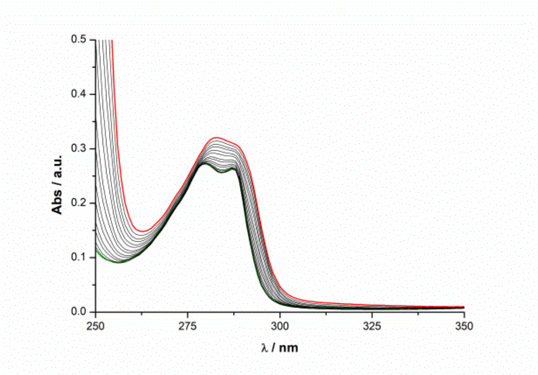

(d
)

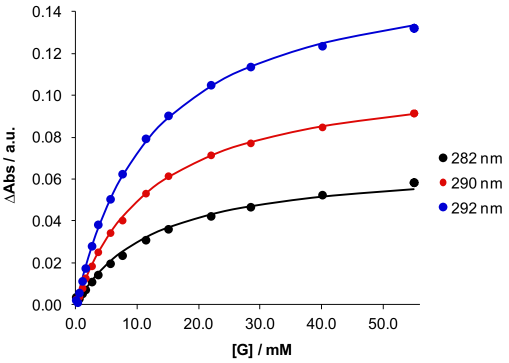

(e
)

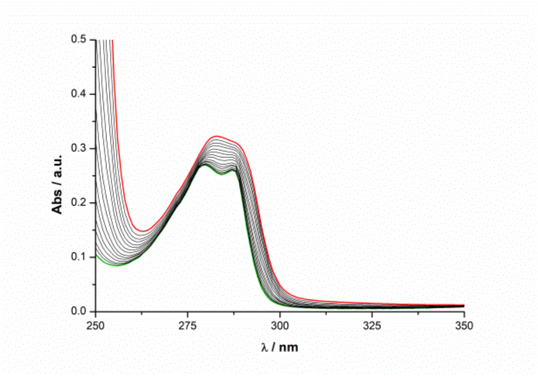

(f)

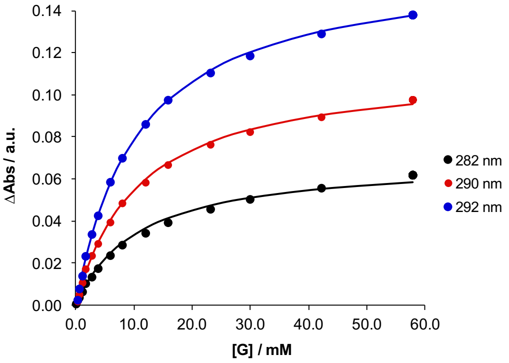

Figure S199 (a, c, e) UV-Vis spectra of **11** (0.161 mM in green) in *n*-octane at increasing concentrations of quinuclidine (from green to red), and (b, d, f) plot of the absorbance of **11** at 282 nm, 290 nm, 292 nm versus the concentration of **11** and its fittings to a 1:1 binding model.

Molecule 12

Dilution Experiment of 12 in *n*-octane

(a)

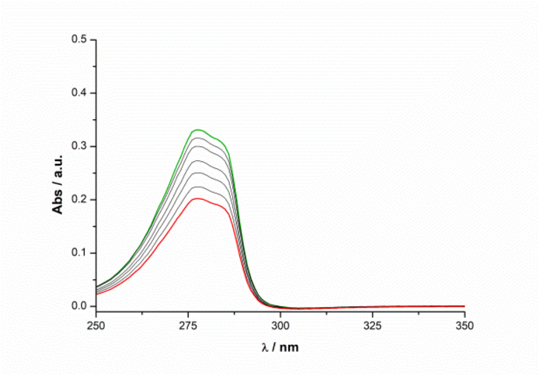

(b)

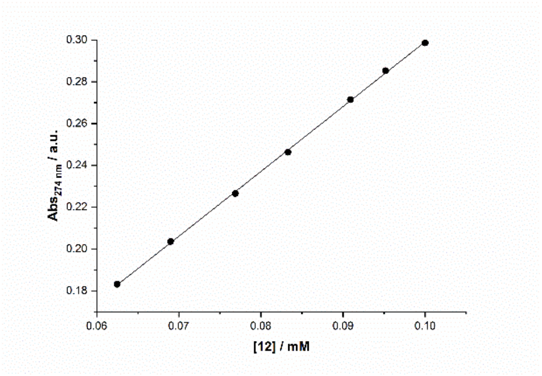

Figure S200 (a) UV-Vis spectra of **12** in *n*-octane at decreasing concentrations (from 0.100 mM in green to 0.063 mM in red), and (b) plot of the absorbance of **12** at 274 nm versus the concentration of **12** and its linear fitting.

Titrations of 12 with quinuclidine in *n*-octane

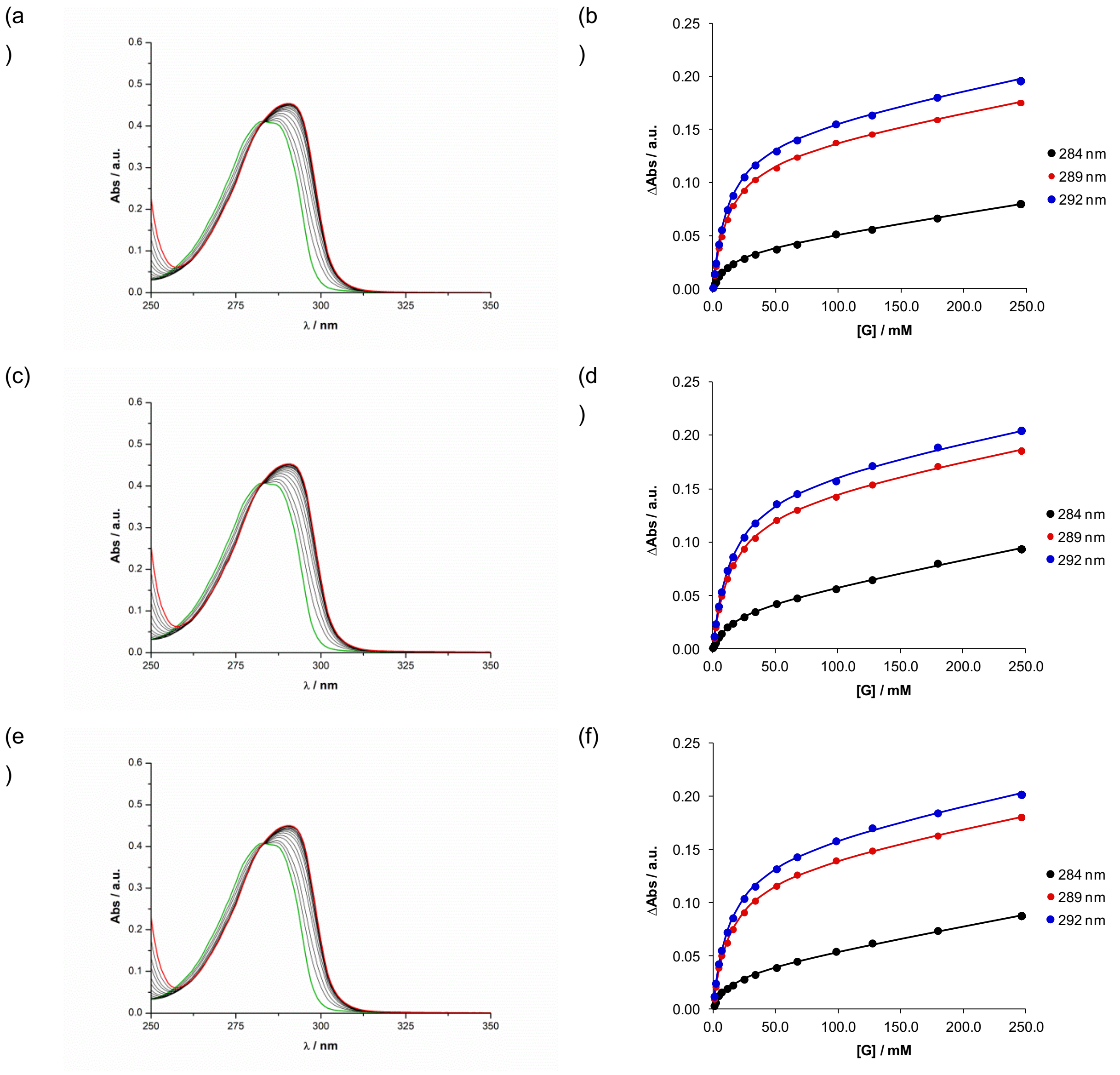

Figure S201 (a, c, e) UV-Vis spectra of **12** (0.100 mM in green) in *n*-octane at increasing concentrations of quinuclidine (from green to red), and (b, d, f) plot of the absorbance of **12** at 284 nm, 289 nm, 292 nm versus the concentration of **12** and its fittings to a 1:1+non-specific binding model.

Molecule 15

Dilution Experiment of 15 in *n*-octane

(a
)

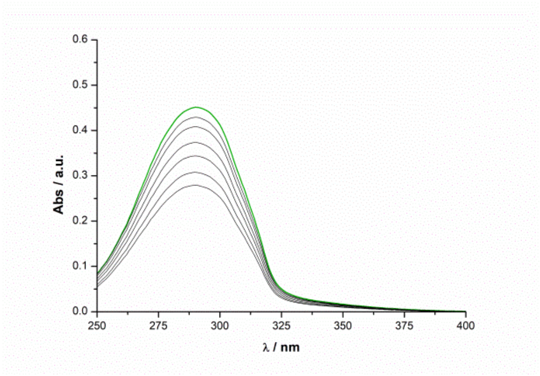

(b
)

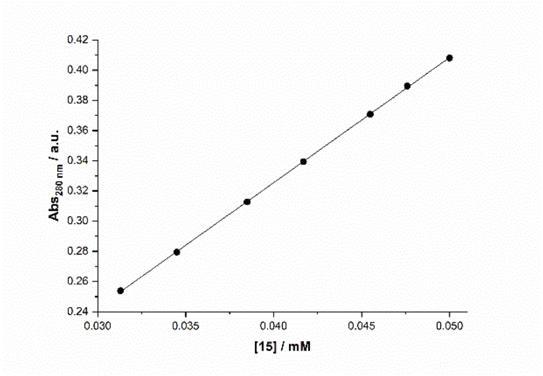

Figure S202 (a) UV-Vis spectra of **15** in *n*-octane at decreasing concentrations (from 0.050 mM in green to 0.031 mM in red), and (b) plot of the absorbance of **15** at 280 nm versus the concentration of **15** and its linear fitting.

Titrations of 15 with quinuclidine in *n*-octane

(a)

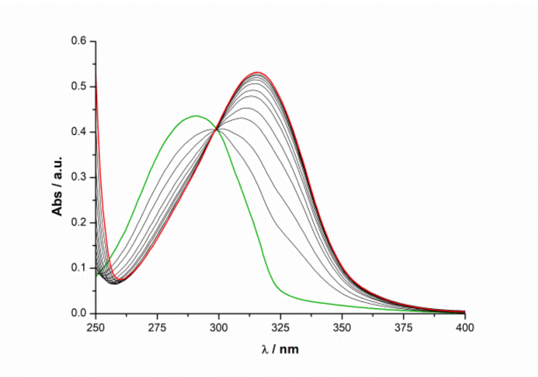

(b)

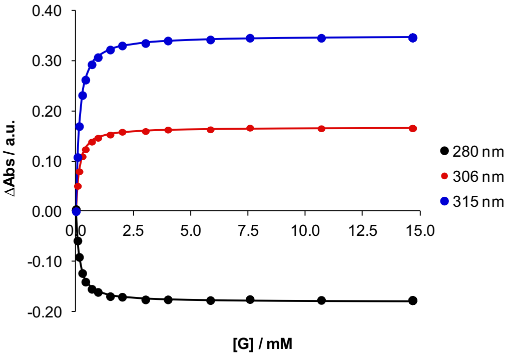

(c)

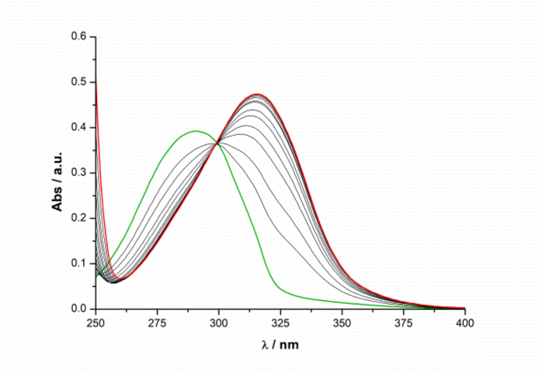

(d)

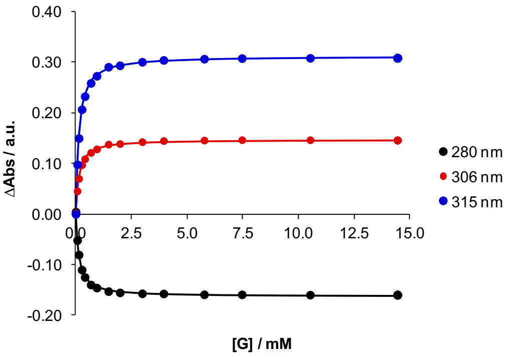

(e)

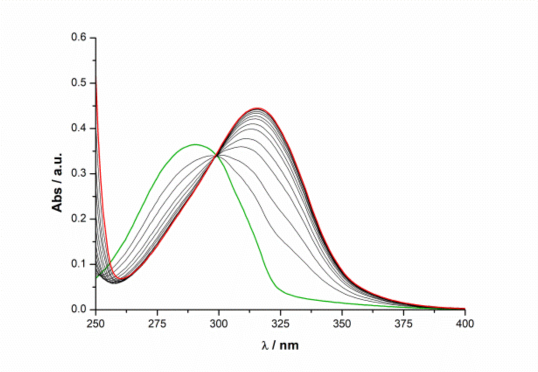

(f)

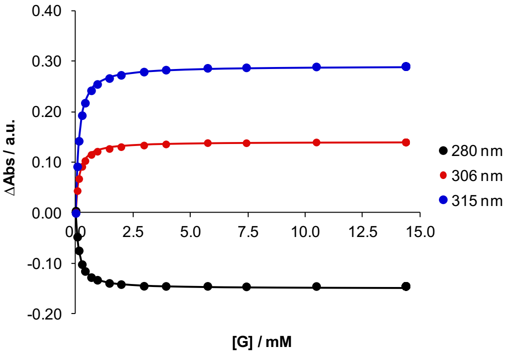

Figure S203 (a, c, e) UV-Vis spectra of **15** (0.050 mM in green) in *n*-octane at increasing concentrations of quinuclidine (from green to red), and (b, d, f) plot of the absorbance of **15** at 280 nm, 306 nm, 315 nm versus the concentration of **15** and its fittings to a 1:1 binding model.

Molecule 16

Dilution Experiment of 16 in *n*-octane

(a)

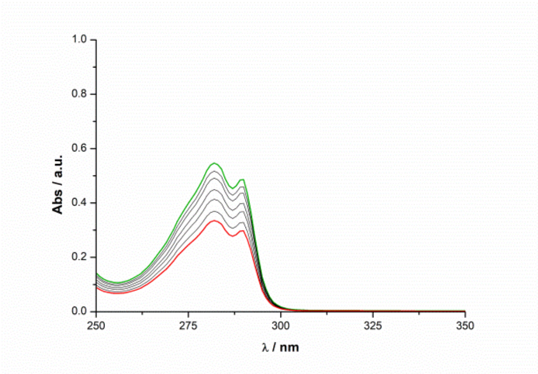

(b)

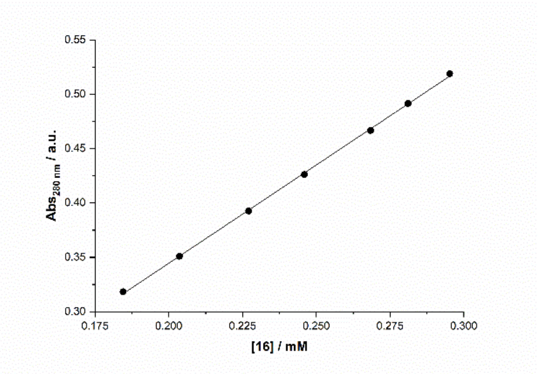

Figure S204 (a) UV-Vis spectra of **16** in *n*-octane at decreasing concentrations (from 0.295 mM in green to 0.185 mM in red), and (b) plot of the absorbance of **16** at 280 nm versus the concentration of **16** and its linear fitting.

Titrations of 16 with quinuclidine in *n*-octane

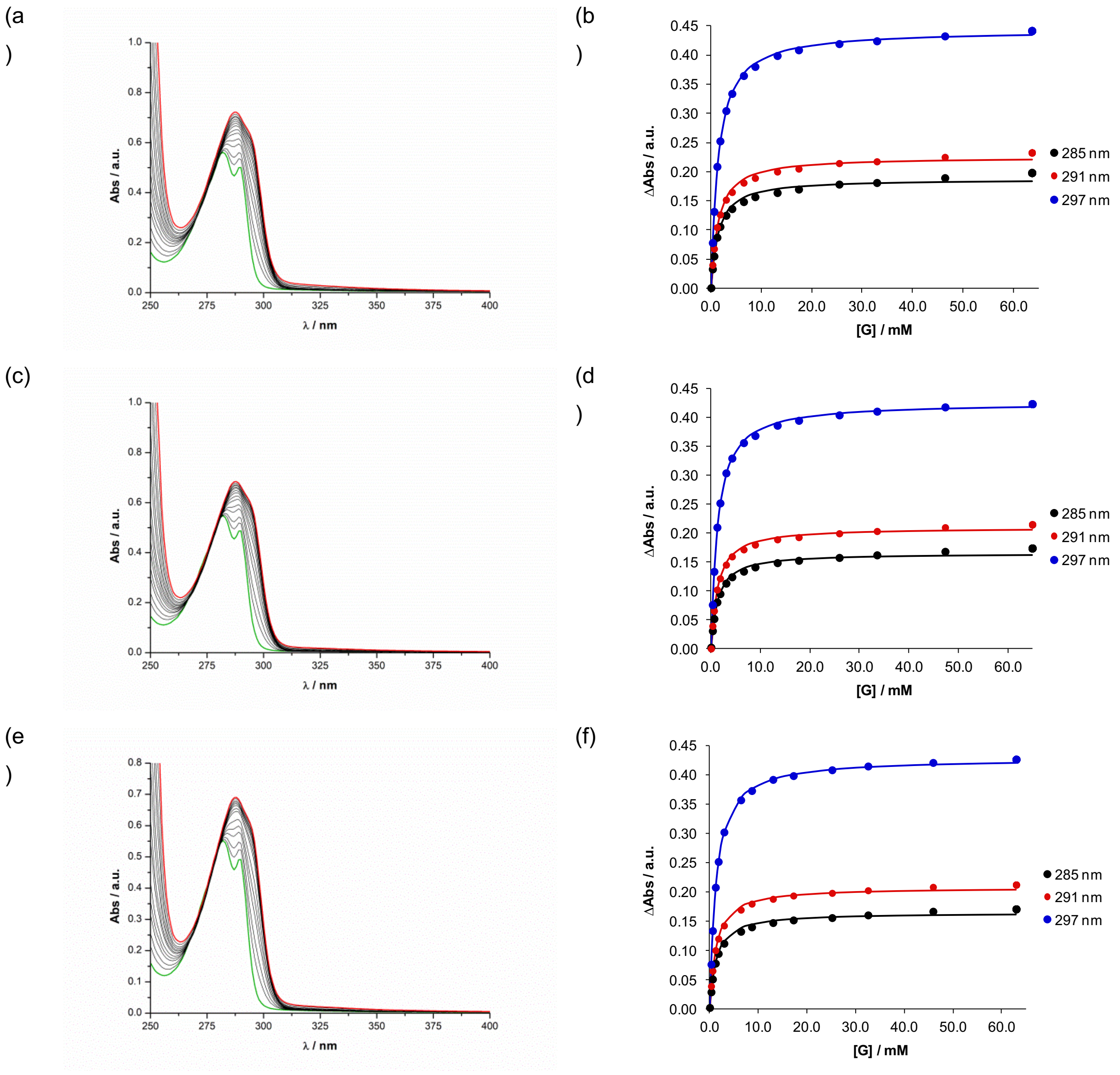

Figure S205 (a, c, e) UV-Vis spectra of **16** (0.295 mM in green) in *n*-octane at increasing concentrations of quinuclidine (from green to red), and (b, d, f) plot of the absorbance of **16** at 285 nm, 291 nm, 297 nm versus the concentration of **16** and its fittings to a 1:1 binding model.

Molecule 17

Dilution Experiment of 17 in *n*-octane

(a
)

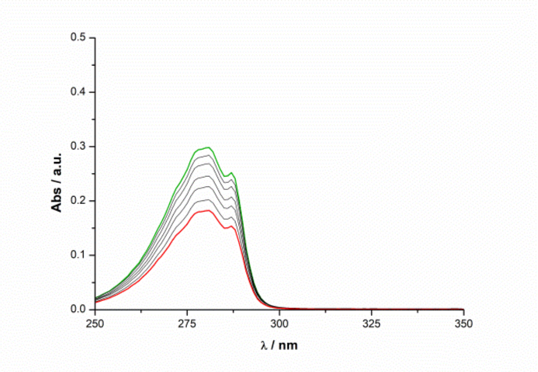

(b
)

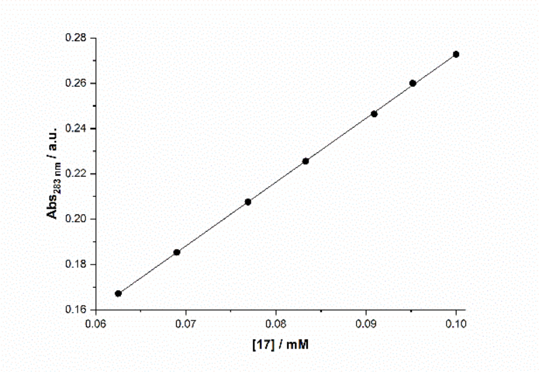

Figure S206 (a) UV-Vis spectra of **17** in *n*-octane at decreasing concentrations (from 0.100 mM in green to 0.063 mM in red), and (b) plot of the absorbance of **17** at 283 nm versus the concentration of **17** and its liear fitting.

Titrations of 17 with quinuclidine in *n*-octane

(a)

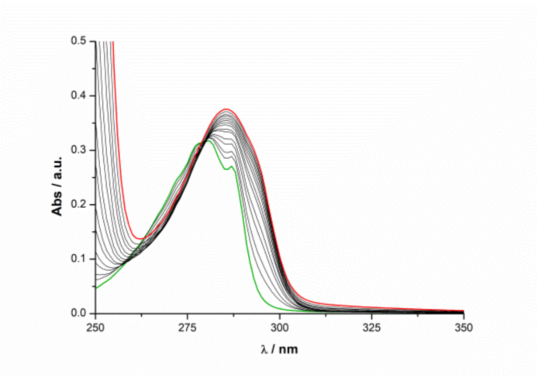

(b)

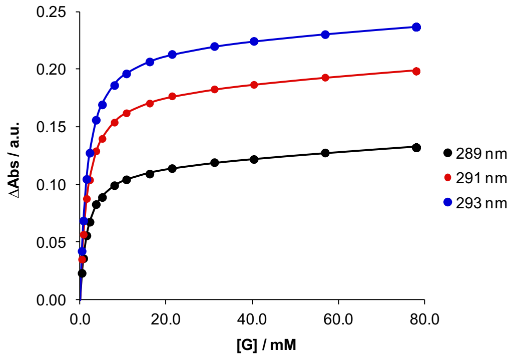

(c)

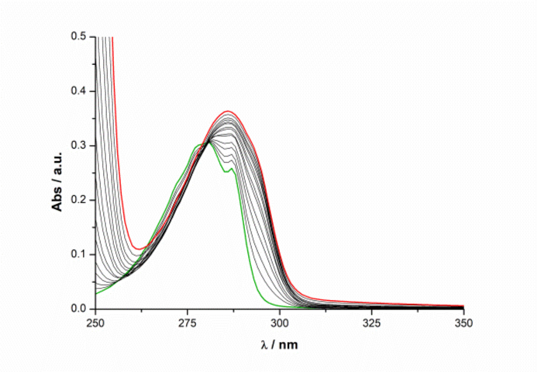

(d)

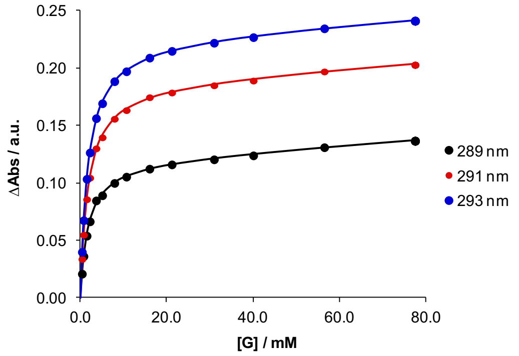

(e)

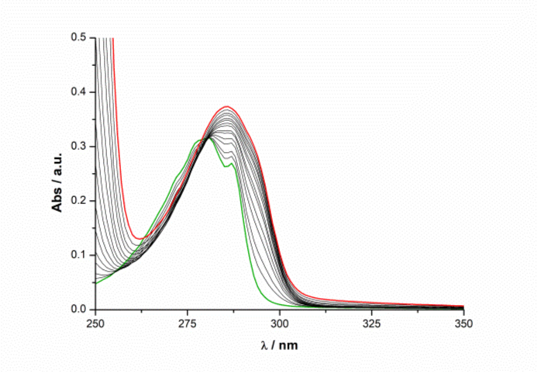

(f)

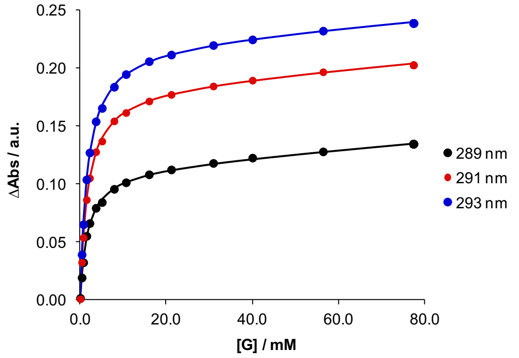

Figure S207 (a, c, e) UV-Vis spectra of **17** (0.100 mM in green) in *n*-octane at increasing concentrations of quinuclidine (from green to red), and (b, d, f) plot of the absorbance of **17** at 289 nm, 291 nm, 293 nm versus the concentration of **17** and its fittings to a 1:1+non-specific binding model.

Molecule 43

Dilution Experiment of 43 in *n*-octane

(a) (b)

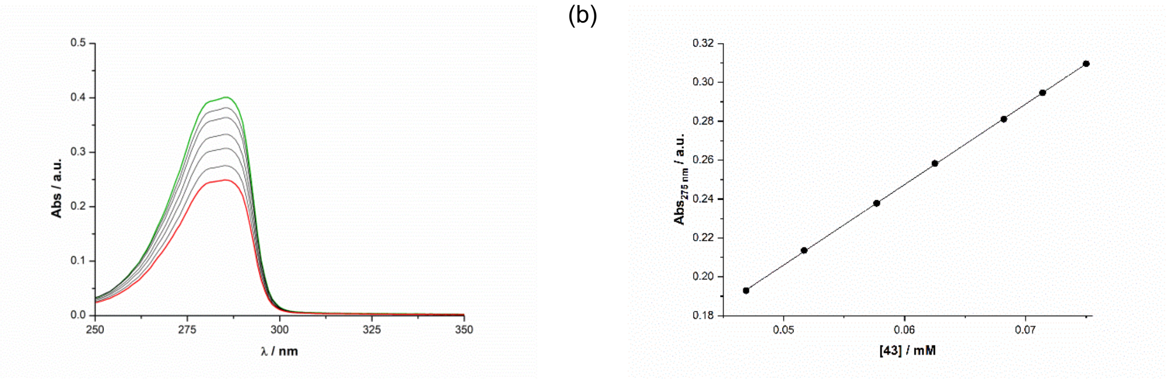

Figure S208 (a) UV-Vis spectra of **43** in *n*-octane at decreasing concentrations (from 0.075 mM in green to 0.047 mM in red), and (b) plot of the absorbance of **43** at 275 nm versus the concentration of **43** and its linear fitting.

Titrations of 43 with quinuclidine in *n*-octane

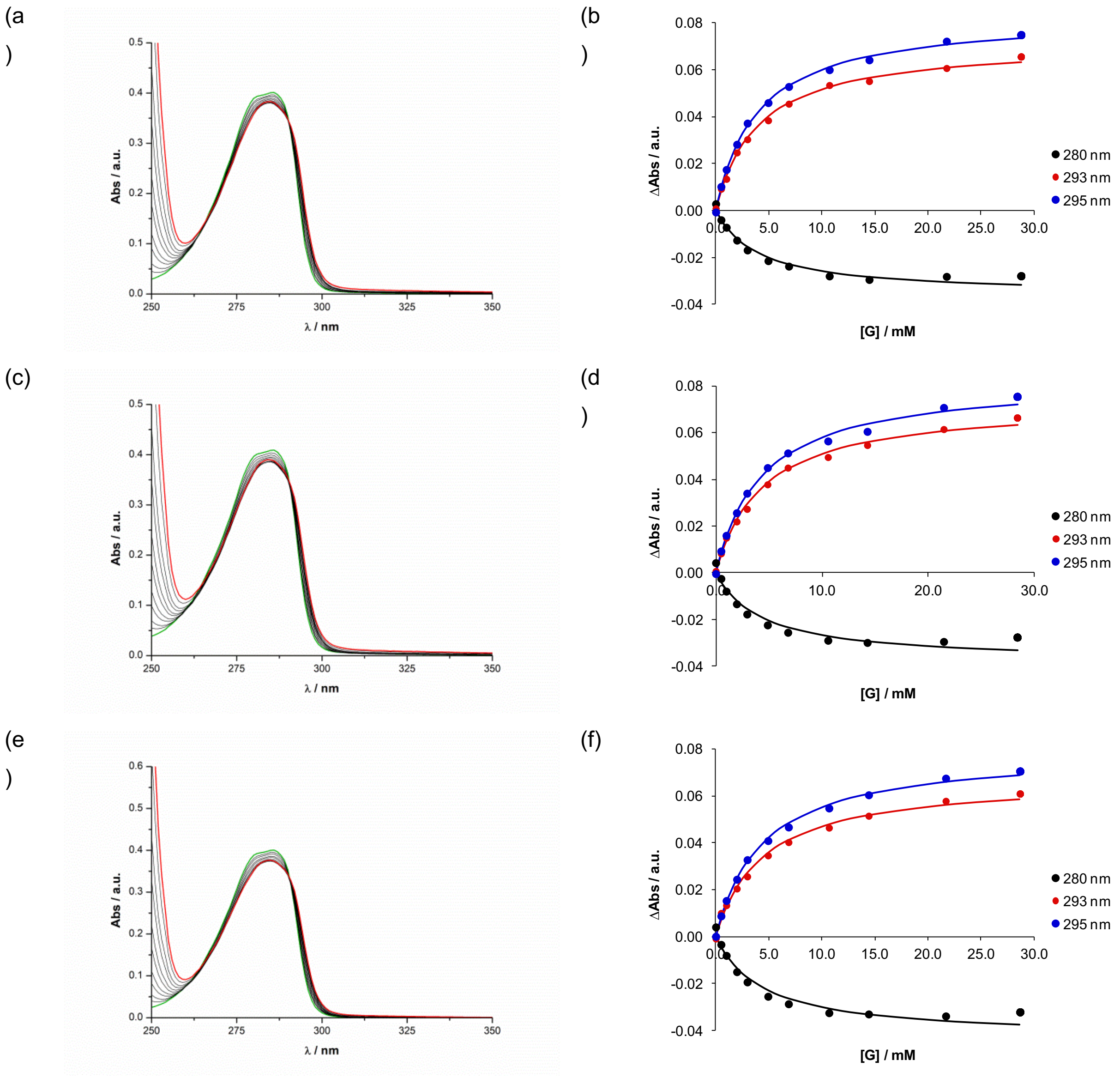

Figure S209 (a, c, e) UV-Vis spectra of **43** (0.075 mM in green) in *n*-octane at increasing concentrations of quinuclidine (from green to red), and (b, d, f) plot of the absorbance of **43** at 280 nm, 293 nm, 295 nm versus the concentration of **43** and its fittings to a 1:1 binding model.

7. Details of Computational Study

Molecular mechanics calculations were done using Schrödinger's Maestro software (2019-1 edition), with $CHCl_3$ as the solvent and OPLS 2005 as the force field (charges assigned from the force field, the cut-off was none so that all non-bonded interactions are considered). The minimization method that was used was PRCG (Polak-Ribier Conjugate Gradient)[18] with a maximum iterations number of 10000, the convergence criterion was a gradient with a convergence threshold of 0.01 kJ mol^{-1} $Å^{-1}$. For each conformational search mixed torsional/low mode sampling was used as the method, the maximum number of steps was 10000, the number of structures saved for each search was 50 and the energy window for saving structures was 50.0 kJ mol^{-1}.

Starting from the structure with the lowest energy and the desired conformation (i.e. with the intramolecular H-bonds present) calculated with molecular mechanics, all the molecules were footprinted as described previously.[19]

	α							
**-X/Y**	**1-5**		**6-9**		**10-14**		**15-19**	
	exp	calc	exp	calc	exp	calc	exp	calc
$-NO_2$	5.3	5.4	5.4	6.0	3.7	4.4	4.6	5.1
-Br	4.8	5.0	5.0	5.3	3.3	3.9	3.9	4.4
-F	4.8	4.9	4.8	5.1	3.2	3.5	3.8	4.1
$-CH_3$	4.6	4.7	-	-	2.8	3.4	3.5	3.7
$-NMe_2$	4.6	4.6	4.5	4.3	2.7	3.0	3.4	3.4

Table S4 H-bond donor parameters (α).

	α	
	exp	calc
**43**	3.5	3.3

Table S5 H-bond donor parameters (α) of other control compounds.

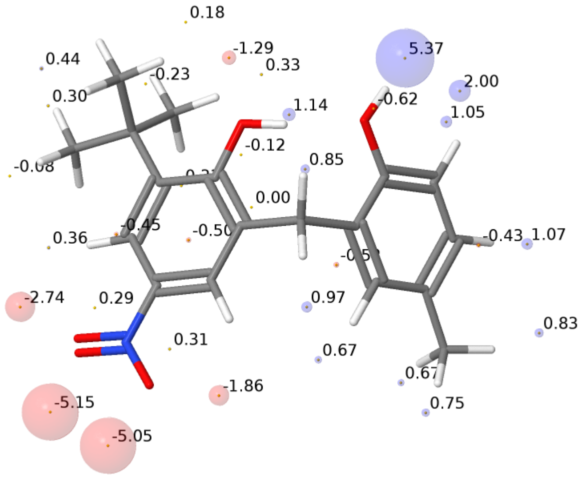

1

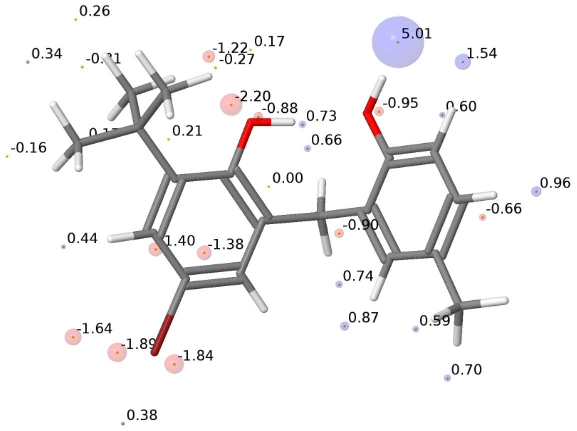

2

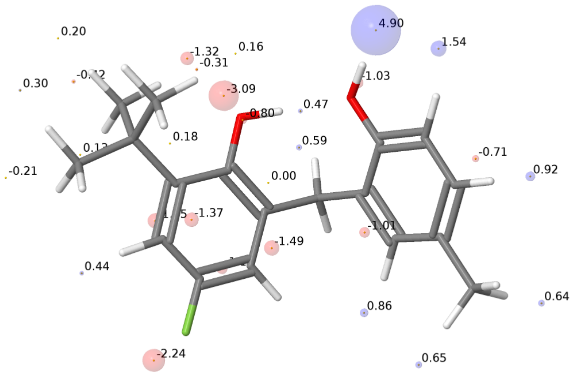

3

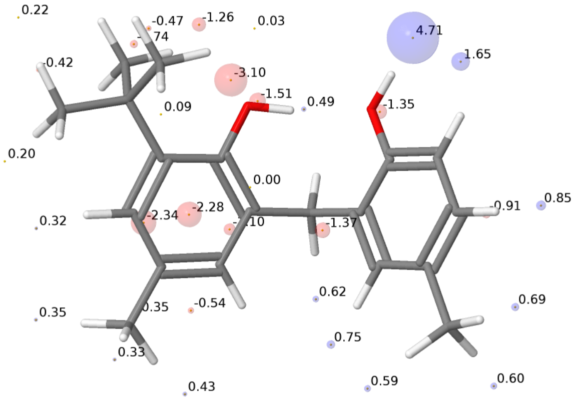

4

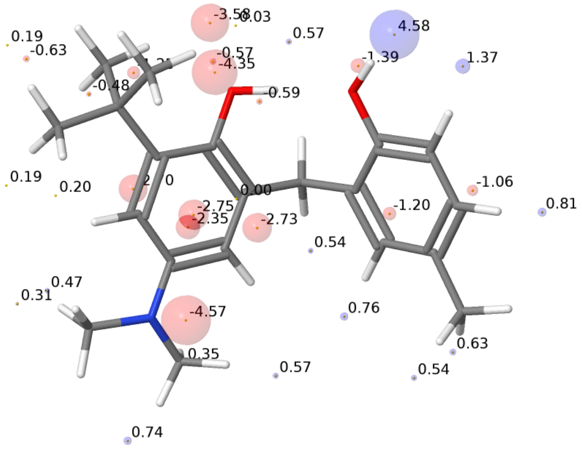

5

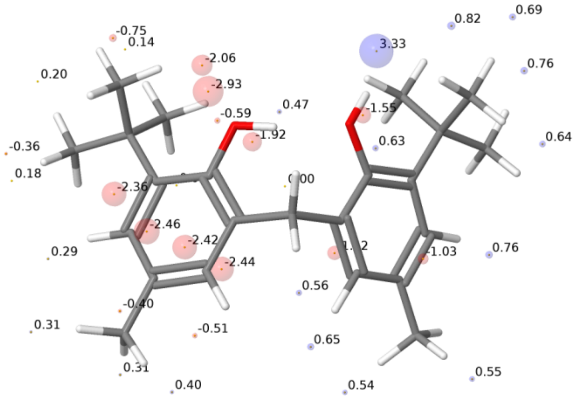

43

Figure S210 Surface site interaction points (SSIPs) for **1-5**, **43**.

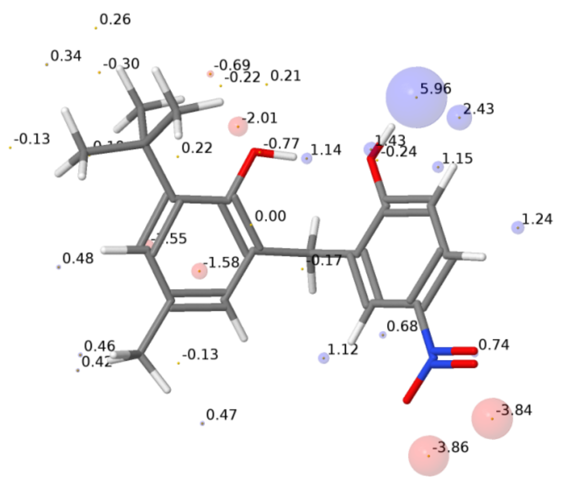

6

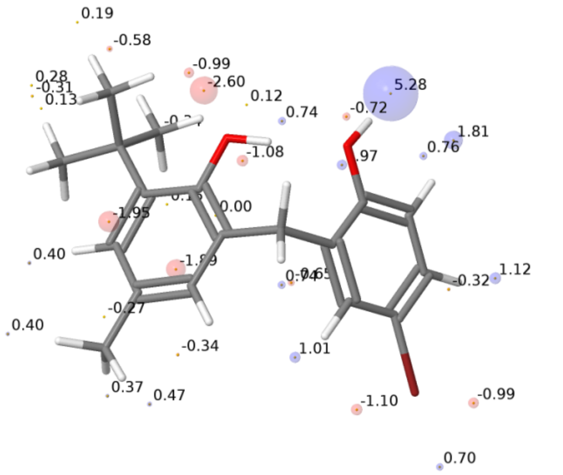

7

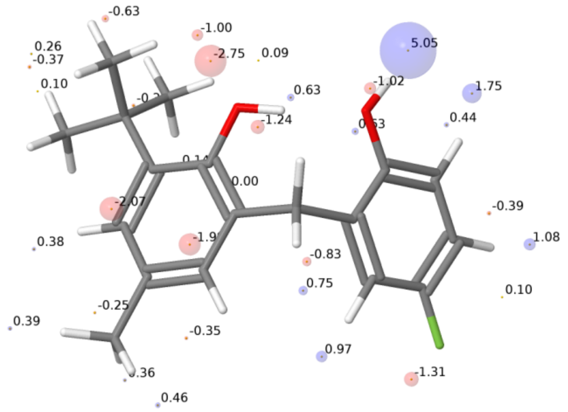

8

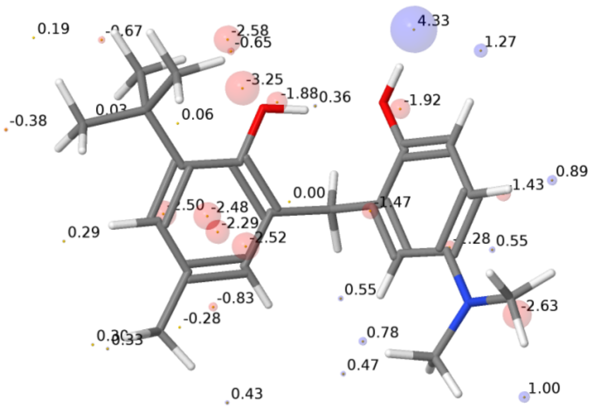

9

Figure S211 Surface site interaction points (SSIPs) for **6**-**9**.

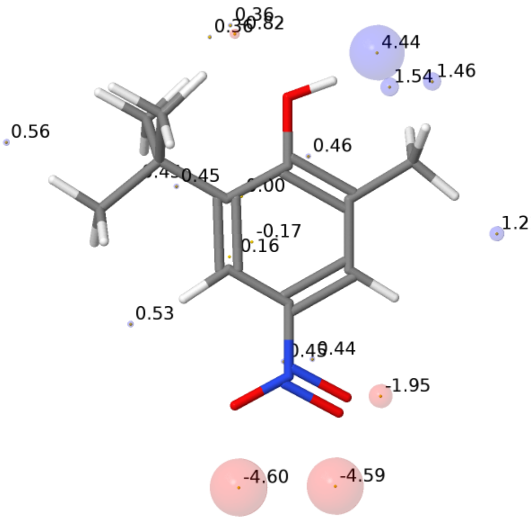

10

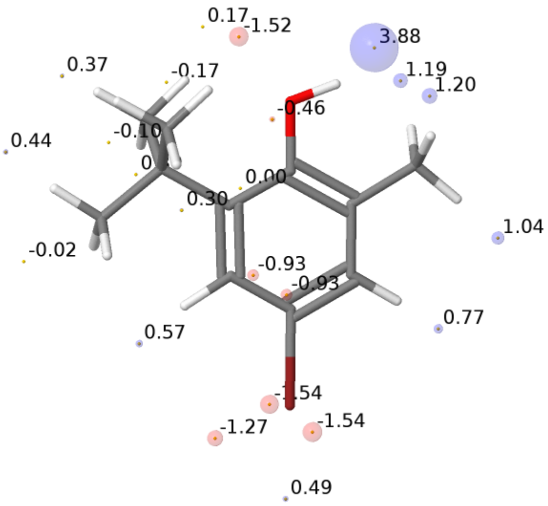

11

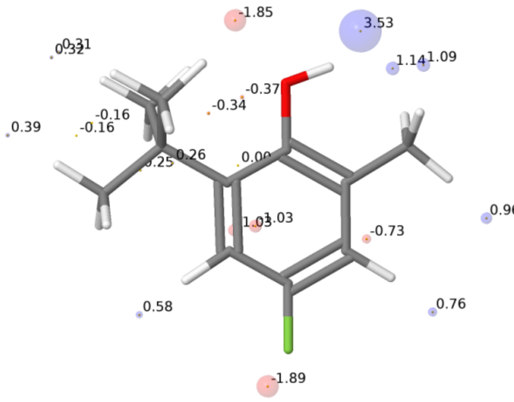

12

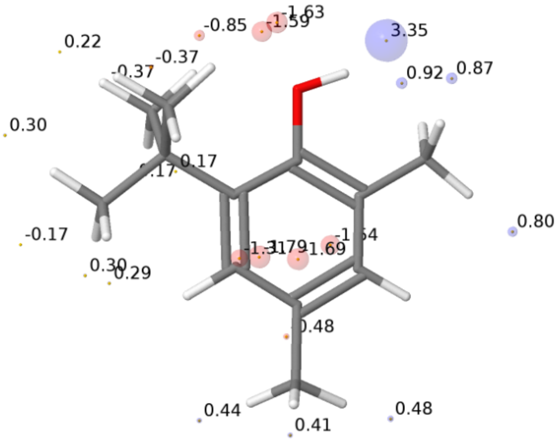

13

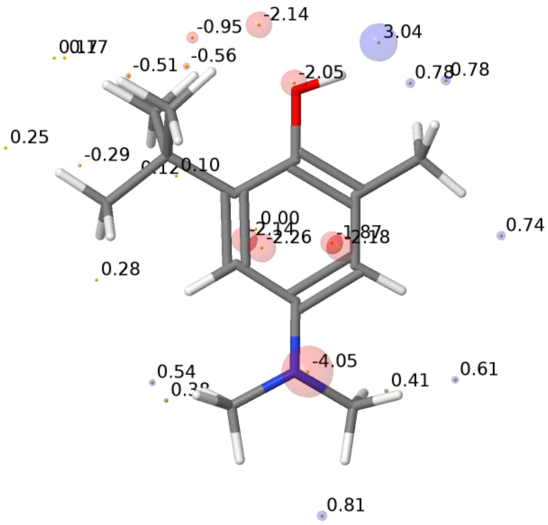

14

Figure S212 Surface site interaction points (SSIPs) for **10-14**.

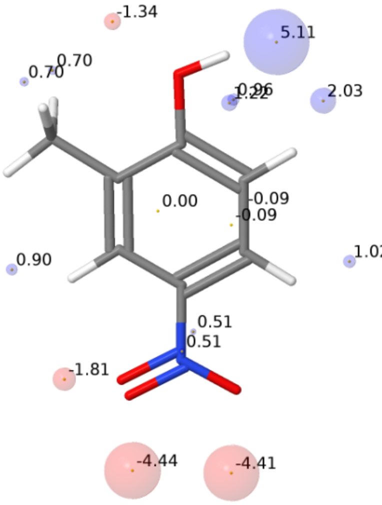

15

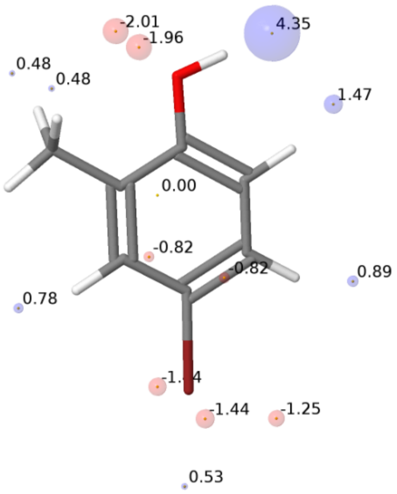

16

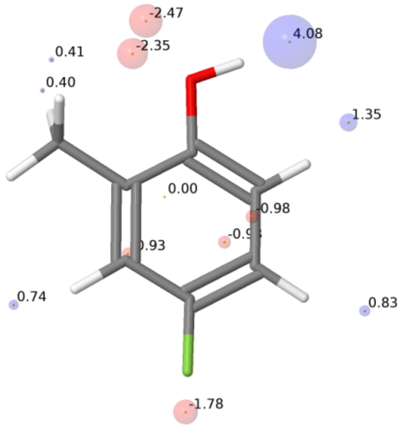

17

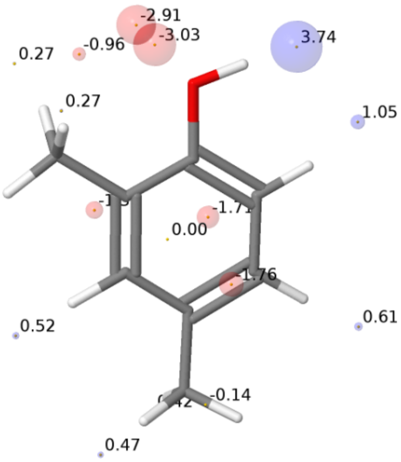

18

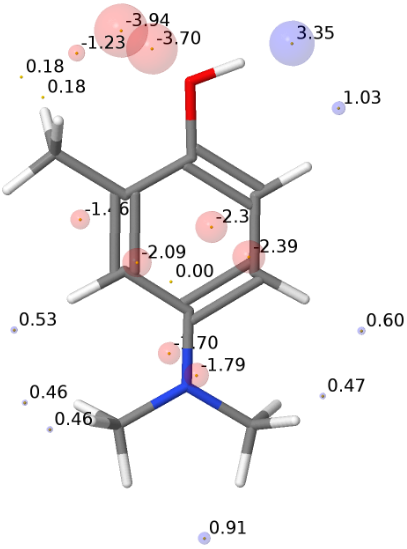

19

Figure S213 Surface site interaction points (SSIPs) for **15-19**.

8. References

1. Fulmer, G. R.; Miller, A. J.; Sherden, N. H.; Gottlieb, H. E.; Mudelman, A.; Stoltz, B. M.; Bercaw, J. E.; Goldberg, K. I. NMR Chemical Shifts of Trace Impurities: Common Laboratory Solvents, Organics, and Gases in Deuterated Solvents Relevant to the Organometallic Chemist. *Organometallics* **2010**, *29* (9), 2176-2179.
2. Gisch, N.; Balzarini, J.; Meier, C. Enzymatically Activated *cyclo*Sal-d4T-monophosphotates: The Third Generation of *cyclo*Sal-Pronucleotides. *Journal of Medicinal Chemistry* **2007**, *50* (7), 1658-1667.
3. Chen, C.-T.; Kao, J.-Q.; Salunke, S. B.; Lin, Y.-H. Enantioselective Aerobic Oxidation of α-Hydroxy-Ketones Catalyzed by Oxidovanadium(V) Methoxides Bearing Chiral, *N*-Salicylidene-*tert*-butylglycinates. *Org. Lett.* **2011**, *13* (1), 26-29.
4. Gal'bershtam, M. A.; Loseva, M. V.; Khrolova, O. R.; Bobyleva, G. K. Synthesis and photochromic properties of indolinospirochromenes with branched alkyl substituents in the 8' position. *Chemistry of Heterocyclic Compounds* **1979**, *15*, 1101-1103.
5. Stasiw, D. E.; Luke, A. M.; Rosen, T.; League, A. B.; Mandal, M.; Neisen, B. D.; Cramer, C. J.; Kol, M.; Tolman, W. B. Mechanism of the Polymerization of *rac*-Lactide by Fast Zinc Alkoxide Catalysts. *Inorg. Chem.* **2017**, *56* (22), 14366-14372.
6. Sartori. G.; Bigi, F.; Maggi, R.; Porta, C. Metal-template *ortho*-regioselective mono and bis-de-*tert*-butylation of poly-*tert*-butylated phenols. *Tetrahedron Lett.* **1994**, *35*, 7073-7076.
7. Bartocci, S.; Sabaté, F.; Mihan, F. Y.; Bosque, R.; Rodríguez, L.; Dalla Cort, A. Novel uranyl(VI) complexes incorporating ethynyl groups as potential halide chemosensors: an experimental and computational approach. *Supramolecular Chemistry* **2017**, *29* (11), 922-927.
8. Wang, J.-F; Xu, X.; Bian, R.-N.; Dong, W.-K.; Ding, Y.-J. Investigation on structurally different Cu(II) and Ni(II) complexes constructed from a novel pyridine-terminal salamo-like ligand. *Inorganica Chimica Acta* **2021**, *516*, 120095.
9. Ingenfeld, B.; Straub, S.; Frömbgen, C.; Lützen, A. Synthesis of Monofunctionalized Calix[5]arenes. *Synthesis* **2018**, *50*, 676-684.
10. DiCiccio, A. M.; Longo, J. M.; Rodríguez-Calero, G. G.; Coates, G. W. Development of Highly Active and Regioselective Catalysts for the Copolymerization of Epoxides with Cyclic Anhydrides: An Unanticipated Effect of Electronic Variation. *J. Am. Chem. Soc.* **2016**, *138* (22), 7107-7113.

11. Larrow, J. F.; Jacobsen, E. N.; Gao, Y.; Hong, Y.; Nie, X.; Zepp, C. M. A Practical Method for the Large-Scale Preparation of [N,N'-Bis(3,5-di-*tert*-butylsalicylidene)-1,2-cyclohexanediaminato(2-)]manganese(III) Chloride, a Highly Enantioselective Epoxidation Catalyst. *J. Org. Chem.* **1994**, *59*, 1939-1942.
12. Menage, S.; Gellon, G.; Pierre, J.-L.; Zurita, D.; Saint-Aman, E. A Class of Ligands Designed as Model for Apogalactose Oxidase. *Bulletin de la Societe Chimique de France* **1997**, *134* (8-9), 785-792.
13. Sheldrick, G. M. SHELXT – Integrated Space-group and Crystal-structure Determination. *Acta Cryst. Sect. A* **2015**, *71*, 3-8.
14. Sheldrick, G. M. Crystal Structure Refinement with SHELXL. *Acta Cryst. Sect. C*, **2015**, *71*, 3-8.
15. Smallcombe, S. H.; Patt, S. L.; Keifer, P. A. WET solvent Suppression and Its Applications to LC NMR and High-Resolution NMR Spectroscopy. *J. Magn. Reson., Ser. A* **1995**, *117*, 295-303.
16. Hoult, D. I. Solvent Peak Saturation with Single Phase and Quadrature Fourier Transformation. *J. Magn. Reson.* **1976**, *21* (2), 337-347.
17. Anslyn, V. E.; Dougherty, D. A. *Modern Physical Organic Chemistry*; University Science Books, 2005, pp 216-221.
18. Polak, E.; Ribiere G. Note sur la Convergence de Méthodes de Directions Conjuguées. Revue Française d'Informatique et de Recherche Opérationelle. Série rouge, **1969**, *16*, 35-43.
19. Calero, C. S.; Farwer, J.; Gardiner, E. J.; Hunter, C. A.; Mackey, M.; Scuderi, S.; Thompson, S.; Vinter, J. G. Footprinting Molecular Electrostatic Potential Surfaces for Calculation of Solvation Energies. *Phys. Chem. Chem. Phys.* **2013**, *15*, 18262-18273.
